# Oxo-Carotenoids as Efficient Superoxide Radical Scavengers

**DOI:** 10.3390/antiox11081525

**Published:** 2022-08-05

**Authors:** Gaosheng Shi, Hyein Kim, Sangho Koo

**Affiliations:** 1 Department of Energy Science and Technology, Myongji University, Myongji-Ro 116, Yongin 17058, Gyeonggi-Do, Korea; 2 Department of Chemistry, Myongji University, Myongji-Ro 116, Yongin 17058, Gyeonggi-Do, Korea

**Keywords:** antioxidant, apocarotenal, carotenoid, oxo-carotenoid, superoxide radical

## Abstract

Oxo-carotenoids containing conjugated carbonyl groups in their chains were designed to be more efficient superoxide radical scavengers than natural carotenoids, β-carotene and canthaxanthin. A practical chain-extension method for polyene dials (e.g., crocetin dial) was also proposed based on Horner–Wadsworth–Emmons olefination. Double aldol condensation between polyene dials and acetophenones with ring substituents produced oxo-carotenoids with substituted benzene rings. The antioxidant activity of oxo-carotenoids was measured using DPPH (radical) and ABTS (cationic radical) scavenging assays and compared with the analysis with the superoxide (anionic radical) probe. An effective conjugation length by carbon–carbon double bonds is important to provide superior antioxidant activity for oxo-carotenoids, regardless of the type of radical probe used in the assay. Increasing electron density is favorable to strong antioxidant activity in DPPH, and the phenol group is favored in ABTS, whereas electron deficient oxo-carotenoids are very potent in the superoxide radical assay. All oxo-carotenoids exhibited 105~151% better superoxide radical scavenging activity compared to beta-carotene (100%), whereas 38~155% in DPPH and 16~96% in ABTS radical scavenging activities were observed.

## 1. Introduction

Carotenoids are important secondary metabolites produced from microalgae to higher plants for energy production in photosynthesis and self-defense such as antibacterial and antioxidant activities [1]. The conjugated polyene chain plays vital roles in light-harvesting [2,3], energy-transference [4,5], and radical-quenching [6]. Provided as a dietary source of fruits and vegetables, human carotenoids benefit healthy living by reducing the risk of disease and aging by eliminating reactive oxygen species (ROS) that cause lipid peroxidation, DNA mutations, protein defects and other oxidative damages [7,8,9]. ROS includes hydroxy radical (HO•), hydroxide (HO^−^), triplet oxygen (^3^O_2_), hydroperoxide (HOO^−^), peroxide (O_2_^−^), and superoxide radical (O_2_^−^^·^), among which superoxide radical is a precursor to most other ROS [10,11,12]. It is the reactivity of the polyene chain towards ROS that exhibits the antioxidant activity of carotenoids [6].

Carotenoids readily undergo one-electron oxidation by photoionization or chemical oxidation to afford carotenoid cationic radical, which is known as a key intermediate in the radical scavenging process by electron transfer [13]. The maximum absorption wavelengths in benzene are 920 nm (astaxanthin), 940 nm (canthaxanthin), 1000 nm (zeaxanthin), 1020 nm (β-carotene), and 1050 nm (lycopene), which are red-shifted from those of the parent carotenoids [14]. The energy gap between HOMO and LUMO of the cationic radicals is the smallest for lycopene and the largest for astaxanthin. The reduction potentials of carotenoid cationic radicals are similar in the range of 1020 ± 40 mV, but the relative ease of electron-transfer to the carotenoids is in the order of astaxanthin > canthaxanthin > zeaxanthin > β-carotene > lycopene [14]. Oxo-carotenoids are not considered as good reducing (radical) agents compared with simple carotenoids. It is reported that carbonyl groups indeed increase the reduction potential of the carotenoids [15,16].

**Figure 1:**
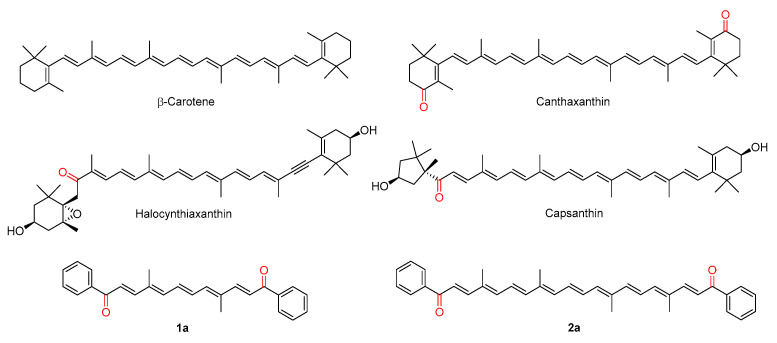
The structures of b-carotene, oxo-carotenoids at the ring or at the chain, and novel oxo-carotenes **1a** and **2a**, designed for superoxide radical scavenging assay.

The efficacy of antioxidant activity of carotenoids is often measured by standard radical scavenging assays utilizing 2,2-diphenyl-1-picrylhydrazyl (DPPH) [17,18,19] or 2,2′-azino-bis(3-ethylbenzothiazoline-6-sulfonic acid) (ABTS) radicals [20]. DPPH radical readily absorbs H• at the allylic positions including methyl groups of carotenoids to produce carotenoid neutral radical. ABTS cationic radical, on the other hand, takes a single electron from the polyene of carotenoids to yield the resonance-stabilized carotenoid cationic radical. Quantitative measurements of the reduction in UV absorption for the above standard radicals by carotenoids represent the antioxidant activities of the carotenoids. These assays work very well for simple carotenoids such as β-carotene and lycopene [21]. However, antioxidant activities of oxo-carotenoids such as canthaxanthin, halocynthiaxanthin, and capsanthin as shown in Figure 1 may be underestimated by these standard radical assays [22,23]. The electron-deficient carotenoids with conjugated carbonyl groups would destabilize the resulting carotenoid (cationic) radicals, and thus the loss of H• or a single electron from the polyene would not be a main mechanism of action for antioxidant activity. On the other hand, anionic (or radical) ROS such as superoxide radical (O_2_^−^^·^) or singlet oxygen would readily transfer a single electron or add to the electron deficient oxo-carotenoids to produce the resonance-stabilized oxo-carotenoid (anionic) radicals [24,25,26]. It is thus necessary to use a (anionic) radical ROS scavenging assay for the fair judgement of the antioxidant activities of oxo-carotenoids.

Pursuing the synthesis of strong carotenoid antioxidant, a series of novel ketonic carotenoids (e.g., **1a** and **2a** in Figure 1) were devised and synthesized by a series of chain-extension and aldol condensation protocols. Antioxidant activities of the ketonic carotenoids using standard DPPH and ABTS radical assays were compared with those of superoxide radical scavenging assays (typically detecting formazan conversion from nitroblue tetrazolium) [27,28,29]. The biggest obstacle in the study of antioxidant activity of the ketonic carotenoids is the fact that their UV absorption wavelengths (496~551 nm) mostly coincide with those of probe molecules such as DPPH radical (517 nm) and formazan (562 nm). This problem can be avoided by separating the probe molecule from ketonic carotenes (or from any other interferences) by HPLC [30] or by choosing a different orthogonal probe system [31]. Herein, we report the details of our studies on the syntheses and anti-oxidant activities of novel oxo-carotenoids.

## 2. Materials and Methods

Reactions were performed in a well-dried flask under argon atmosphere unless noted otherwise. Solvents for extraction and chromatography were reagent grade and used as received. Column chromatography was performed with silica gel 60 (70–230 mesh) using a mixture of EtOAc/hexane as eluent. ^1^H- and ^13^C-NMR spectra were, respectively, recorded on a 400 MHz and 100 MHz NMR spectrometer in deuterated chloroform (CDCl_3_) with tetramethylsilane (TMS) as an internal reference unless noted otherwise.

2-(4-Chlorobut-2-en-2-yl)-5,5-dimethyl-1,3-dioxane (**4**) [32]: Following the previously published procedure, (*E*)-4-chloro-2-methylbut-2-enal (30.1 g, 0.25 mol) was prepared in an 86% overall yield from isoprene (50 mL, 0.50 mol) and *N*-bromosuccinimide (70.0 g, 0.39 mol) through 1-bromo-2-methylbut-3-en-2-ol and isoprene monoxide in 3 steps [32]. The protection of aldehyde (10.0 g, 84 mmol) was performed by the reaction with neopentyl glycol (9.7 g, 92 mmol) and *p*-TsOH (800 mg, 4.2 mmol) in toluene under reflux with a Dean–Stark trap for 3 h to give **4** (8.63 g, 39 mmol) in 66% yield as a 10:1 mixture of *E*/*Z*-isomers with light-yellow oil. Data for **4** are as follows: R_f_ = 0.55 (1:12 acetone/hexane); ^1^H NMR (*E*-isomer) δ = 0.72 (s, 3H), 1.19 (s, 3H), 1.79 (d, *J* = 2.4 Hz, 3H), 3.47 (d, *J* = 11.6 Hz, 2H), 3.64 (d, *J* = 11.6 Hz, 2H), 4.09 (d, *J* = 7.6 Hz, 2H), 4.71 (s, 1H), 5.85 (tq, *J_t_* = 7.6, *J_q_* = 2.4 Hz, 1H) ppm; (*Z*-isomer) δ = 0.73 (s, 3H), 1.20 (s, 3H), 1.84 (d, *J* = 2.4 Hz, 3H), 3.47 (d, *J* = 11.6 Hz, 2H), 3.64 (d, *J* = 11.6 Hz, 2H), 4.16 (dq, *J_d_* = 8.0, *J_q_* = 0.8 Hz, 2H), 5.12 (s, 1H), 5.58 (tq, *J_t_* = 8.0, *J_q_* = 2.4 Hz, 1H) ppm; and ^13^C NMR* δ = 11.4 (18.3), 21.8, 22.9, 30.2, 39.5 (39.4), 77.2, 103.5 (98.6), 124.3 (125.3), 138.2 (138.3) ppm. * *Z*-isomer in parenthesis.

Diethyl (*E*)-(3-(5,5-dimethyl-1,3-dioxan-2-yl)but-2-en-1-yl)phosphonate (**5**) [33]: To a stirred mixture of **4** (12.64 g, 57.8 mmol) and triethyl phosphite (9.60 g, 57.8 mmol), we added NaI (869 mg, 5.80 mmol). The mixture was heated at 120 °C for 12 h and cooled to room temperature. The resulting mixture was purified using SiO_2_ flash column chromatography to produce phosphonate **5** (13.5 g, 44.0 mmol; a 10:1 mixture of *E*/*Z*-isomer) in 76% yield as a clear oil. Data for **5** are as follows: R_f_ = 0.41 (20:1 CH_2_Cl_2_/MeOH); ^1^H NMR (*E*-isomer) δ = 0.68 (s, 3H), 1.16 (s, 3H), 1.26 (t, *J* = 7.2 Hz, 6H), 1.71 (dd, *J* = 4.2, 1.4 Hz, 3H), 2.57 (dd, *J* = 22.4, 8.0 Hz, 2H), 3.43 (d, *J* = 11.6 Hz, 2H), 3.59 (d, *J* = 11.6 Hz, 2H), 3.98–4.13 (m, 4H), 4.69 (s, 1H), 5.60 (dt, *J_d_* = 8.0, *J_t_* = 8.0 Hz, 1H) ppm; ^13^C NMR δ = 11.4 (*J_d_* = 2.3 Hz), 16.4 (*J_d_* = 6.0 Hz), 21.8, 22.9, 26.0 (*J_d_* = 139.5 Hz), 30.1, 61.9 (*J_d_* = 6.6 Hz), 77.1, 104.3, 117.9 (*J_d_* = 10.7 Hz), 137.5 (*J_d_* = 14.2 Hz) ppm; IR (KBr) ν = 2979, 1473, 1393, 1250, 1164, 1104, 1023, 959, 870, 793, 704 cm^−1^; and HRMS (ESI) calcd for C_14_H_27_O_5_P + Na 329.1488, found to be 329.1493.

(*E*)-3-(5,5-Dimethyl-1,3-dioxan-2-yl)but-2-enal (**6**) [34]: To a stirred solution of acetal **4** (0.59 g, 1.93 mmol) in DMSO (10 mL), the following were added: K_2_HPO_4_ (0.40 g, 2.28 mmol), KH_2_PO_4_ (81 mg, 0.60 mmol), and NaI (29 mg, 0.19 mmol). The mixture was heated at 80 °C for 12 h and cooled to room temperature. The mixture was diluted with EtOAc, washed with H_2_O, dried over anhydrous Na_2_SO_4_, filtered, and concentrated under reduced pressure. The crude product was purified using SiO_2_ flash chromatography to produce **6** (0.16 g, 0.85 mmol, a 5:1 mixture of *E*/*Z*-isomers) in 44% yield and unreacted **4** (0.16 g, 0.51 mmol). Data for **6**: R_f_ = 0.25 (1:3 hexane/acetone); ^1^H NMR (*E*-isomer) δ = 0.73 (s, 3H), 1.18 (s, 3H), 2.18 (s, 3H), 3.50 (d, *J* = 10.8 Hz, 2H), 3.66 (d, *J* = 10.8 Hz, 2H), 4.80 (s, 1H), 6.13 (d, *J* = 8.8 Hz, 1H), 10.06 (d, *J* = 8.8 Hz, 1H) ppm; (*Z*-isomer) δ = 0.70 (s, 3H), 1.21 (s, 3H), 2.02 (s, 3H), 3.50 (d, *J* = 10.8 Hz, 2H), 3.66 (d, *J* = 10.8 Hz, 2H), 4.71 (s, 1H), 5.88 (d, *J* = 9.2 Hz, 1H), 10.08 (d, *J* = 9.2 Hz, 1H) ppm; and ^13^C NMR* δ = 12.6(19.4), 21.7 (21.8), 22.9 (23.0), 30.3 (30.2), 77.3 (77.2), 102.2 (98.1), 127.6 (130.1), 155.6 (156.0), 191.6 (190.5) ppm. * *Z*-isomer in parenthesis.

(2*E*,4*E*,6*E*)-2,7-Dimethylocta-2,4,6-trienedial (**1**) [35]: To a stirred solution of phosphonate **5** (0.18 g, 0.61 mmol) in THF (20 mL) we slowly added NaH (0.33 g, 5.53 mmol) at 0 °C. While stirring for 5 min, a solution of aldehyde **6** (0.10 g, 0.55 mmol) in THF (5 mL) was slowly added to the above mixture. The mixture was stirred at 25 °C for 12 h. The mixture was then treated with 1 M HCl solution (10 mL) and stirred at 25 °C for 1 h. The organic phase was extracted with EtOAc, dried over anhydrous Na_2_SO_4_, filtered, and concentrated under reduced pressure. The crude product was purified using SiO_2_ flash column chromatography to produce **1** (75 mg, 0.46 mmol) in 83% yield as a light-yellow solid. Data for **1** are as follows: ^1^H NMR δ = 1.94 (s, 6H), 6.96–7.03 (m, 2H), 7.03–7.10 (m, 2H), 9.54 (s, 2H) ppm.

(2*E*,4*E*,6*E*,8*E*,10*E*,12*E*,14*E*)-2,6,11,15-Tetramethylhexadeca-2,4,6,8,10,12,14-heptaenedial (**2**) [32,36]: To a stirred solution of phosphonate **5** (24.63 g, 80.39 mmol) in a 1:1 mixture of THF/*t*-BuOH (80 mL), we slowly added *t*-BuOK (12.30 g, 0.110 mol) at 0 °C. While stirring for 5 min at 25 °C, a solution of aldehyde **1** (6.00 g, 36.54 mmol) in THF (20 mL) was added to the above mixture. The mixture was stirred at 25 °C for 12 h, and 1 M HCl solution (150 mL) was added. The resulting mixture was stirred at 25 °C for 2 h. The organic phase was extracted with EtOAc, dried over anhydrous Na_2_SO_4_, filtered, and concentrated under reduced pressure. The crude product was purified by performing trituration from Et_2_O twice to obtain all-(*E*)-**2** (10.00 g, 33.74 mmol) in 92% yield as a red solid. Data for **2** are as follows: R_f_ = 0.39 (3:1 hexane:acetone); ^1^H NMR δ = 1.91 (s, 6H), 2.03 (s, 6H), 6.41–6.51 (m, 2H), 6.68–6.75 (m, 4H), 6.75–6.82 (m, 2H), 6.89–6.99 (m, 2H), 9.47 (s, 2H) ppm; ^13^C NMR δ = 9.7, 12.8, 123.7, 132.0, 136.7, 137.1, 137.4, 145.4, 148.8, 194.5 ppm; and UV (2:1 DMSO/CH_2_Cl_2_, c = 0.26 mmol/L): λ (ε) = 480 nm (411,538).

(2*E*,4*E*,6*E*,8*E*,10*E*,12*E*,14*E*,16*E*,18*E*,20*E*,22*E*)-2,6,10,15,19,23-Hexamethyltetracosa-2,4,6,8,10,12,14,16,18,20,22-undecaenedial (**3**) [32,36]: To a stirred solution of phosphonate **5** (1.5 g, 4.9 mmol) in a 1:1 mixture of THF/*t*-BuOH (60 mL), we slowly added *t*-BuOK (2.0 g, 17.8 mmol). Stirring the mixture at 120 °C for 5 min, a solution of aldehyde **2** (0.5 g, 1.69 mmol) in THF (10 mL) was added. The mixture was stirred at 120 °C for 12 h and cooled to 25 °C. A 1 M HCl solution (70 mL) was added to the above solution, and the resulting mixture was stirred at 25 °C for 30 min. The organic phase was extracted with EtOAc, dried over anhydrous Na_2_SO_4_, filtered, and concentrated under reduced pressure. The crude product was first triturated with Et_2_O to produce **3** (0.20 g, 0.47 mmol) as a red solid. The mother liquor was then concentrated and purified by performing SiO_2_ flash column chromatography to obtain **3** (0.39 g, 0.91 mmol). The combined total yield of **3** (0.59 g, 1.38 mmol) was 82%. Data for **3** are as follows: ^1^H NMR δ = 1.91 (s, 6H), 2.01 (s, 6H), 2.03 (s, 6H), 6.31–6.41 (m, 2H, H_11_), 6.44 (d, *J* = 11.2 Hz, 2H, H_7_), 6.50 (d, *J* = 14.8 Hz, 2H, H_9_), 6.65–6.75 (m, 2H, H_12_), 6.68 (dd, *J* = 14.8, 11.2 Hz, 2H, H_8_), 6.70 (dd, *J* = 14.8, 11.2 Hz, 2H, H_4_), 6.75 (d, *J* = 14.8 Hz, 2H, H_5_), 6.94 (d, *J* = 11.2 Hz, H_3_), 9.46 (s, 2H, H_1_) ppm; ^13^C NMR δ = 9.7, 12.8, 12.8, 122.6, 124.9, 131.0, 134.6, 135.3, 136.7, 137.0, 137.6, 140.8, 146.0, 149.3, 194.6 ppm; UV (2:1 DMSO/CH_2_Cl_2_, c = 0.52 mmol/L): λ (ε) = 525 nm (108,637); IR (KBr) ν = 2922, 1668, 1609, 1541, 1405, 1377, 1357, 1319, 1278, 1180, 1001, 971, 827, 759, 691 cm^−1^; and HRMS (EI) calcd for C_30_H_36_O_2_ 428.2715, found to be 428.2715.

All-(*E*)-4,9-dimethyl-1,12-diphenyldodeca-2,4,6,8,10-pentaene-1,12-dione (**1a**): To a stirred solution of C_10_ dialdehyde **1** (0.32 g, 1.95 mmol) and acetophenone (0.7 g, 5.85 mmol) in a mixed solvent of H_2_O (2 mL) and MeOH (20 mL) we added NaOH (0.39 g, 9.7 mmol). The mixture was stirred at 25 °C for 12 h and quenched with 1 M HCl solution. The mixture was extracted with EtOAc, dried over anhydrous Na_2_SO_4_, filtered, and concentrated under reduced pressure. The crude product was purified using SiO_2_ flash chromatography to produce **1a** (0.52 g, 1.41 mmol) in 72% yield as a yellow solid. The all-(*E*) product was obtained by performing recrystallization with Et_2_O and MeOH. Data for **1a** are as follows: R_f_ = 0.58 (2:1 hexane/acetone); ^1^H NMR δ = 2.08 (s, 6H), 6.61–6.71 (m, 2H), 6.79–6.89 (m, 2H), 7.04 (d, *J* = 15.2 Hz, 2H), 7.46–7.51 (m, 4H), 7.50 (d, *J* = 15.2 Hz, 2H), 7.55–7.60 (m, 2H), 7.95–8.00 (m, 4H) ppm; ^13^C NMR δ = 13.0, 121.3, 128.4, 128.6, 132.6, 132.9, 136.8, 138.5, 139.9, 148.5, 190.5 ppm; UV (2:1 DMSO/CH_2_Cl_2_, c = 0.26 mmol/L) λ_max_ (ε) = 441 nm (269,615); IR (KBr) ν = 1644, 1596, 1580, 1561, 1395, 1369, 1319, 1262, 1215, 1185, 1036, 1019, 989, 956, 846, 813, 776, 692, 678 cm^−1^; HRMS (ESI) calcd for C_26_H_24_O_2_ + Na 391.1669, found to be 391.1671.

All-(*E*)-2,7-dimethyl-10-oxo-10-phenyldeca-2,4,6,8-tetraenal (**1a-al**): To a stirred solution of C_10_ dialdehyde **1** (0.20 g, 1.22 mmol) and acetophenone (0.40 g, 3.65 mmol) in THF (20 mL) we added 1M THF solution of NaHMDS (3.7 mL, 3.7 mmol) at −78 °C. The mixture was then stirred at 25 °C for 2 h and quenched with 1 M HCl solution. The mixture was extracted with EtOAc, dried over anhydrous Na_2_SO_4_, filtered, and concentrated under reduced pressure. The crude product was purified by performing SiO_2_ flash chromatography to produce **1a-al** (0.11 g, 0.42 mmol) in 35% yield as a yellow solid. The all-(*E*) product was obtained by performing recrystallization with Et_2_O and MeOH. Data for **1a-al** are as follows: R_f_ = 0.36 (3:1 hexane/acetone); ^1^H NMR δ = 1.91 (s, 3H), 2.11 (s, 3H), 6.66 (d, *J* = 11.6 Hz, 1H), 6.88 (dd, *J* = 14.4, 11.6 Hz, 1H), 6.97 (dd, *J* = 11.6, 1.2 Hz, 1H), 7.04 (dd, *J* = 14.4, 11.6 Hz, 1H), 7.09 (d, *J* = 15.2 Hz, 1H), 7.46–7.53 (m, 2H), 7.52 (d, *J* = 15.2 Hz, 1H), 7.55–7.60 (m, 1H), 7.95–8.00 (m, 2H) 9.50 (s, 1H) ppm; ^13^C NMR δ = 9.8, 13.1, 122.8, 128.4, 128.6, 131.2, 132.8, 136.1, 138.2, 138.6, 138.9, 139.2, 147.5, 148.0, 190.4, 194.5 ppm; and HRMS (ESI) calcd for C_18_H_18_O_2_ + Na 289.1199, found to be 289.1213.

All-(*E*)-4,8,13,17-tetramethyl-1,20-diphenylicosa-2,4,6,8,10,12,14,16,18-nonaene-1,20-dione (**2a**): To a stirred solution of C_20_ dial **2** (0.15 g, 0.50 mmol) and acetophenone (0.17 g, 1.3 mmol) in THF (10 mL) we added 40% solution of triton B in MeOH (0.85 g, 2.0 mmol). The mixture was stirred at 25 °C for 12 h and quenched with 1 M HCl solution. The mixture was extracted with EtOAc, dried over anhydrous Na_2_SO_4_, filtered, and concentrated under reduced pressure. The crude product was purified by SiO_2_ flash column chromatography to give **2a** (0.10 g, 0.20 mmol) in 40% yield as red solid. The all-(*E*) product was obtained by recrystallization from Et_2_O and MeOH. Data for **2a**: R_f_ = 0.35 (3:1 hexane/acetone); ^1^H NMR δ = 2.01 (s, 6H), 2.07 (s, 6H), 6.33–6.43 (m, 2H), 6.56 (d, *J* = 14.4 Hz, 2H), 6.63 (d, *J* = 11.6 Hz, 2H), 6.69 (dd, *J* = 14.4, 11.6 Hz, 2H), 6.66–6.76 (m, 2H), 6.98 (d, *J* = 15.2 Hz, 2H), 7.45–7.51 (m, 4H), 7.51–7.60 (m, 2H), 7.94–7.99 (m, 4H) ppm; ^13^C NMR δ = 12.8, 29.7, 107.1, 115.8, 120.4, 124.7, 128.3, 128.5, 132.4, 134.3, 137.1, 138.7, 141.2, 142.2, 149.3, 190.5 ppm; UV (2:1 DMSO/CH_2_Cl_2_, c = 0.52 mmol/L): λ_max_ (ε) = 496 nm (111,516); IR (KBr) ν = 3053, 3014, 2979, 2931, 1718, 1670, 1597, 1579, 1447, 1362, 1274, 1259, 1215, 1179, 1072, 1017, 1001, 972, 752, 699, 667 cm^−1^; and HRMS (EI) calcd for C_36_H_36_O_2_ 500.2715, found to be 500.2712; HRMS (ESI) calcd for C_36_H_36_O_2_ + Na 523.2608, found to be 523.2610.

All-(*E*)-2,6,11,15-tetramethyl-18-oxo-18-phenyloctadeca-2,4,6,8,10,12,14,16-octaenal (**2a-al**): To a stirred solution of C_20_ dialdehyde **2** (0.15 g, 0.50 mmol) and acetophenone (0.17 g, 1.3 mmol) in MeOH (20 mL) we added NaOH (0.39 g, 9.7 mmol). The mixture was stirred at 70 °C for 12 h then cooled to room temperature. Most of the solvent was removed under reduced pressure, and the crude mixture was treated with 1 M HCl solution (50 mL). The above mixture was then extracted with EtOAc, dried over anhydrous Na_2_SO_4_, filtered, and concentrated under reduced pressure. The crude product was purified by performing SiO_2_ flash chromatography to obtain **2a-al** (0.12 g, 0.29 mmol) in 57% yield as a red solid. The all-(*E*) product was obtained by performing recrystallization with Et_2_O and MeOH. Data for **2a-al** are as follows: R_f_ = 0.41 (3:1 hexane/acetone); ^1^H NMR δ = 1.25 (s, 3H), 1.91 (s, 3H), 2.02 (s, 3H), 2.07 (s, 3H), 6.39 (d, *J* = 10.0 Hz, 1H), 6.45 (d, *J* = 10.0 Hz, 1H), 6.56 (d, *J* = 14.4 Hz, 1H), 6.63 (d, *J* = 11.4 Hz, 1H), 6.69 (dd, *J* = 14.4, 11.4 Hz, 1H), 6.67–6.75 (m, 2H), 6.77 (dd, *J* = 14.4, 11.6 Hz, 1H), 6.94 (d, *J* = 10.0 Hz, 1H), 6.69 (dd, *J* = 14.4, 11.6 Hz, 1H), 6.98 (d, *J* = 15.2 Hz, 1H), 7.45–7.52 (m, 2H), 7.52–7.59 (m, 1H), 7.55 (d, *J* = 15.2 Hz, 1H), 7.94–8.00 (m, 2H), 9.46 (s, 1H) ppm; ^13^C NMR δ = 9.7, 12.8, 12.9, 29.7, 120.6, 123.3, 125.2, 128.3, 128.5, 130.9, 132.5, 134.3, 134.6, 134.9, 135.2, 136.4, 137.1, 137.8, 138.7, 141.0, 142.0, 145.7, 149.1, 149.2, 190.6, 194.6 ppm; and HRMS (ESI) calcd for C_28_H_30_O_2_ + Na 421.2138, found to be 421.2142.

All-(*E*)-4,8,12,17,21,25-hexamethyl-1,28-diphenyloctacosa-2,4,6,8,10,12,14,16,18,20,22,24,26-tridecaene-1,28-dione (**3a**): Following the general procedure for **2a**, the reaction of C_30_ dial **3** (0.30 g, 0.70 mmol) and acetophenone (0.25 g, 2.10 mmol) with 40% solution of triton B in MeOH (0.59 g, 3.50 mmol) in THF (10 mL) at 25 °C for 12 h provided all-(*E*)-**3a** (0.11 g, 0.17 mmol) in 25% yield as a black-red solid. The all-(*E*) product was obtained by recrystallization from Et_2_O and MeOH. Data for **3a** are as follows: R_f_ = 0.35 (3:1 hexane/acetone); ^1^H NMR (CDCl_3_) δ = 2.00 (s, 6H), 2.02 (s, 6H), 2.06 (s, 6H), 6.30–6.75 (m, 12H), 6.36 (d, *J* = 11.6 Hz, 2H), 6.46 (d, *J* = 14.4 Hz, 2H), 6.97 (d, *J* = 16.0 Hz, 2H), 7.55 (d, *J* = 16.0 Hz, 2H), 7.45–7.52 (m, 4H), 7.53–7.60 (m, 2H), 7.95–8.00 (m, 4H) ppm; UV (CHCl_3_, c = 0.065 mmol/L): λ (ε) = 550 nm (153,574); IR (KBr) ν = 2922, 2853, 1729, 1650, 1570, 1532, 1458, 1376, 1215, 962, 762 cm^−1^; and HRMS (FAB) calcd for C_46_H_48_O_2_ 632.3654, found to be 632.3658.

DPPH radical assay [17,18,19]: DPPH (1,1-diphenyl-2-picrylhydrazyl) radical stock solution was prepared in EtOH at a concentration of 2.5 M and stored at −18 °C. The stock solutions of synthetic keto-carotenoids or beta-carotene and canthaxanthin as controls were prepared at a concentration of 1.67 M in a 1:2 (*v*:*v*) mixed solvent of CH_2_Cl_2_ and DMSO and stored at –18 °C. An aliquot of 200 μL of the above carotenoids or the blank—CH_2_Cl_2_/DMSO (1:2) was added to 300 μL of DPPH radical in EtOH. The mixture was vortexed for 1 min and left to stand at 25 °C for 2 h in the dark. Because the UV absorption values for DPPH radical (517 nm) and for most ketonic carotenoids (496~550 nm) overlap significantly, separation of the DPPH radical by HPLC was necessary [30]. The above resulting solution was filtered using a 0.45 μm polyether sulfone membrane filter (HYUNDAI Micro Co., LTD., Seoul, Korea) and an aliquot (10 μL) of the sample was injected for HPLC analysis. The reversed-phase HPLC system (Waters Corporation, Milford, MA, USA) consisted of a binary pump, a system controller (Empowers 3 Software), an auto-injector, and a photodiode array detector with an Agilent Poroshell 120 EC-C18 column (4 µm, 4.6 × 150 mm, Carbon Load: 1%). Isocratic elution was carried out with MeOH (0.5 mL/min) and with H_2_O (0.5 mL/min) at a total flow rate of 1 mL/min. The DPPH peaks (~3.4 min retention time) were monitored at 517 nm in trifold analyses. The reduced integration of the DPPH peak area (*PA*) by the carotenoids from that of the blank was calculated as the DPPH radical scavenging activity in the following formulation.

Scavenging Activity = {1 − (*PA*_sample_/*PA*_blank_)}

ABTS (2,2’-azino-bis(3-ethylbenzohiazoline-6-sulfonic acid) cationic radical assay [20]: Each stock solution of 7.4 mM ABTS diazonium salt in H_2_O and 2.6 mM potassium persulfate in H_2_O was prepared. The working solution was prepared by mixing the two stock solutions in the ratio of 1:1 and performing a reaction at 25 °C for 12 h in the dark. The aliquot of 5 mL resulting solution was then diluted with 250 mL MeOH to obtain an absorbance of 0.7 at 734 nm. Fresh ABTS cationic radical solution (600 μL) was added to 2.5 M carotenoid solution in 50 μL of CH_2_Cl_2_/DMSO (1:2 *v*:*v*) and reacted at 25 °C for 2 h in the dark. The resulting mixture (0.3 mL) was placed in a microcuvette in a double beam UV-Vis spectrophotometer (Scinco NEOSYS-2000), in which the absorbance was measured at 734 nm. Particular care was taken to minimize the loss of free radical activity of the ABTS radical stock solution by storing at 4 °C until it was used. The absorbances (*A*) of ABTS cationic radical in the blank and in the carotenoid were calculated and the ABTS cationic radical scavenging activity was obtained using the following formulation.

Scavenging Activity = {1 − (*A*_sample_/*A*_blank_)}

Superoxide radical Assay [27,28,29]: Superoxide radicals are generated in the PMS-NADH-O_2_ (phenazine methosulfate-nicotinamide adenine dinucleotide-oxygen) system, which normally utilize the reduction of nitroblue tetrazolium (NBT) into formazan with 562 nm absorption for superoxide radical scavenging assay. Du to overlapping absorption of formazan with keto-carotenes; however, NBT could not be used in this experiment. Instead, *t*-butylhydroquinone (TBHQ) was utilized for the oxidation to the corresponding benzoquinone by superoxide radicals with 290 nm absorption [31]. The tubes bearing the reaction mixture {100 μL of 1.0 mM of TBHQ in EtOH/H_2_O (1:1) + 200 μL of 468 μM NADH in Phosphate-buffered saline solution (pH = 7.0) + 200 μL of 60 μM PMS + 100 μL of 2 mM ketonic carotene in CH_2_Cl_2_/DMSO (1:2)} were vortexed for 60 s, and then incubated at 25 °C for 1 h in the dark. The above resulting solution was filtered through 0.45 μm polyether sulfone membrane filter (HYUNDAI Micro Co., LTD., Seoul, Korea) and an aliquot (10 μL) of the sample was injected for HPLC analysis. The reversed-phase HPLC system (Waters Corporation) consisted of a binary pump, a system controller (Empowers 3 Software), an auto-injector, and a photodiode array detector with an Agilent Poroshell 120 EC-C18 column (4 µm, 4.6 × 150 mm, Carbon Load: 1%). Isocratic elution was performed with MeOH (0.7 mL/min) and with H_2_O (0.3 mL/min) at a total flow rate of 1 mL/min. The TBHQ peaks (~2.4 min retention time) were monitored at 290 nm in trifold analyses. The capability of O_2_^•−^ scavenging activity was calculated using the following equation.

Inhibition Ratio = (A_x_ − A_1_) / (A_0_ − A_1_)

where A_1_ and A_x_ are the peak areas of the TBHQ probe in the absence and presence of superoxide anion radical scavenger (keto-carotenoid), respectively; and A_0_ is the peak area of the TBHQ probe at initial concentration in the reaction mixture.

## 3. Results and Discussion

### 3.1. Syntheses of Oxo-Carotenoids

**Scheme 1:**
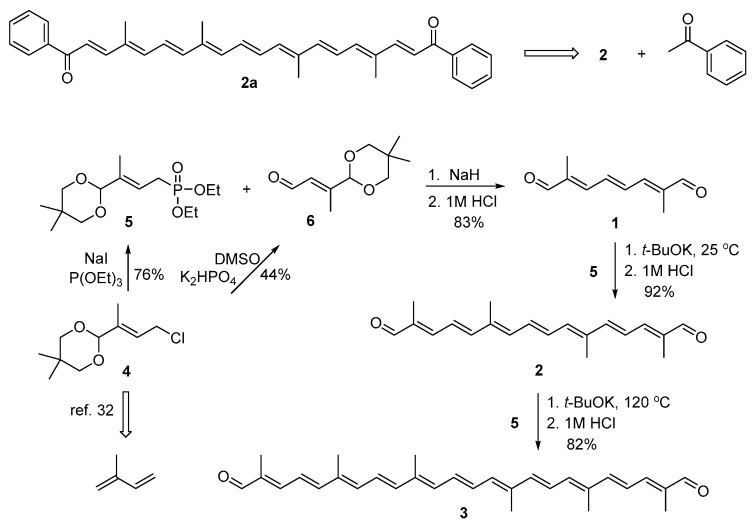
Preparation of ketonic carotene **2a** by aldol condensation of crocetin dial **2** with acetophenone and a chain-extension protocol for polyene dials **2** and **3** based on HWE olefination.

We designed ketonic carotene **2a** as a main model substrate for oxo-carotenoids in superoxide radical scavenging assay, which would be prepared by aldol condensation between acetophenone and C_20_ crocetin dial **2** (Scheme 1) [37]. Sliwka and we independently reported the chain-extension protocol for the synthesis of polyene dials **2** and **3** based on Wittig and Julia–Kocienski olefinations, respectively [32,36]. Even though high yields were reported for the syntheses of **2** and **3** by Sliwka, only milligram quantities were obtained after HPLC separation from the microwave-assisted Wittig reaction [36]. On the other hand, the chain-extension by double Julia-Kocienski olefination produced polyene dials **2** and **3** in sub-gram quantities, but mono-coupling was a major problem for the elongated polyene dial **3** [32]. The yield of C_30_ polyene dial **3** was dropped significantly because of incomplete deprotection of acetal units. It was thus necessary to develop a practical synthetic method of polyene dials **2** and **3** in over-gram quantities for the preparation of various ketonic carotenoids by aldol condensation with acetophenones.

We investigated the synthesis of polyene dials **2** and **3** based on Horner–Wadsworth–Emmons (HWE) olefination in the hope that more nucleophilic phosphonate (e.g., **5**) carbanion would efficiently produce the chain-extended higher homologues and that the byproduct phosphenic acid would turn into phosphoric acid in aqueous acidic medium (1M HCl) to help with the acetal deprotection (Scheme 1). Acetal-protected allylic chloride **4**, prepared from isoprene at an 86% overall yield using the published procedure [32], was converted into C_5_ phosphonate **5** at a 76% yield using the sequence of Finkelstein (NaI) and Arbuzov reactions (triethyl phosphite). On the other hand, DMSO-mediated oxidation of acetal-protected allylic chloride **4** produced the corresponding C_5_ aldehyde **6** in 44% yield [34]. The HWE reaction of C_5_ phosphonate **5** with C_5_ aldehyde **6** under NaH in THF at 25 °C, followed by the direct hydrolysis of the acetal protection in aqueous acidic (1M HCl) solution smoothly produced C_10_ 2,7-dimethyl-2,4,6-octatrienedial (**1**) in 83% yield.

We then tested a chain extension protocol utilizing C_5_ phosphonate **5** for the practical synthesis of polyene dials **2** and **3**. The HWE reaction of C_10_ dial **1** (6.0 g, 1 equiv.) with C_5_ phosphonate **5** (2.2 equiv.) under *t*-BuOK in THF/*t*-BuOH at ambient temperature, followed by 1M HCl addition, produced C_20_ crocetin dial **2** in 92% yield (10.0 g) after purification by trituration from Et_2_O. Further extension to C_30_ polyene dial **3** (590 mg, 1.38 mmol) was performed equally well with an 82% yield by conducting a HWE reaction of C_5_ phosphonate **5** (1.50 g, 4.90 mmol) with C_20_ crocetin dial **2** (500 mg, 1.69 mmol) even though a higher temperature of 120 °C was required for the double olefination. Once again, deprotection of acetal was effectively completed upon the addition of aqueous acidic solution presumably by the effect of the by-product, phosphoric acid.

With the series of polyene dials **1-3** in hand, the optimal conditions for aldol condensations with acetophenone, which seemed easy to achieve but were not, were carefully studied (Table 1). Initial screening of C_10_ dial **1** with acetophenone under aqueous NaOH or NaH in THF conditions was disappointing (entries 1–2). No condensation product was obtained when using K_2_CO_3_ base in MeOH at 25 °C (entry 3). The single condensation product **1a-al** started to form in low yields at 70 °C in MeOH using DBU or NaOMe as a base (entries 4–5), which was optimized in THF at −78 °C using NaHMDS base (35% yield, entry 7). The double aldol condensation product **1a** was first obtained in 20% yield when Triton B was used in MeOH at 70 °C, which was maximized with the NaOH base in MeOH/H_2_O (10:1) at 25 °C to obtain a 72% yield (entries 8–9). Homogeneity of reagents and base in solvent appeared to be most important in the double aldol condensation between C_10_ dial **1** and acetophenone.

The conditions for double aldol condensation between C_20_ crocetin dial **2** and acetophenone were then investigated starting with the above optimal condition using NaOH base in MeOH/H_2_O (10:1) at 25 °C (Table 2). It was surprising that no condensation product was obtained under this condition (entry 1). The homogeneity issue again was decisive. The longer chain of **2** resulted in lower polarity, and removal of water from the reaction medium provided a single condensation product of **2a-al** at a 48% yield when LiOMe was used as a base at 25 °C (entry 2). The highest yield (57%) of **2a-al** was obtained from the NaOH base in MeOH at 70 °C, while low temperature (−78 °C) reactions with the metal amide bases in THF produced much lower yields (9~13%) of **2a-al** (entries 3–5). The reaction using Triton B as a base in MeOH produced no condensation products at 25 °C and no detectible product at 70 °C by decomposition (entries 6 and 7). The double aldol condensation product **2a** was finally obtained in 40% yield under a lower polar medium of THF at 25 °C with Triton B as a base (entry 8) [38]. Double aldol condensation of acetophenone with C_30_ polyene dial **3** was carried out under the above optimum condition of Triton B in THF at 25 °C to give **3a** in 25% yield.

**Figure 2:**
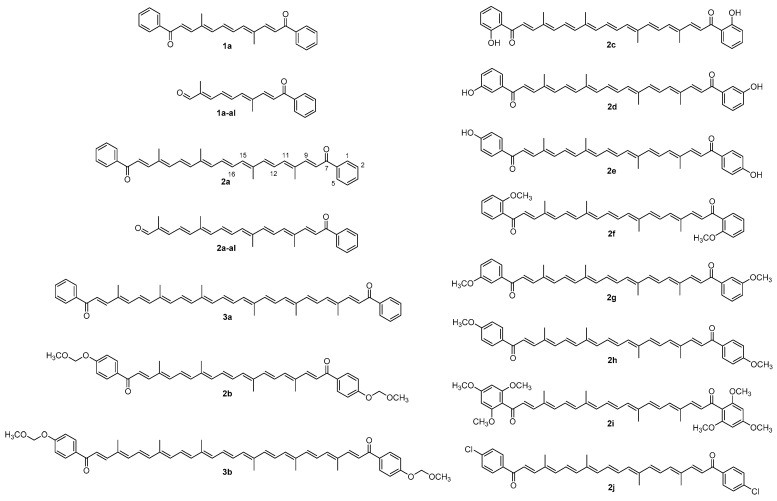
Structures of various novel oxo-carotenoids prepared by the aldol condensation between polyene dials **1-3** and acetophenones with different ring substituents.

Acetophenones with different ring substituents were selected to diversify the oxo-carotenoid end groups. Various novel oxo-carotenoids **2a-2j** and **3a-3b** were prepared by conducting double aldol condensation between polyene dials **2** or **3** and acetophenones with the ring substituents to evaluate the effects of hydroxy and methoxy groups in terminal benzene rings on the antioxidant activities of the ketonic carotenoids (Figure 2) [39]. MOM substitution (**2b** and **3b**) was chosen for solubility reasons and electronegative chlorine substitution (**2j**) for comparison with other electron-rich groups. The specific reaction conditions for double aldol condensation and their yields are summarized in Table 3 (Supplementary Materials provide the experimental procedure and analytical data for **2b-2j** and **3b**), together with the maximum UV absorption wavelengths (λ_max_) for the conjugated polyene system. The Density Functional Theory (DFT) calculation {rb3lyp/6–31 g(d,p) as an optimization function set} was performed to obtain the most stable geometries (Supplementary Materials), as well as the energy gaps (ΔE) between HOMO and LUMO levels and the dihedral angles between the benzene ring and the polyene chain (Table 3).

The double aldol condensation of C_20_ crocetin dial **2** and acetophenones substituted with -OMOM, -OH, -OMe, and -Cl was mostly performed under NaOH base in MeOH at 70 °C to obtain oxo-carotenoids **2b**, **2c**, **2d**, **2f**, **2g**, and **2j**. Moderate to good yields (44~89%) were obtained except for **2j** with *para*-chlorine substituent (16% yield). Oxo-carotenes **2h** (69% yield) and **2i** (39% yield) containing a *para*-methoxy group were successfully prepared by the condition using Triton B in THF at 25 °C. Preparation of oxo-carotenes **2e** with *para*-hydroxy and **3b** with *para*-methoxymethoxy substituent required harsh condition using *t*-BuOK base in toluene at 110 °C, but low yields (18% and 9%, respectively) were obtained.

The wavelength (λ_max_) of maximum UV absorption is related to the energy gap between HOMO and LUMO levels and represents the effective conjugation length of the π-system in oxo-carotenoids. The number (*N*) of conjugated C=C bonds for **3a** and **3b** was 13, whereas it was *N* = 9 for **2a-2j**, and *N* = 5 for **1a**. The UV absorption (λ_max_) for **3a** is 550 nm, for **2a** it is 496 nm, and for **1a** it is 441 nm, which decreases systematically as *N* decreases. Although DFT calculations predicted similar energy gaps between HOMO and LUMO levels within the **2a-2j** series (*N* = 9), a significant red-shift of λ_max_ was evident for ketonic carotenoids **2b-2j** with auxochrome substituents on the benzene ring. Relatively precise values (510~520 nm) were observed except for **2c** (549 nm) and **2i** (497 nm), which can be explained by the effectiveness of conjugation. The dihedral angle between the benzene ring and the polyene chain for **2c** was predicted to be 0° for maximum conjugation due to intramolecular hydrogen bonding between the *ortho*-OH and carbonyl groups of the chain, whereas the 53.5° dihedral angle for **2i** indicates poor conjugation because of the sterically crowded *ortho*-dimethoxy substituents.

### 3.2. Antioxidant Activities of Oxo-Carotenoids

Antioxidant activities for the prepared novel oxo-carotenoids together with apo-carotenedials (**1–3**) and apo-carotenals (**1a-al**, **2a-al**) were then tested by applying the standard DPPH (radical) and ABTS (cationic radical) scavenging assays with β-carotene and canthaxanthin as references for simple and oxo-carotenoid, respectively. Unlike ABTS cationic radical which absorbs UV at 734 nm, the maximum absorption wavelength (517 nm) of DPPH radical overlaps with that of the above oxo-carotenoids (496~550 nm). DPPH radical is stable and the HPLC method for separation and measurement of the pure DPPH radical without interferences from oxo-carotenoids and other sources is reliable [30]. Therefore, the DPPH radical scavenging analysis of oxo-carotenoids and apocarotenoids was performed using the HPLC method.

Superoxide anionic radical (O_2_^−^^·^) can be generated nonenzymatically by the reaction of phenazine methosulfate (PMS) and nicotinamide adenine dinucleotide (NADH) under aerobic conditions, which readily reduces nitroblue tetrazolium (NBT) to formazan [27,28,29]. The antioxidant activity of carotenoids is regarded as the competition with NBT for superoxide radical, which is often measured quantitatively by the reduction of the formazan absorption peak at 560 nm. Unfortunately, the coincidence of the UV absorption peaks with oxo-carotenoids prevented us from utilizing a formazan probe in the superoxide radical assay, and formazan was not even detected when using the HPLC method. Instead, *t*-butylhydroquinone (TBHQ) was chosen as an orthogonal probe in the oxidation to the corresponding benzoquinone by superoxide radicals [31]. The reduction in the TBHQ peak at 290 nm by oxo-carotenoids represents the antioxidant activity in superoxide radical assay. The HPLC method was utilized again to eliminate any potential interferences for the assay in the short wavelength UV region.

**Figure 3:**
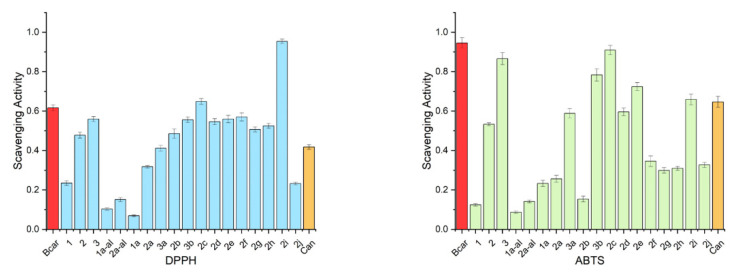

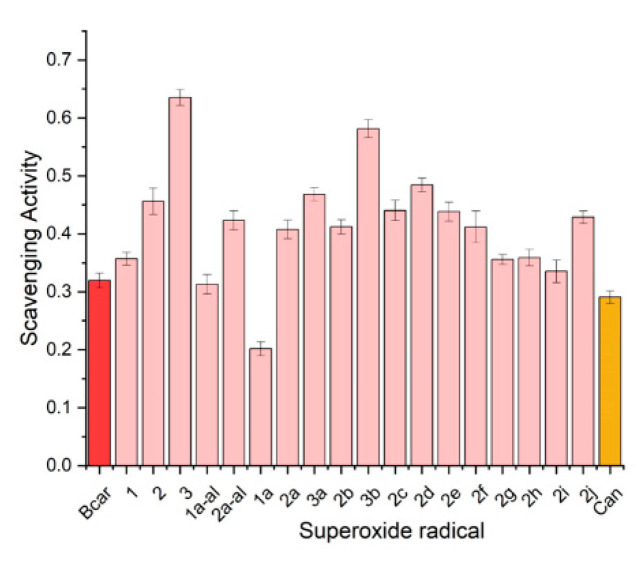
Antioxidant activity of oxo-carotenoids by the scavenging activity for DPPH, ABTS, and Superoxide radicals.

The antioxidant activity of the oxo-carotenoid was represented by the scavenging activity for each probe radical, which was measured as the reduced fraction in the UV absorption peak of each probe by oxo-carotenoids. The scavenging activity values of oxo-carotenoids for DPPH radical (DR), ABTS cationic radical (AR), and superoxide anionic radical (SR) were measured three times and the mean and standard deviation are summarized in Figure 3 (see also Table S1 in Supplementary Materials) together with β-carotene and canthaxanthin as references. It is very interesting to compare the trend of radical scavenging activity of carotenoids in DR/AR (neutral and cationic radical) assays with that of the SR (anionic radical) assay in Figure 3.

The antioxidant activity of β-carotene in DR and AR assays is higher (20~30%) than that of canthaxanthin as was reported [22,23], whereas almost the same activities were observed in the SR assay. β-Carotene is a better antioxidant than oxo-carotenoids in DR and AR assays except for in the case of **2c** (2’-OH) and **2i** (2’,4’,6’-trimethoxy) in DR assay (vide infra), but it is the weakest antioxidant in SR scavenging except the short **1a** (*N* = 5) series. The carbonyl groups in the rings are better in terms of scavenging DR and AR; on the other hand, those in the chain are effective in scavenging SR based on the comparison between **2a** and canthaxanthin.

The antioxidant activities of the DR and AR assays were of a similar trend for the oxo-carotenoids as outlined in the following: (1) apo-carotenedials **1–2** are much better in radical scavenging than the corresponding apo-carotenals (**1a-al** and **2a-al**); (2) the substitution effect in terminal rings for radical quenching follows in the order of ortho > para > meta -OH and -OMe groups (**2c-2h**); (3) electron-rich terminal ring (**2i**) scavenges the radicals better than the electron-deficient one (**2j**). There also exist certain differences in that electron density provided by the substituents in the terminal rings is more important in DR assay (**2i** vs. **2j**), and that phenolic end groups (**2c-2e**) are superior to the corresponding anisole end groups (**2f-2h**) in the AR assay. Effective conjugation by intramolecular hydrogen bonding between *ortho*-phenol and the carbonyl groups in **2c** (0° dihedral angle) is noteworthy for the 90.9% scavenging activity in the AR assay.

The antioxidant activity of oxo-carotenoids with the SR assay is much different to those for the DR and AR assays. In the SR assay, apocarotenals (**1a-al** and **2a-al**) were found to be good antioxidants given the corresponding apo-carotenedials (**1–2**). The Phenol end groups (**2c-2e**) are still better than anisole end groups (**2f-2h**), but there is no systematic positional effect of the substituent OH or OMe. Oxo-carotene **2j** with an electron-deficient *para*-chlorobenzene ring is superior to the electron-rich oxo-carotene **2i**, which is opposite to the results for the DR and AR assays.

**Scheme 2:**
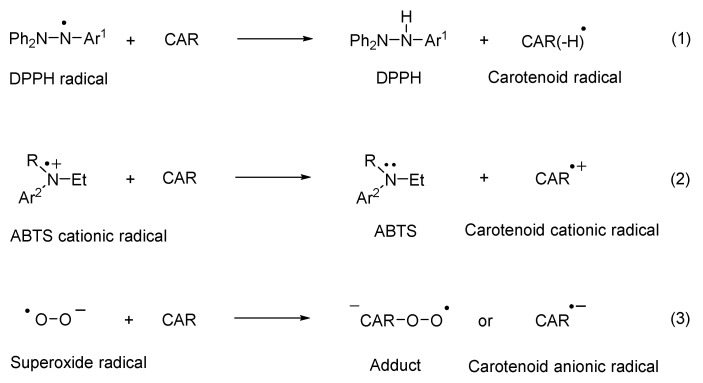
Antioxidant radical quenching mechanism of carotenoids for each radical probe: (**1**) hydrogen radical transfer to DPPH; (**2**) single electron transfer to ABTS; (**3**) adduct formation or single electron transfer to carotenoids from superoxide radical.

All the experimental results on antioxidant activity of the oxo-carotenoids for each radical probe agree well with the mechanism in Scheme 2. Facile hydrogen radical abstraction from many allylic positions of β-carotene makes it a better antioxidant compared with most oxo-carotenoids in the DR assay, whereas high electron-density in the phenyl rings of oxo-carotene **2i** (1,3,5-trimethoxy substitution) reversed the activity by stabilizing the resultant radical species (1). A single electron transfer from the polyene chain of oxo-carotenoids to ABTS cationic radical produces unstable electron-deficient cationic radical activity of keto-carotenes, which positions β-carotene as a better antioxidant in the AR assay (2). On the other hand, the electron-deficient oxo-carotenoids would readily accept an electron [15] or form an adduct [16] from superoxide radical to produce carotenoid anionic radical, which means oxo-carotenoids are better antioxidants than β-carotene (3). Because the size of the frontier molecular orbital coefficient is correlated with the reaction site [40], DFT calculation was performed for **2b** using the Multiwfn program [41] to predict the position of superoxide radical addition (Supplementary Materials). It is the central carbon (C-16) of the polyene chain that produces the highest coefficient of 7.33% in LUMO. It was demonstrated that superoxide anionic radical readily reacts with the conjugated carbonyl chain of oxo-carotenoids to produce the resonance-stabilized carotenoid anionic radical. Therefore, the antioxidant activity of oxo-carotenoids including xanthophylls should be fairly evaluated by anionic (radical) probe, such as the superoxide radical scavenging assay.

## 4. Conclusions

We developed a synthetic method of ketonic carotenoids as a simple model for oxo-carotenoids by performing aldol condensation of polyene dials and acetophenones containing different ring substituents. A practical chain-extension protocol for polyene dials was also established on a multigram scale based on the Horner–Wadsworth––Emmons reaction. The antioxidant activity of oxo-carotenoids was measured by performing a standard radical scavenging assay utilizing DPPH and ABTS radicals and we compared the results with those of the superoxide anionic radical assay. Although β-carotene was demonstrated to be a very potent antioxidant by DPPH and ABTS assays, the oxo-carotenoids exhibit superior antioxidant activity than the natural carotenoid in the superoxide radical scavenging assay. Each assay method suggests different antioxidant activity for the carotenoids, and the selection of the right probe for the ROS species of interest is important to obtain an impartial evaluation of carotenoids. It is the conjugated polyene chains that provide antioxidant activity to carotenoids. Increasing the electron density and increasing the effective conjugation length would be beneficial for superior antioxidant activity in DPPH and ABTS assays. The latter is also true for the superoxide radical scavenging assay but reducing the electron density in this case would favor superior antioxidant activity. Oxo-carotenoids are powerful antioxidants that were demonstrated to be valuable using the scavenging assay for superoxide radical, which is a precursor of most ROS.

## Figures and Tables

**Table 1:** Optimization study for aldol condensation between C_10_ dial **1** and acetophenone—Product yields of **1a-al** (mono-coupling) and **1a** (di-coupling).

Entry ^a^	Base	Solvent	Condition (°C, h)	1a-al (%)	1a (%)
1	NaOH	H_2_O	25, 12	0	0
2 ^b^	NaH	THF	70, 12	-	-
3	K_2_CO_3_	MeOH	25, 12	0	0
4	DBU	MeOH	70, 12	12	0
5	NaOMe	MeOH	70, 3	20	0
6	LDA	THF	−78, 2	21	0
7	NaHMDS	THF	−78 to 25, 2	35	0
8	Triton B	MeOH	70, 12	0	20
9	NaOH	*aq*. MeOH	25, 12	0	72

^a^ Acetophenone (3 equiv.) and C_10_ dial **1** (1 equiv.) were reacted with each base (5 equiv.) under the specified condition in each solvent. ^b^ no detectible product was obtained (decomposed).

**Table 2:** Optimization Study for Aldol Condensation between C_20_ dial **2** and acetophenone—Product yields of **2a-al** (mono-coupling) and **2a** (di-coupling).

Entry ^a^	Base	Solvent	Condition (°C, h)	2a-al (%)	2a (%)
1	NaOH	*aq*. MeOH	25, 12	0	0
2	LiOMe	MeOH	25, 12	48	0
3	NaOH	MeOH	70, 12	57	0
4	LDA	THF	−78, 2	9	0
5	NaHMDS	THF	−78, 2	13	0
6	Triton B	MeOH	25, 12	0	0
7 ^b^	Triton B	MeOH	70, 5	-	-
8	Triton B	THF	25, 12	0	40

^a^ Acetophenone (3 equiv.) and C_20_ dial **2** (1 equiv.) were reacted with each base (5 equiv.) under the specified condition in each solvent. ^b^ no detectible product was obtained (decomposed).

**Table 3:** The reaction condition and the yield for oxo-carotenoids in Figure 2, their UV maximum absorption wavelength, energy gap between HOMO and LUMO levels, and dihedral angle between benzene ring and polyene chain by DFT {rb3lyp/6–31 g(d,p)} calculation.

Compd.	Condition ^a^	Yield (%)	UV (nm)	ΔE ^b^ (kcal/mol)	Angle ^c^ (°)
**1a**	A	72	441	62.89	12.3
**2a**	B	40	496	49.21	13.0
**3a**	B	25	550	42.96	13.1
**2b**	C	44	520	49.50	6.6
**3b**	D	18	551	43.18	7.8
**2c**	C	46	549	48.39	0.0
**2d**	C	87	518	49.18	13.4
**2e**	D	9 ^d^	516	49.48	5.5
**2f**	C	89	516	49.27	28.8
**2g**	C	59	510	49.28	13.0
**2h**	B	69	515	49.51	5.6
**2i**	B	39	497	49.99	53.5
**2j**	C	16	518	48.82	11.3

^a^ A = NaOH was used as a base in MeOH/H_2_O (10:1) at 25 °C; B = Triton B was used as a base in THF at 25 °C; C = NaOH was used as a base in MeOH at 70 °C; D = *t*-BuOK was used as a base in toluene at 110 °C. ^b^ Energy gap between HOMO and LUMO levels. ^c^ Dihedral angle between benzene ring and polyene chain. ^d^ **2e** was not purified by SiO_2_ column but recrystallized from cold MeOH.

## Data Availability

All data are included within the article and supplementary materials.

## Supplementary Materials

The following supporting information can be downloaded at https://www.mdpi.com/article/10.3390/antiox11081525/s1, Experimental Procedure for **2b**–**2j** and **3b**, Cartesian coordinates for the optimized geometry by DFT calculation, LUMO coefficient of **2b**, Table S1: Antioxidant activity by DPPH, ABTS, and superoxide radical scavenging assays, ^1^H- and ^13^C-NMR spectra, Assignment of *E*-stereochemistry for **4** by N.O.E., and Peak assignment for **2b** by COSY, HMBC, and DEPT.
