# Supplementary material for: Oxo-Carotenoids as Efficient Superoxide Radical Scavengers
Source: Antioxidants (Basel). 2022 Aug 5;11(8):1525. doi: 10.3390/antiox11081525 (PMC9405038; doi:10.3390/antiox11081525)
Supplement: Supplementary file 1 [file antioxidants-11-01525-s001.zip › Supplementary Materials-1.pdf]

Supplementary Materials-1 for

Oxo-carotenoids as Efficient Superoxide Radical Scavengers

Gaosheng Shi,[a] Hyein Kim[b] and Sangho Koo[a,b]*

[a] Department of Energy Science and Technology, [b] Department of Chemistry, Myongji University, Myongji-Ro 116, Yongin, 17058, Gyeonggi-Do, Korea

Table of Contents

1. Experimental Procedure for **2b**–**2j** and **3b** ------------------------------ S-2

2. Cartesian coordinates for the optimized geometry by DFT calculation ----- S-9

3. LUMO coefficient of **2b** -- S-35

4. Table S1: Antioxidant activity by DPPH, ABTS, and superoxide radical scavenging assays --- S-36

1. Experimental Procedure for 2b-2j and 3b.

All-(*E*)-1,20-bis(4-(methoxymethoxy)phenyl)-4,8,13,17-tetramethylicosa-2,4,6,8,10,12,14,16,18-nonaene-1,20-dione (**2b**).

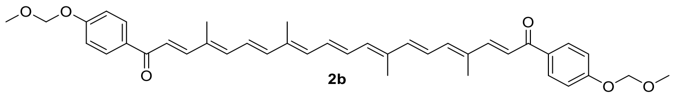
2b

To a stirred solution of C_{20} dial **2** (0.30 g, 1.01 mmol) and 4′-methoxymethylacetophenone (0.54 g, 3.03 mmol) in MeOH (30 mL) was added NaOH (0.90 g, 20.2 mmol). The mixture was heated at 70 °C for 12 h and cooled to room temperature. The mixture was quenched with 1 M HCl solution, extracted with EtOAc, dried over anhydrous Na_2SO_4, filtered, and concentrated under reduced pressure. The crude product was recrystallized from MeOH to give **2b** (0.28 g, 0.45 mmol) in 44% yield as red solid. Data for **2b**: R_f = 0.28 (3:1 hexane/acetone); ^{1}H NMR ($CDCl_3$) δ = 2.01 (s, 6H), 2.06 (s, 6H), 3.50 (s, 6H), 5.25 (s, 4H), 6.33–6.43 (m, 2H), 6.55 (d, *J* = 14.4 Hz, 2H), 6.62 (d, *J* = 11.2 Hz, 2H), 6.69 (dd, *J* = 14.4, 11.2 Hz, 2H), 6.66–6.76 (m, 2H), 6.97 (d, *J* = 15.2 Hz, 2H), 7.10 (d, *J* = 8.8 Hz, 4H), 7.54 (d, *J* = 15.2 Hz, 2H), 7.97 (d, *J* = 8.8 Hz, 4H) ppm; ^{13}C NMR ($CDCl_3$) δ = 12.8, 12.9, 56.2, 94.1, 115.7, 120.2, 124.7, 130.5, 131.3, 132.5, 134.3, 135.1, 137.0, 140.8, 142.0, 148.6, 160.7, 188.9 ppm; UV (2:1 DMSO/CH_2Cl_2, c = 0.26 mmol/L): λ (ε) = 520 nm (385,000); IR (KBr) ν = 2924, 2851, 1734, 1672, 1644, 1600, 1546, 1508, 1441, 1418, 1358, 1302, 1261, 1242, 1218, 1200, 1170, 1153, 1081, 986, 922, 828, 749, 656 cm^{-1}; HRMS (EI) calcd for $C_{40}H_{44}O_6$ 620.3138, found 620.3135.

All-(*E*)-1,20-bis(2-hydroxyphenyl)-4,8,13,17-tetramethylicosa-2,4,6,8,10,12,14,16,18-nonaene-1,20-dione (**2c**).

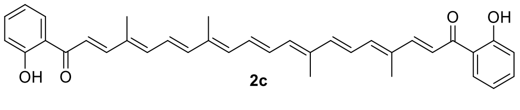
2c

Following the general procedure for **2b**, the reaction of C_{20} dial **2** (0.10 g, 0.34 mmol) and 2′-

hydroxyacetophenone (0.14 g, 1.03 mmol) with NaOH (0.27 g, 6.74 mmol) in MeOH (10 mL) at 70 °C for 12 h provided **2c** (83 mg, 0.16 mmol) in 46% yield as red solid. The all-(*E*) product was obtained by recrystallization from Et_2O and MeOH. Data for **2c**: R_f = 0.42 (3:1 hexane/acetone); ^{1}H NMR δ = 2.02 (s, 6H), 2.08 (s, 6H), 6.35–6.48 (m, 2H), 6.59 (d, *J* = 14.4 Hz, 2H), 6.68 (d, *J* = 11.6 Hz, 2H), 6.68–6.80 (m, 4H), 6.91 (dd, *J* = 8.4, 8.0 Hz, 2H), 7.00 (d, *J* = 8.4 Hz, 2H), 7.09 (d, *J* = 15.2 Hz, 2H), 7.46 (dd, *J* = 8.4, 8.0 Hz, 2H), 7.66 (d, *J* = 15.2 Hz, 2H), 7.86 (d, *J* = 8.4 Hz, 2H), 13.01 (s, 2H) ppm; ^{13}C NMR δ = ppm; UV (2:1 DMSO/CH_2Cl_2, c = 0.26 mmol/L): λ_{max} (ε) = 549 nm (432,692); IR (KBr) ν = 3387, 2923, 1713, 1629, 1578, 1538, 1486, 1440, 1362, 1340, 1304, 1275, 1256, 1199, 1157, 1026, 966. 751, 688 cm^{-1}; HRMS (ESI) calcd for $C_{36}H_{36}O_4$+Na 555.2506, found 555.2507.

All-(*E*)-1,20-bis(3-hydroxyphenyl)-4,8,13,17-tetramethylicosa-2,4,6,8,10,12,14,16,18-nonaene-1,20-dione (**2d**).

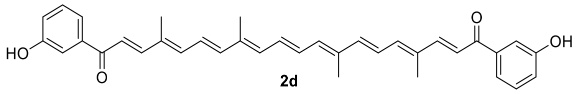

Following the general procedure for **2b**, the reaction of C_{20} dial **2** (0.10 g, 0.34 mmol) and 3′-hydroxyacetophenone (0.23 g, 1.69 mmol) with NaOH (0.13 g, 15 mmol) in MeOH (10 mL) at 70 °C for 12 h produced **2d** (0.157 g, 0.295 mmol) in 87% yield as red solid, which was all-(*E*) product. Data for **2d**: R_f = 0.23 (1:1 hexane/acetone); ^{1}H NMR (DMSO-d_6) δ = 1.98 (s, 6H), 2.05 (s, 6H), 6.43–6.54 (m, 2H), 6.65 (d, *J* = 14.8 Hz, 2H), 6.72–6.81 (m, 4H), 6.79–6.88 (m, 2H), 7.01 (ddd, *J* = 8.0, 2.4, 1.2 Hz, 2H), 7.08 (d, *J* = 15.2 Hz, 2H), 7.32 (t, *J* = 8.0 Hz, 2H), 7.35 (dd, *J* = 2.4, 1.2 Hz, 2H), 7.42 (d, *J* = 15.2 Hz, 2H), 7.48 (dt, J_d = 8.0, J_t = 1.2 Hz, 2H), 9.77 (s, 2H) ppm; ^{13}C NMR (DMSO-d_6) δ = 13.0, 13.1, 114.8, 119.6, 120.3, 120.9, 125.7, 130.2, 132.2, 135.0, 135.7, 137.6, 139.9, 141.6, 142.6, 148.7, 158.1, 189.2 ppm; UV (2:1 DMSO/CH_2Cl_2, c = 0.26 mmol/L):

λ_{max} (ε) = 518 nm (253,076); IR (KBr) ν = 3378, 3235, 1639, 1579, 1537, 1449, 1288, 1217, 1181, 998, 968, 879, 831, 784, 760, 671, 651 cm^{-1}; HRMS (ESI) calcd for $C_{36}H_{36}O_4$+Na 555.2506, found 555.2513.

All-(*E*)-1,20-bis(4-hydroxyphenyl)-4,8,13,17-tetramethylicosa-2,4,6,8,10,12,14,16,18-nonaene-1,20-dione (**2e**).

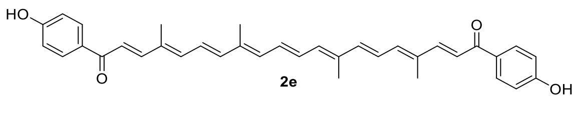

To a stirred solution of C_{20} dial **2** (0.10 g, 0.34 mmol) and 4′-hydroxyacetophenone (0.23 g, 1.69 mmol) in *t*-BuOH/toluene (5 mL/15 mL) was added *t*-BuOK (0.38 g, 3.40 mmol). The mixture was heated at 110 °C for 12 h and cooled to room temperature. The mixture was quenched with 1 M HCl solution, extracted with EtOAc (20 mL). The organic layer was placed in a –5 °C refrigerator overnight to give a precipitate, which was filtered to give **2e** (15 mg, 0.03 mmol) in 9% yield as red solid. Data for **2e**: R_f = 0.15 (3:1 hexane/acetone); ^{1}H NMR δ = 1.98 (s, 6H), 2.05 (s, 6H), 6.41–6.54 (m, 2H), 6.63 (d, *J* = 14.4 Hz, 2H), 6.72–6.86 (m, 6H), 6.85 (d, *J* = 8.4 Hz, 4H), 7.16 (d, *J* = 14.8 Hz, 2H), 7.39 (d, *J* = 14.8 Hz, 2H), 7.95 (d, *J* = 8.4 Hz, 4H), 10.37 (br s, 2H) ppm; UV (2:1 DMSO/CH_2Cl_2, c = 0.26 mmol/L): λ_{max} (ε) = 516 nm (317,692); IR (KBr) ν = 2923, 2854, 1713, 1668, 1653, 1603, 1552, 1506, 1464, 1362, 1221, 1154, 990, 634 cm^{-1}; HRMS (ESI) calcd for $C_{36}H_{36}O_4$+Na 555.2506, found 555.2509.

All-(*E*)-1,20-bis(2-methoxyphenyl)-4,8,13,17-tetramethylicosa-2,4,6,8,10,12,14,16,18-nonaene-1,20-dione (**2f**).

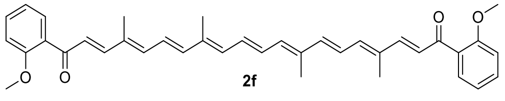

Following the general procedure for **2b**, the reaction of C_{20} dial **2** (0.10 g, 0.34 mmol) and 2′-methoxyacetophenone (0.15 g, 1.01 mmol) with NaOH (0.13 g, 3.25 mmol) in MeOH (10 mL) at

70 °C for 12 h gave all-(*E*)-**2f** (0.168 g, 0.30 mmol) in 89% yield as red solid. Data for **2f**: R_f = 0.37 (3:1 hexane/acetone); ^{1}H NMR ($CDCl_3$) δ = 1.99 (s, 6H), 2.01 (s, 6H), 3.88 (s, 6H), 6.30–6.41 (m, 2H), 6.51 (d, *J* = 14.4 Hz, 2H), 6.55 (d, *J* = 11.6 Hz, 2H), 6.64–6.75 (m, 2H), 6.60 (dd, *J* = 14.4, 11.6 Hz, 2H), 6.80 (d, *J* = 15.2 Hz, 2H), 6.98 (dd, *J* = 8.4, 0.8 Hz, 2H), 7.02 (dt, J_d = 0.8, J_t = 7.6 Hz, 2H), 7.32 (d, *J* = 15.2 Hz, 2H), 7.44 (dt, J_d = 2.0, J_t = 8.4 Hz, 2H), 7.55 (dd, *J* = 7.6, 2.0 Hz, 2H) ppm; ^{13}C NMR (CDCl3) δ = 12.8, 12.8, 55.7, 111.6, 120.6, 124.8, 125.8, 129.8, 130.1, 131.2, 132.3, 134.5, 135.0, 137.0, 140.5, 141.8, 148.1, 157.8, 193.2 ppm; UV (2:1 DMSO/CH_2Cl_2, c = 0.26 mmol/L): λ_{max} (ε) = 516 nm (368,846); IR (KBr) ν = 1642, 1597, 1583, 1541, 1484, 1326, 1312, 1287, 1242, 1215, 1194, 1059, 1020. 978, 775, 760, 649 cm^{-1}; HRMS (ESI) calcd for $C_{38}H_{40}O_4$+Na 583.2819, found 583.2821.

All-(*E*)-1,20-bis(3-methoxyphenyl)-4,8,13,17-tetramethylicosa-2,4,6,8,10,12,14,16,18-nonaene-1,20-dione (**2g**).

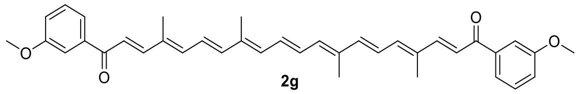
2g

Following the general procedure for **2b**, the reaction of C_{20} dial **2** (0.10 g, 0.34 mmol) and 3′-methoxyacetophenone (0.152 g, 1.01 mmol) with NaOH (0.135 g, 3.4 mmol) in MeOH (10 mL) at 70 °C for 12 h provided all-(*E*)-**2g** (0.113 g, 0.201 mmol) in 59% yield as red solid. Data for **2g**: R_f = 0.35 (3:1 hexane/acetone); ^{1}H NMR ($CDCl_3$) δ = 2.01 (s, 6H), 2.06 (s, 6H), 3.88 (s, 6H), 6.33–6.43 (m, 2H), 6.56 (d, *J* = 14.0 Hz, 2H), 6.63 (d, *J* = 11.2 Hz, 2H), 6.69 (dd, *J* = 14.0, 11.2 Hz, 2H), 6.67–6.77 (m, 2H), 6.95 (d, *J* = 15.2 Hz, 2H), 7.10 (ddd, *J* = 8.0, 2.8, 0.8 Hz, 2H), 7.38 (dd, *J* = 8.0, 7.6 Hz, 2H), 7.50 (dd, *J* = 4.0, 2.4 Hz, 2H), 7.55 (d, *J* = 15.2 Hz, 2H), 7.55 (ddd, *J* = 8.0, 1.8, 0.8 Hz, 2H) ppm; ^{13}C NMR ($CDCl_3$) δ = 12.8, 12.9, 55.5, 112.8, 118.8, 120.4, 120.9, 124.7, 129.4, 131.0, 134.3, 135.2, 137.1, 140.1, 141.2, 142.2, 149.3, 159.8, 190.2 ppm; UV (2:1 DMSO/CH_2Cl_2, c = 0.26 mmol/L): λ_{max} (ε) = 510 nm (230,000); IR (KBr) ν = 2938, 2835, 1676,

1595, 1581, 1542, 1486, 1463, 1429, 1261, 1195, 1171, 1034, 971, 874, 756, 683 cm^{-1}; HRMS (ESI) calcd for $C_{38}H_{40}O_4$+Na 583.2819, found 583.2824.

All-(*E*)-1,20-bis(4-methoxyphenyl)-4,8,13,17-tetramethylicosa-2,4,6,8,10,12,14,16,18-nonaene-1,20-dione (**2h**).

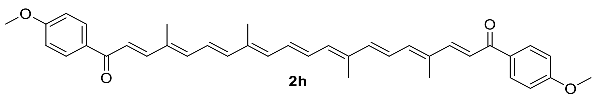

Following the general procedure for **2a**, the reaction of C_{20} dial **2** (0.10 g, 0.34 mmol) and 4′-methoxyacetophenone (0.28 g, 1.69 mmol) with 40% methanol solution of triton B (1.60 g, 2.36 mmol) in THF (10 mL) at 25 °C for 12 h produced **2h** (0.13 g, 0.23 mmol) in 69% yield as red solid. Data for **2h**: R_f = 0.27 (3:1 hexane/acetone); ^{1}H NMR ($CDCl_3$) δ = 2.01 (s, 6H), 2.06 (s, 6H), 3.88 (s, 6H), 6.33–6.43 (m, 2H), 6.55 (d, *J* = 14.4 Hz, 2H), 6.62 (d, *J* = 11.2 Hz, 2H), 6.64–6.75 (m, 4H), 6.96 (d, *J* = 8.8 Hz, 4H), 6.99 (d, *J* = 15.2 Hz, 2H), 7.54 (d, *J* = 15.2 Hz, 2H), 7.99 (d, *J* = 8.8 Hz, 4H) ppm; UV (2:1 DMSO/CH_2Cl_2, c = 0.26 mmol/L): λ (ε) = 515 nm (75,384); IR (KBr) ν = 2924, 2852, 1735, 1641, 1601, 1546, 1510, 1457, 1418, 1397, 1332, 1304, 1256, 1219, 1177, 1119, 1020, 978, 958, 817 cm^{-1}; HRMS (ESI) calcd for $C_{38}H_{40}O_4$+Na 583.2819, found 583.2823.

All-(*E*)-4,8,13,17-tetramethyl-1,20-bis(2,4,6-trimethoxyphenyl)icosa-2,4,6,8,10,12,14,16,18-nonaene-1,20-dione (**2i**).

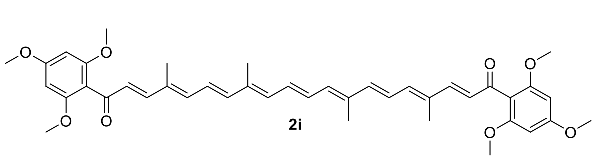

Following the general procedure for **2a**, the reaction of C_{20} dial **2** (0.10 g, 0.34 mmol) and 2′,4′,6′-trimethoxyacetophenone (0.21 g, 1.01 mmol) with 40% methanol solution of triton B (0.71 g, 1.69 mmol) in THF (10 mL) at 25 °C for 12 h gave **2i** (0.083 g, 0.16 mmol) in 39% yield as red solid. The all-(*E*) product was obtained by recrystallization from Et_2O and MeOH. Data for **2i**: R_f = 0.26 (2:1 hexane/acetone); ^{1}H NMR

($CDCl_3$) δ = 1.97 (s, 12H), 3.76 (s, 12H), 3.85 (s, 6H), 6.15 (s, 4H), 6.26–6.36 (m, 2H), 6.43 (d, *J* = 15.6 Hz, 2H), 6.44 (d, *J* = 11.6 Hz, 2H), 6.47 (d, *J* = 14.4 Hz, 2H), 6.63 (dd, *J* = 14.4, 11.6 Hz, 2H), 6.64–6.74 (m, 2H), 7.05 (d, *J* = 15.6 Hz, 2H) ppm; ^{13}C NMR ($CDCl_3$) δ = 12.8, 12.8, 55.4, 55.9, 90.7, 112.1, 124.7, 127.8, 131.1, 134.6, 134.8, 137.0, 139.9, 141.6, 149.0, 158.6, 162.1, 194.5 ppm; UV (2:1 DMSO/CH_2Cl_2, c = 0.26 mmol/L): λ (ε) = 497 nm (183,846); IR (KBr) ν = 3003, 2937, 2840, 1643, 1602, 1587, 1549, 1494, 1455, 1437, 1412, 1368, 1337, 1261, 1225, 1204, 1155, 1126, 1085, 1022, 973, 921, 812, 753, 666, 645 cm^{-1}; HRMS (FAB) calcd for $C_{42}H_{49}O_8$ 681.3427, found 681.3431.

All-(*E*)-1,20-bis(4-chlorophenyl)-4,8,13,17-tetramethylicosa-2,4,6,8,10,12,14,16,18-nonaene-1,20-dione (**2j**).

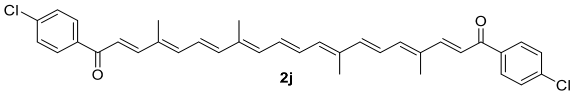

Following the general procedure for **2b**, the reaction of C_{20} dial **2** (0.10 g, 0.34 mmol) and 4′-chloroacetophenone (0.16 g, 1.01 mmol) with NaOH (0.27 g, 6.75 mmol) in MeOH (10 mL) at 70 °C for 12 h provided all-(*E*)-**2j** (0.032 g, 0.056 mmol) in 16% yield as red solid after recrystallization from MeOH. Data for **2j**: R_f = 0.32 (3:1 hexane/acetone); ^1H NMR ($CDCl_3$) δ = 2.01 (s, 6H), 2.06 (s, 6H), 6.34–6.44 (m, 2H), 6.55 (d, *J* = 14.0 Hz, 2H), 6.64 (d, *J* = 11.6 Hz, 2H), 6.61–6.76 (m, 4H), 6.92 (d, *J* = 15.6 Hz, 2H), 7.45 (d, *J* = 8.0 Hz, 4H), 7.55 (d, *J* = 15.6 Hz, 2H), 7.91 (d, *J* = 8.0 Hz, 4H) ppm; UV (2:1 DMSO/CH_2Cl_2, c = 0.26 mmol/L): λ (ε) = 518 nm (180,384); IR (KBr) ν = 2920, 2853, 1734, 1649, 1590, 1551, 1488, 1463, 1398, 1367, 1332, 1299, 1285, 1261, 1218, 1097, 1033. 1011, 976, 955, 823, 760, 735, 676 cm^{-1}; HRMS (EI) calcd for $C_{36}H_{34}Cl_2O_2$ 568.1936, found 568.1835.

All-(E)-1,28-bis(4-(methoxymethoxy)phenyl)-4,8,12,17,21,25-hexamethyloctacosa-2,4,6,8,10,12,14,16,18,20,22,24,26-tridecaene-1,28-dione (**3b**).

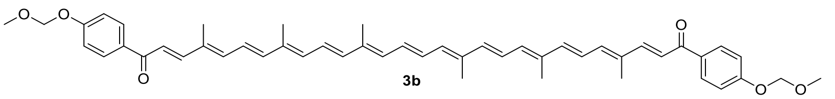

To a stirred solution of C_{30} dial **3** (85 mg, 0.198 mmol) and 4′-methoxymethylacetophenone (0.178 g, 0.991 mmol) in toluene (15 mL) was added *t*-BuOK (0.22 g, 0.198 mmol). The mixture was stirred at 110 °C for 12 h and cooled to room temperature. The mixture was diluted with EtOAc, washed with H_2O, dried over anhydrous Na_2SO_4, filtered, and concentrated under reduced pressure. The crude product was recrystallized from methanol to give all-(E)-**3b** (0.026 g, 0.035 mmol) in 18% yield as dark red solid. Data for **3b**: R_f = 0.5 (2:1 hexane/acetone); ^1H NMR ($CDCl_3$) δ = 2.01 (s, 6H), 2.06 (s, 6H), 3.49 (s, 6H), 5.25 (s, 4H), 6.30–6.40 (m, 2H), 6.36 (d, J = 11.6 Hz, 2H), 6.46 (d, J = 14.8 Hz, 2H), 6.57 (dd, J = 14.8, 11.6 Hz, 2H), 6.61–6.71 (m, 8H), 6.96 (d, J = 15.6 Hz, 2H), 7.10 (d, J = 8.8 Hz, 4H), 7.54 (d, J = 15.6 Hz, 2H), 7.97 (d, J = 8.8 Hz, 4H) ppm; UV (2:1 DMSO/CH_2Cl_2, c = 0.521 mmol/L): λ (ε) = 551 nm (137,044); IR (KBr) ν = 2921, 1646, 1604, 1591, 1578, 1527, 1509, 1443, 1407, 1364, 1330, 1306, 1252, 1209, 1171, 1154, 1105, 1079, 1038, 994, 975, 962, 926, 881, 863, 833, 820, 742, 661 cm^{-1}; HRMS (FAB) calcd for $C_{50}H_{56}O_6$ 752.4077, found 752.4076.

2. Cartesian coordinates for the optimized geometry by DFT calculation

{B3LYP/6–311G(d,p) function set}

Compound **1a**

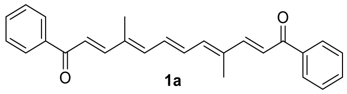

1a

Symbol	X	Y	Z
C	0.566091	0.381132	0.044707
C	-0.566074	-0.381185	0.044766
C	1.885656	-0.175317	0.044344
C	-1.885639	0.175265	0.044344
H	-0.453057	-1.462917	0.045110
H	0.453074	1.462864	0.044905
C	3.081396	0.493411	0.044766
H	1.934660	-1.263826	0.043779
C	-3.081379	-0.493462	0.044872
H	-1.934641	1.263773	0.043630
C	3.203095	1.996467	0.046715
C	4.291015	-0.301751	0.044153
C	-3.203079	-1.996518	0.047029
C	-4.290998	0.301699	0.044164
H	3.753804	2.338547	0.930614
H	2.236338	2.500064	0.042503
H	3.762160	2.340466	-0.831176
C	5.564152	0.159902	0.039536
H	4.172531	-1.383994	0.048203
H	-3.762149	-2.340638	-0.830811
H	-2.236323	-2.500116	0.042882
H	-3.753784	-2.338474	0.930979
C	-5.564136	-0.159953	0.039617
H	-4.172515	1.383943	0.048067
C	6.698953	-0.791172	0.056316
H	5.766998	1.224179	0.046321
C	-6.698932	0.791133	0.056273
H	-5.766977	-1.224228	0.046545
C	8.102937	-0.260338	-0.016782
O	6.508625	-2.004469	0.143143
C	-8.102940	0.260341	-0.016834
O	-6.508582	2.004429	0.143042
C	8.418304	1.066282	-0.348810

C	9.149419	-1.159417	0.245739
C	-8.418390	-1.066200	-0.349115
C	-9.149369	1.159402	0.245956
C	9.747806	1.483477	-0.411078
H	7.634727	1.778957	-0.581867
C	10.474821	-0.741388	0.192142
H	8.889721	-2.183777	0.489420
C	-9.747913	-1.483333	-0.411351
H	-7.634869	-1.778861	-0.582385
C	-10.474792	0.741436	0.192378
H	-8.889613	2.183701	0.489832
C	10.777788	0.583247	-0.136201
H	9.978668	2.511086	-0.675901
H	11.274149	-1.445515	0.403973
C	-10.777837	-0.583119	-0.136216
H	-9.978839	-2.510880	-0.676362
H	-11.274075	1.445551	0.404419
H	11.812426	0.911017	-0.180352
H	-11.812491	-0.910842	-0.180340

Compound **2a**

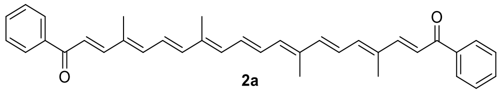

Symbol	X	Y	Z
C	10.441666	0.201197	-0.027022
H	10.760966	1.236571	-0.033536
C	9.123849	-0.116559	-0.030413
H	8.887594	-1.179428	-0.035184
C	8.009614	0.804498	-0.029051
C	6.746757	0.271320	-0.027984
H	6.678374	-0.816370	-0.027901
C	5.493720	0.964900	-0.027578
H	5.503407	2.050605	-0.027829
C	4.294168	0.312970	-0.027234
H	4.312781	-0.776850	-0.027233
C	2.986518	0.919467	-0.027037
C	1.890218	0.090775	-0.026803
H	2.089448	-0.980959	-0.026798
C	0.510240	0.455985	-0.026689
H	0.246905	1.511420	-0.026681
C	-0.510237	-0.455982	-0.026683

H	-0.246903	-1.511417	-0.026712
C	-1.890214	-0.090769	-0.026732
H	-2.089443	0.980966	-0.026698
C	-2.986513	-0.919459	-0.026934
C	-4.294165	-0.312963	-0.027036
H	-4.312779	0.776858	-0.027046
C	-5.493718	-0.964893	-0.027270
H	-5.503405	-2.050598	-0.027494
C	-6.746754	-0.271312	-0.027608
H	-6.678370	0.816378	-0.027534
C	-8.009610	-0.804490	-0.028633
C	-9.123846	0.116566	-0.030030
H	-8.887591	1.179435	-0.034755
C	-10.441664	-0.201109	-0.026760
H	-10.760957	-1.236566	-0.033346
C	-11.463101	0.867144	-0.046914
C	8.294642	2.285737	-0.030090
H	8.877568	2.568074	-0.914636
H	7.387676	2.890840	-0.023560
H	8.889215	2.567229	0.846945
C	2.887994	2.425630	-0.027194
H	1.857442	2.780298	-0.027065
H	3.384933	2.847982	0.853847
H	3.384613	2.847739	-0.908527
C	-2.887984	-2.425621	-0.027075
H	-3.385160	-2.847780	-0.908067
H	-1.857431	-2.780286	-0.027595
H	-3.384367	-2.847926	0.854306
C	-8.294633	-2.285730	-0.029671
H	-8.877022	-2.568183	-0.914538
H	-7.387668	-2.890828	-0.022502
H	-8.889738	-2.567110	0.847036
C	11.463107	-0.867136	-0.047147
O	-11.144507	2.053840	-0.135240
C	-12.917913	0.492454	0.023887
C	-13.859475	1.495853	-0.256251
C	-13.377207	-0.787310	0.371096
C	-15.222840	1.224824	-0.205774
H	-13.488672	2.482738	-0.510739
C	-14.744457	-1.057097	0.430605
H	-12.676021	-1.576802	0.618781
C	-15.669559	-0.054473	0.137586
H	-15.939954	2.008706	-0.431633
H	-15.086702	-2.049917	0.707635
H	-16.733894	-0.267506	0.179513
O	11.144525	-2.053824	-0.135617

C	12.917910	-0.492459	0.023925
C	13.859509	-1.495780	-0.256357
C	13.377154	0.787212	0.371550
C	15.222867	-1.224758	-0.205638
H	13.488744	-2.482600	-0.511155
C	14.744395	1.056986	0.431311
H	12.675929	1.576624	0.619378
C	15.669537	0.054446	0.138129
H	15.940013	-2.008574	-0.431621
H	15.086603	2.049728	0.708666
H	16.733866	0.267471	0.180250

Compound **3a**

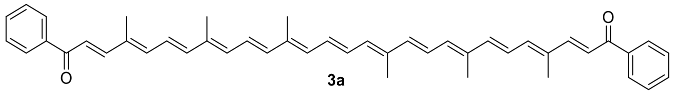

Symbol	X	Y	Z
C	16.033794	-1.173175	-0.050754
C	15.164659	0.020993	-0.025151
H	15.619265	1.004525	-0.028761
C	13.815655	-0.117538	-0.027429
H	13.439690	-1.139396	-0.035377
C	12.834589	0.943278	-0.021345
C	11.511508	0.583645	-0.020174
H	11.299220	-0.485397	-0.023258
C	10.361496	1.436755	-0.016219
H	10.514370	2.511712	-0.013783
C	9.086229	0.948545	-0.016001
H	8.961939	-0.134418	-0.018644
C	7.869015	1.720270	-0.012973
C	6.674140	1.042247	-0.013302
H	6.732657	-0.046393	-0.015509
C	5.350890	1.582592	-0.011399
H	5.233344	2.662038	-0.009810
C	4.231639	0.797416	-0.011762
H	4.374488	-0.283252	-0.013223
C	2.865391	1.250414	-0.010613
C	1.867481	0.303414	-0.010959
H	2.186456	-0.739070	-0.011723
C	0.456662	0.510545	-0.010611
H	0.075963	1.529636	-0.010501
C	-0.456661	-0.510519	-0.010587
H	-0.075963	-1.529611	-0.010549
C	-1.867479	-0.303387	-0.010815

H	-2.186455	0.739097	-0.011532
C	-2.865387	-1.250389	-0.010425
C	-4.231637	-0.797396	-0.011522
H	-4.374489	0.283271	-0.012909
C	-5.350886	-1.582575	-0.011227
H	-5.233338	-2.662021	-0.009740
C	-6.674137	-1.042232	-0.013088
H	-6.732656	0.046408	-0.015194
C	-7.869010	-1.720258	-0.012825
C	-9.086227	-0.948536	-0.015783
H	-8.961940	0.134427	-0.018325
C	-10.361492	-1.436750	-0.016042
H	-10.514363	-2.511708	-0.013717
C	-11.511507	-0.583643	-0.019914
H	-11.299221	0.485400	-0.022919
C	-12.834587	-0.943279	-0.021107
C	-13.815654	0.117536	-0.027175
H	-13.439691	1.139395	-0.034998
C	-15.164658	-0.021000	-0.025047
H	-15.619255	-1.004536	-0.028798
C	13.314436	2.373465	-0.018051
H	13.928120	2.579348	-0.902968
H	12.495848	3.093667	-0.007279
H	13.942827	2.570412	0.858542
C	7.968632	3.226567	-0.009821
H	6.992964	3.712613	-0.007494
H	8.517327	3.578964	0.871401
H	8.515132	3.582831	-0.890848
C	2.595488	2.735847	-0.009204
H	3.040114	3.213208	-0.890152
H	1.531120	2.970372	-0.008471
H	3.040911	3.211647	0.872191
C	-2.595473	-2.735820	-0.009094
H	-3.039609	-3.213057	-0.890360
H	-1.531103	-2.970334	-0.007793
H	-3.041377	-3.211751	0.871983
C	-7.968622	-3.226555	-0.009812
H	-8.515097	-3.582740	-0.890887
H	-6.992953	-3.712598	-0.007502
H	-8.517343	-3.579033	0.871363
C	-13.314429	-2.373468	-0.017926
H	-13.927582	-2.579473	-0.903187
H	-12.495844	-3.093664	-0.006555
H	-13.943344	-2.570300	0.858312
O	15.561105	-2.307434	-0.141707
O	-15.561090	2.307445	-0.141290

C	-16.033792	1.173169	-0.050600
C	17.526205	-0.995758	0.017537
C	18.325095	-2.114154	-0.269949
C	18.152891	0.209499	0.369339
C	19.712581	-2.027539	-0.222397
H	17.825267	-3.041507	-0.527646
C	19.544084	0.294437	0.425927
H	17.563602	1.083989	0.622916
C	20.326725	-0.820952	0.125453
H	20.318257	-2.898921	-0.454020
H	20.016178	1.231445	0.706596
C	-17.526212	0.995737	0.017470
C	-18.325062	2.114255	-0.269662
C	-18.152948	-0.209657	0.368702
C	-19.712553	2.027635	-0.222295
H	-17.825195	3.041704	-0.526936
C	-19.544149	-0.294608	0.425093
H	-17.563702	-1.084267	0.621968
C	-20.326748	0.820911	0.124994
H	-20.318195	2.899117	-0.453629
H	-20.016281	-1.231731	0.705313
H	21.410073	-0.751858	0.165134
H	-21.410101	0.751811	0.164527

Compound **2b**

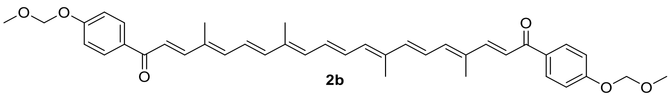

2b

Symbol	X	Y	Z
C	-10.437297	-0.405436	-0.129787
H	-10.732556	-1.447824	-0.136248
C	-9.126796	-0.061048	-0.134121
H	-8.912793	1.006489	-0.134105
C	-7.993407	-0.959458	-0.138573
C	-6.741588	-0.401581	-0.138701
H	-6.694835	0.687255	-0.135871
C	-5.474692	-1.070144	-0.141908
H	-5.463007	-2.155873	-0.144163
C	-4.288070	-0.395242	-0.141900
H	-4.327751	0.694032	-0.139964
C	-2.968710	-0.976189	-0.143851
C	-1.888363	-0.126947	-0.143604
H	-2.108071	0.940806	-0.142586
C	-0.501512	-0.465524	-0.144141

H	-0.218007	-1.515758	-0.144226
C	0.501508	0.465545	-0.144180
H	0.218003	1.515779	-0.144384
C	1.888359	0.126968	-0.143570
H	2.108067	-0.940785	-0.142423
C	2.968708	0.976207	-0.143882
C	4.288067	0.395257	-0.141806
H	4.327743	-0.694017	-0.139776
C	5.474690	1.070156	-0.141792
H	5.463009	2.155885	-0.144143
C	6.741584	0.401588	-0.138457
H	6.694827	-0.687247	-0.135557
C	7.993405	0.959461	-0.138306
C	9.126790	0.061046	-0.133809
H	8.912781	-1.006489	-0.133684
C	10.437293	0.405428	-0.129590
H	10.732560	1.447813	-0.136198
C	11.480773	-0.644067	-0.134302
C	-8.248444	-2.446196	-0.142988
H	-8.829951	-2.737409	-1.025622
H	-7.329484	-3.033016	-0.142908
H	-8.833052	-2.742272	0.735968
C	-2.841211	-2.480231	-0.145846
H	-1.803996	-2.814996	-0.146518
H	-3.329717	-2.913187	0.734786
H	-3.329995	-2.910792	-1.027507
C	2.841217	2.480250	-0.146033
H	3.330298	2.910741	-1.027561
H	1.804005	2.815021	-0.147082
H	3.329432	2.913271	0.734731
C	8.248448	2.446197	-0.142827
H	8.829386	2.737452	-1.025826
H	7.329491	3.033023	-0.142108
H	8.833626	2.742224	0.735762
C	-11.480781	0.644055	-0.134533
O	11.177102	-1.838120	-0.185565
C	12.924001	-0.244794	-0.086446
C	13.884235	-1.260114	-0.215925
C	13.377977	1.073050	0.097315
C	15.247362	-0.989532	-0.177404
H	13.528711	-2.277577	-0.337290
C	14.735740	1.362657	0.133876
H	12.676050	1.890279	0.220305
C	15.676521	0.335410	-0.012304
H	15.967063	-1.796309	-0.238702
H	15.092032	2.378490	0.268387

O	-11.177115	1.838104	-0.185902
C	-12.924008	0.244785	-0.086584
C	-13.884245	1.260111	-0.215986
C	-13.377981	-1.073059	0.097182
C	-15.247372	0.989539	-0.177376
H	-13.528723	2.277574	-0.337363
C	-14.735744	-1.362658	0.133828
H	-12.676054	-1.890299	0.220100
C	-15.676528	-0.335403	-0.012268
H	-15.967070	1.796323	-0.238613
H	-15.092033	-2.378492	0.268341
O	-16.990628	-0.725886	0.028187
O	16.990621	0.725896	0.028069
C	17.988873	-0.173605	-0.437921
H	18.872231	0.463680	-0.576701
H	17.681534	-0.622433	-1.388128
C	-17.988917	0.173631	-0.437692
H	-18.872281	-0.463655	-0.576434
H	-17.681652	0.622501	-1.387903
O	18.255105	-1.232700	0.426494
O	-18.255099	1.232685	0.426789
C	18.823267	-0.835220	1.670792
H	18.130681	-0.215424	2.250544
H	19.039737	-1.751300	2.222600
H	19.757930	-0.276431	1.517604
C	-18.823165	0.835145	1.671112
H	-18.130528	0.215334	2.250787
H	-19.039608	1.751198	2.222975
H	-19.757831	0.276349	1.517968

Compound **3b**

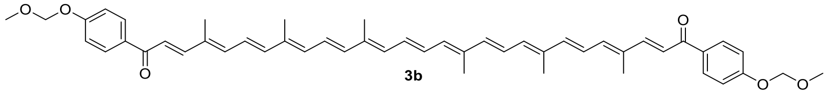

Symbol	X	Y	Z
C	16.069633	0.636710	0.212364
C	15.159094	-0.526348	0.135081
H	15.576737	-1.525354	0.096986
C	13.816445	-0.340699	0.132285
H	13.476647	0.692614	0.180453
C	12.798571	-1.365647	0.072890
C	11.488917	-0.961115	0.077265
H	11.313059	0.113442	0.125439

C	10.310362	-1.773471	0.026762
H	10.427123	-2.851890	-0.021931
C	9.052220	-1.243348	0.037841
H	8.963659	-0.157995	0.087257
C	7.810036	-1.973229	-0.009061
C	6.637949	-1.257082	0.011798
H	6.731789	-0.172094	0.062671
C	5.297777	-1.753035	-0.024592
H	5.145448	-2.826979	-0.074817
C	4.204338	-0.932819	0.001435
H	4.381664	0.141557	0.051928
C	2.824260	-1.340768	-0.031019
C	1.856935	-0.363210	0.003132
H	2.208884	0.667423	0.053038
C	0.440236	-0.524432	-0.018518
H	0.027307	-1.529709	-0.067847
C	-0.440237	0.524367	0.019585
H	-0.027307	1.529645	0.068895
C	-1.856936	0.363146	-0.002063
H	-2.208885	-0.667486	-0.051986
C	-2.824261	1.340704	0.032101
C	-4.204338	0.932756	-0.000397
H	-4.381664	-0.141621	-0.050855
C	-5.297776	1.752978	0.025521
H	-5.145445	2.826926	0.075655
C	-6.637948	1.257028	-0.010922
H	-6.731789	0.172039	-0.061768
C	-7.810033	1.973181	0.009839
C	-9.052217	1.243308	-0.037189
H	-8.963657	0.157951	-0.086523
C	-10.310355	1.773443	-0.026359
H	-10.427113	2.851868	0.022210
C	-11.488910	0.961095	-0.076988
H	-11.313055	-0.113468	-0.125052
C	-12.798561	1.365640	-0.072848
C	-13.816436	0.340698	-0.132319
H	-13.476640	-0.692622	-0.180363
C	-15.159083	0.526359	-0.135320
H	-15.576723	1.525371	-0.097363
C	13.228927	-2.810196	0.010709
H	13.829899	-3.074452	0.888838
H	12.386248	-3.500706	-0.034424
H	13.855601	-2.991173	-0.870563
C	7.860354	-3.480439	-0.078143
H	6.869408	-3.933356	-0.111354
H	8.406941	-3.811601	-0.968866

H	8.385349	-3.893107	0.791298
C	2.507092	-2.815236	-0.100564
H	2.927229	-3.344975	0.762301
H	1.435817	-3.014860	-0.121071
H	2.946142	-3.266065	-0.998148
C	-2.507097	2.815172	0.101633
H	-2.946651	3.266126	0.998903
H	-1.435830	3.014775	0.122739
H	-2.926729	3.344811	-0.761544
C	-7.860350	3.480395	0.078851
H	-8.407694	3.811584	0.969095
H	-6.869415	3.933271	0.112938
H	-8.384577	3.893079	-0.791051
C	-13.228912	2.810198	-0.010843
H	-13.855697	2.991255	0.870335
H	-12.386234	3.500706	0.034339
H	-13.829771	3.074385	-0.889070
O	15.627413	1.782631	0.323420
O	-15.627402	-1.782622	-0.323633
C	-16.069623	-0.636694	-0.212656
C	17.550837	0.412965	0.167203
C	18.383319	1.520605	0.389510
C	18.158610	-0.825712	-0.102156
C	19.769288	1.413727	0.360798
H	17.909164	2.478144	0.575777
C	19.541445	-0.952062	-0.131300
H	17.558515	-1.706804	-0.300487
C	20.353003	0.163676	0.109300
H	20.388733	2.291625	0.495857
H	20.016231	-1.906606	-0.332338
C	-17.550830	-0.412932	-0.167688
C	-18.383296	-1.520550	-0.390159
C	-18.158621	0.825739	0.101659
C	-19.769268	-1.413655	-0.361624
H	-17.909132	-2.478087	-0.576408
C	-19.541459	0.952105	0.130632
H	-17.558539	1.706810	0.300121
C	-20.353000	-0.163609	-0.110135
H	-20.388706	-2.291539	-0.496809
H	-20.016258	1.906643	0.331665
O	21.704742	-0.066992	0.068528
O	-21.704740	0.067075	-0.069527
C	22.583252	0.901718	0.626819
H	23.533081	0.361971	0.737798
H	22.210786	1.235413	1.600861
C	-22.583191	-0.901606	-0.627962

H	-23.533010	-0.361853	-0.739014
H	-22.210622	-1.235249	-1.601982
O	-22.737490	-2.050033	0.145163
O	22.737475	2.050104	-0.146384
C	23.365368	1.820583	-1.404116
H	22.757418	1.173979	-2.046178
H	23.482296	2.796442	-1.878055
H	24.356147	1.361703	-1.273112
C	-23.365499	-1.820580	1.402849
H	-23.482490	-2.796467	1.876715
H	-22.757600	-1.174027	2.045010
H	-24.356257	-1.361674	1.271779

Compound **2c**

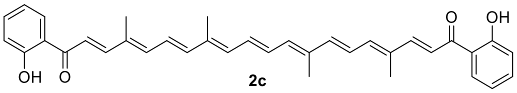

2c

Symbol	X	Y	Z
C	-10.430585	-0.432662	0.000051
H	-10.720654	-1.475158	-0.000083
C	-9.118945	-0.084212	0.000193
H	-8.902133	0.982268	0.000299
C	-7.988253	-0.983046	0.000178
C	-6.736245	-0.423162	0.000221
H	-6.690710	0.665704	0.000303
C	-5.469506	-1.089921	0.000163
H	-5.455512	-2.175498	0.000036
C	-4.284500	-0.411196	0.000225
H	-4.327526	0.677901	0.000306
C	-2.964072	-0.988394	0.000160
C	-1.887160	-0.134276	0.000116
H	-2.111009	0.932543	0.000175
C	-0.499491	-0.467879	-0.000022
H	-0.211908	-1.516915	-0.000103
C	0.499485	0.467822	-0.000078
H	0.211902	1.516857	0.000003
C	1.887154	0.134218	-0.000217
H	2.111003	-0.932600	-0.000276
C	2.964066	0.988336	-0.000260
C	4.284494	0.411139	-0.000323
H	4.327519	-0.677959	-0.000406
C	5.469500	1.089863	-0.000255
H	5.455506	2.175440	-0.000125
C	6.736238	0.423103	-0.000312

H	6.690702	-0.665762	-0.000396
C	7.988247	0.982987	-0.000262
C	9.118938	0.084151	-0.000271
H	8.902124	-0.982329	-0.000385
C	10.430578	0.432600	-0.000119
H	10.720654	1.475095	0.000024
C	11.474713	-0.601983	-0.000128
C	-8.242416	-2.469897	0.000061
H	-8.824052	-2.764558	-0.881283
H	-7.322769	-3.055444	0.000466
H	-8.824821	-2.764546	0.880892
C	-2.831354	-2.491860	0.000071
H	-1.793051	-2.823061	0.000685
H	-3.318803	-2.925308	0.880975
H	-3.317747	-2.925122	-0.881514
C	2.831347	2.491802	-0.000170
H	3.318812	2.925252	-0.881064
H	1.793044	2.823003	-0.000803
H	3.317724	2.925063	0.881424
C	8.242412	2.469837	-0.000140
H	8.824838	2.764484	-0.880958
H	7.322765	3.055385	-0.000569
H	8.824027	2.764498	0.881217
C	-11.474722	0.601924	0.000056
O	11.142767	-1.811893	-0.000237
C	12.904438	-0.231713	0.000064
C	13.881120	-1.273212	0.000155
C	13.361149	1.104664	0.000157
C	15.249946	-0.949305	0.000313
C	14.710878	1.413784	0.000315
H	12.643447	1.916849	0.000094
C	15.655445	0.374633	0.000393
H	15.964004	-1.765822	0.000372
H	15.034235	2.449438	0.000378
O	-11.142804	1.811840	0.000259
C	-12.904452	0.231702	-0.000031
C	-13.881021	1.273289	0.000045
C	-13.361281	-1.104634	-0.000195
C	-15.249880	0.949526	-0.000056
C	-14.711043	-1.413620	-0.000290
H	-12.643659	-1.916888	-0.000253
C	-15.655508	-0.374374	-0.000223
H	-15.963855	1.766115	0.000011
H	-15.034502	-2.449242	-0.000418
H	16.716418	0.608623	0.000519
H	-16.716504	-0.608259	-0.000298

H	-12.543785	2.582979	0.000346
H	12.544062	-2.583075	0.000027
O	-13.544149	2.566568	0.000232
O	13.544427	-2.566533	0.000108

Compound **2d**

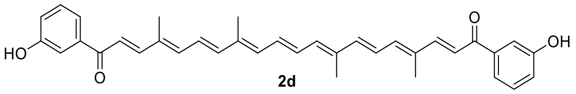

2d

Symbol	X	Y	Z
C	10.443909	-0.006138	-0.072841
H	10.783144	1.022714	-0.080358
C	9.120324	-0.299027	-0.075317
H	8.863389	-1.357108	-0.079004
C	8.024311	0.643652	-0.074096
C	6.751264	0.135203	-0.072344
H	6.661466	-0.950939	-0.071682
C	5.512208	0.853456	-0.071857
H	5.543636	1.938738	-0.072457
C	4.299821	0.225710	-0.071026
H	4.296497	-0.864271	-0.070717
C	3.004661	0.858455	-0.070727
C	1.891723	0.052236	-0.070309
H	2.069120	-1.023334	-0.070194
C	0.519461	0.445453	-0.070194
H	0.277713	1.506046	-0.070248
C	-0.519454	-0.445463	-0.070183
H	-0.277706	-1.506056	-0.070194
C	-1.891716	-0.052246	-0.070322
H	-2.069113	1.023324	-0.070245
C	-3.004655	-0.858464	-0.070719
C	-4.299814	-0.225720	-0.071037
H	-4.296490	0.864261	-0.070772
C	-5.512202	-0.853465	-0.071828
H	-5.543629	-1.938748	-0.072380
C	-6.751257	-0.135212	-0.072334
H	-6.661459	0.950929	-0.071725
C	-8.024304	-0.643661	-0.074050
C	-9.120317	0.299019	-0.075320
H	-8.863382	1.357099	-0.079046
C	-10.443902	0.006130	-0.072853
H	-10.783131	-1.022724	-0.080333
C	-11.44486	1.093074	-0.091836

C	8.338437	2.118972	-0.075869
H	8.926072	2.389538	-0.960940
H	7.443540	2.741777	-0.068681
H	8.939414	2.388976	0.800341
C	2.936646	2.366309	-0.071032
H	1.913481	2.741771	-0.070372
H	3.442541	2.778607	0.809669
H	3.441254	2.778193	-0.952668
C	-2.936639	-2.366318	-0.070969
H	-3.441342	-2.778238	-0.952534
H	-1.913475	-2.741781	-0.070407
H	-3.442440	-2.778582	0.809803
C	-8.338430	-2.118981	-0.075752
H	-8.925970	-2.389613	-0.960866
H	-7.443534	-2.741786	-0.068420
H	-8.939502	-2.388921	0.800413
C	11.444869	-1.093081	-0.091777
O	-11.106066	2.273943	-0.177926
C	-12.908155	0.745126	-0.022948
C	-13.831050	1.764200	-0.310855
C	-13.374158	-0.526845	0.329212
C	-15.194870	1.502205	-0.258183
H	-13.449711	2.744759	-0.569851
C	-14.747524	-0.780926	0.388931
H	-12.701845	-1.336956	0.584916
C	-15.662183	0.232450	0.090063
H	-15.909146	2.287565	-0.487372
O	11.106087	-2.273952	-0.177877
C	12.908157	-0.745118	-0.022846
C	13.831075	-1.764137	-0.310875
C	13.374128	0.526818	0.329484
C	15.194889	-1.502118	-0.258161
H	13.449760	-2.744674	-0.569994
C	14.747489	0.780920	0.389249
H	12.701789	1.336874	0.585296
C	15.662171	-0.232398	0.090253
H	15.909184	-2.287432	-0.487449
H	16.730697	-0.031392	0.132560
H	-16.730714	0.031462	0.132338
O	-15.131710	-2.044108	0.747601
H	-16.096636	-2.089550	0.760778
O	15.131647	2.044061	0.748091
H	16.096571	2.089516	0.761296

Compound **2e**

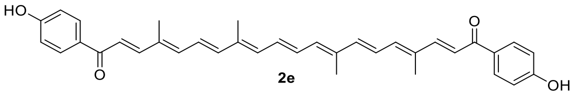

2e

Symbol	X	Y	Z
C	-10.443459	-0.185009	-0.022040
H	-10.760010	-1.221118	-0.025769
C	-9.125959	0.131850	-0.025651
H	-8.889522	1.194626	-0.027599
C	-8.011826	-0.790219	-0.027307
C	-6.748528	-0.258747	-0.027813
H	-6.678835	0.828866	-0.027041
C	-5.496059	-0.953904	-0.029247
H	-5.507338	-2.039629	-0.029954
C	-4.295442	-0.304200	-0.029630
H	-4.312064	0.785671	-0.029064
C	-2.988703	-0.912989	-0.030533
C	-1.890612	-0.086803	-0.030611
H	-2.087595	0.985370	-0.030265
C	-0.511273	-0.454789	-0.030880
H	-0.250173	-1.510807	-0.030918
C	0.511274	0.454789	-0.030888
H	0.250174	1.510807	-0.030941
C	1.890613	0.086803	-0.030617
H	2.087595	-0.985371	-0.030256
C	2.988704	0.912988	-0.030554
C	4.295443	0.304199	-0.029646
H	4.312064	-0.785672	-0.029063
C	5.496060	0.953903	-0.029277
H	5.507339	2.039627	-0.030000
C	6.748529	0.258745	-0.027836
H	6.678835	-0.828868	-0.027044
C	8.011827	0.790216	-0.027343
C	9.125959	-0.131853	-0.025668
H	8.889521	-1.194629	-0.027601
C	10.443459	0.185005	-0.022054
H	10.760012	1.221114	-0.025798
C	11.464379	-0.885860	-0.028876
C	-8.298031	-2.271279	-0.028702
H	-8.885182	-2.552181	-0.910939
H	-7.391581	-2.877220	-0.027044
H	-8.888904	-2.553230	0.850707
C	-2.893108	-2.419381	-0.031203
H	-1.863225	-2.776036	-0.031683
H	-3.390517	-2.841076	0.849906
H	-3.391026	-2.840284	-0.912406

C	2.893109	2.419380	-0.031247
H	3.391019	2.840270	-0.912460
H	1.863226	2.776035	-0.031722
H	3.390526	2.841089	0.849851
C	8.298034	2.271276	-0.028763
H	8.885224	2.552154	-0.910982
H	7.391584	2.877218	-0.027164
H	8.888868	2.553250	0.850665
C	-11.464379	0.885856	-0.028881
O	11.135817	-2.073838	-0.075616
C	12.915376	-0.518132	0.013187
C	13.850698	-1.559692	-0.103049
C	13.401580	0.791529	0.170657
C	15.215399	-1.310566	-0.071723
H	13.471405	-2.569117	-0.218223
C	14.765508	1.055412	0.207694
H	12.717785	1.626076	0.277998
C	15.679633	0.002938	0.083875
H	15.925557	-2.129639	-0.166728
H	15.142142	2.064894	0.332745
O	-11.135815	2.073834	-0.075597
C	-12.915376	0.518130	0.013176
C	-13.850695	1.559700	-0.102998
C	-13.401586	-0.791537	0.170575
C	-15.215397	1.310579	-0.071676
H	-13.471398	2.569130	-0.218122
C	-14.765515	-1.055417	0.207605
H	-12.717795	-1.626095	0.277860
C	-15.679636	-0.002931	0.083851
H	-15.925552	2.129661	-0.166631
H	-15.142152	-2.064904	0.332599
O	-17.005157	-0.314219	0.122911
O	17.005153	0.314229	0.122943
H	-17.524720	0.495581	0.030266
H	17.524719	-0.495563	0.030252

Compound **2f**

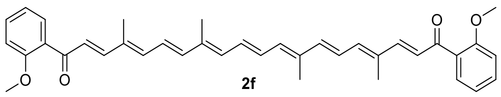

2f

Symbol	X	Y	Z
C	-10.446800	0.078102	-0.124140
H	-10.803700	-0.942340	-0.105220

C	-9.123390	0.371498	-0.127120
H	-8.860460	1.427843	-0.134280
C	-8.031260	-0.576770	-0.121300
C	-6.754790	-0.077620	-0.122110
H	-6.657360	1.007889	-0.125330
C	-5.520520	-0.804670	-0.120070
H	-5.560270	-1.889770	-0.118560
C	-4.302910	-0.187300	-0.120800
H	-4.290380	0.902641	-0.122500
C	-3.012920	-0.830770	-0.119870
C	-1.892470	-0.035110	-0.120370
H	-2.060140	1.042067	-0.121160
C	-0.523670	-0.440500	-0.120160
H	-0.291520	-1.503290	-0.120150
C	0.523672	0.440496	-0.120170
H	0.291521	1.503289	-0.120090
C	1.892473	0.035107	-0.120470
H	2.060140	-1.042070	-0.121330
C	3.012915	0.830771	-0.119990
C	4.302910	0.187295	-0.121040
H	4.290373	-0.902650	-0.122790
C	5.520517	0.804658	-0.120350
H	5.560271	1.889765	-0.118790
C	6.754788	0.077611	-0.122500
H	6.657355	-1.007900	-0.125770
C	8.031255	0.576751	-0.121740
C	9.123389	-0.371502	-0.127670
H	8.860456	-1.427860	-0.134870
C	10.446800	-0.078120	-0.124750
H	10.803650	0.942315	-0.105800
C	11.444590	-1.167430	-0.162120
C	-8.354730	-2.050120	-0.116480
H	-8.942810	-2.320480	-1.001240
H	-7.464060	-2.679040	-0.103470
H	-8.961190	-2.309660	0.758968
C	-2.957980	-2.339270	-0.118570
H	-1.938070	-2.723590	-0.117720
H	-3.467310	-2.746290	0.762666
H	-3.466500	-2.747770	-0.999570
C	2.957978	2.339264	-0.118610
H	3.466420	2.747811	-0.999640
H	1.938074	2.723588	-0.117650
H	3.467393	2.746238	0.762599
C	8.354735	2.050108	-0.116860

H	8.942787	2.320498	-1.001640
H	7.464064	2.679031	-0.103810
H	8.961218	2.309604	0.758574
C	-11.44460	1.167405	-0.161400
O	11.09686	-2.346230	-0.253670
C	12.91901	-0.848380	-0.177280
C	13.74378	-1.801360	-0.792450
C	13.53260	0.295715	0.388158
C	15.12106	-1.634190	-0.891960
H	13.25723	-2.684320	-1.192330
C	14.92071	0.465062	0.295888
C	15.70514	-0.492230	-0.346700
H	15.73019	-2.383960	-1.386480
H	15.39684	1.336077	0.728525
O	-11.09690	2.346212	-0.252920
C	-12.91900	0.848365	-0.176420
C	-13.74380	1.801327	-0.791550
C	-13.53260	-0.295700	0.389135
C	-15.12110	1.634162	-0.890940
H	-13.25730	2.684257	-1.191530
C	-14.92070	-0.465030	0.296993
C	-15.70520	0.492234	-0.345570
H	-15.73030	2.383911	-1.385440
H	-15.39680	-1.336020	0.729721
H	16.77880	-0.339770	-0.411110
H	-16.77880	0.339780	-0.409880
O	-12.72580	-1.186170	1.040561
O	12.72590	1.186218	1.039597
C	13.31507	2.310785	1.675905
H	14.03055	2.006422	2.449327
H	13.81914	2.968110	0.956566
H	12.49155	2.853383	2.142079
C	-13.31500	-2.310690	1.676982
H	-14.03040	-2.006270	2.450442
H	-13.81910	-2.968050	0.957721
H	-12.49140	-2.853280	2.143120

Compound **2g**

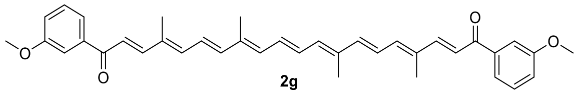

Symbol	X	Y	Z

C	10.442347	-0.206177	-0.118331
H	10.801037	0.816073	-0.123474
C	9.113626	-0.474051	-0.122292
H	8.836822	-1.527103	-0.127924
C	8.035416	0.489190	-0.120450
C	6.752976	0.005145	-0.120237
H	6.642491	-1.079089	-0.121071
C	5.527758	0.746907	-0.119593
H	5.579914	1.831401	-0.119163
C	4.303565	0.142592	-0.119826
H	4.279282	-0.947127	-0.120422
C	3.020763	0.800120	-0.119495
C	1.892437	0.015628	-0.119654
H	2.049006	-1.063178	-0.119941
C	0.527998	0.435290	-0.119566
H	0.306818	1.500369	-0.119549
C	-0.527996	-0.435289	-0.119569
H	-0.306816	-1.500368	-0.119548
C	-1.892434	-0.015626	-0.119668
H	-2.049003	1.063179	-0.119956
C	-3.020760	-0.800118	-0.119516
C	-4.303562	-0.142590	-0.119850
H	-4.279279	0.947128	-0.120449
C	-5.527755	-0.746906	-0.119612
H	-5.579911	-1.831400	-0.119176
C	-6.752973	-0.005144	-0.120256
H	-6.642488	1.079090	-0.121098
C	-8.035413	-0.489189	-0.120464
C	-9.113623	0.474052	-0.122317
H	-8.836819	1.527104	-0.127950
C	-10.442344	0.206178	-0.118366
H	-10.801032	-0.816072	-0.123508
C	-11.422987	1.311997	-0.137806
C	8.377494	1.958279	-0.119908
H	8.970823	2.218821	-1.004192
H	7.494599	2.597998	-0.112386
H	8.982964	2.215483	0.757067
C	2.981866	2.309016	-0.119034
H	1.966147	2.704195	-0.118699
H	3.495173	2.711026	0.762146
H	3.494824	2.711552	-1.000176
C	-2.981863	-2.309015	-0.119053
H	-3.494864	-2.711553	-1.000168
H	-1.966144	-2.704194	-0.118768
H	-3.495127	-2.711023	0.762153
C	-8.377491	-1.958278	-0.119912

H	-8.970771	-2.218839	-1.004225
H	-7.494596	-2.597997	-0.112327
H	-8.983009	-2.215464	0.757034
C	11.422991	-1.311995	-0.137764
O	-11.061243	2.486008	-0.224824
C	-12.892081	0.992524	-0.067675
C	-13.797982	2.031004	-0.347746
C	-13.383413	-0.268620	0.278254
C	-15.163423	1.791395	-0.291545
H	-13.400083	3.005960	-0.603133
C	-14.763694	-0.503480	0.342547
H	-12.728169	-1.094845	0.527190
C	-15.661302	0.528579	0.051930
H	-15.863924	2.591079	-0.514552
O	11.061248	-2.486007	-0.224791
C	12.892082	-0.992521	-0.067609
C	13.797990	-2.030986	-0.347713
C	13.383406	0.268610	0.278379
C	15.163430	-1.791374	-0.291489
H	13.400098	-3.005934	-0.603142
C	14.763685	0.503473	0.342696
H	12.728154	1.094820	0.527346
C	15.661300	-0.528570	0.052043
H	15.863936	-2.591045	-0.514523
H	16.731761	-0.366139	0.091609
H	-16.731764	0.366151	0.091478
O	-15.125281	-1.772611	0.699262
O	15.125264	1.772590	0.699470
C	-16.508424	-2.074365	0.791213
H	-16.569038	-3.121848	1.089887
H	-17.009512	-1.454907	1.546157
H	-17.017272	-1.944972	-0.172721
C	16.508405	2.074344	0.791450
H	17.017265	1.944996	-0.172483
H	16.569012	3.121814	1.090172
H	17.009486	1.454854	1.546373

Compound **2h**

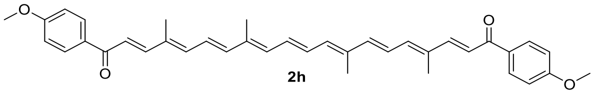

Symbol	X	Y	Z
C	10.442772	0.243212	-0.021907
H	10.753263	1.281164	-0.026335

C	9.127286	-0.081474	-0.025622
H	8.897208	-1.145658	-0.026989
C	8.007620	0.834060	-0.028053
C	6.747408	0.295440	-0.028504
H	6.683907	-0.792554	-0.027158
C	5.490962	0.983504	-0.030527
H	5.496196	2.069283	-0.031683
C	4.293933	0.327270	-0.030886
H	4.316487	-0.762499	-0.029860
C	2.983854	0.928914	-0.032205
C	1.890185	0.096916	-0.032057
H	2.092912	-0.974194	-0.031399
C	0.508861	0.457473	-0.032383
H	0.242115	1.512089	-0.032531
C	-0.508853	-0.457492	-0.032248
H	-0.242106	-1.512107	-0.032197
C	-1.890177	-0.096936	-0.031926
H	-2.092905	0.974174	-0.031500
C	-2.983846	-0.928934	-0.031875
C	-4.293926	-0.327290	-0.030785
H	-4.316483	0.762479	-0.030029
C	-5.490954	-0.983528	-0.030406
H	-5.496182	-2.069307	-0.031312
C	-6.747402	-0.295469	-0.028652
H	-6.683906	0.792526	-0.027488
C	-8.007613	-0.834093	-0.028221
C	-9.127282	0.081437	-0.025931
H	-8.897210	1.145622	-0.027531
C	-10.442766	-0.243256	-0.022031
H	-10.753239	-1.281214	-0.026143
C	-11.470640	0.821552	-0.028254
C	8.285285	2.316760	-0.030253
H	8.870829	2.600502	-0.912650
H	7.375388	2.917524	-0.028838
H	8.874674	2.602492	0.848929
C	2.880127	2.434789	-0.033375
H	1.848327	2.785874	-0.035432
H	3.374048	2.859395	0.848314
H	3.377018	2.858151	-0.913977
C	-2.880119	-2.434808	-0.032714
H	-3.376079	-2.858314	-0.913778
H	-1.848318	-2.785895	-0.033581
H	-3.374973	-2.859269	0.848515
C	-8.285272	-2.316795	-0.030163
H	-8.871764	-2.600494	-0.911937
H	-7.375370	-2.917554	-0.029786

H	-8.873713	-2.602576	0.849646
C	11.470644	-0.821601	-0.028038
O	-11.148593	2.011326	-0.075201
C	-12.918779	0.443858	0.014425
C	-13.862296	1.474569	-0.103901
C	-13.396242	-0.870411	0.174640
C	-15.229774	1.223099	-0.073473
H	-13.490701	2.486796	-0.221186
C	-14.756415	-1.138893	0.211122
H	-12.707930	-1.700992	0.284467
C	-15.685049	-0.094298	0.084780
H	-15.927139	2.046433	-0.171637
H	-15.129353	-2.149667	0.338143
O	11.148606	-2.011379	-0.074913
C	12.918776	-0.443884	0.014589
C	13.862316	-1.474504	-0.104349
C	13.396203	0.870326	0.175386
C	15.229787	-1.222988	-0.074004
H	13.490748	-2.486693	-0.222044
C	14.756371	1.138846	0.211821
H	12.707860	1.700807	0.285778
C	15.685029	0.094352	0.084823
H	15.927174	-2.046243	-0.172672
H	15.129286	2.149569	0.339312
O	16.994592	0.460754	0.129463
O	-16.994620	-0.460666	0.129436
C	-17.985491	0.551141	0.010717
H	-17.916256	1.072879	-0.951717
H	-18.945993	0.038240	0.073372
H	-17.913179	1.284133	0.823575
C	17.985487	-0.550956	0.010122
H	17.916141	-1.072234	-0.952553
H	18.945977	-0.038046	0.072890
H	17.913316	-1.284341	0.822637

Compound **2i**

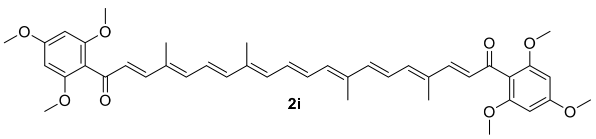

Symbol	X	Y	Z
C	0.513693	-0.452027	-0.126553
C	-0.513689	0.452028	-0.126622

C	1.891665	-0.078151	-0.126705
C	-1.891660	0.078151	-0.126559
H	-0.257436	1.509333	-0.126676
H	0.257440	-1.509332	-0.126296
C	2.993886	-0.898592	-0.125704
H	2.083765	0.994992	-0.127644
C	-2.993879	0.898593	-0.125694
H	-2.083761	-0.994992	-0.127216
C	2.904239	-2.405515	-0.123930
C	4.298937	-0.285494	-0.126220
C	-2.904224	2.405517	-0.124312
C	-4.298932	0.285498	-0.126043
H	1.875704	-2.766210	-0.124562
H	3.402884	-2.823890	0.758144
H	3.404704	-2.826070	-1.003929
C	5.501600	-0.930929	-0.124563
H	4.311726	0.804486	-0.128016
H	-3.403488	2.824146	0.757285
H	-3.404064	2.825824	-1.004789
H	-1.875686	2.766204	-0.124300
C	-5.501595	0.930932	-0.124627
H	-4.311722	-0.804482	-0.127525
C	6.753997	-0.234422	-0.125110
H	5.515311	-2.016757	-0.122393
C	-6.753992	0.234423	-0.124991
H	-5.515308	2.016760	-0.122819
C	8.017139	-0.764816	-0.123302
H	6.683399	0.853198	-0.127368
C	-8.017139	0.764811	-0.123412
H	-6.683389	-0.853197	-0.126880
C	8.303528	-2.245912	-0.119721
C	9.135200	0.154539	-0.125065
C	-8.303543	2.245905	-0.120341
C	-9.135191	-0.154554	-0.124927
H	8.887143	-2.525388	0.765228
H	7.397448	-2.852584	-0.126327
H	8.901300	-2.526185	-0.994779
C	10.449694	-0.169073	-0.121297
H	8.901509	1.218052	-0.128587
H	-8.901186	2.525895	-0.995583
H	-7.397469	2.852584	-0.127041
H	-8.887294	2.525654	0.764427
C	-10.449687	0.169052	-0.121388
H	-8.901490	-1.218066	-0.128054
C	11.484456	0.891064	-0.093001
H	10.784686	-1.199820	-0.124295

C	-11.484442	-0.891084	-0.092779
H	-10.784685	1.199795	-0.124778
C	12.924986	0.452544	-0.017112
O	11.190597	2.082298	-0.087560
C	-12.924977	-0.452553	-0.017015
O	-11.190578	-2.082315	-0.087055
C	13.472706	-0.464971	-0.939393
C	13.789310	0.991249	0.956576
C	-13.789269	-0.990924	0.956887
C	-13.472739	0.464623	-0.939607
C	14.816720	-0.833136	-0.900200
O	12.604859	-0.937875	-1.882510
C	15.143277	0.626641	1.019924
O	13.222944	1.842662	1.851483
C	-15.143240	-0.626315	1.020140
O	-13.222870	-1.841999	1.852092
C	-14.816757	0.832776	-0.900516
O	-12.604936	0.937186	-1.882938
C	15.643333	-0.281333	0.083871
H	15.253935	-1.521831	-1.609637
C	13.090992	-1.833815	-2.870242
H	15.783998	1.039261	1.784084
C	14.033099	2.437146	2.850118
C	-15.643337	0.281314	0.083774
H	-15.783931	-1.038676	1.784467
C	-14.033013	-2.436164	2.850926
H	-15.254000	1.521204	-1.610197
C	-13.091142	1.832734	-2.870990
O	16.941574	-0.699087	0.047326
H	13.473677	-2.760689	-2.425061
H	13.880695	-1.373662	-3.476447
H	12.236733	-2.065620	-3.507765
H	14.466783	1.687487	3.525064
H	13.369723	3.089647	3.419131
H	14.840225	3.038545	2.413097
O	-16.941588	0.699028	0.047106
H	-14.840132	-3.037717	2.414107
H	-14.466704	-1.686288	3.525627
H	-13.369626	-3.088468	3.420152
H	-13.880863	1.372328	-3.476979
H	-13.473829	2.759768	-2.426144
H	-12.236921	2.064316	-3.508643
C	17.851301	-0.172657	1.000723
C	-17.851289	0.172932	1.000712
H	17.941889	0.917460	0.915001
H	17.557429	-0.429621	2.026343

H	18.816173	-0.631166	0.779658
H	-17.941865	-0.917217	0.915390
H	-17.557398	0.430273	2.026233
H	-18.816172	0.631348	0.779502

Compound **2j**

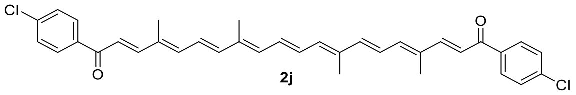

Symbol	X	Y	Z
C	10.440533	0.146387	-0.045019
H	10.765539	1.179954	-0.051831
C	9.119965	-0.162622	-0.048056
H	8.876768	-1.223884	-0.051620
C	8.012458	0.765349	-0.047511
C	6.746286	0.239174	-0.046411
H	6.671757	-0.848094	-0.045722
C	5.497525	0.939700	-0.046587
H	5.512865	2.025290	-0.047325
C	4.294683	0.293487	-0.046144
H	4.308065	-0.796389	-0.045699
C	2.990222	0.906376	-0.046321
C	1.890322	0.082278	-0.046023
H	2.084765	-0.990303	-0.045768
C	0.512174	0.453826	-0.046081
H	0.253566	1.510392	-0.046178
C	-0.512166	-0.453895	-0.046069
H	-0.253558	-1.510461	-0.046057
C	-1.890315	-0.082346	-0.046107
H	-2.084754	0.990235	-0.045944
C	-2.990217	-0.906441	-0.046375
C	-4.294675	-0.293548	-0.046246
H	-4.308054	0.796328	-0.045917
C	-5.497518	-0.939758	-0.046567
H	-5.512861	-2.025347	-0.047163
C	-6.746278	-0.239228	-0.046428
H	-6.671745	0.848040	-0.045880
C	-8.012452	-0.765398	-0.047407
C	-9.119954	0.162578	-0.048035
H	-8.876754	1.223838	-0.051730
C	-10.440524	-0.146425	-0.044931
H	-10.765524	-1.179994	-0.051604
C	-11.452348	0.928228	-0.062021
C	8.306277	2.244851	-0.049335

H	8.890779	2.523527	-0.934008
H	7.402832	2.855129	-0.043471
H	8.901813	2.523639	0.827917
C	2.898656	2.412917	-0.046893
H	1.869781	2.772353	-0.046799
H	3.397491	2.833175	0.834046
H	3.397060	2.832450	-0.928420
C	-2.898661	-2.412983	-0.046797
H	-3.397531	-2.832622	-0.928006
H	-1.869790	-2.772429	-0.047211
H	-3.397041	-2.833126	0.834459
C	-8.306278	-2.244899	-0.049031
H	-8.890728	-2.523703	-0.933699
H	-7.402837	-2.855181	-0.043020
H	-8.901868	-2.523553	0.828226
C	11.452365	-0.928261	-0.062027
O	-11.128940	2.114369	-0.140972
C	-12.910869	0.565671	0.002824
C	-13.842253	1.588108	-0.238107
C	-13.388266	-0.717874	0.305985
C	-15.209401	1.342432	-0.193711
H	-13.462933	2.579549	-0.459814
C	-14.756128	-0.979894	0.362257
H	-12.701451	-1.528536	0.521875
C	-15.655368	0.053856	0.106690
H	-15.925168	2.133664	-0.386663
H	-15.122045	-1.971560	0.603050
O	11.128980	-2.114410	-0.140937
C	12.910874	-0.565668	0.002831
C	13.842294	-1.588014	-0.238341
C	13.388219	0.717833	0.306261
C	15.209433	-1.342283	-0.193937
H	13.463015	-2.579428	-0.460237
C	14.756070	0.979902	0.362557
H	12.701363	1.528405	0.522364
C	15.655350	-0.053751	0.106732
H	15.925231	-2.133439	-0.387083
H	15.121951	1.971528	0.603567
Cl	-17.380860	-0.270337	0.167881
Cl	17.380829	0.270506	0.167939

3. LUMO coefficient of 2b.

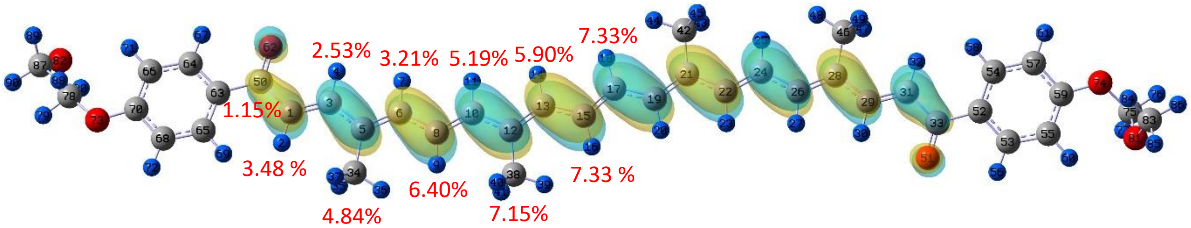

DFT calculation was performed with the Gaussian 16 program. The geometry optimization of compound **2b** was carried out at the B3LYP/6–31g(d,p) levels of theory. Calculation of the molecular orbital coefficient of the frontier orbitals was carried out by Multiwfn program (Lu, T.; Chen, F. Multiwfn: A Multifunctional Wavefunction Analyzer. *J. Comput. Chem.* **2012**, *33*, 580–592).

Atom	Coefficient	Atom	Coefficient	Atom	Coefficient
1 (C)	3.47706 %	31 (C)	3.47702 %	61 (H)	-0.00000 %
2 (H)	-0.00001 %	32 (H)	-0.00001 %	62 (O)	1.43182 %
3 (C)	2.52878 %	33 (C)	1.15381 %	63 (C)	0.02918 %
4 (H)	0.00004 %	34 (C)	-0.01162 %	64 (C)	0.31557 %
5 (C)	4.83798 %	35 (H)	0.12933 %	65(C)	0.15607 %
6 (C)	3.20956 %	36 (H)	0.00001 %	66 (C)	0.00406 %
7 (H)	0.00000 %	37 (H)	0.12968 %	67 (H)	0.00020 %
8 (C)	6.39990 %	38 (C)	-0.02036 %	68 (C)	0.01512 %
9 (H)	0.00000 %	39 (H)	0.00000 %	69 (H)	0.00358 %
10 (C)	5.18468 %	40 (H)	0.18073 %	70 (C)	0.24118 %
11 (H)	0.00000 %	41 (H)	0.18124 %	71 (H)	0.00004 %
12 (C)	7.15036 %	42 (C)	-0.02036 %	72 (H)	-0.00000 %
13 (C)	5.90123 %	43 (H)	0.18116 %	73 (O)	0.02813 %
14 (H)	0.00000 %	44 (H)	0.00000 %	74 (O)	0.02813 %
15 (C)	7.33370 %	45 (H)	0.18080 %	75 (C)	0.00182 %
16 (H)	0.00000 %	46 (C)	-0.01162 %	76 (H)	0.00054 %
17 (C)	7.33373 %	47 (H)	0.12944 %	77 (H)	0.00533 %
18 (H)	0.00000 %	48 (H)	0.00001 %	78 (C)	0.00182 %
19 (C)	5.90118 %	49 (H)	0.12957 %	79 (H)	0.00054 %
20 (H)	0.00000 %	50 (C)	1.15384 %	80 (H)	0.00533 %
21 (C)	7.15035 %	51 (O)	1.43180 %	81 (O)	0.00122 %
22 (C)	5.18462 %	52 (C)	0.02918 %	82 (O)	0.00122 %
23 (H)	0.00000 %	53 (C)	0.31556 %	83 (C)	0.00013 %
24 (C)	6.39986 %	54 (C)	0.15607 %	84 (H)	0.00001 %
25 (H)	0.00000 %	55 (C)	0.00406 %	85 (H)	0.00006 %
26 (C)	3.20952 %	56 (H)	0.00020 %	86 (H)	0.00001 %
27 (H)	0.00000 %	57 (C)	0.01512 %	87 (C)	0.00013 %
28 (C)	4.83792 %	58 (H)	0.00359 %	88 (H)	0.00001 %
29 (C)	2.52874 %	59 (C)	0.24117 %	89 (H)	0.00006 %
30 (H)	0.00004 %	60 (H)	0.00004 %	90 (H)	0.00001 %

4. Table S1. Antioxidant activity by DPPH, ABTS and superoxide radical scavenging assays

Compound	DPPH		ABTS		$O_2^{-\cdot}$ (superoxide radical)	
	Mean	Stdev.	Mean	Stdev.	Mean	Stdev.
β-carotene	0.6167	0.01528	0.9459	0.0263	0.3199	0.0128
**1**	0.2352	0.01172	0.1245	0.0067	0.3572	0.01106
**2**	0.4779	0.01474	0.5332	0.0077	0.4561	0.02266
**3**	0.5596	0.01249	0.8661	0.0306	0.6353	0.01373
**1a-al**	0.1030	0.00636	0.0868	0.0061	0.3130	0.01674
**2a-al**	0.1516	0.01031	0.1416	0.0072	0.4234	0.0163
**1a**	0.0689	0.00476	0.2333	0.0158	0.2019	0.01176
**2a**	0.3178	0.00609	0.2565	0.0169	0.4075	0.01608
**3a**	0.4117	0.01438	0.5884	0.0242	0.4683	0.01168
**2b**	0.4855	0.02317	0.1537	0.014	0.4122	0.01255
**3b**	0.5564	0.01378	0.7838	0.0309	0.5814	0.01575
**2c**	0.6490	0.01462	0.9094	0.0239	0.4403	0.01764
**2d**	0.5463	0.01488	0.5960	0.0197	0.4843	0.01179
**2e**	0.5596	0.01887	0.7243	0.0205	0.4384	0.01635
**2f**	0.5702	0.02054	0.3459	0.0269	0.4121	0.02737
**2g**	0.5068	0.01252	0.2991	0.0145	0.3556	0.0088
**2h**	0.5252	0.01273	0.3096	0.0096	0.3589	0.01409
**2i**	0.9539	0.01155	0.6592	0.0271	0.3353	0.01943
**2j**	0.2327	0.00685	0.3268	0.0130	0.4287	0.01075
canthaxanthin	0.4179	0.01158	0.6465	0.0278	0.2907	0.01073
