# Supplementary material for: Oxo-Carotenoids as Efficient Superoxide Radical Scavengers
Source: Antioxidants (Basel). 2022 Aug 5;11(8):1525. doi: 10.3390/antiox11081525 (PMC9405038; doi:10.3390/antiox11081525)
Supplement: Supplementary file 1 [file antioxidants-11-01525-s001.zip › Supplementary Materials-2.pdf]

Supplementary Materials-2 for

Oxo-carotenoids as Efficient Superoxide Radical Scavengers

Gaosheng Shi,[a] Hyein Kim[b] and Sangho Koo[a,b]*

[a] Department of Energy Science and Technology, [b] Department of Chemistry, Myongji University, Myongji-Ro 116, Yongin, 17058, Gyeonggi-Do, Korea

Table of Contents

(1) ^{1}H- and ^{13}C-NMR Spectra -- S-2

(2) Assignment of *E*-stereochemistry for **4** by N.O.E. -- S-59

(3) Peak assignment for **2b** by COSY, HMBC, and DEPT --- S-63

(1) ^{1}H- and ^{13}C-NMR Spectra

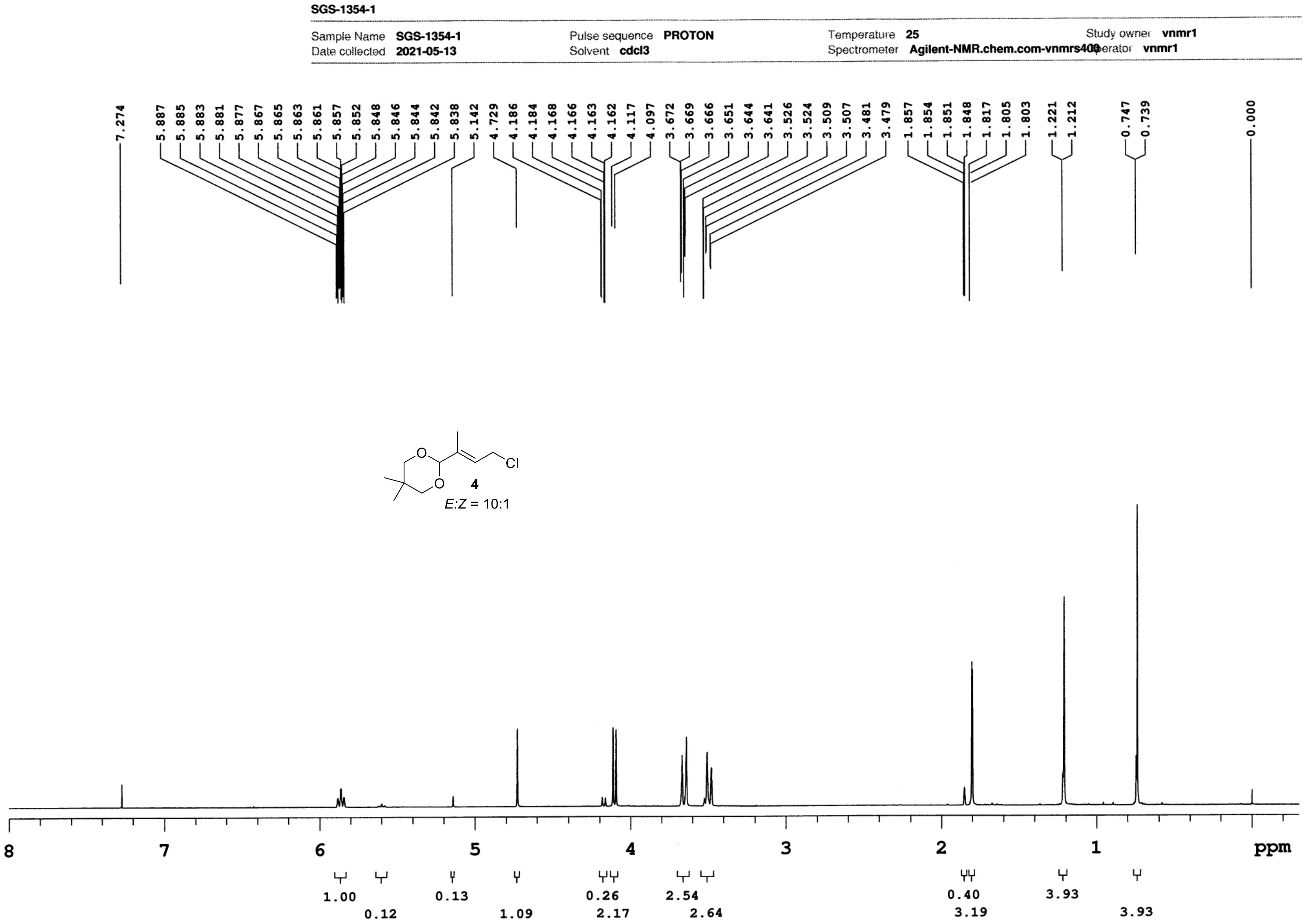

SGS-1354-1-13C

Sample Name	**SGS-1354-1-13C**	Pulse sequence	**CARBON**	Temperature	**25**	Study owner	**vnmr1**
Date collected	**2021-05-17**	Solvent	**cdcl3**	Spectrometer	**Agilent-NMR.chem.com-vnmrs400**	Operator	**vnmr1**

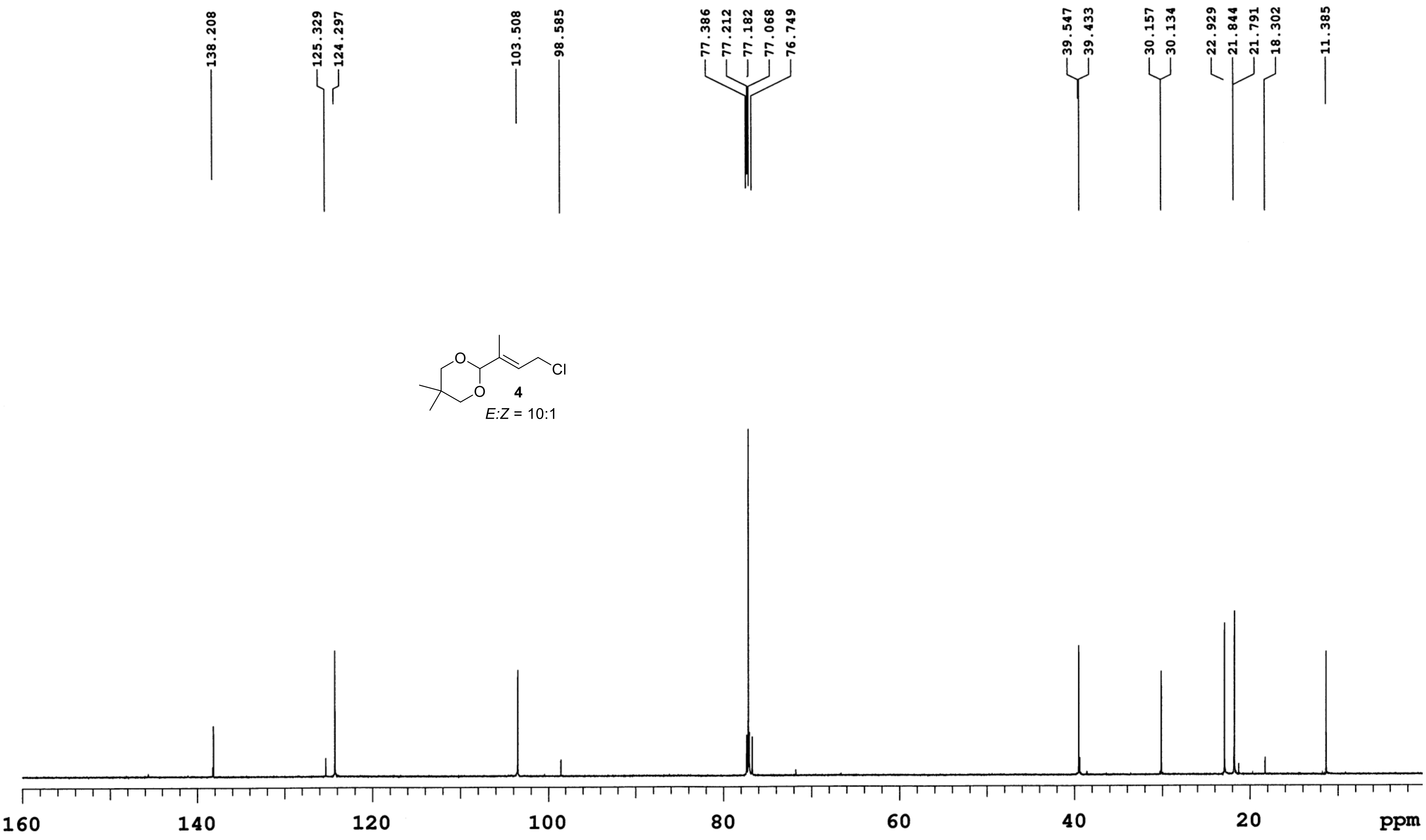

SGS-1540-11

Sample Name	**SGS-1540-11**	Pulse sequence	**PROTON**	Temperature	**25**	Study owner	**vnmr1**
Date collected	**2021-11-09**	Solvent	**cdcl3**	Spectrometer	**Agilent-NMR.chem.com-vnmrs400**	Operator	**vnmr1**

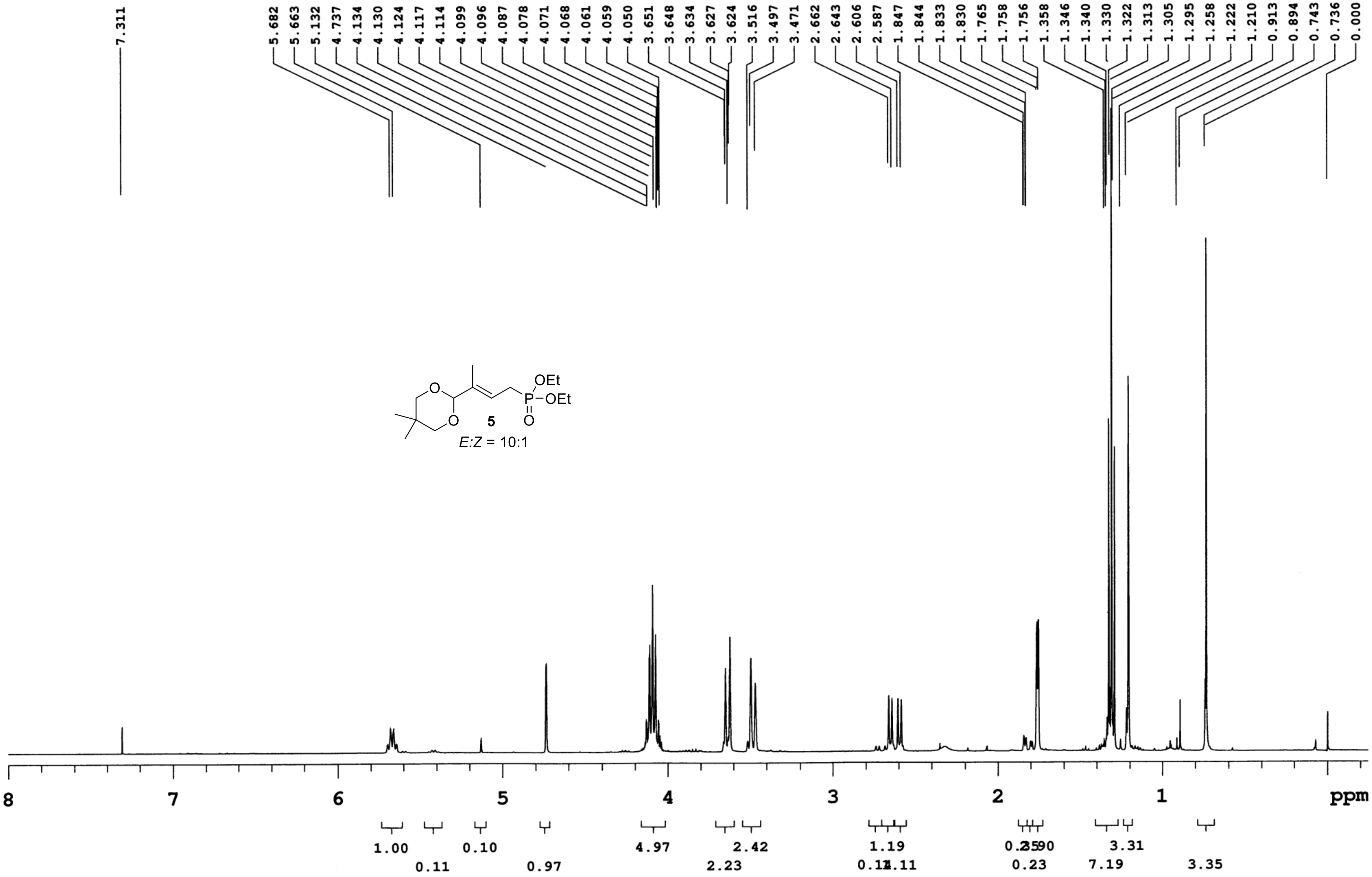

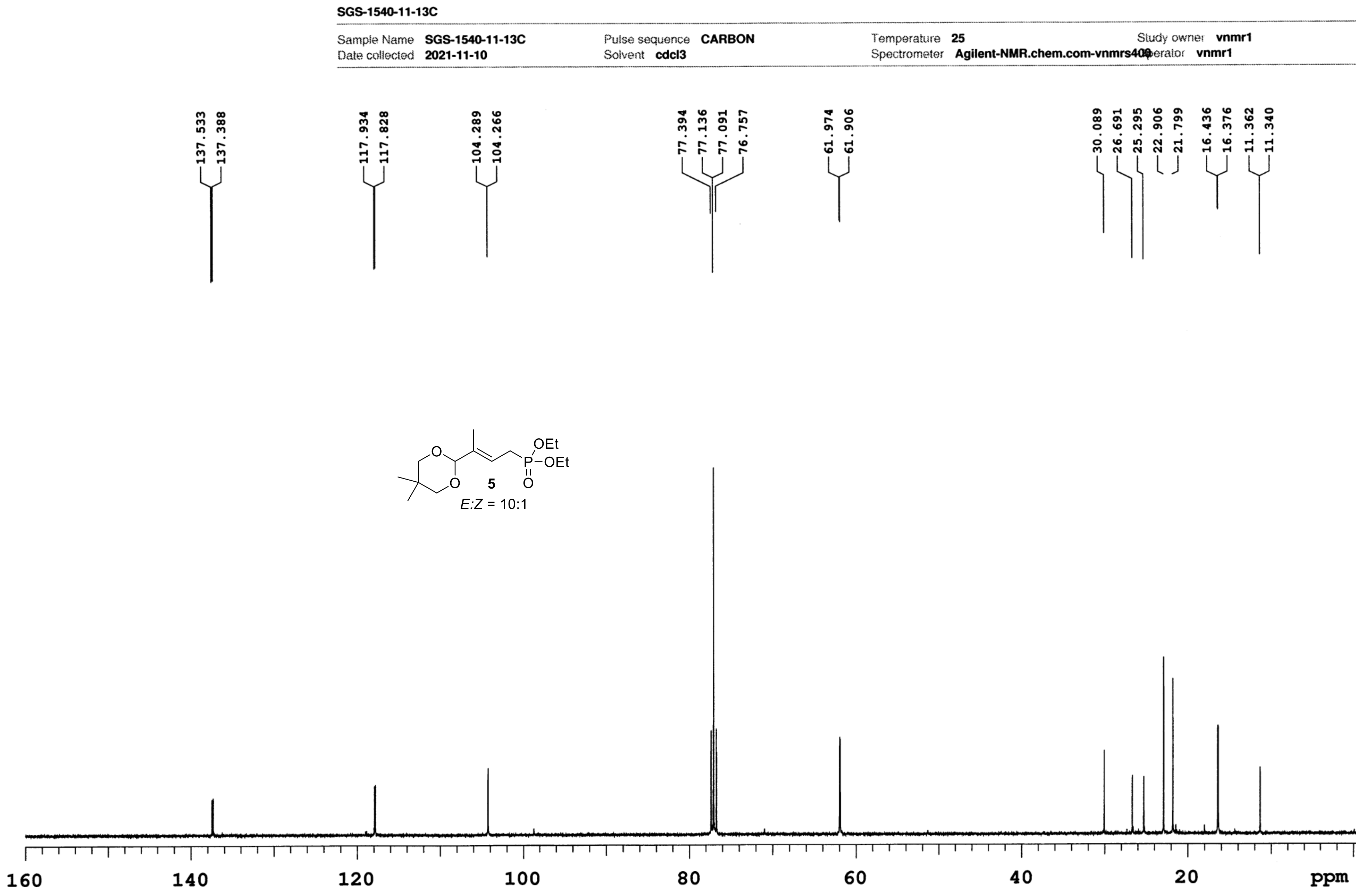
SGS-1540-11-13C
Sample Name SGS-1540-11-13C
Date collected 2021-11-10
Pulse sequence CARBON
Solvent cdcl3
Temperature 25
Spectrometer Agilent-NMR.chem.com-vnmrs400
Study owner vnmr1
Operator vnmr1
137.533
137.388
117.934
117.828
104.289
104.266
77.394
77.136
77.091
76.757
61.974
61.906
30.089
26.691
25.295
22.906
21.799
16.436
16.376
11.362
11.340
OEt
P
OEt
O
O
O
5
E:Z = 10:1
160
140
120
100
80
60
40
20
ppm

SGS-1621-2

Sample Name **SGS-1621-2**	Pulse sequence **PROTON**	Temperature **25**	Study owner **vnmr1**
Date collected **2021-11-10**	Solvent **cdcl3**	Spectrometer **Agilent-NMR.chem.com-vnmrs400**	Operator **vnmr1**

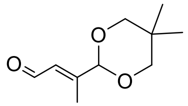

6

E:Z > 5:1

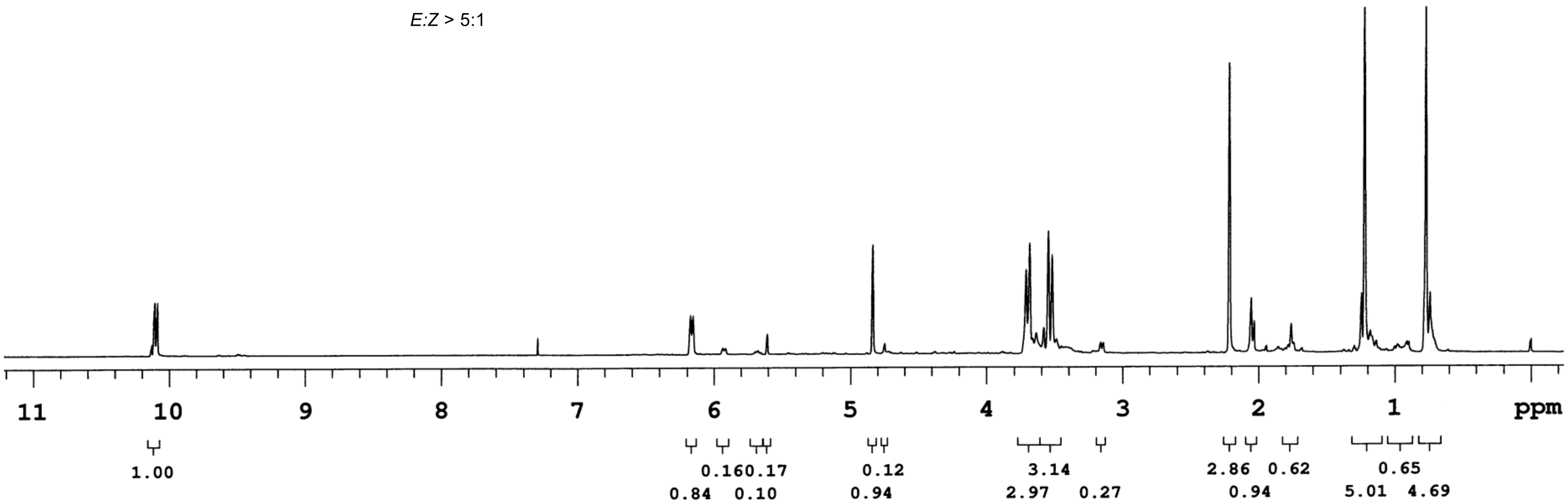

SGS-1621-2

Sample Name	SGS-1621-2	Pulse sequence	CARBON	Temperature	25	Study owner	vnmr1
Date collected	2021-11-12	Solvent	cdcl3	Spectrometer	Agilent-NMR.chem.com-vnmrs400	Operator	vnmr1

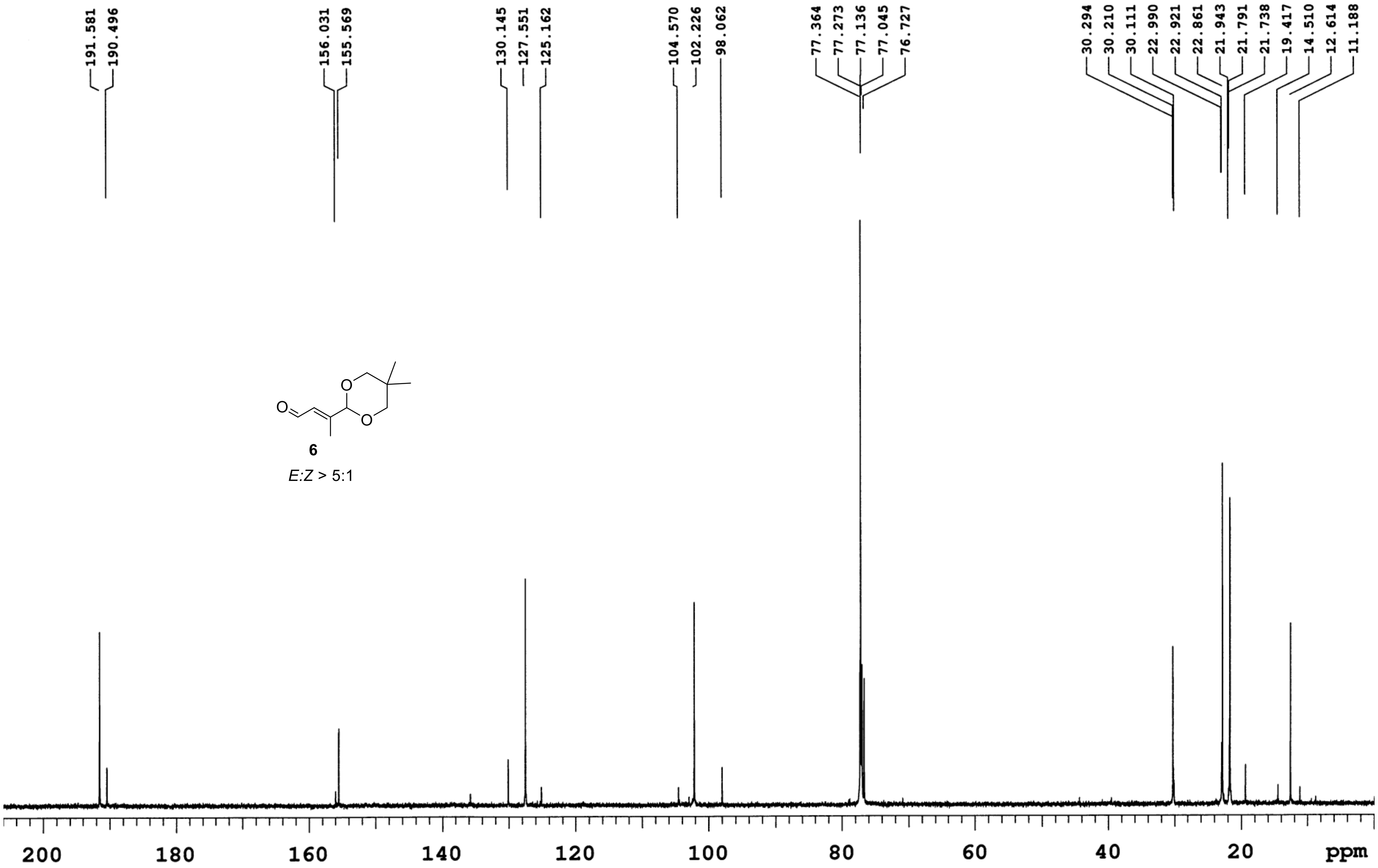

SGS-1541-21re

Sample Name	**SGS-1541-21re**	Pulse sequence	**PROTON**	Temperature	**25**	Study owner	**vnmr1**
Date collected	**2021-10-12**	Solvent	**cdcl3**	Spectrometer	**Agilent-NMR.chem.com-vnmrs400**	Operator	**vnmr1**

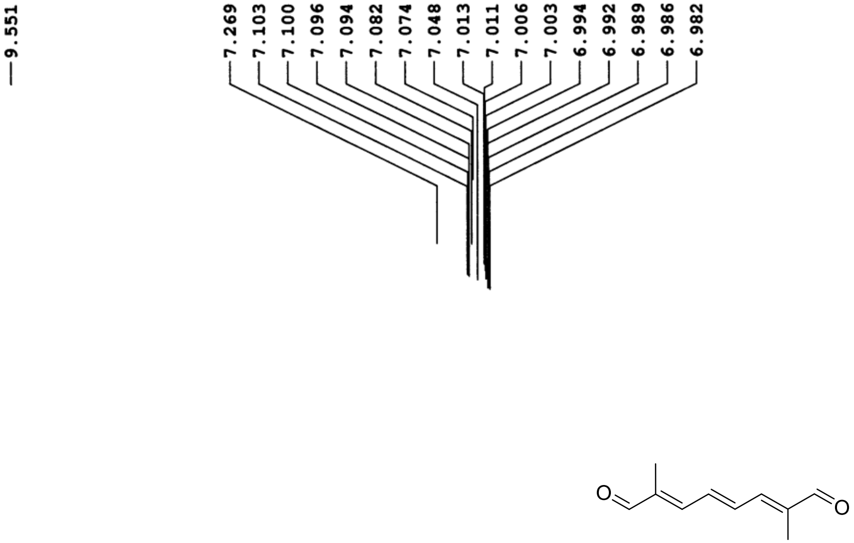

1

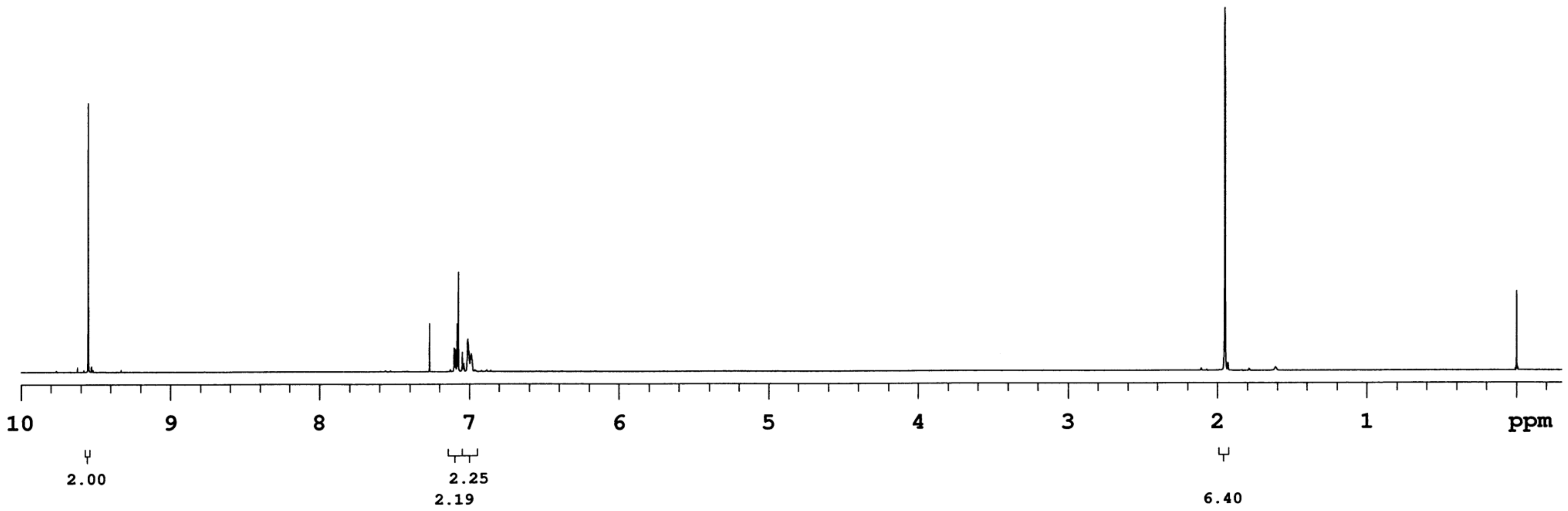

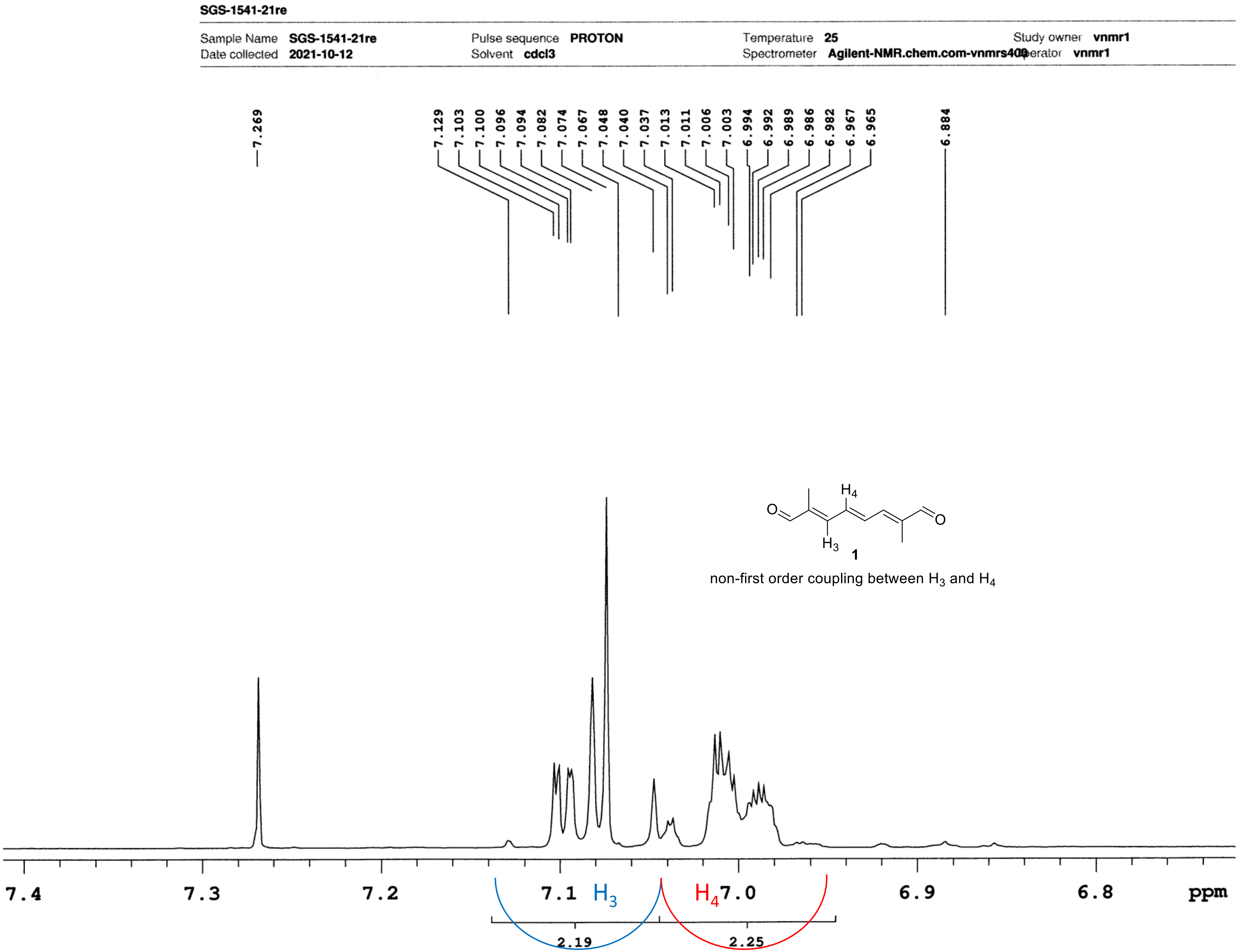
SGS-1541-21re
Sample Name SGS-1541-21re
Date collected 2021-10-12
Pulse sequence PROTON
Solvent cdcl3
Temperature 25
Spectrometer Agilent-NMR.chem.com-vnmrs400
Study owner vnmr1
Operator vnmr1
7.269
7.129 7.103 7.100 7.096 7.094 7.082 7.074 7.067 7.048 7.040 7.037 7.013 7.011 7.006 7.003 6.994 6.992 6.989 6.986 6.982 6.967 6.965
6.884
H4
O
O
H3
1
non-first order coupling between H3 and H4
7.4 7.3 7.2 7.1 7.0 6.9 6.8 ppm
H3
H4
2.19
2.25

SGS-1412-C20

Sample Name	**SGS-1412-C20**	Pulse sequence	**PROTON**	Temperature	**25**	Study owner	**vnmr1**
Date collected	**2021-11-16**	Solvent	**cdcl3**	Spectrometer	**Agilent-NMR.chem.com-vnmrs400**	Operator	**vnmr1**

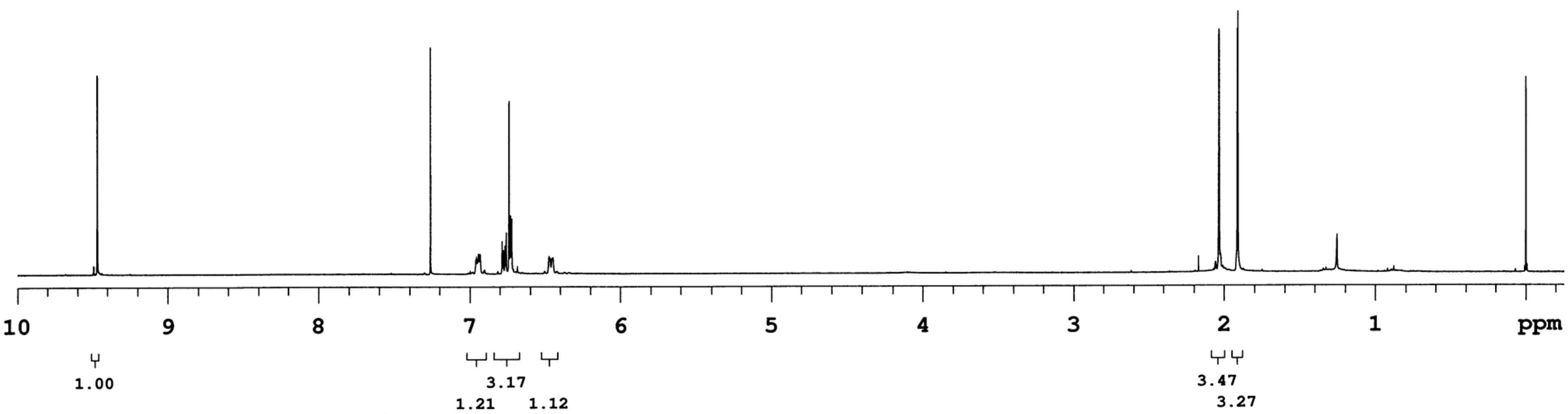

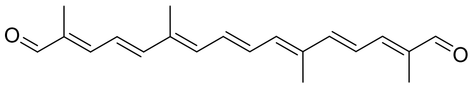

2

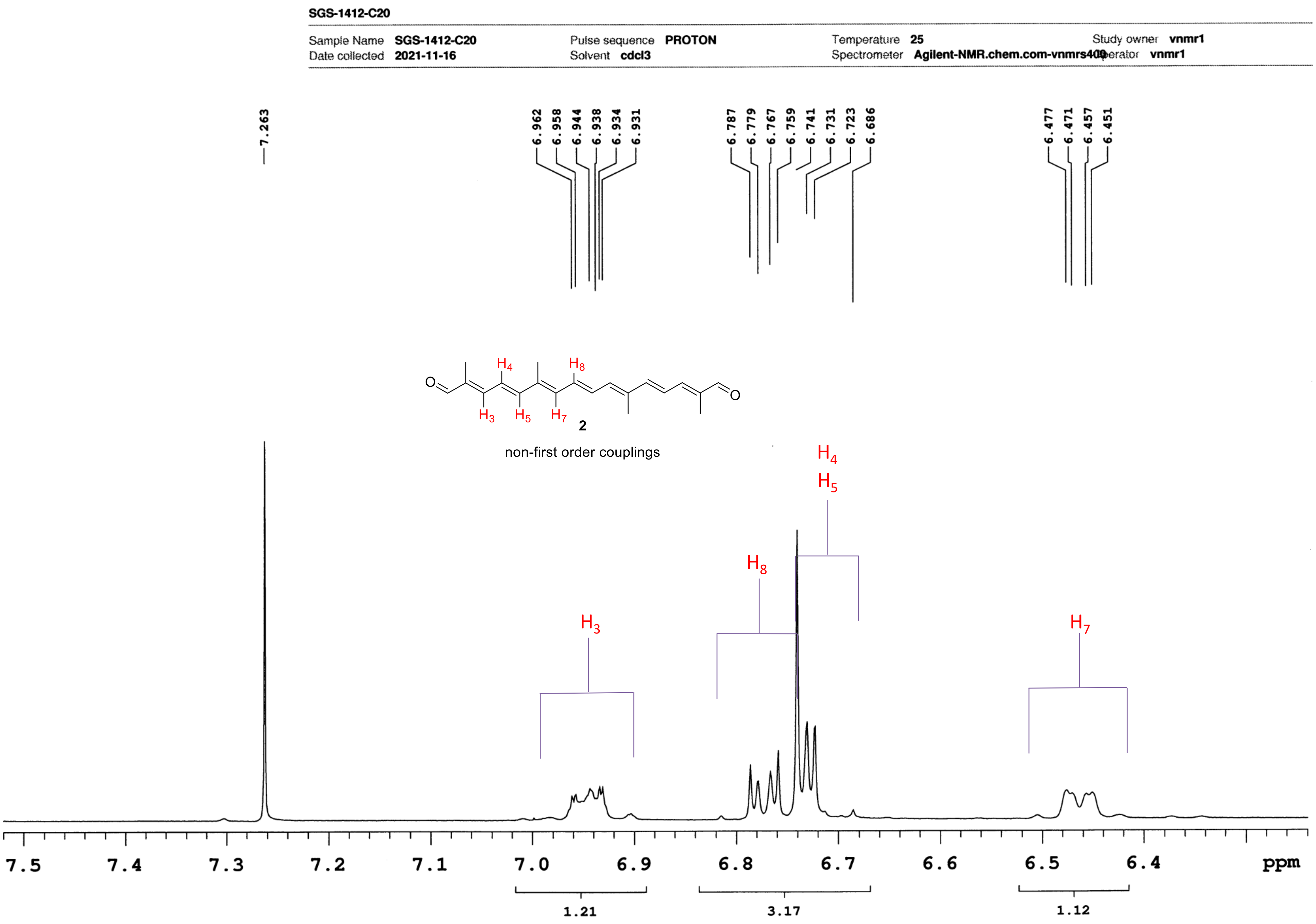
SGS-1412-C20
Sample Name SGS-1412-C20
Date collected 2021-11-16
Pulse sequence PROTON
Solvent cdcl3
Temperature 25
Spectrometer Agilent-NMR.chem.com-vnmrs400
Study owner vnmr1
Operator vnmr1
7.263
6.962 6.958 6.944 6.938 6.934 6.931
6.787 6.779 6.767 6.759 6.741 6.731 6.723 6.686
6.477 6.471 6.457 6.451
H4 H8 H3 H5 H7
2
non-first order couplings
H4
H5
H8
H3
H7
7.5 7.4 7.3 7.2 7.1 7.0 6.9 6.8 6.7 6.6 6.5 6.4 ppm
1.21
3.17
1.12

SGS-1412-C20-13C

Sample Name **SGS-1412-C20-13C**
Date collected **2021-11-17**
Pulse sequence **CARBON**
Solvent **cdcl3**
Temperature **25**
Spectrometer **Agilent-NMR.chem.com-vnmrs400**
Study owner **vnmr1**
Operator **vnmr1**

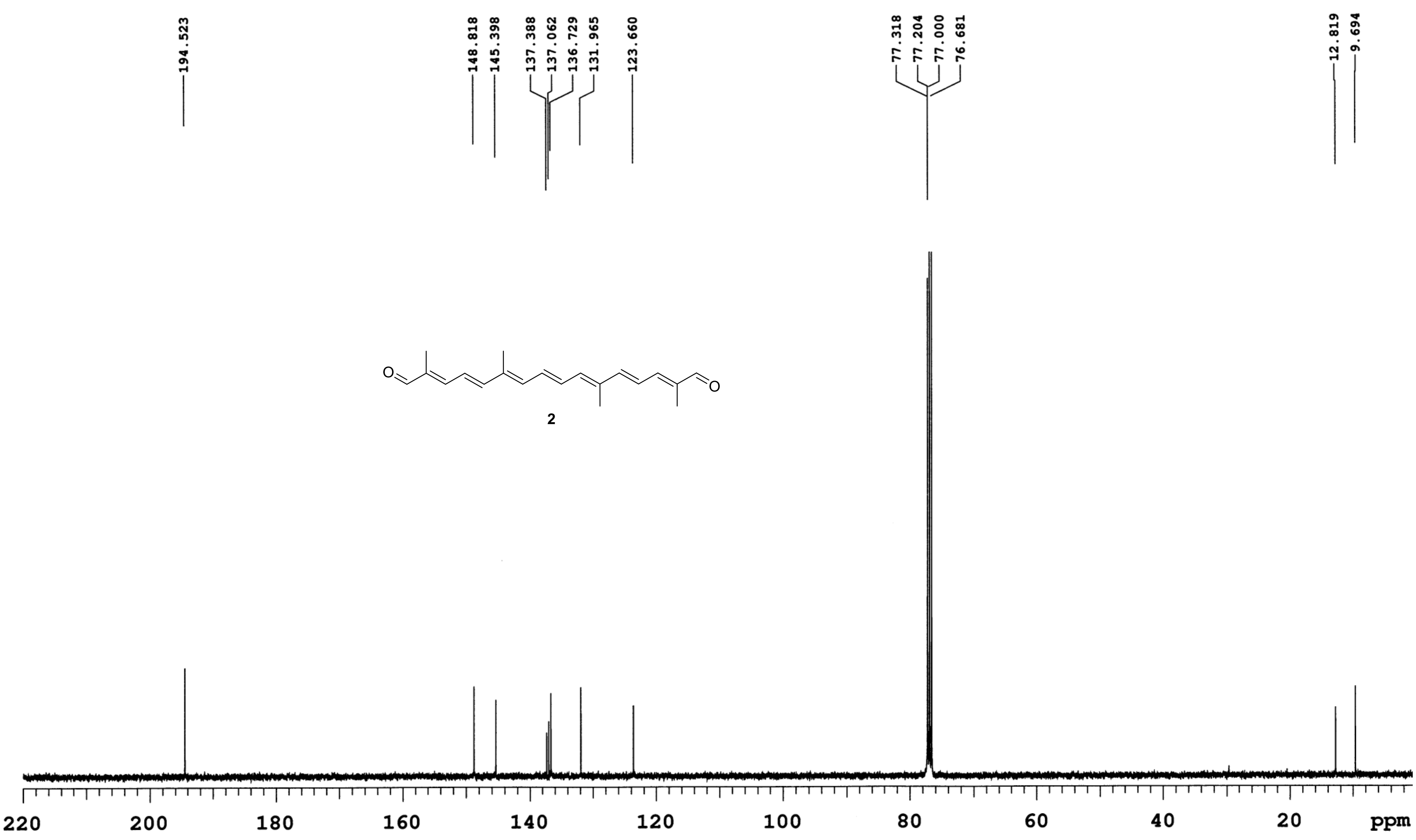

SGS-1492-23re

Sample Name	**SGS-1492-23re**	Pulse sequence	**PROTON**	Temperature	**25**	Study owner	**vnmr1**
Date collected	**2021-07-05**	Solvent	**cdcl3**	Spectrometer	**Agilent-NMR.chem.com-vnmrs400**	Operator	**vnmr1**

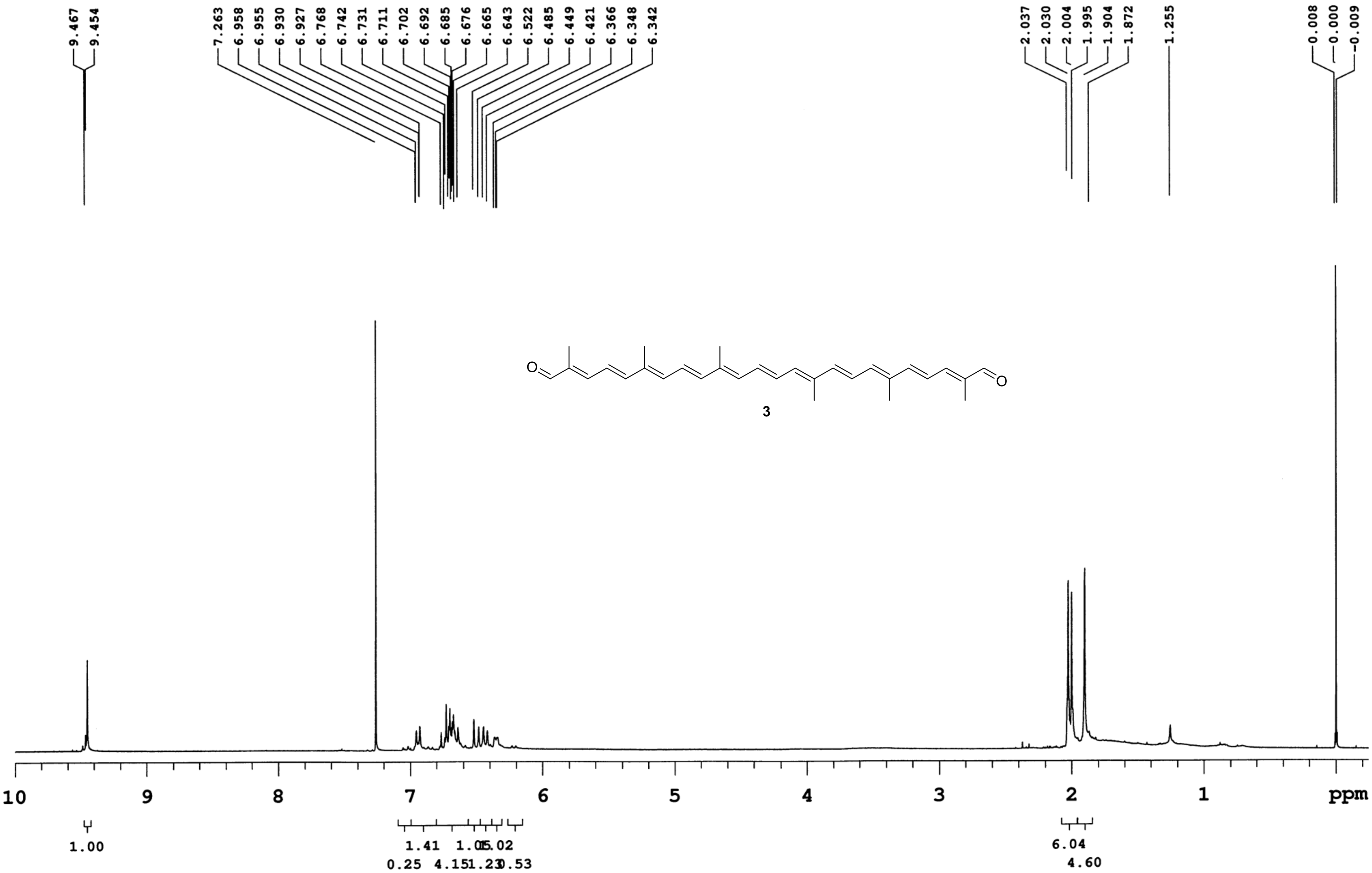

SGS-1492-23re

Sample Name	SGS-1492-23re	Pulse sequence	PROTON	Temperature	25	Study owner	vnmr1
Date collected	2021-07-05	Solvent	cdcl3	Spectrometer	Agilent-NMR.chem.com-vnmrs400	Operator	vnmr1

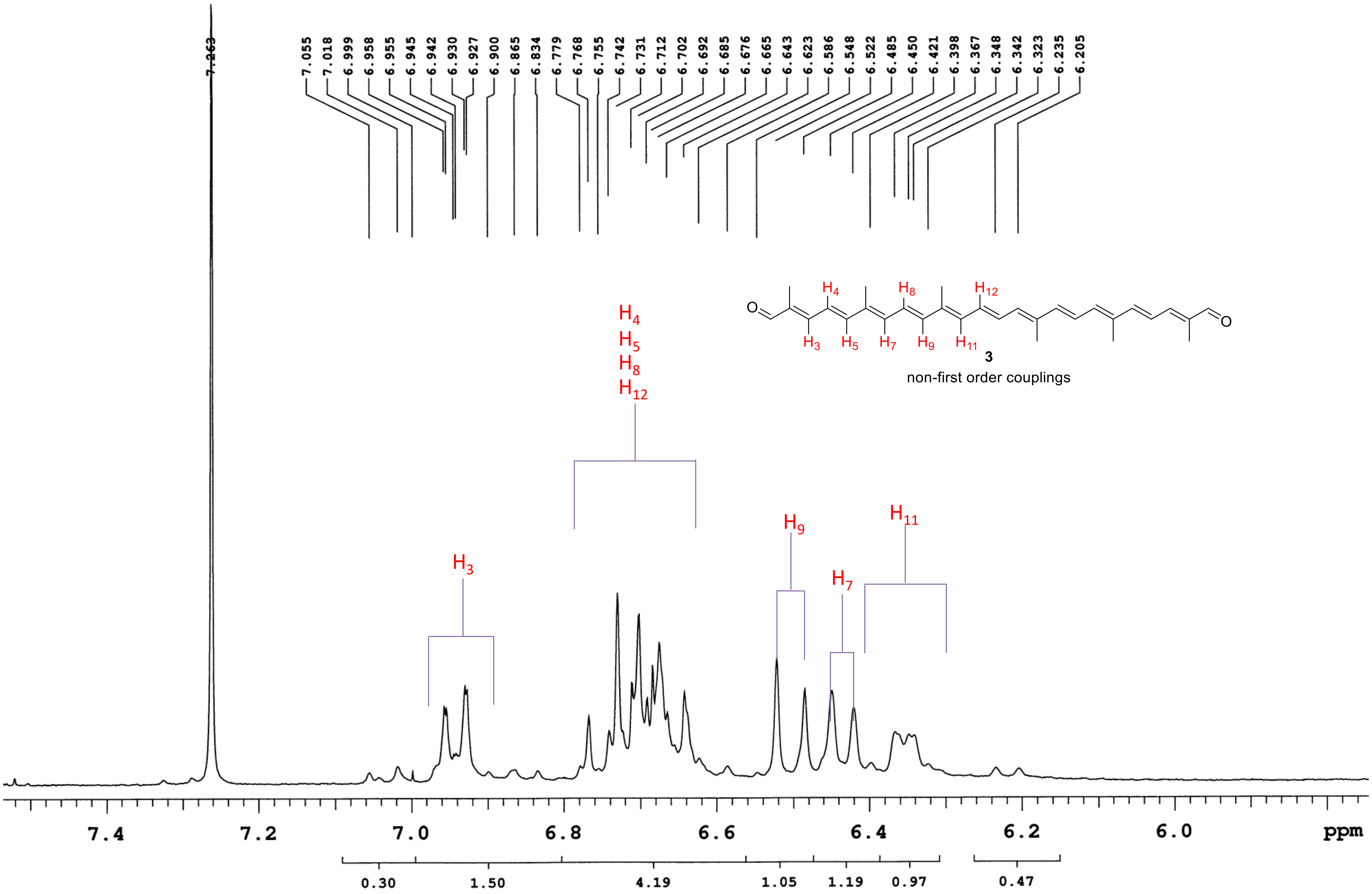

SGS-1492-23re

Sample Name **SGS-1492-23re** | Pulse sequence **CARBON** | Temperature **25** | Study owner **vnmr1**
Date collected **2021-07-02** | Solvent **cdcl3** | Spectrometer **Agilent-NMR.chem.com-vnmrs400** | Operator **vnmr1**

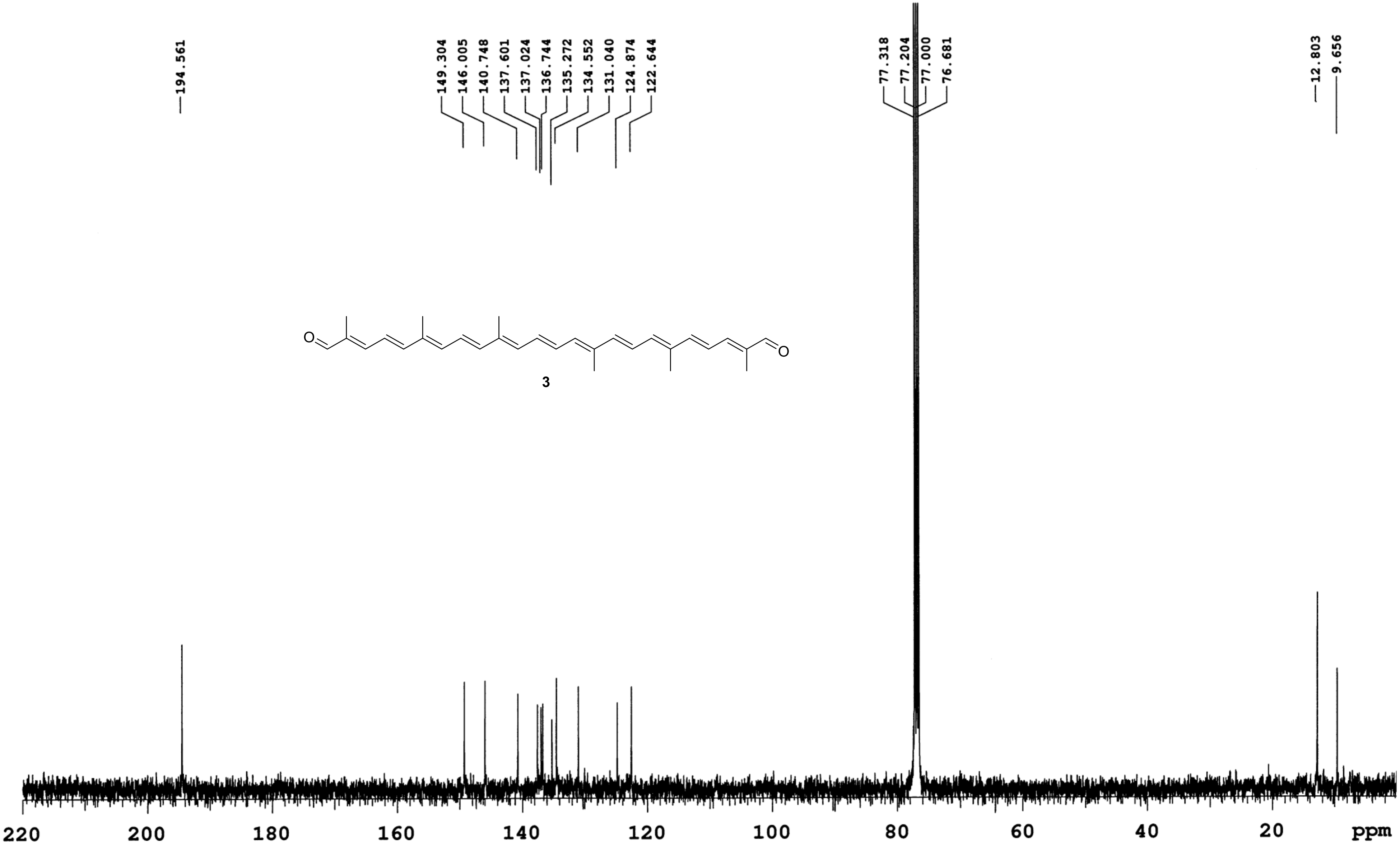

SGS-1332-1re

Sample Name SGS-1332-1re
Date collected 2021-06-02
Pulse sequence PROTON
Solvent cdcl3
Temperature 25
Spectrometer Agilent-NMR.chem.com-vnmrs400
Study owner vnmr1
Operator vnmr1

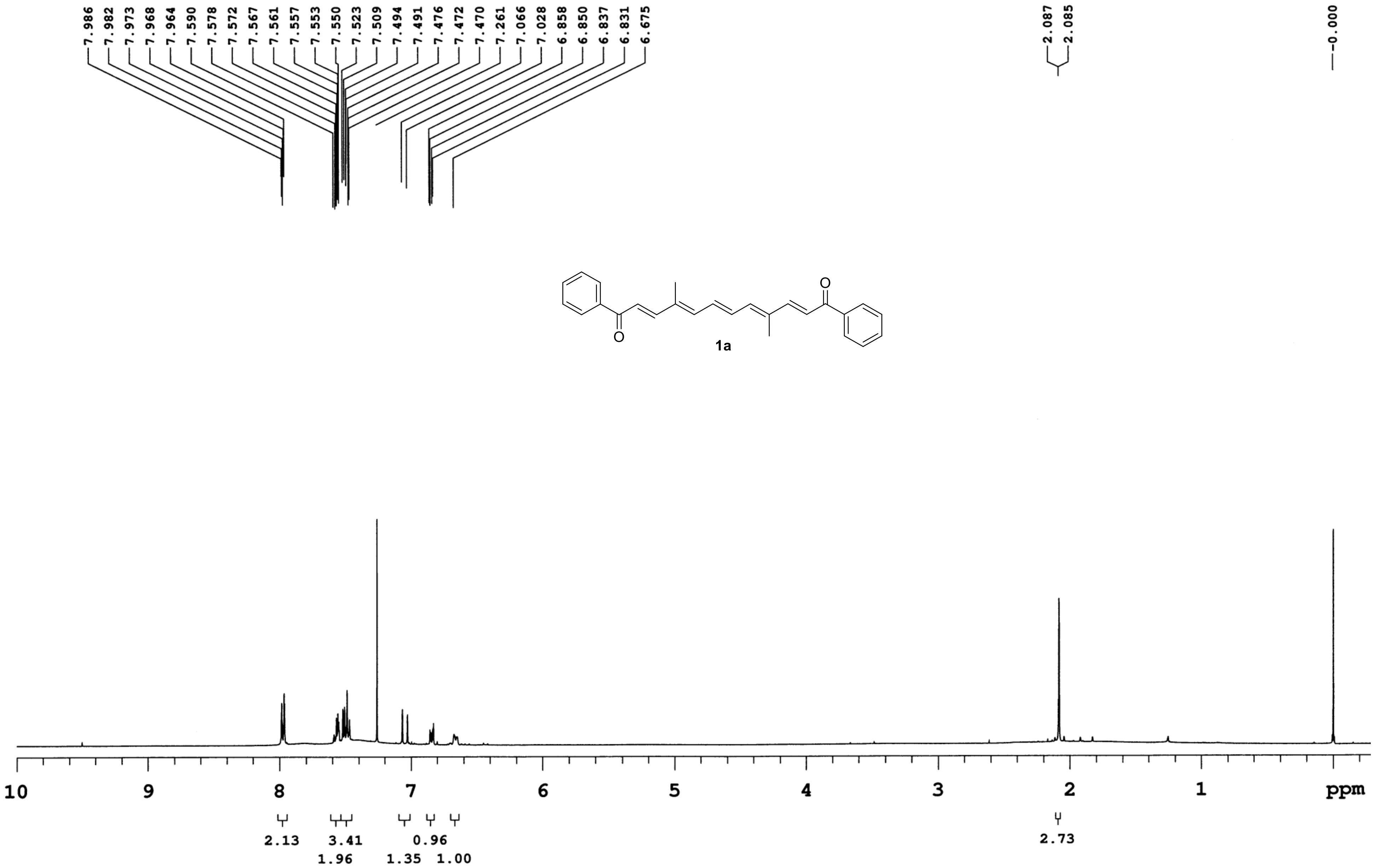

1a

SGS-1332-1re

Sample Name SGS-1332-1re Pulse sequence PROTON Temperature 25 Study owner vnmr1
Date collected 2021-06-02 Solvent cdcl3 Spectrometer Agilent-NMR.chem.com-vnmrs400 Operator vnmr1

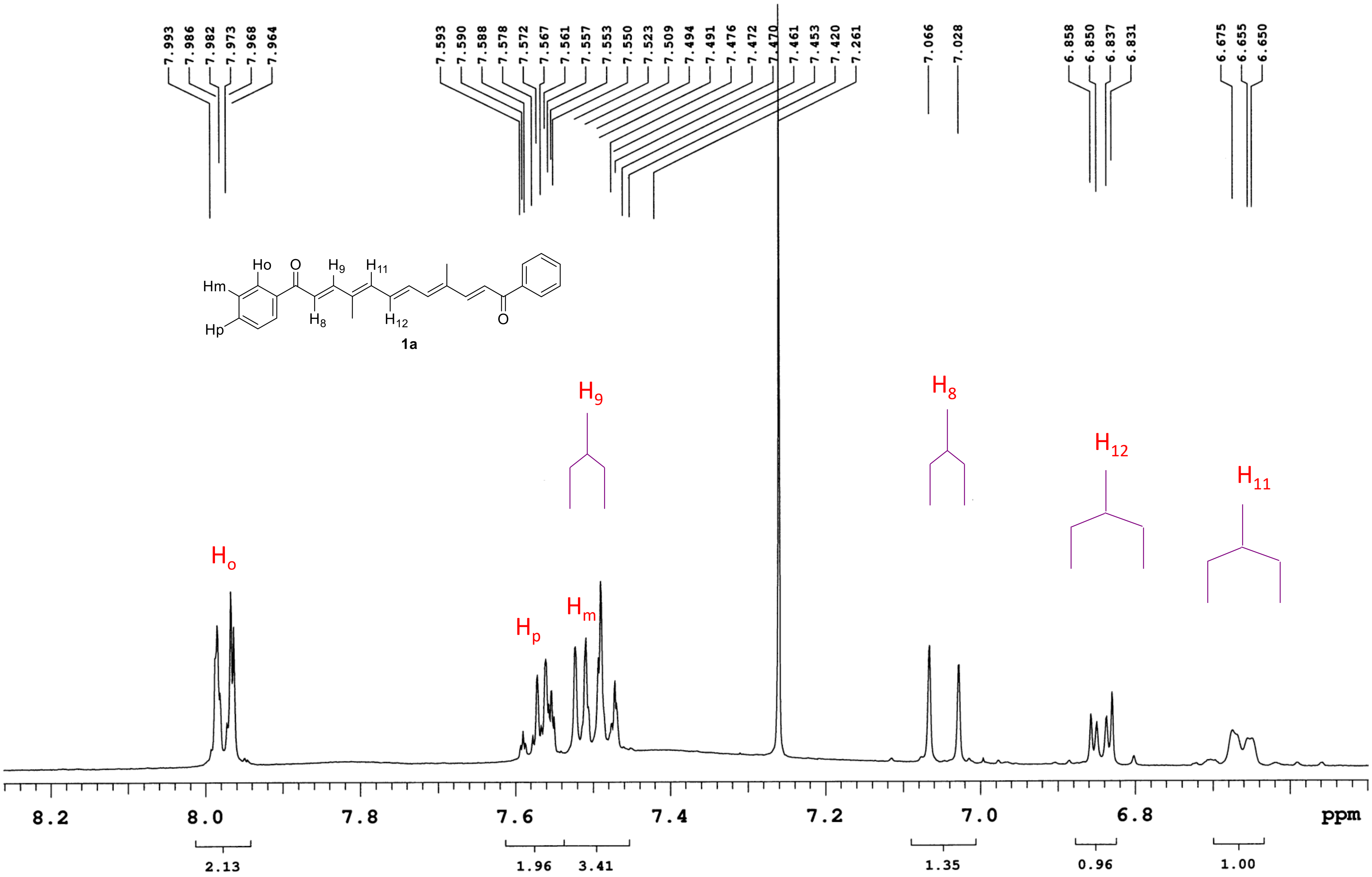

SGS-1396-2re

Sample Name **SGS-1396-2re** Date collected **2021-06-16** Pulse sequence **PROTON** Solvent **cdcl3** Temperature **25** Spectrometer **Agilent-NMR.chem.com-vnmrs400** Study owner **vnmr1** Operator **vnmr1**

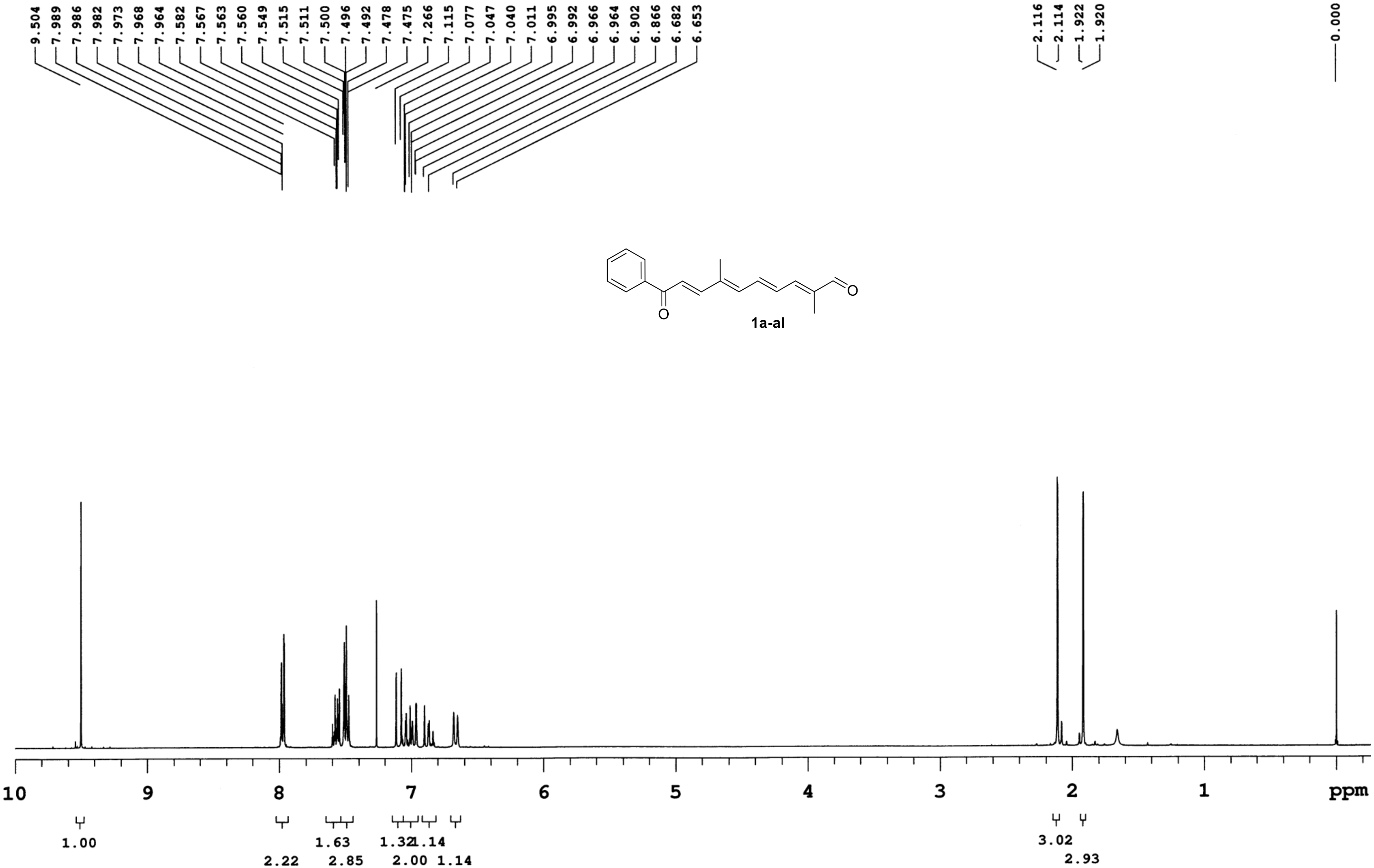

1a-al

SGS-1396-2re

Sample Name SGS-1396-2re
Date collected 2021-06-16
Pulse sequence PROTON
Solvent cdcl3
Temperature 25
Spectrometer Agilent-NMR.chem.com-vnmrs400
Study owner vnmr1
Operator vnmr1

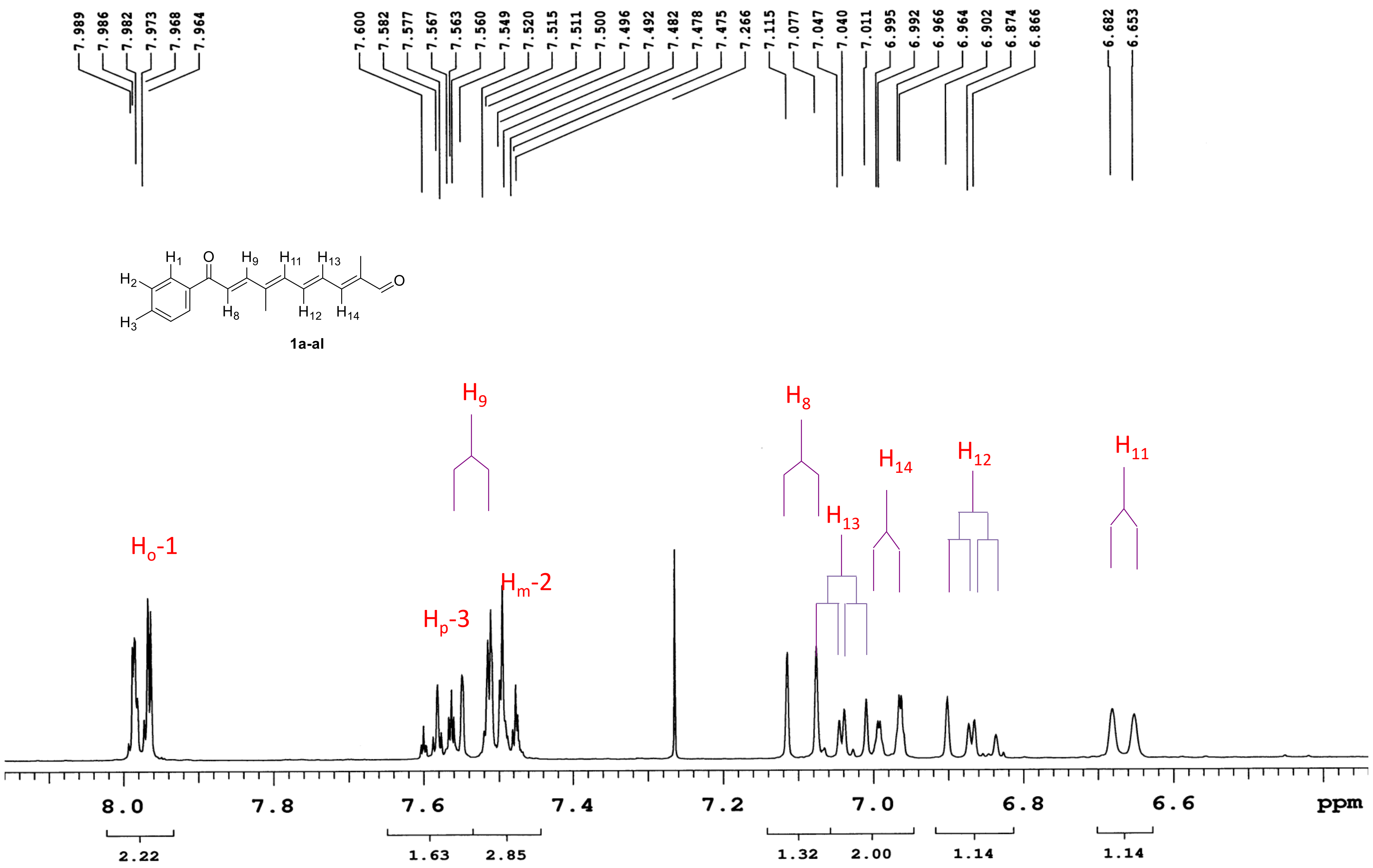

SGS-1396-2re-13C

Sample Name **SGS-1396-2re-13C** Pulse sequence **CARBON** Temperature **25** Study owner **vnmr1**
Date collected **2021-06-18** Solvent **cdcl3** Spectrometer **Agilent-NMR.chem.com-vnmrs400** Operator **vnmr1**

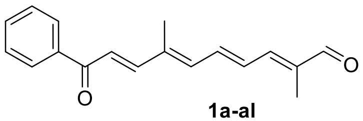

1a-al

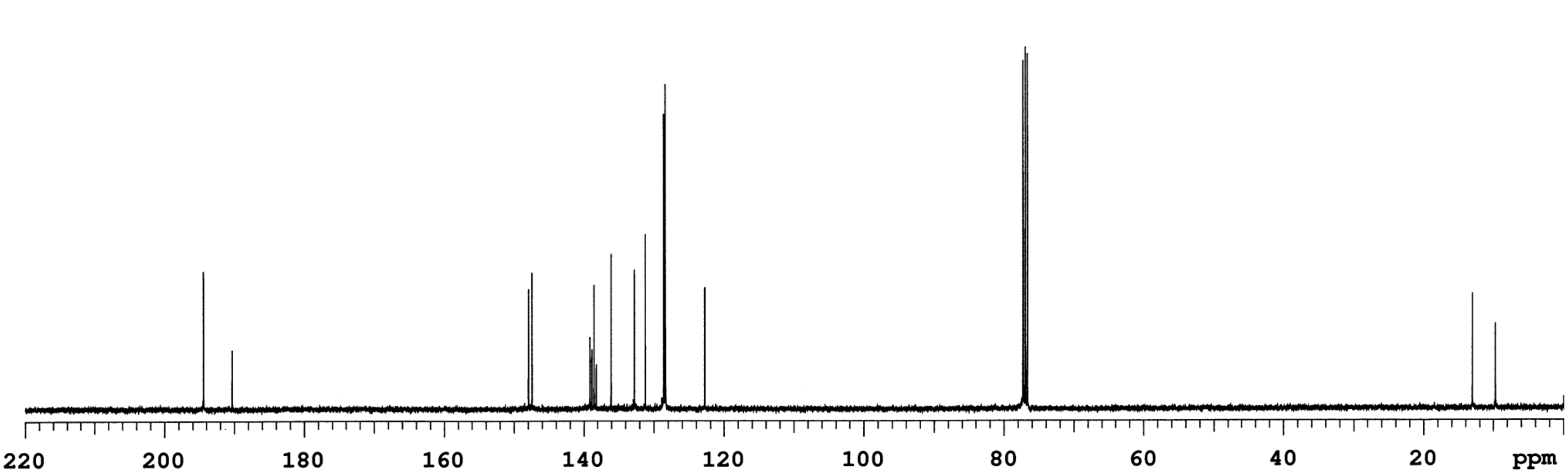

SGS-1420-12re

Sample Name **SGS-1420-12re** Pulse sequence **PROTON** Temperature **25** Study owner **vnmr1**
Date collected **2021-06-14** Solvent **cdcl3** Spectrometer **Agilent-NMR.chem.com-vnmrs400** Operator **vnmr1**

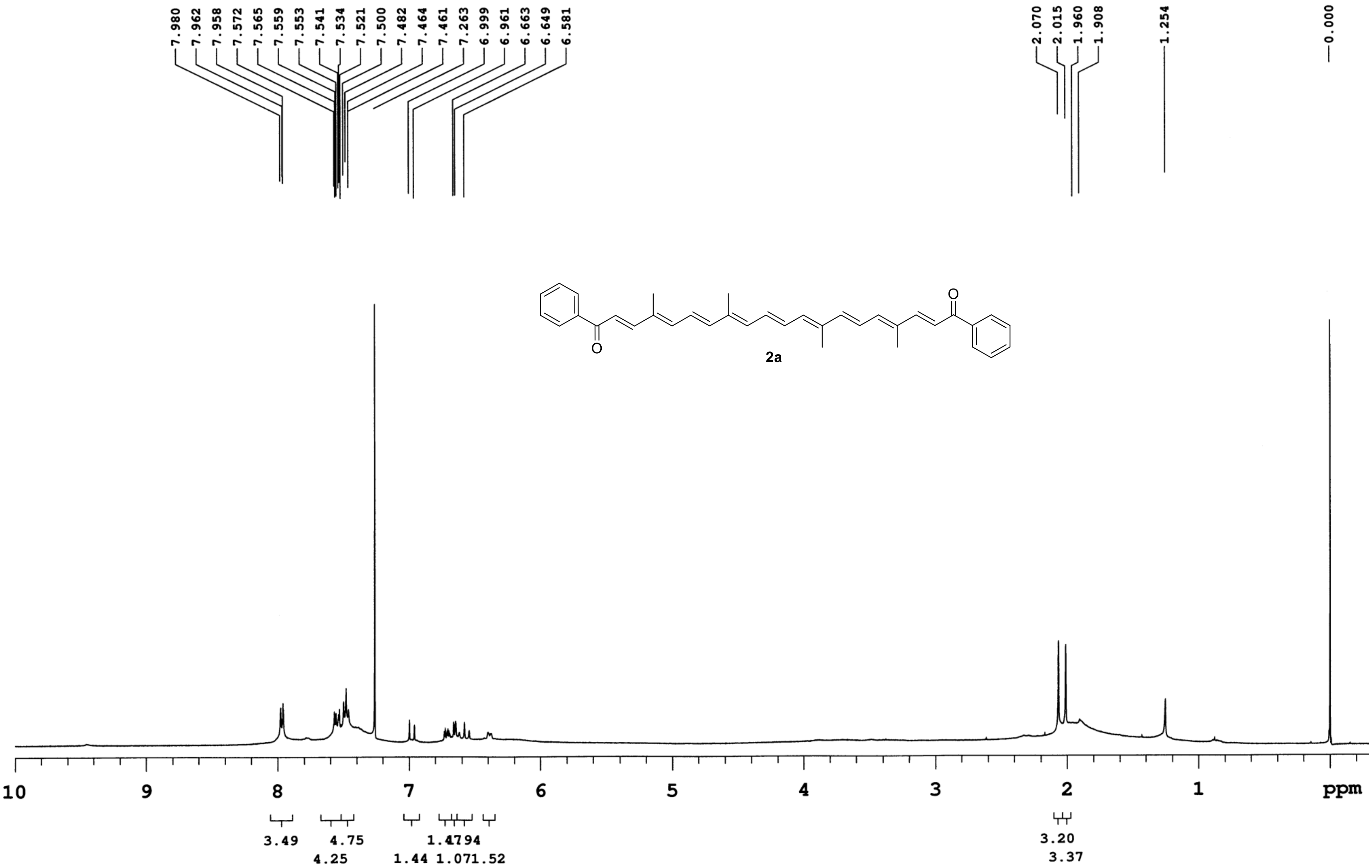

SGS-1420-12re

Sample Name	SGS-1420-12re	Pulse sequence	PROTON	Temperature	25	Study owner	vnmr1
Date collected	2021-06-14	Solvent	cdcl3	Spectrometer	Agilent-NMR.chem.com-vnmrs400	Operator	vnmr1

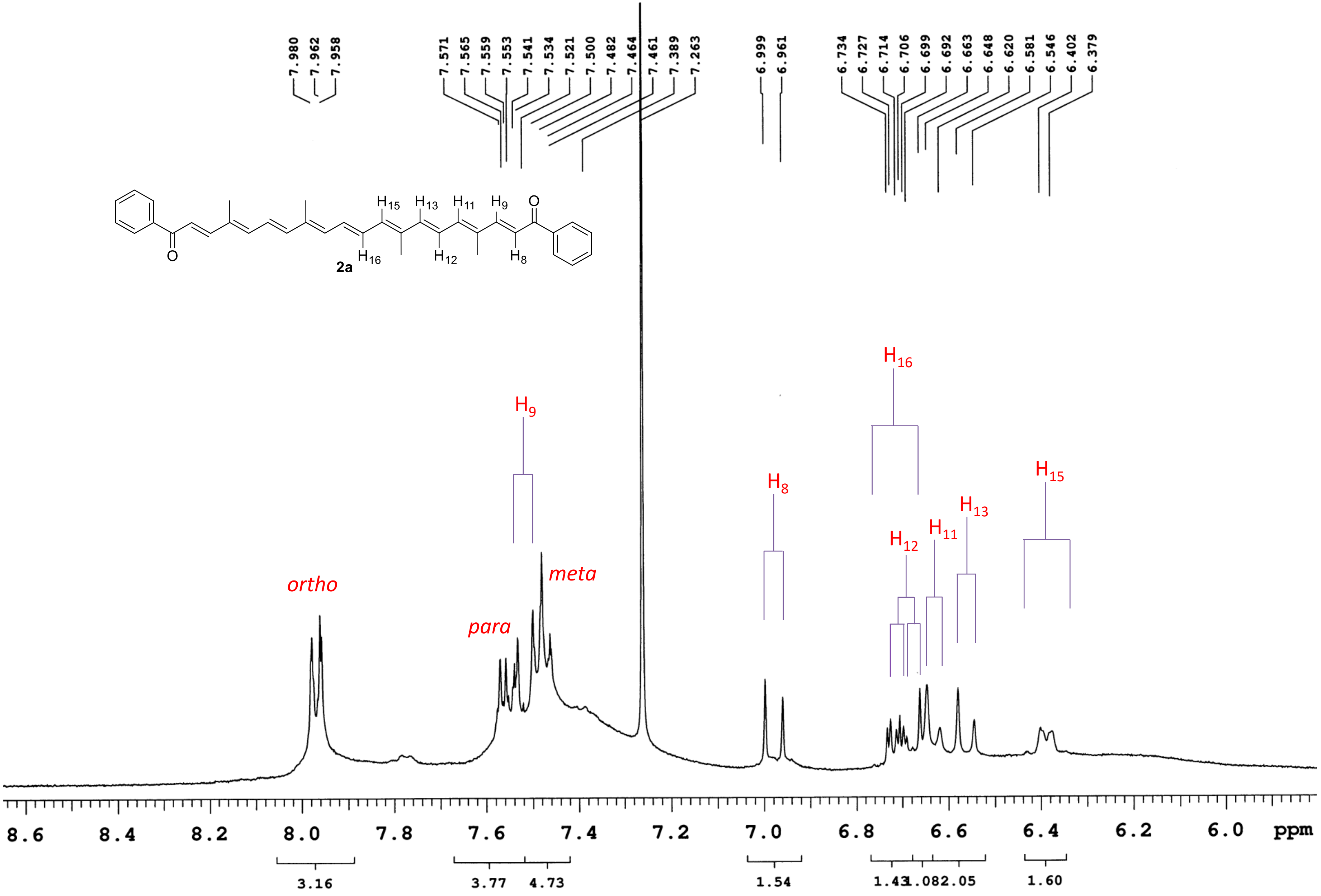

Parameter	Value
Title	SGS-1420-11re-13C_CARBON_01
Instrument	vnmrs
Solvent	cdcl3
Pulse Sequence	s2pul
Probe	5mm_ATB
Number of Scans	2000
Acquisition Date	2021-11-16T17:32:46
Spectrometer Frequency	100.60

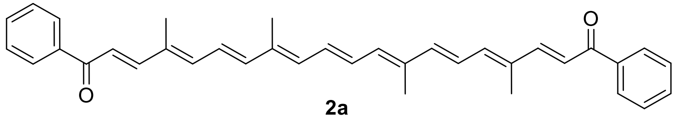

2a

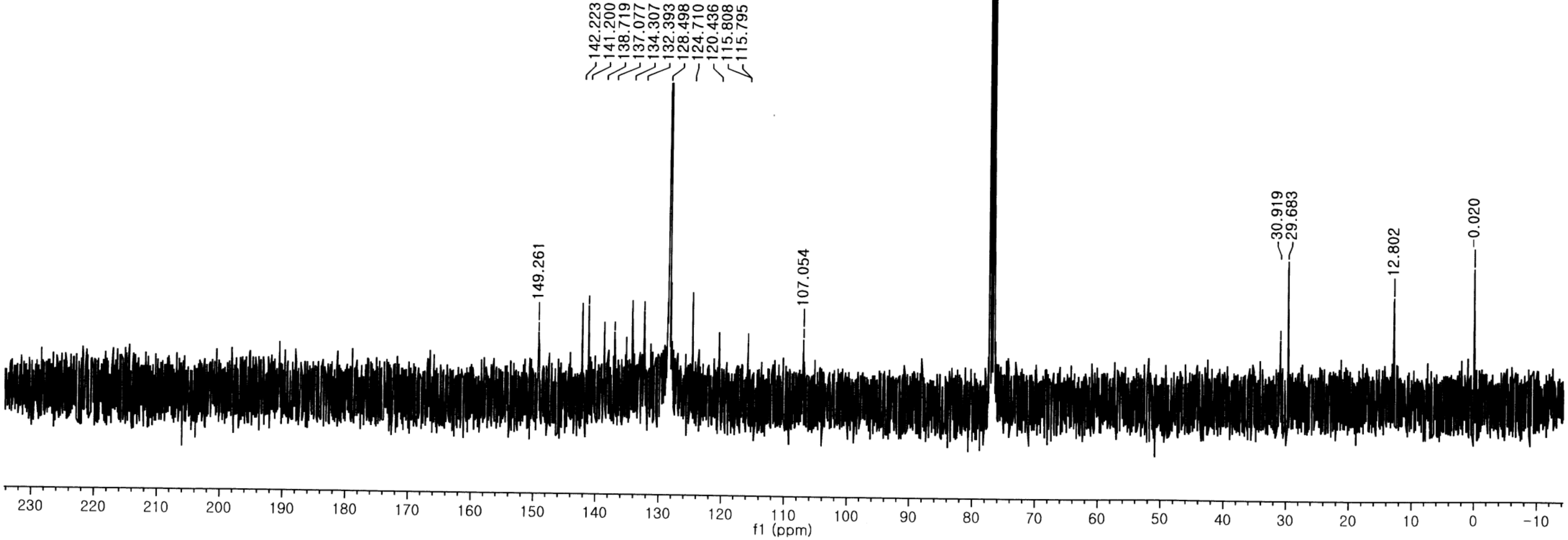

SGS-1421-11re

Sample Name	**SGS-1421-11re**	Pulse sequence	**PROTON**	Temperature	**25**	Study owner	**vnmr1**
Date collected	**2021-11-16**	Solvent	**cdcl3**	Spectrometer	**Agilent-NMR.chem.com-vnmrs400**	Operator	**vnmr1**

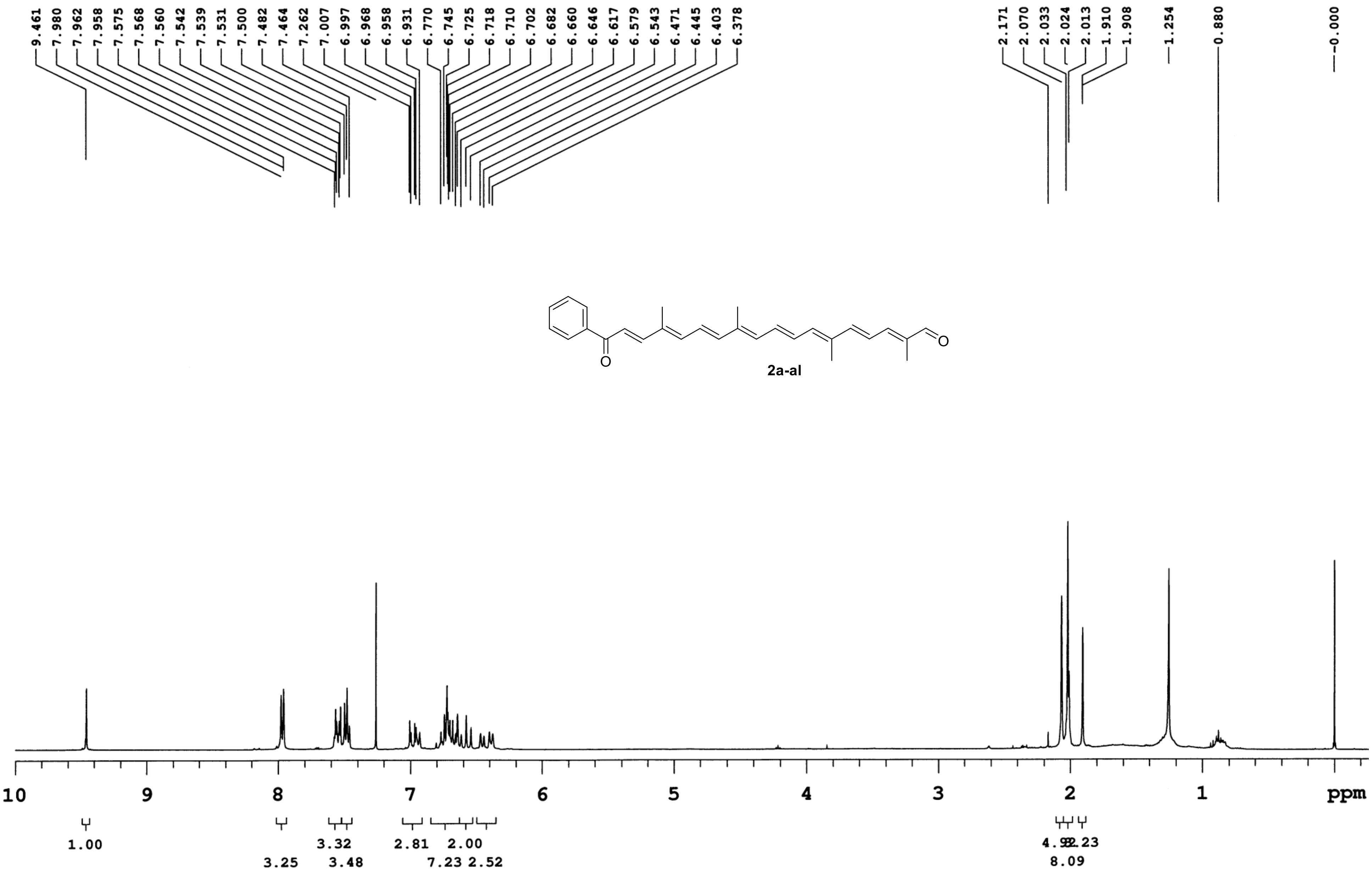

SGS-1421-11re

Sample Name SGS-1421-11re
Date collected 2021-11-16
Pulse sequence PROTON
Solvent cdcl3
Temperature 25
Spectrometer Agilent-NMR.chem.com-vnmrs400
Study owner vnmr1
Operator vnmr1

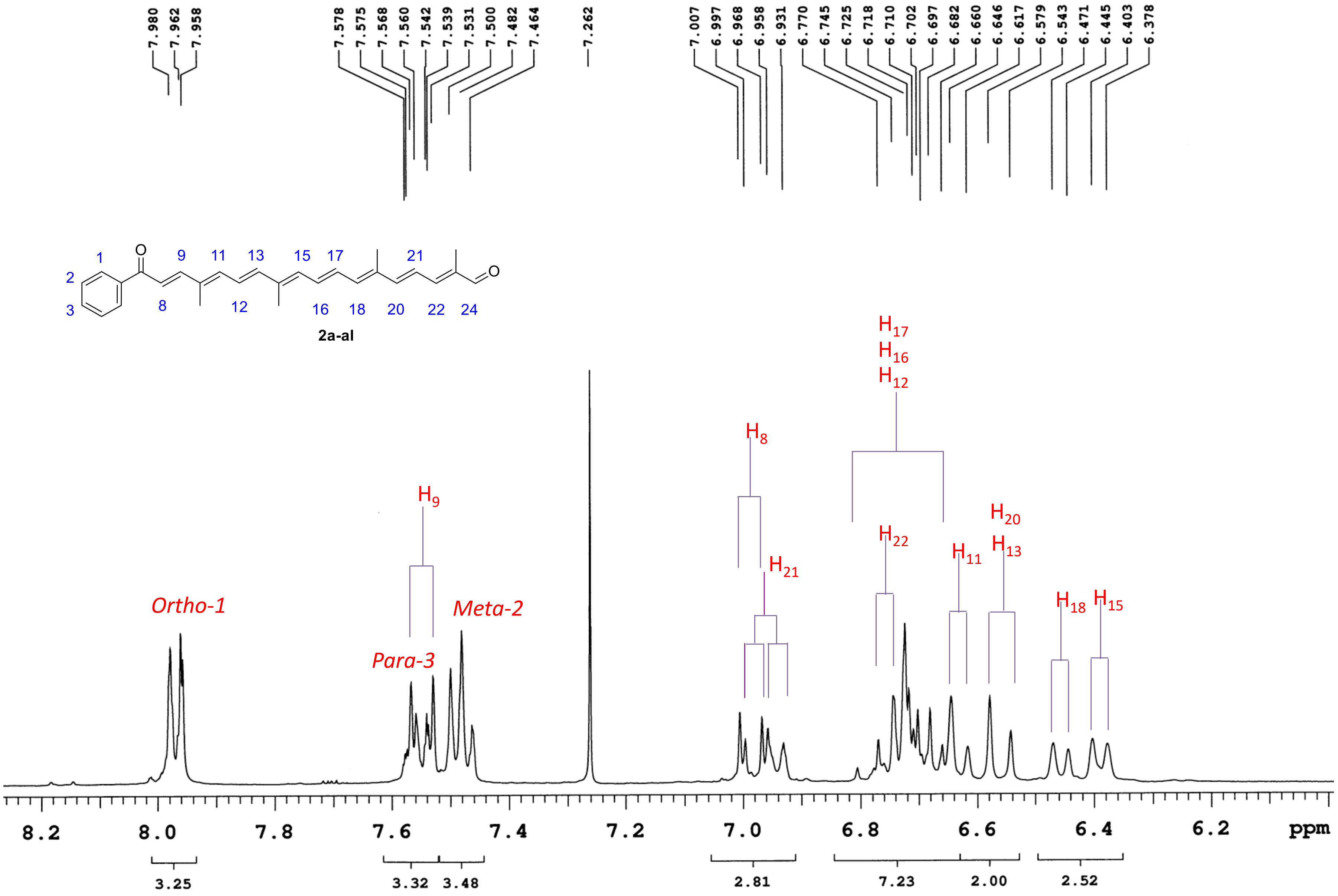

SGS-1421-11re

Sample Name	SGS-1421-11re	Pulse sequence	CARBON	Temperature	25	Study owner	vnmr1
Date collected	2021-11-17	Solvent	cdcl3	Spectrometer	Agilent-NMR.chem.com-vnmrs400	Operator	vnmr1

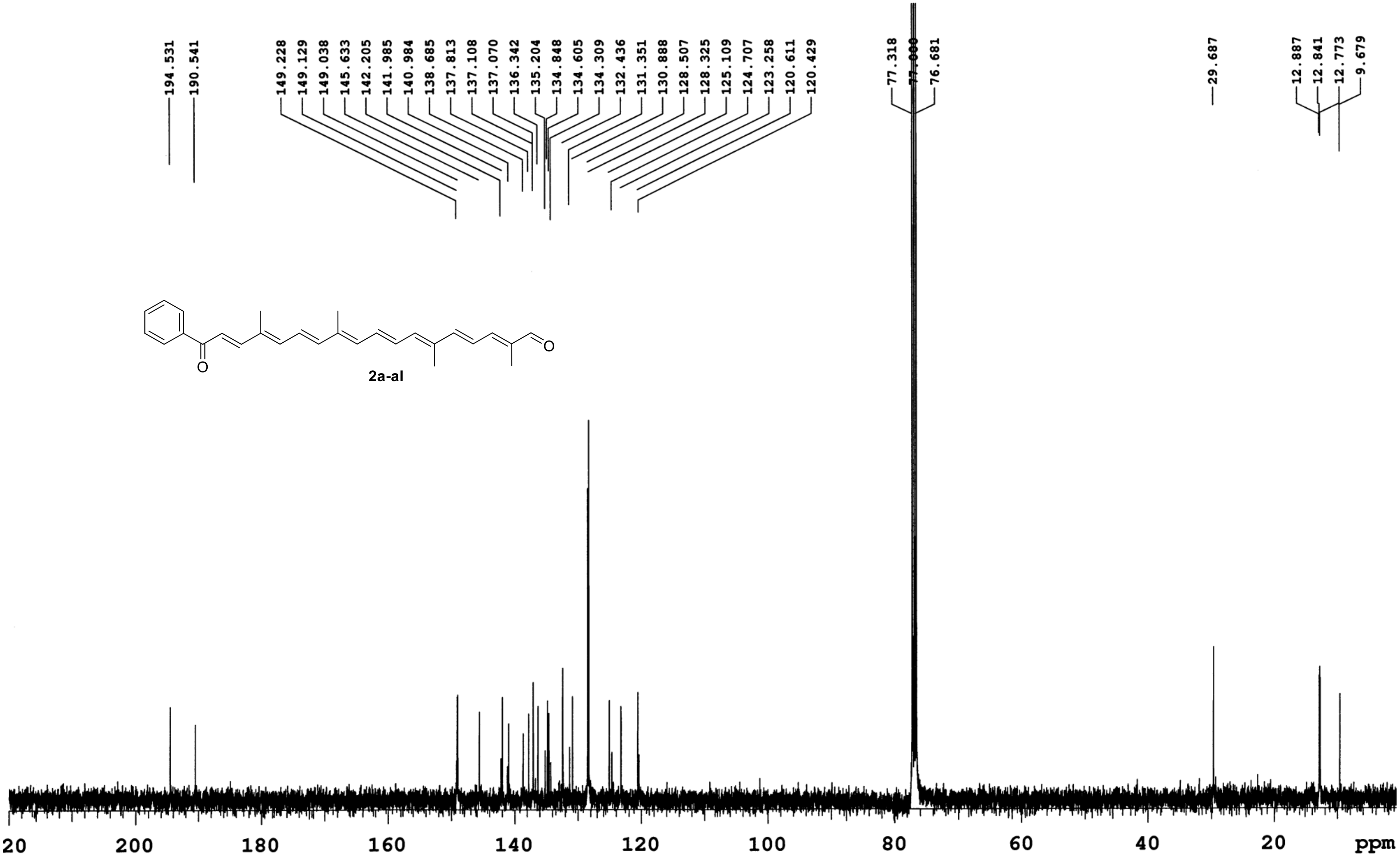

SGS-1557-3re

Sample Name **SGS-1557-3re** Pulse sequence **PROTON** Temperature **25** Study owner **vnmr1**
Date collected **2021-09-07** Solvent **cdcl3** Spectrometer **Agilent-NMR.chem.com-vnmrs400** Operator **vnmr1**

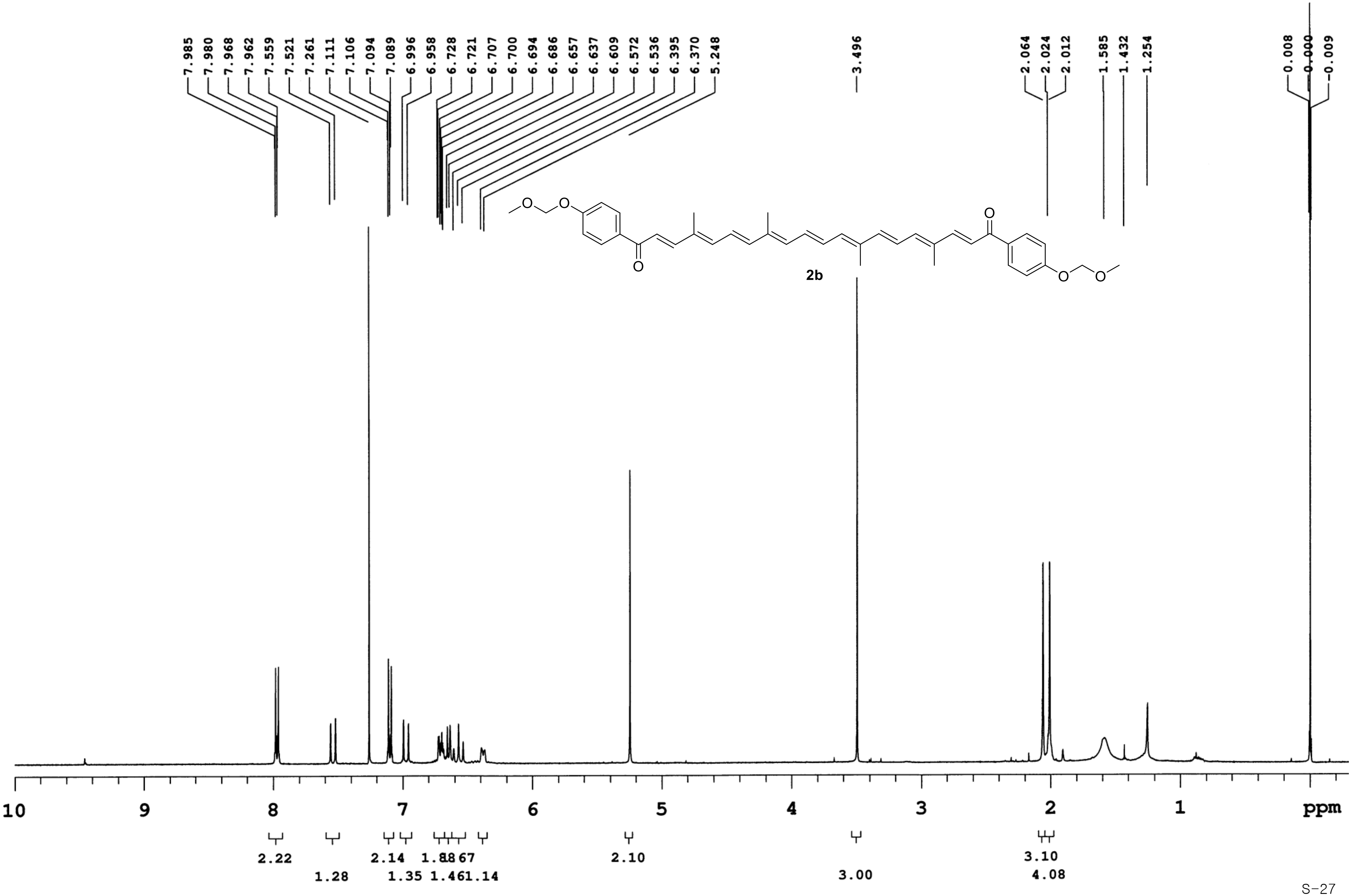

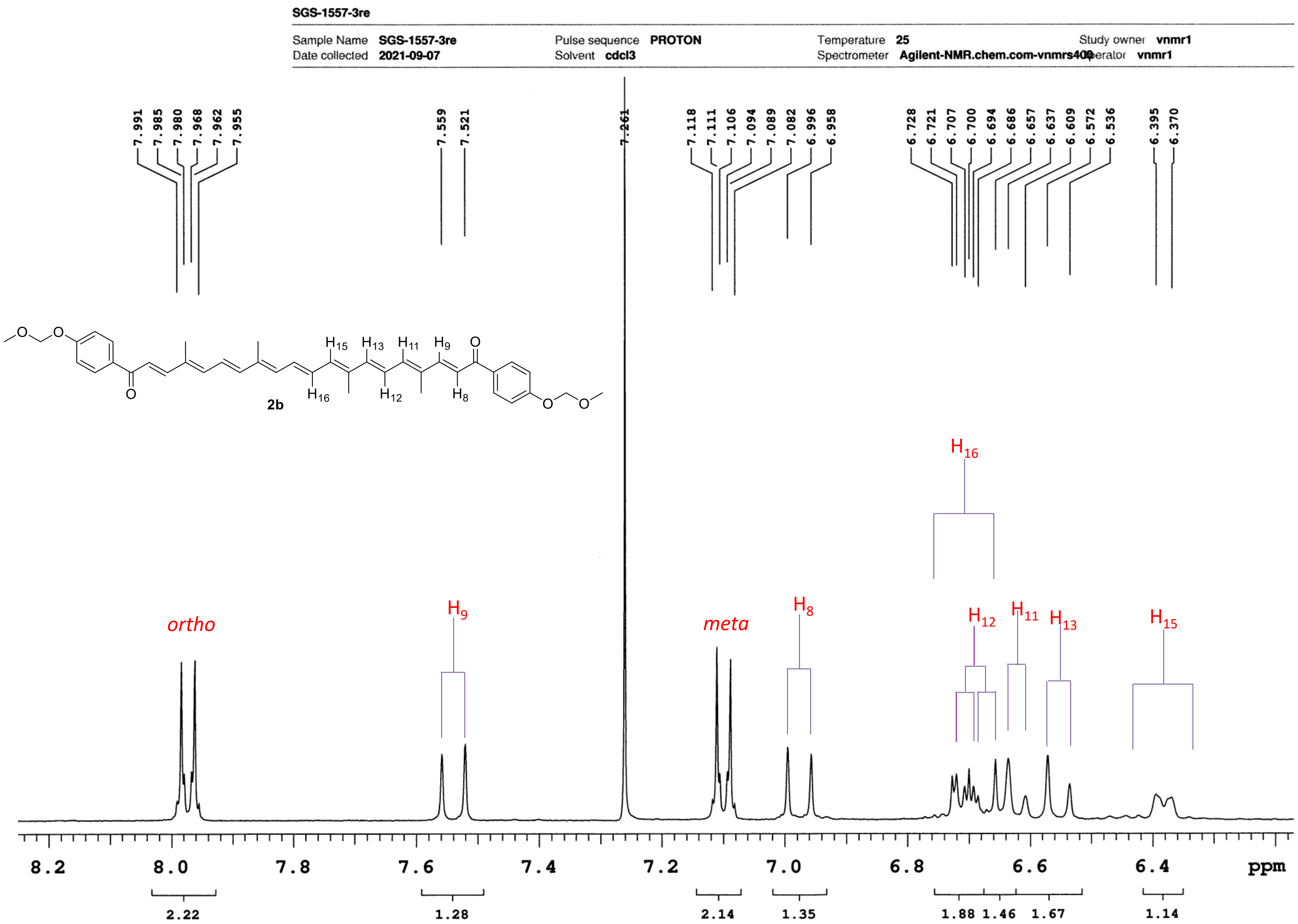
SGS-1557-3re
Sample Name SGS-1557-3re
Date collected 2021-09-07
Pulse sequence PROTON
Solvent cdcl3
Temperature 25
Spectrometer Agilent-NMR.chem.com-vnmrs400
Study owner vnmr1
Operator vnmr1
7.991 7.985 7.980 7.968 7.962 7.955
7.559 7.521
7.261
7.118 7.111 7.106 7.094 7.089 7.082 6.996 6.958
6.728 6.721 6.707 6.700 6.694 6.686 6.657 6.637 6.609 6.572 6.536
6.395 6.370
2b
H15 H13 H11 H9 H16 H12 H8
ortho
H9
meta
H8
H16
H12
H11
H13
H15
8.2 8.0 7.8 7.6 7.4 7.2 7.0 6.8 6.6 6.4 ppm
2.22 1.28 2.14 1.35 1.88 1.46 1.67 1.14

SGS-1557-3re-13C

Sample Name	**SGS-1557-3re-13C**	Pulse sequence	**CARBON**	Temperature	**25**	Study owner	**vnmr1**
Date collected	**2021-09-09**	Solvent	**cdcl3**	Spectrometer	**Agilent-NMR.chem.com-vnmrs400**	Operator	**vnmr1**

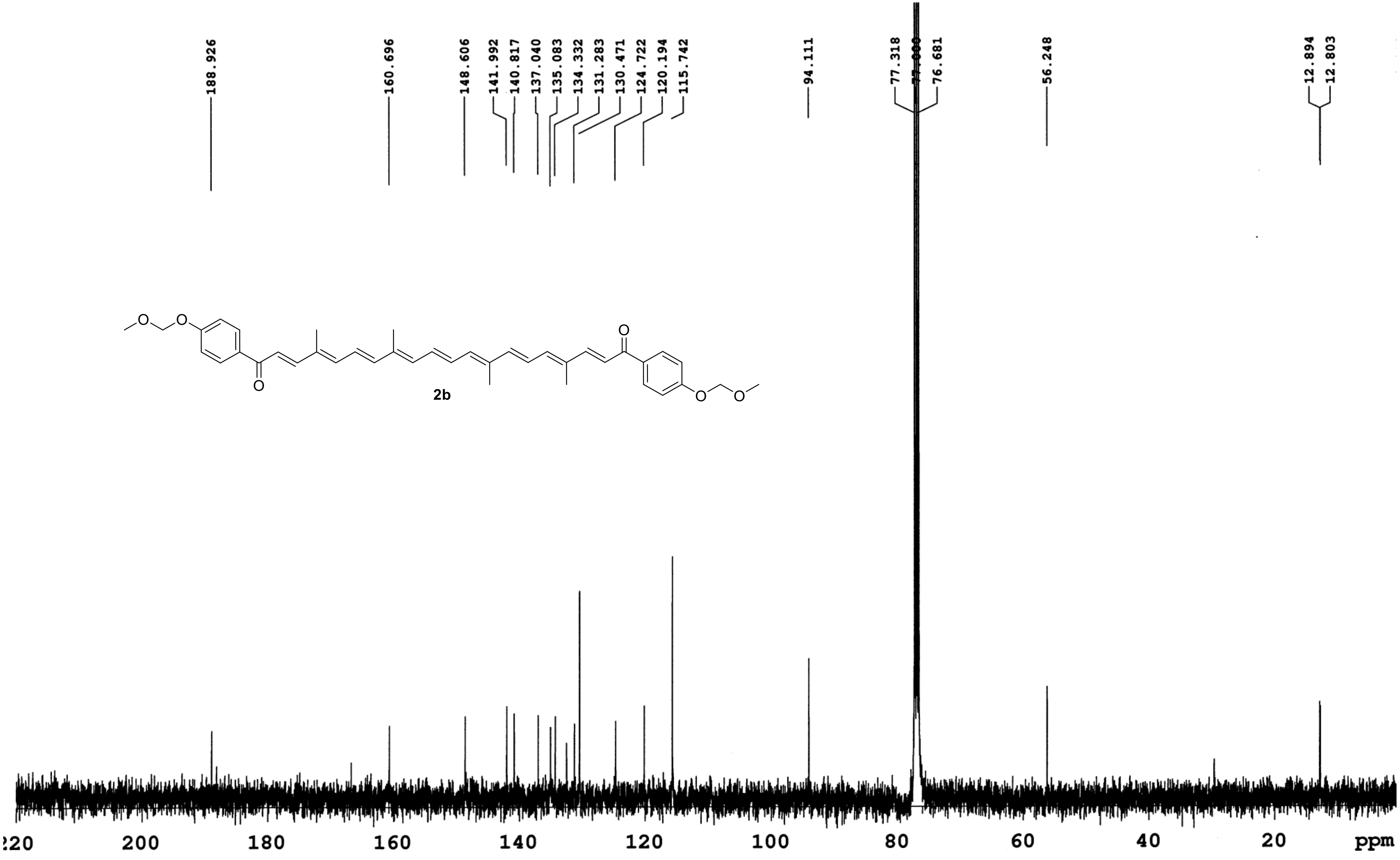

SGS-1557-3re-13C

Sample Name **SGS-1557-3re-13C** Date collected **2021-09-09**

Pulse sequence **CARBON** Solvent **cdcl3**

Temperature **25** Spectrometer **Agilent-NMR.chem.com-vnmrs400**

Study owner **vnmr1** Operator **vnmr1**

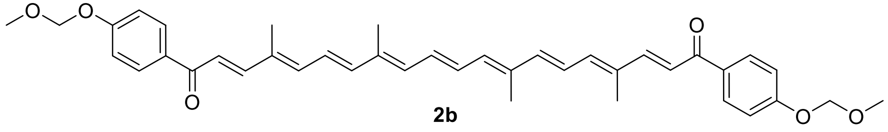

2b

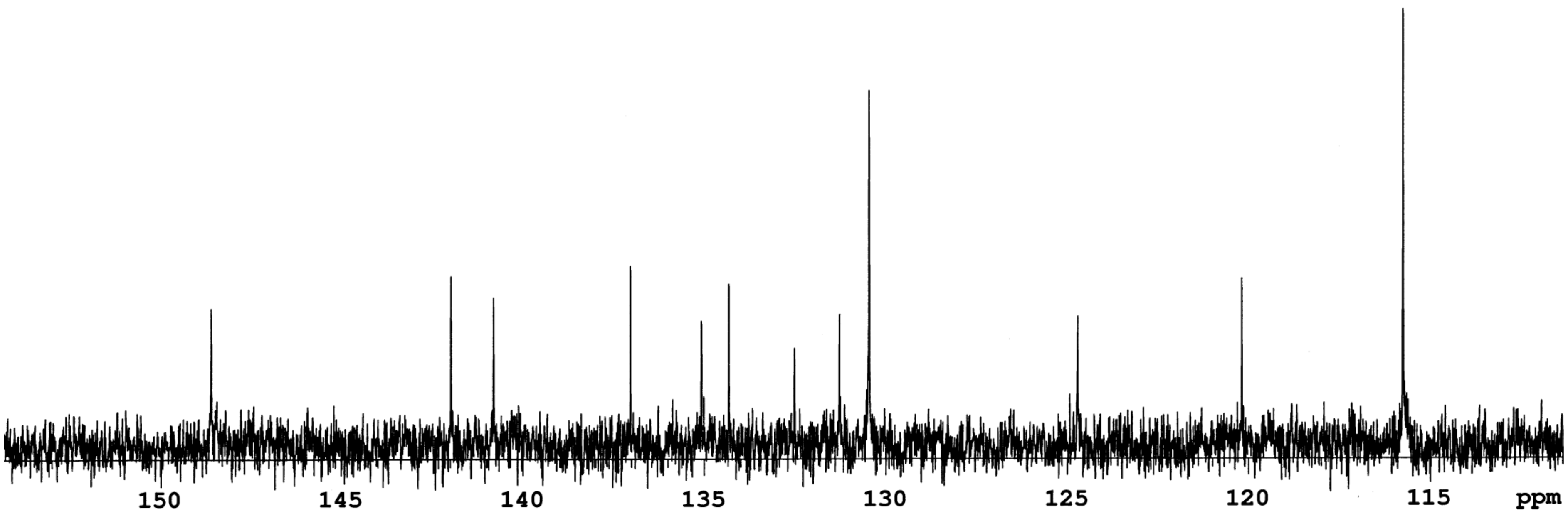

SGS-1521-EA2

Sample Name	**SGS-1521-EA2**	Pulse sequence	**PROTON**	Temperature	**25**	Study owner	**vnmr1**
Date collected	**2021-09-01**	Solvent	**cdcl3**	Spectrometer	**Agilent-NMR.chem.com-vnmrs400**	Operator	**vnmr1**

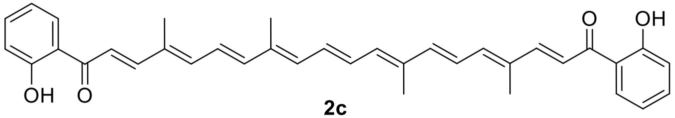

2c

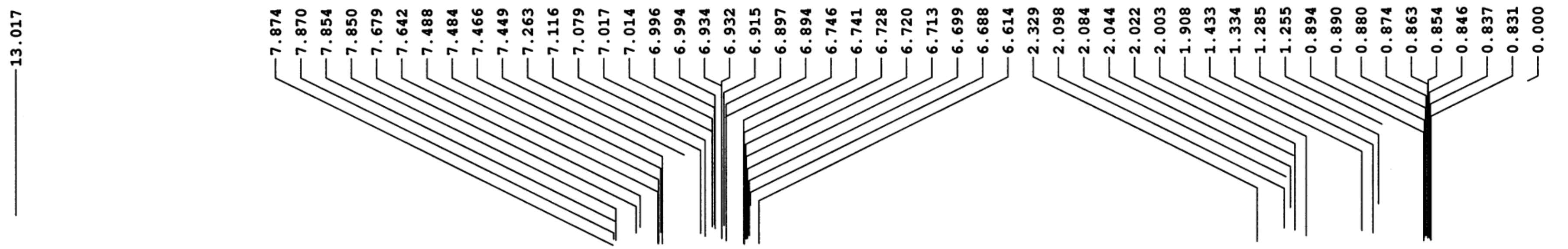

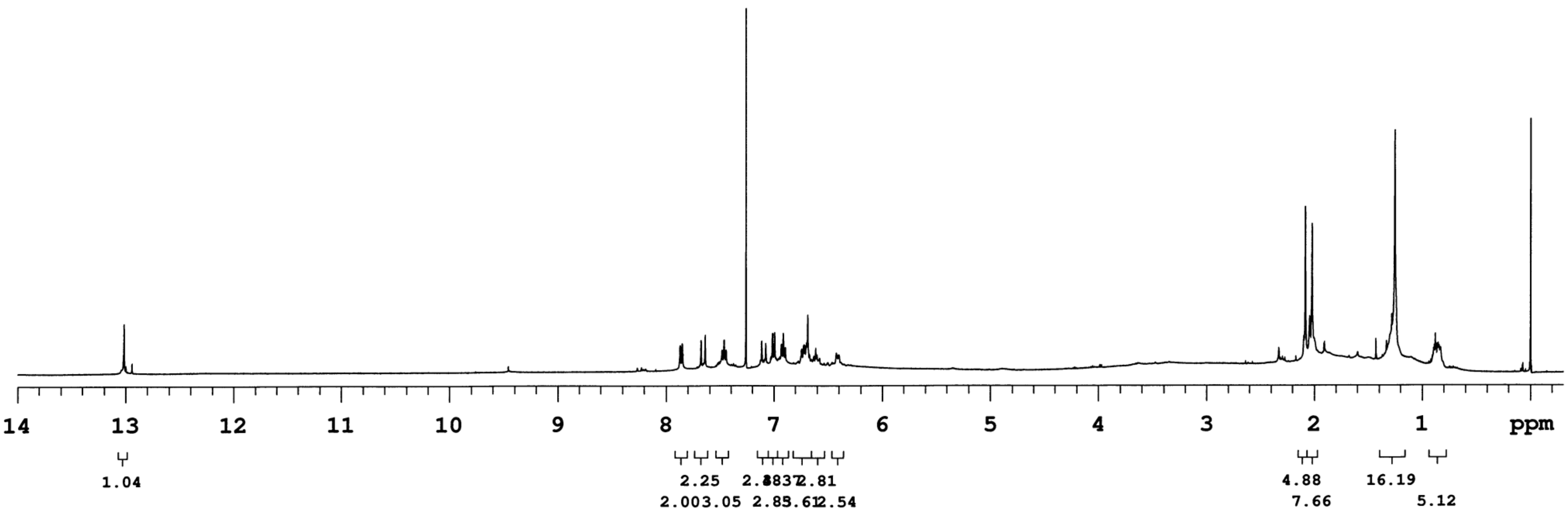

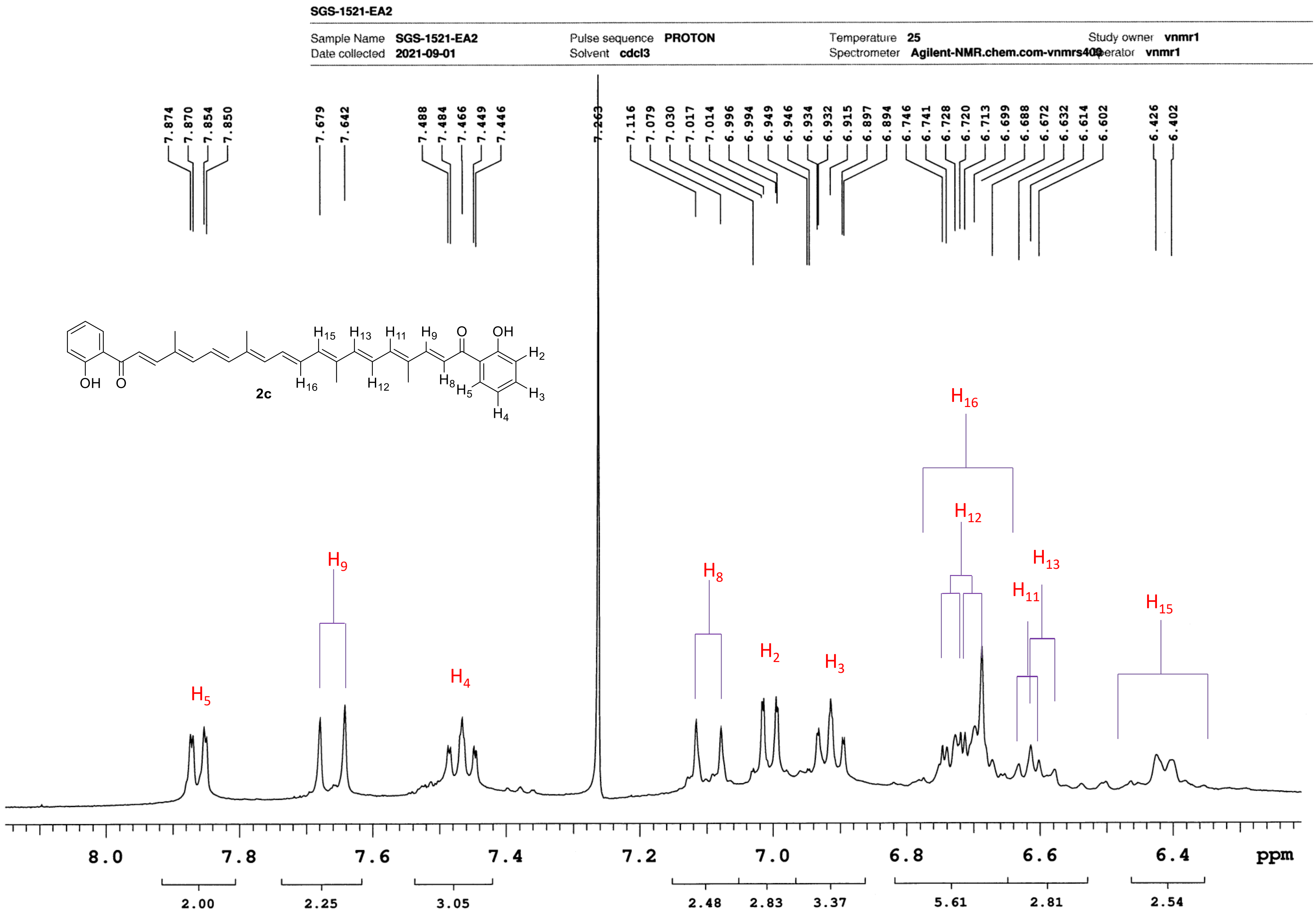
SGS-1521-EA2
Sample Name SGS-1521-EA2
Date collected 2021-09-01
Pulse sequence PROTON
Solvent cdcl3
Temperature 25
Spectrometer Agilent-NMR.chem.com-vnmrs400
Study owner vnmr1
Operator vnmr1
7.874 7.870 7.854 7.850 7.679 7.642 7.488 7.484 7.466 7.449 7.446 7.263 7.116 7.079 7.030 7.017 7.014 6.996 6.994 6.949 6.946 6.934 6.932 6.915 6.897 6.894 6.746 6.741 6.728 6.720 6.713 6.699 6.688 6.672 6.632 6.614 6.602 6.426 6.402
2c
OH O
H15 H13 H11 H9 O OH
H2
H16 H12 H8 H5 H3
H4
H16
H12
H13
H11
H15
H9
H8
H2
H3
H5
H4
8.0 7.8 7.6 7.4 7.2 7.0 6.8 6.6 6.4 ppm
2.00 2.25 3.05 2.48 2.83 3.37 5.61 2.81 2.54

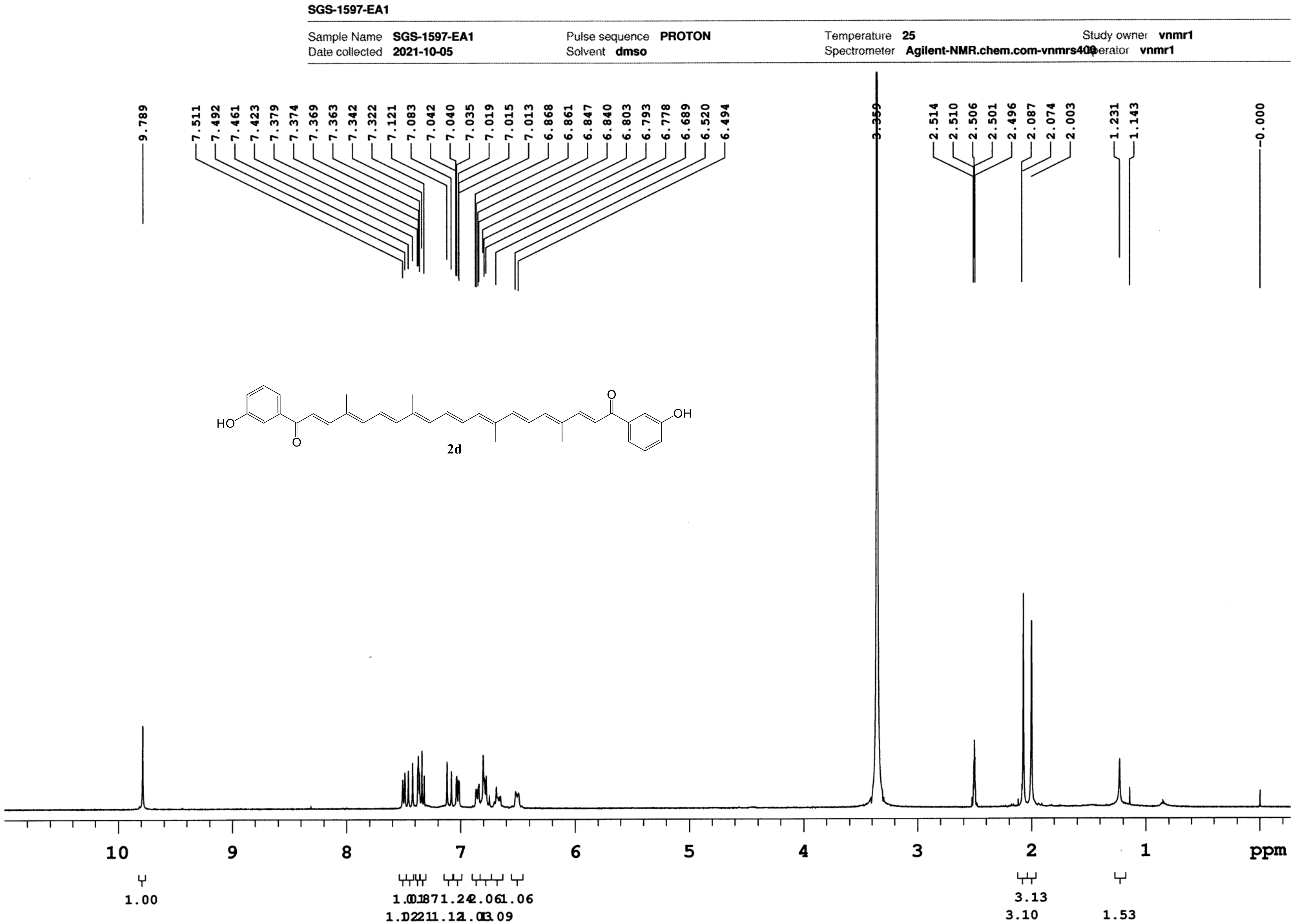
SGS-1597-EA1
Sample Name SGS-1597-EA1
Date collected 2021-10-05
Pulse sequence PROTON
Solvent dmso
Temperature 25
Spectrometer Agilent-NMR.chem.com-vnmrs400
Study owner vnmr1
Operator vnmr1
9.789
7.511 7.492 7.461 7.423 7.379 7.374 7.369 7.363 7.342 7.322 7.121 7.083 7.042 7.040 7.035 7.019 7.015 7.013 6.868 6.861 6.847 6.840 6.803 6.793 6.778 6.689 6.520 6.494
2.514 2.510 2.506 2.501 2.496 2.087 2.074 2.003
1.231 1.143
-0.000
HO
OH
O
O
2d
10 9 8 7 6 5 4 3 2 1 ppm
1.00
1.06
3.13
3.10
1.53

SGS-1597-EA1

Sample Name	SGS-1597-EA1	Pulse sequence	PROTON	Temperature	25	Study owner	vnmr1
Date collected	2021-10-05	Solvent	dmso	Spectrometer	Agilent-NMR.chem.com-vnmrs400	Operator	vnmr1

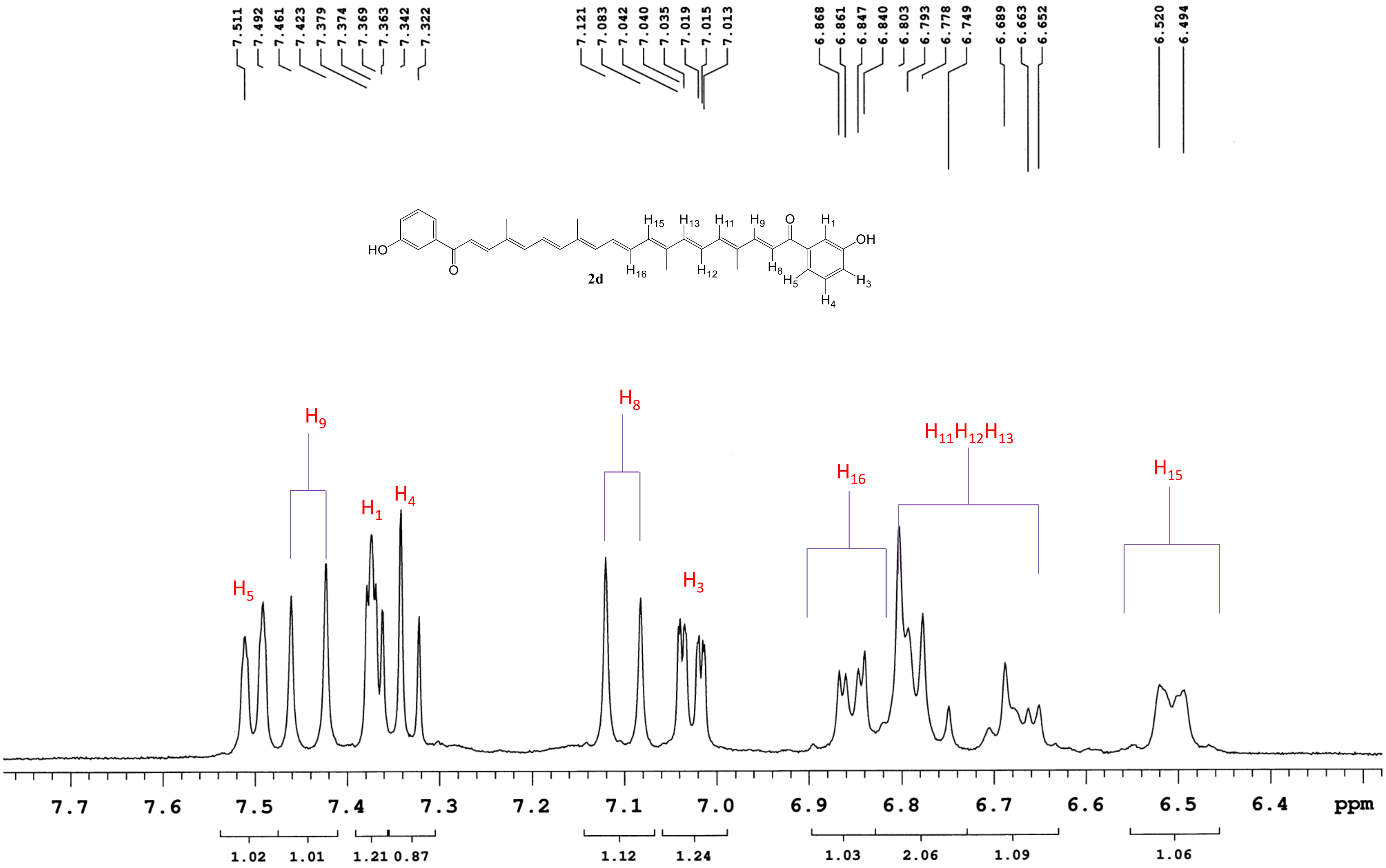

Parameter	Value
Data File Name	F:/ NMR/ 2110-koo-4-06/ SGS-1597-EA1_01/ SGS-1597-EA1_CARBON_01.fid/ fid
Instrument	vnmrs
Solvent	dmso
Probe	5mm_ATB
Number of Scans	2000
Acquisition Date	2021-10-06T01:45:21
Spectrometer Frequency	100.60

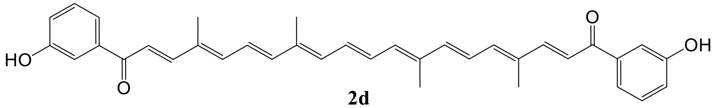

2d

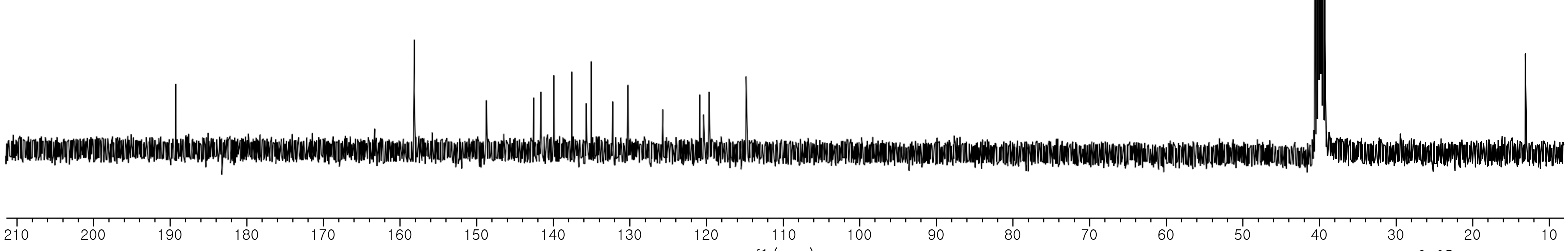

Parameter	Value
Data File Name	F:/ NMR/ 2110-koo-4-06/ SGS-1597-EA1_01/ SGS-1597-EA1_CARBON_01.fid/ fid
Instrument	vnmrs
Solvent	dmso
Probe	5mm_ATB
Number of Scans	2000
Acquisition Date	2021-10-06T01:45:21
Spectrometer Frequency	100.60

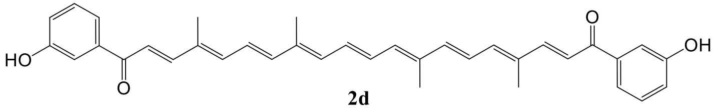

2d

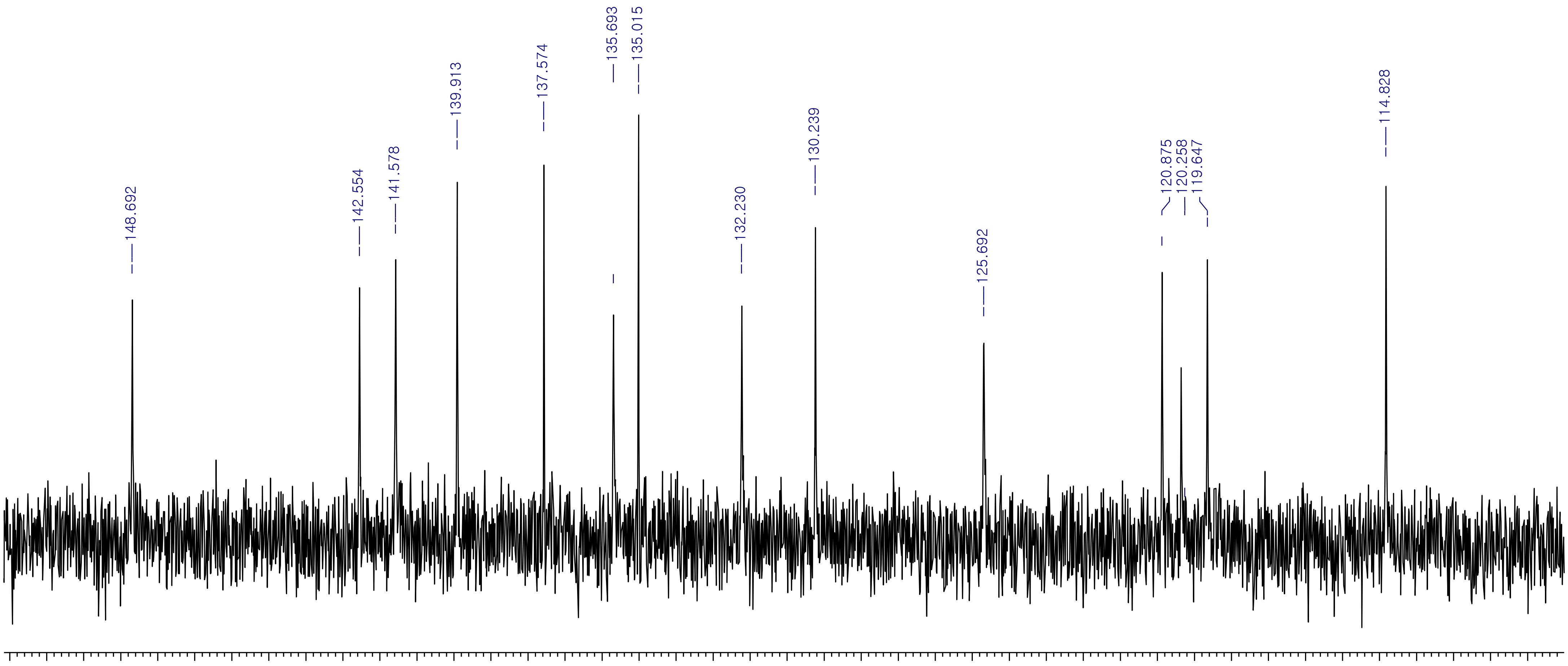

SGS-2022-2re-1

Sample Name	**SGS-2022-2re-1**	Pulse sequence	**PROTON**	Temperature	**25**	Study owner	**vnmr1**
Date collected	**2022-01-04**	Solvent	**dmso**	Spectrometer	**Agilent-NMR.chem.com-vnmrs400**	Operator	**vnmr1**

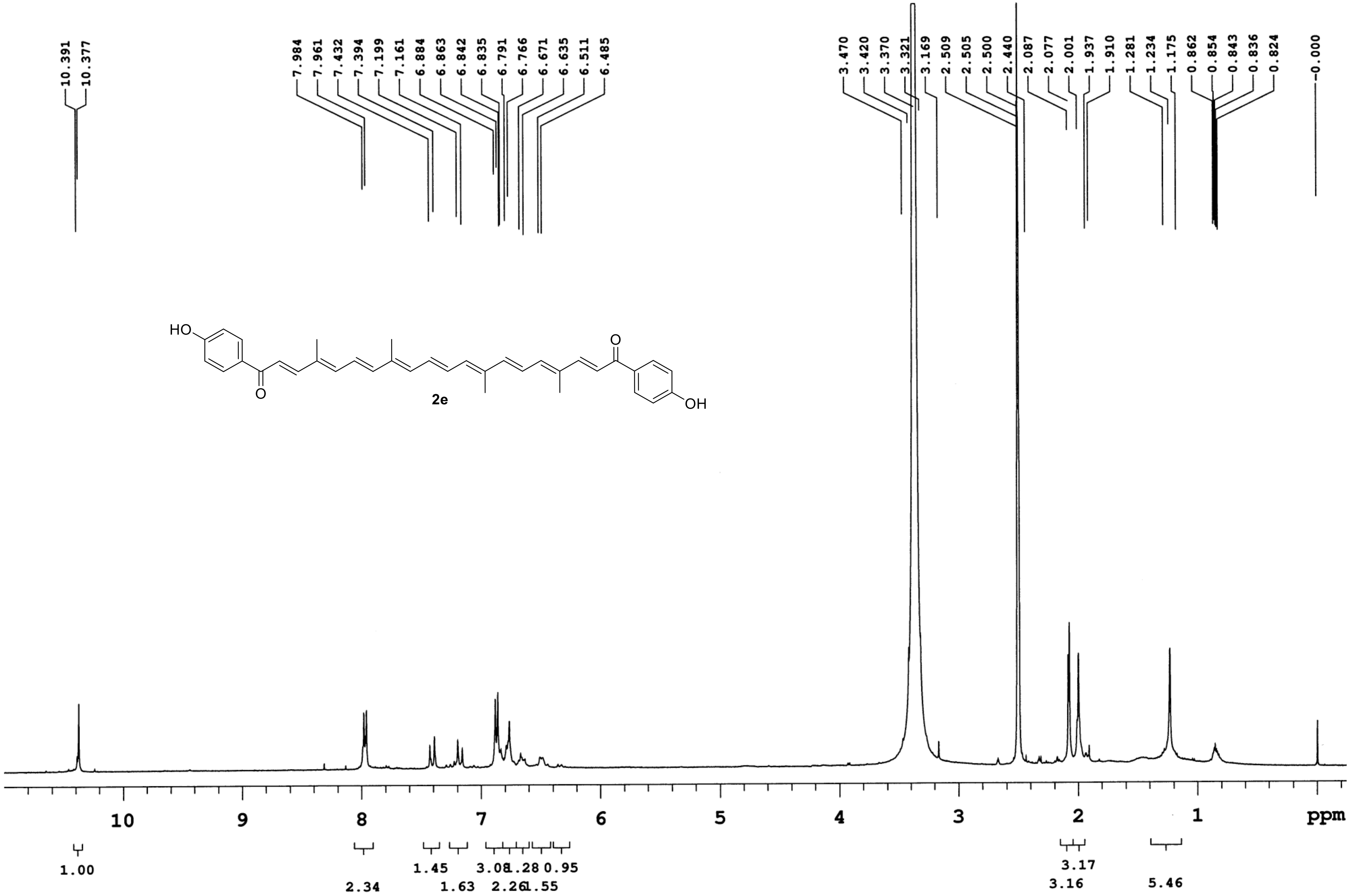

SGS-2022-2re-1

Sample Name **SGS-2022-2re-1** Pulse sequence **PROTON** Temperature **25** Study owner **vnmr1**
Date collected **2022-01-04** Solvent **dmso** Spectrometer **Agilent-NMR.chem.com-vnmrs400** Operator **vnmr1**

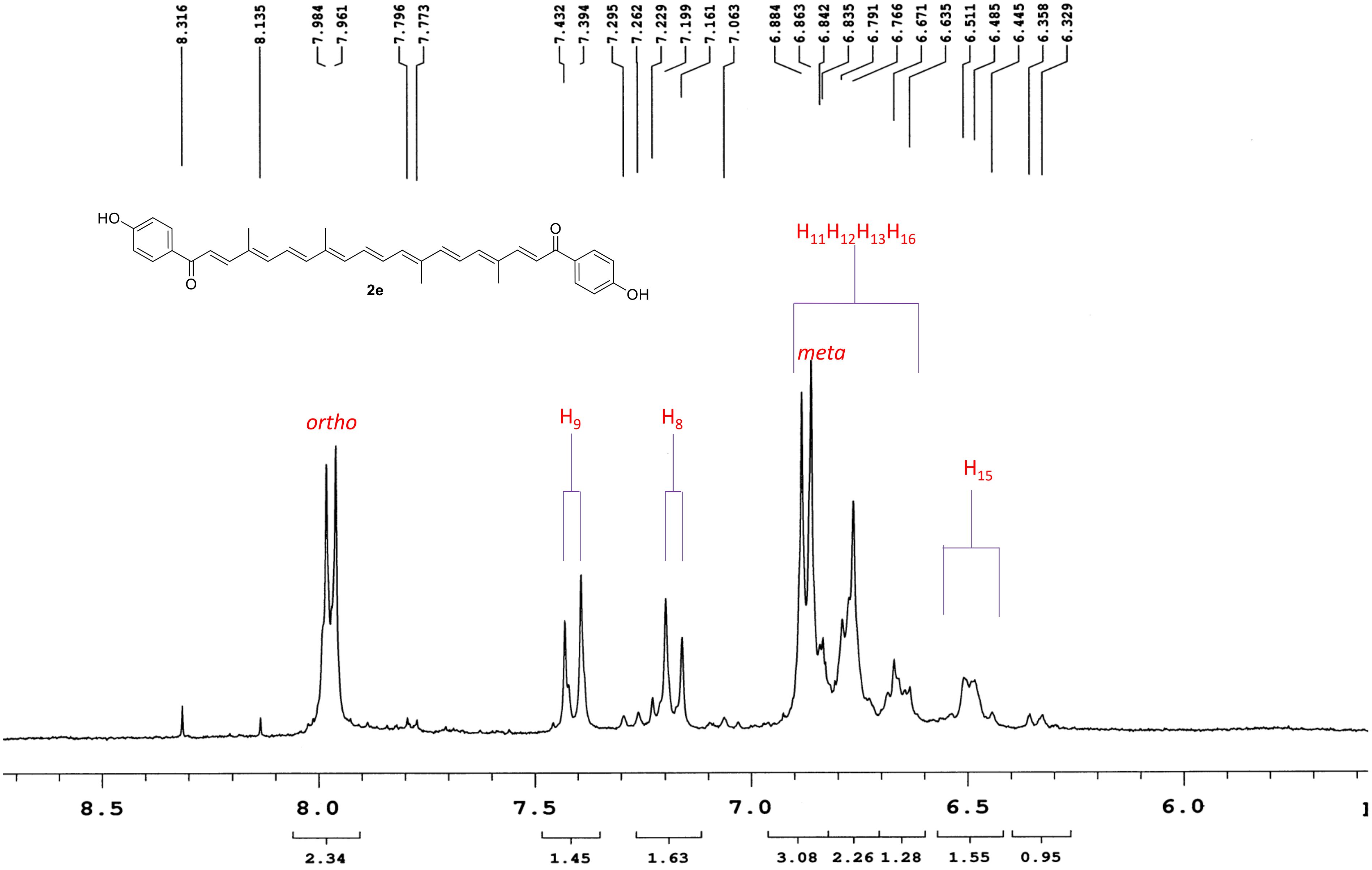

SGS-1547-1

Sample Name	SGS-1547-1	Pulse sequence	PROTON	Temperature	25	Study owner	vnmr1
Date collected	2021-08-31	Solvent	cdcl3	Spectrometer	Agilent-NMR.chem.com-vnmrs400	Operator	vnmr1

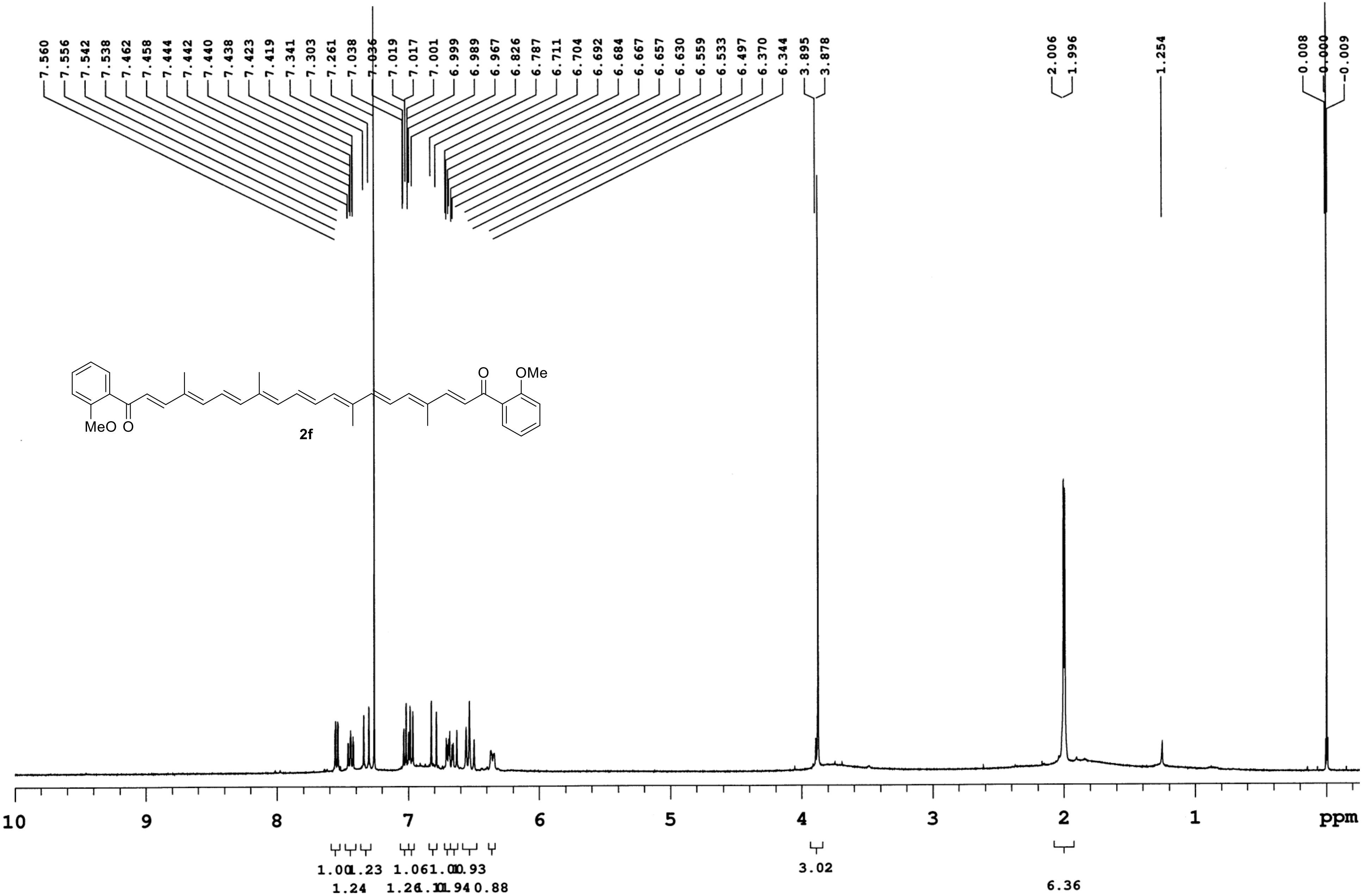

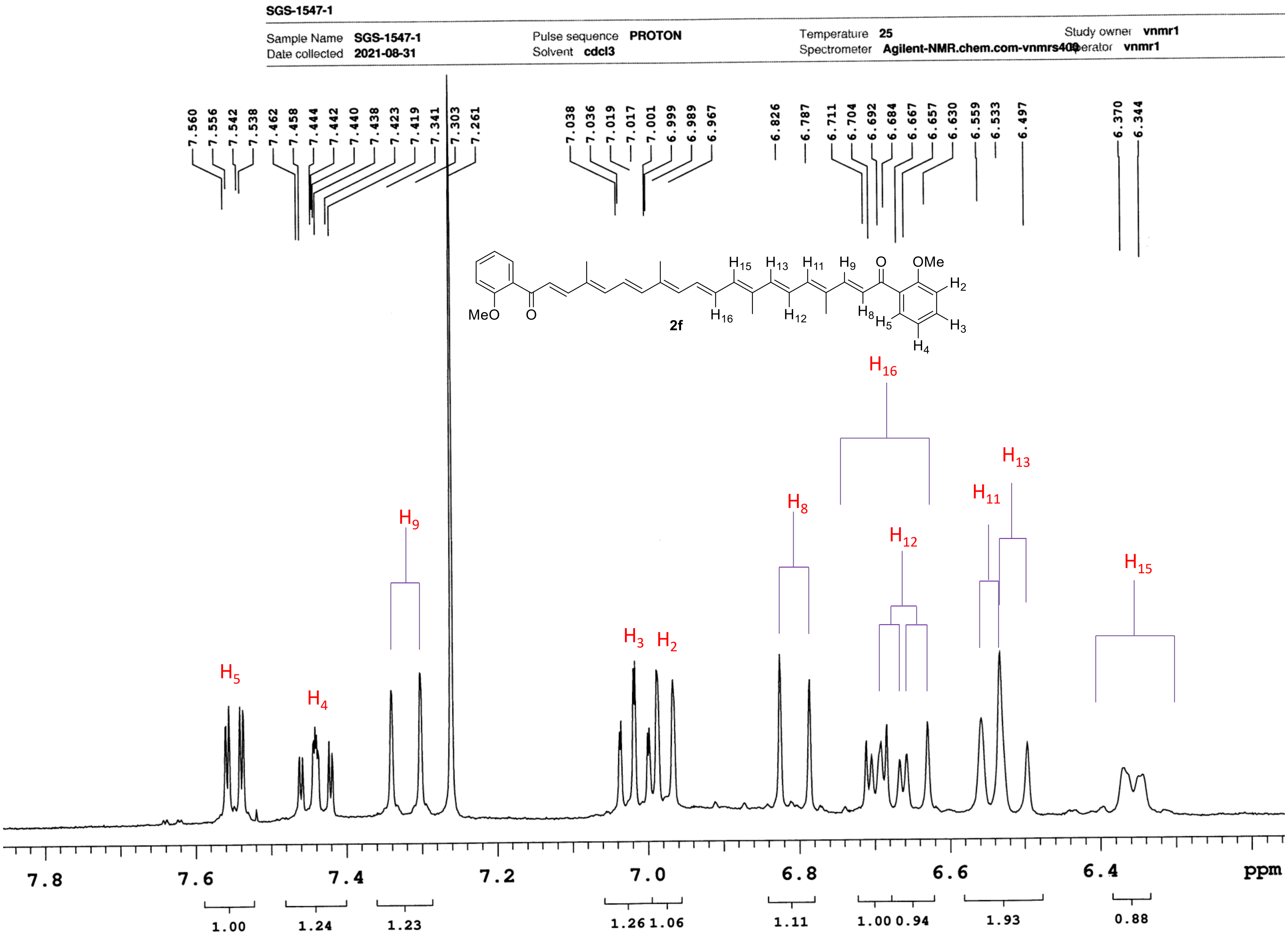
SGS-1547-1
Sample Name SGS-1547-1
Date collected 2021-08-31
Pulse sequence PROTON
Solvent cdcl3
Temperature 25
Spectrometer Agilent-NMR.chem.com-vnmrs400
Study owner vnmr1
Operator vnmr1
7.560 7.556 7.542 7.538 7.462 7.458 7.444 7.442 7.440 7.438 7.423 7.419 7.341 7.303 7.261
7.038 7.036 7.019 7.017 7.001 6.999 6.989 6.967
6.826 6.787 6.711 6.704 6.692 6.684 6.667 6.657 6.630 6.559 6.533 6.497
6.370 6.344
MeO
O
2f
H15 H13 H11 H9 O OMe
H2
H16 H12 H8 H5 H3
H4
H16
H13
H11
H9
H8
H12
H15
H3 H2
H5
H4
7.8 7.6 7.4 7.2 7.0 6.8 6.6 6.4 ppm
1.00 1.24 1.23 1.26 1.06 1.11 1.00 0.94 1.93 0.88

Parameter	Value
Data File Name	F:/ NMR/ 2109-koo-4-01/ SGS-1547-1_01/ SGS-1547-1_CARBON_01.fid/ fid
Instrument	vnmrs
Solvent	cdcl3
Probe	5mm_ATB
Number of Scans	3000
Acquisition Date	2021-09-01T10:36:07
Spectrometer Frequency	100.60

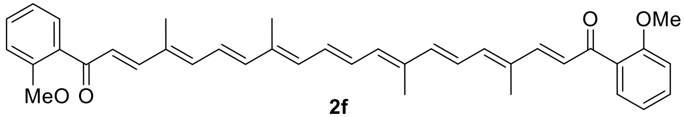

2f

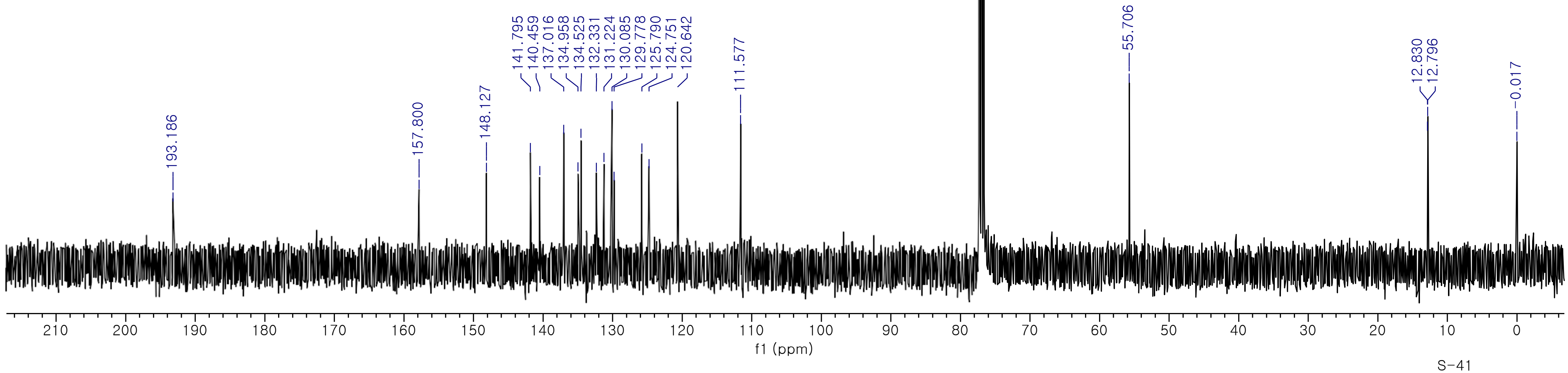

SGS-1600-2re

Sample Name	**SGS-1600-2re**	Pulse sequence	**PROTON**	Temperature	**25**	Study owner	**vnmr1**
Date collected	**2021-10-08**	Solvent	**cdcl3**	Spectrometer	**Agilent-NMR.chem.com-vnmrs400**	Operator	**vnmr1**

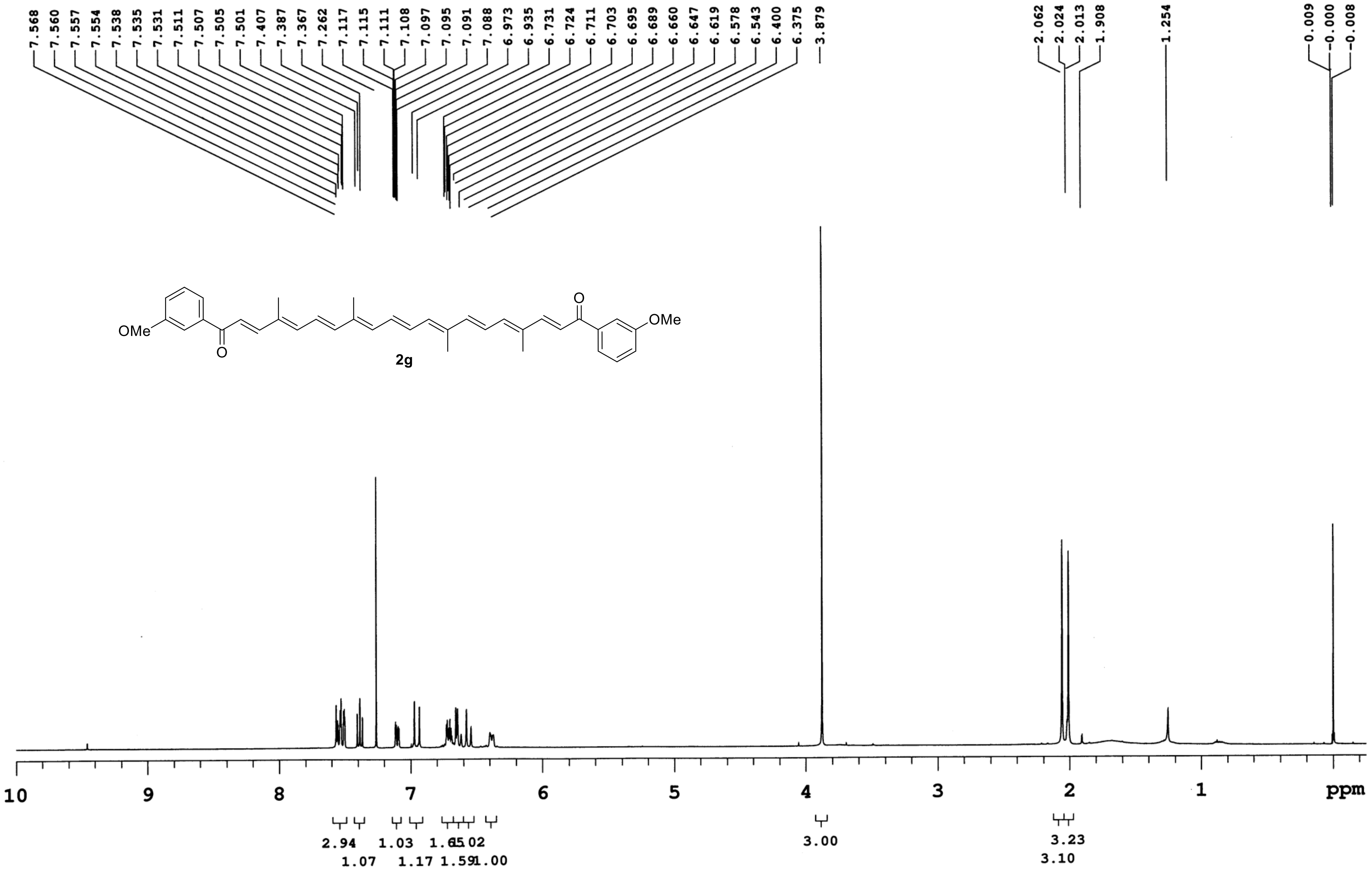

SGS-1600-2re

Sample Name	SGS-1600-2re	Pulse sequence	PROTON	Temperature	25
Date collected	2021-10-08	Solvent	cdcl3	Spectrometer	Agilent-NMR.chem.com-vnmrs400

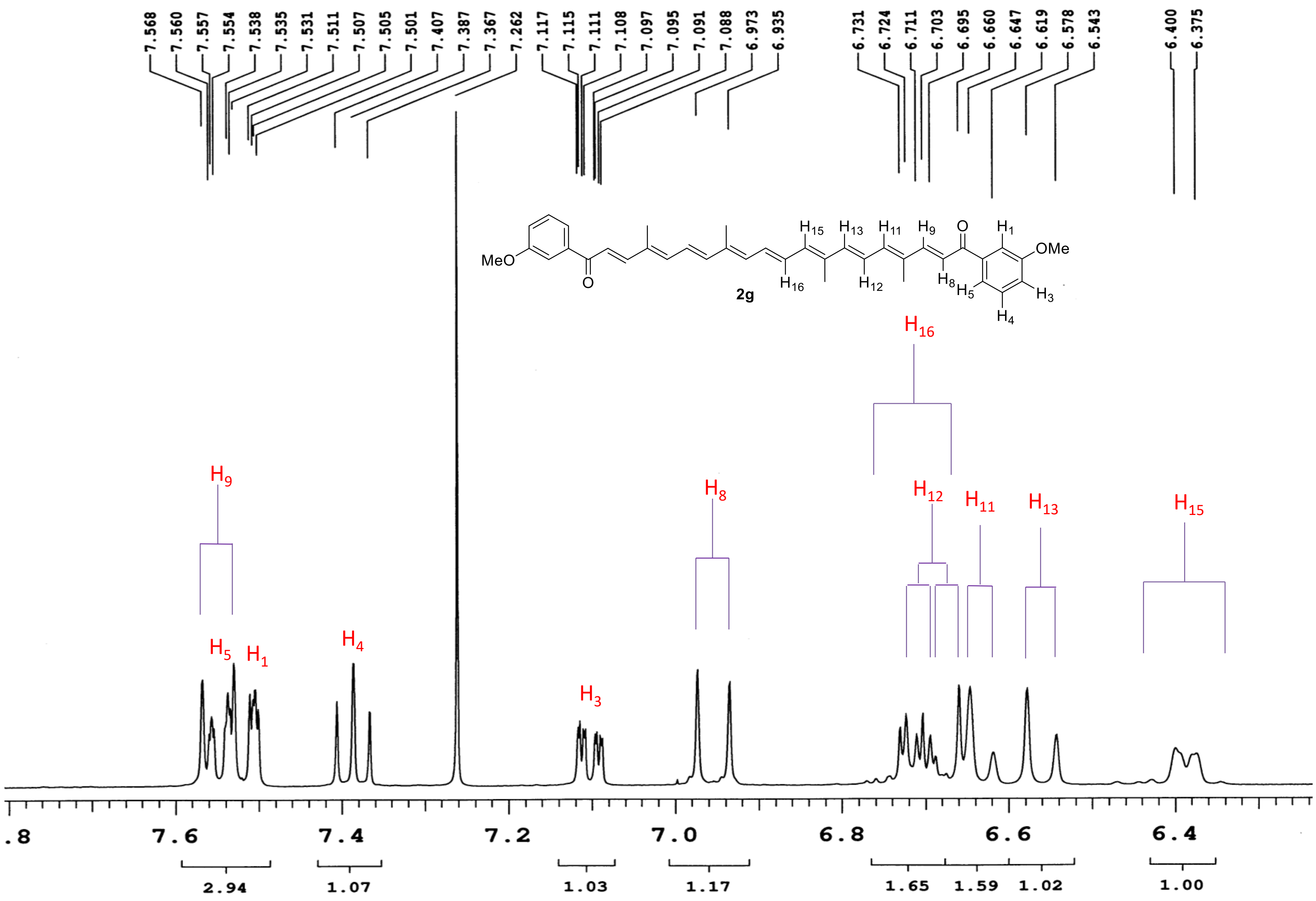

Parameter	Value
Data File Name	F:/ NMR/ 2110-koo-4-12/ SGS-1600-2re-13C_01/ SGS-1600-2re-13C_CARBON_01.fid/fid
Instrument	vnmrs
Solvent	cdcl3
Probe	5mm_ATB
Number of Scans	2000
Acquisition Date	2021-10-08T20:33:05
Spectrometer Frequency	100.60

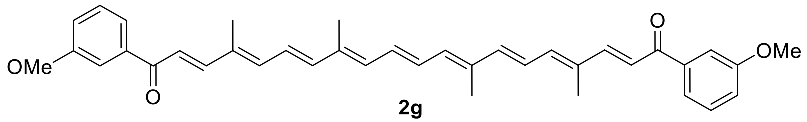

2g

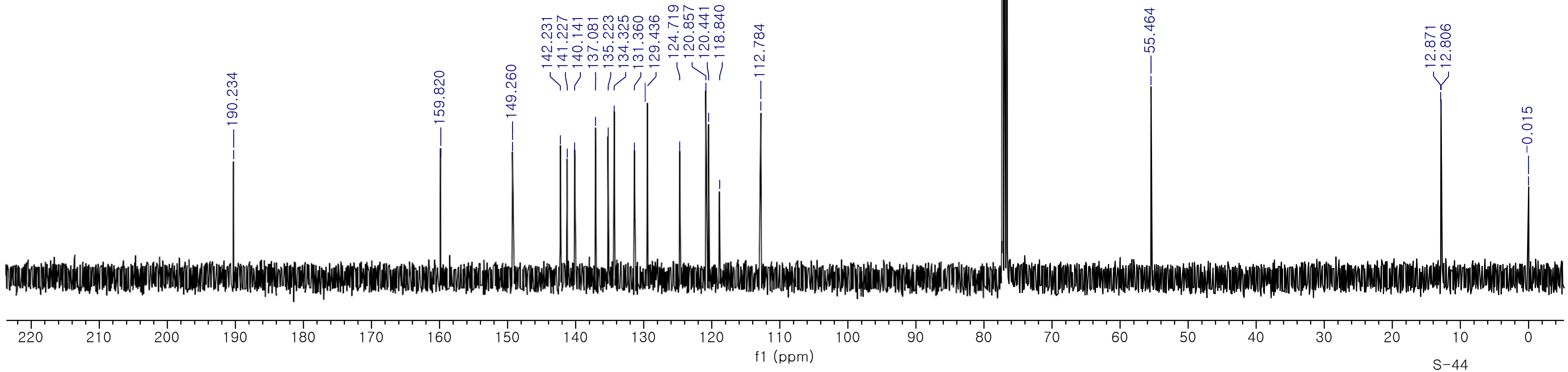

SGS-1629-3re

Sample Name SGS-1629-3re
Date collected 2022-01-05
Pulse sequence PROTON
Solvent cdcl3
Temperature 25
Spectrometer Agilent-NMR.chem.com-vnmrs400
Study owner vnmr1
Operator vnmr1

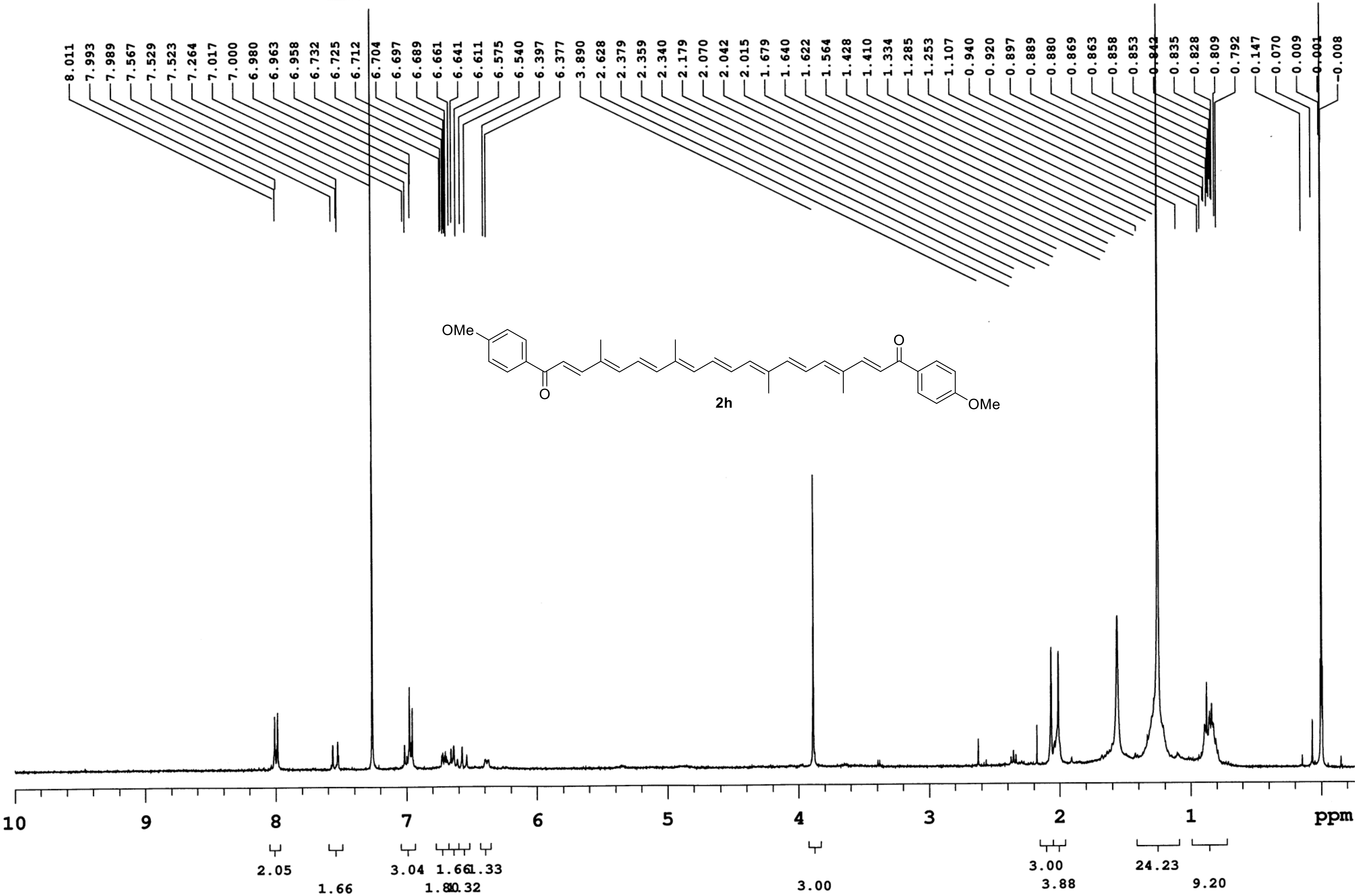

2h

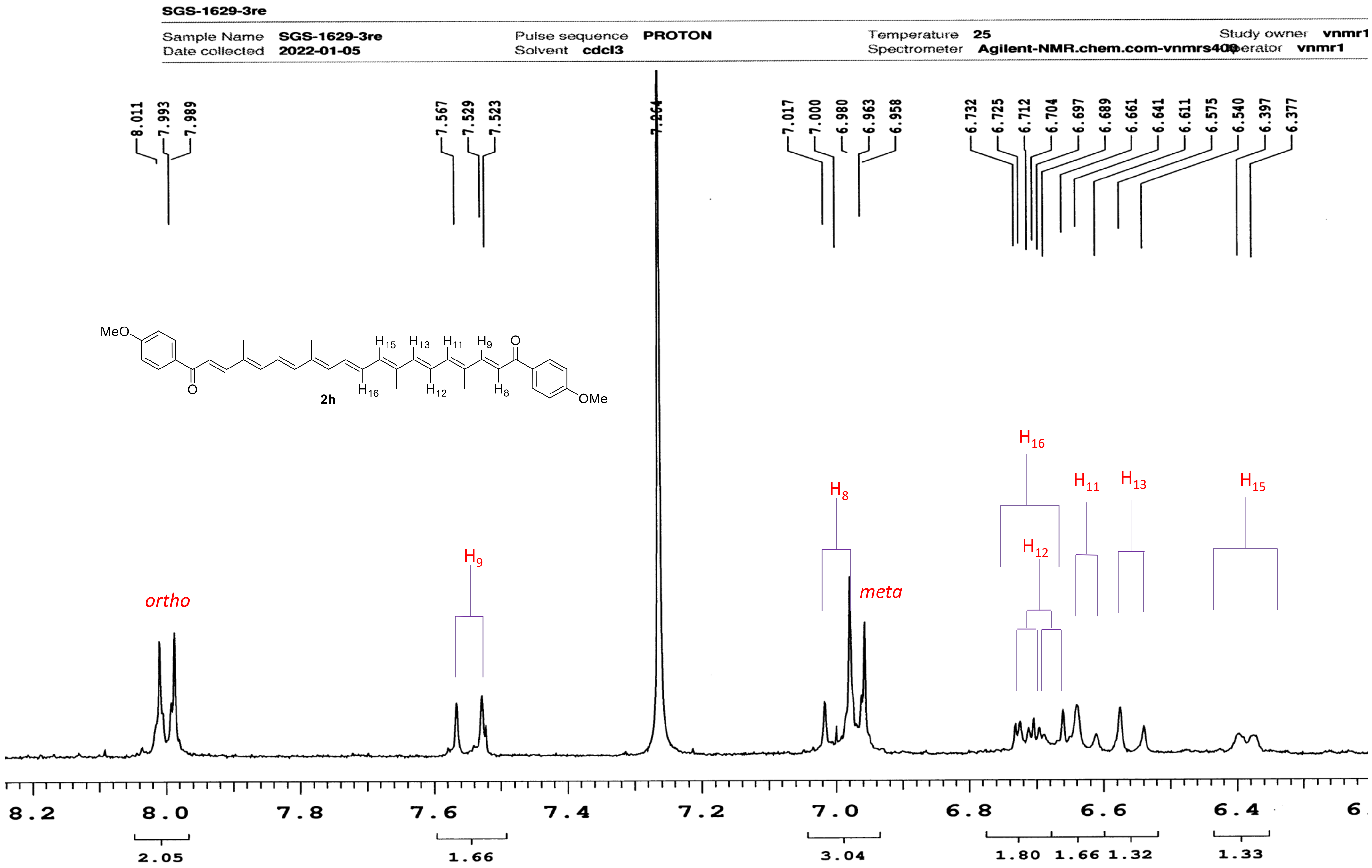

SGS-1629-3re
Sample Name SGS-1629-3re
Date collected 2022-01-05
Pulse sequence PROTON
Solvent cdcl3
Temperature 25
Spectrometer Agilent-NMR.chem.com-vnmrs400
Study owner vnmr1
Operator vnmr1
8.011 7.993 7.989
7.567 7.529 7.523
7.264
7.017 7.000 6.980 6.963 6.958
6.732 6.725 6.712 6.704 6.697 6.689 6.661 6.641 6.611 6.575 6.540 6.397 6.377
MeO
O
H15 H13 H11 H9
H16 H12 H8
2h
OMe
ortho
H9
H8
meta
H16
H12
H11
H13
H15
8.2 8.0 7.8 7.6 7.4 7.2 7.0 6.8 6.6 6.4 6.
2.05 1.66 3.04 1.80 1.66 1.32 1.33

SGS-1573-4t

Sample Name	**SGS-1573-4t**	Pulse sequence	**PROTON**	Temperature	**25**	Study owner	**vnmr1**
Date collected	**2021-09-24**	Solvent	**cdcl3**	Spectrometer	**Agilent-NMR.chem.com-vnmrs400**	Operator	**vnmr1**

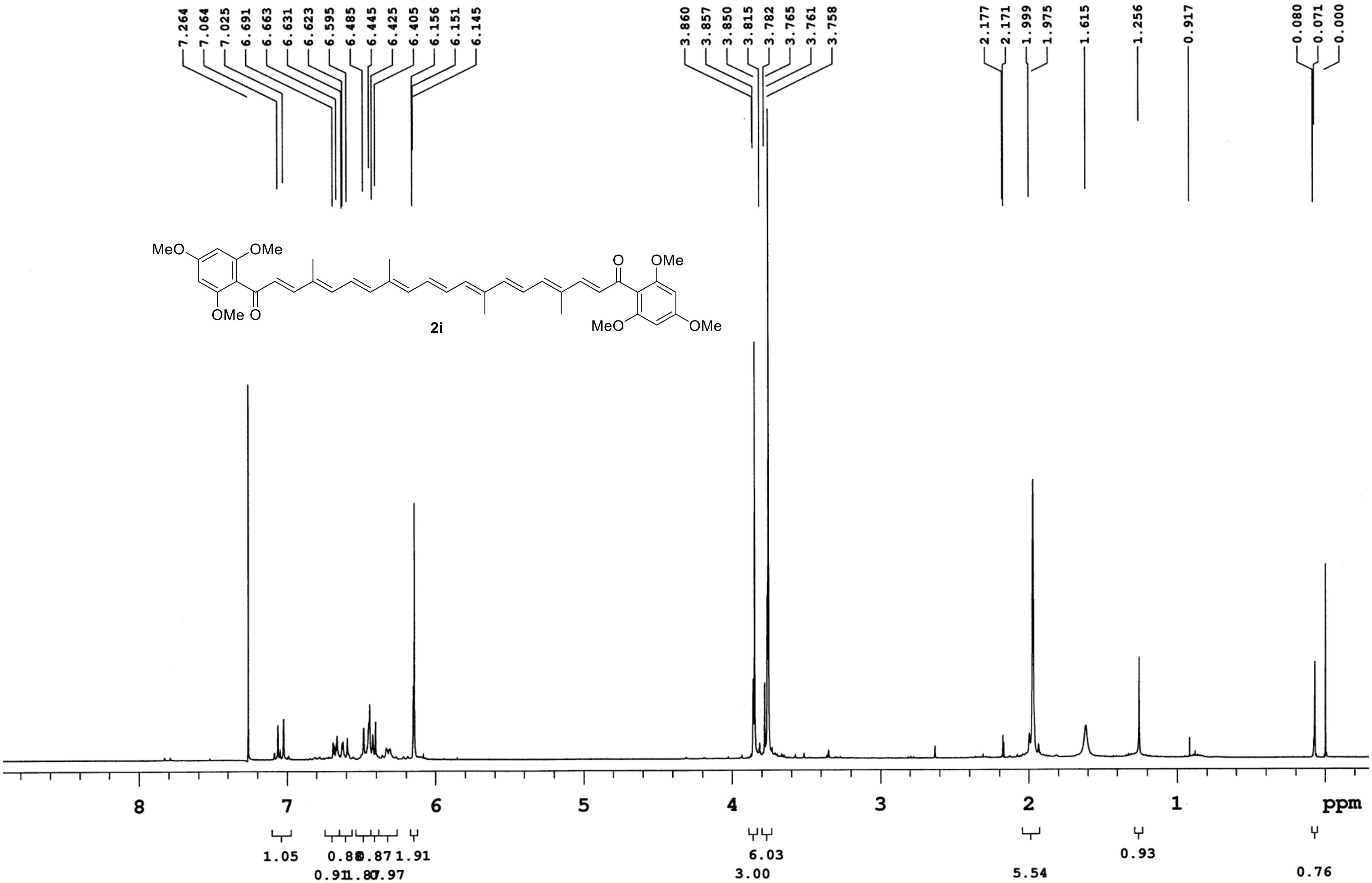

SGS-1573-4t

Sample Name SGS-1573-4t
Date collected 2021-09-24
Pulse sequence PROTON
Solvent cdcl3
Temperature 25
Spectrometer Agilent-NMR.chem.com-vnmrs400
Study owner vnmr1
Operator vnmr1

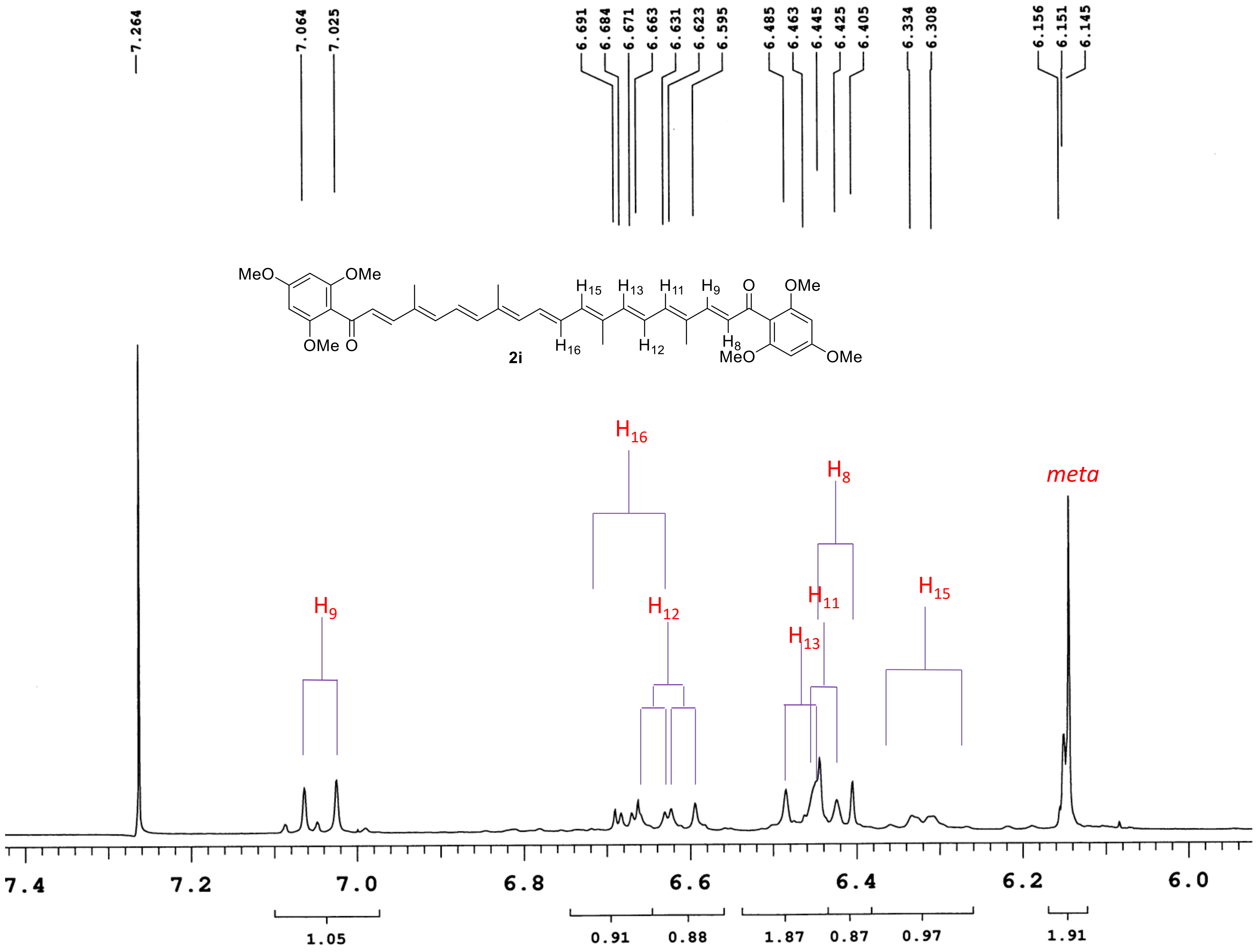

SGS-1573-4t

Sample Name **SGS-1573-4t** Pulse sequence **CARBON** Temperature **25** Study owner **vnmr1**
Date collected **2021-09-29** Solvent **cdcl3** Spectrometer **Agilent-NMR.chem.com-vnmrs400** Operator **vnmr1**

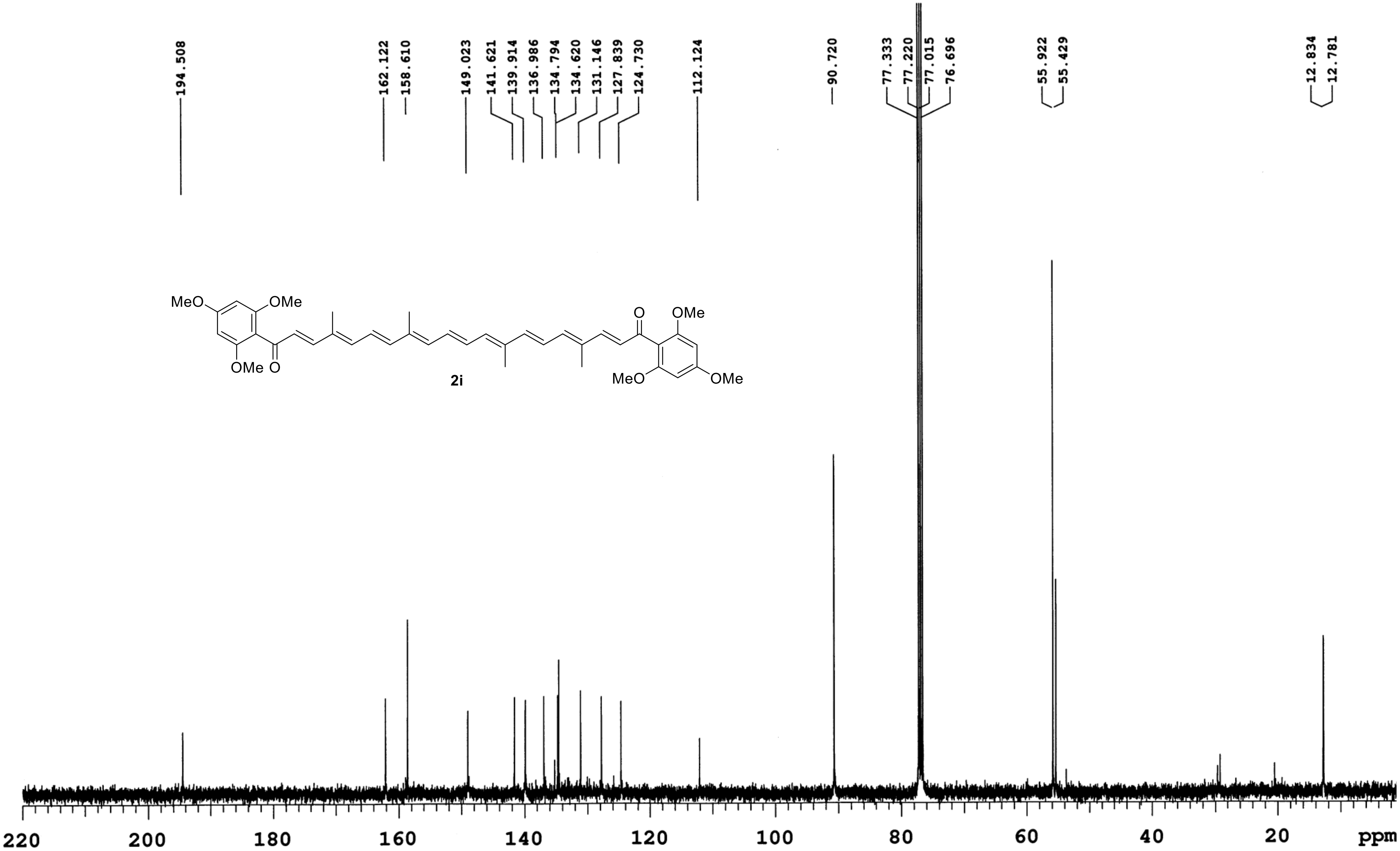

2i

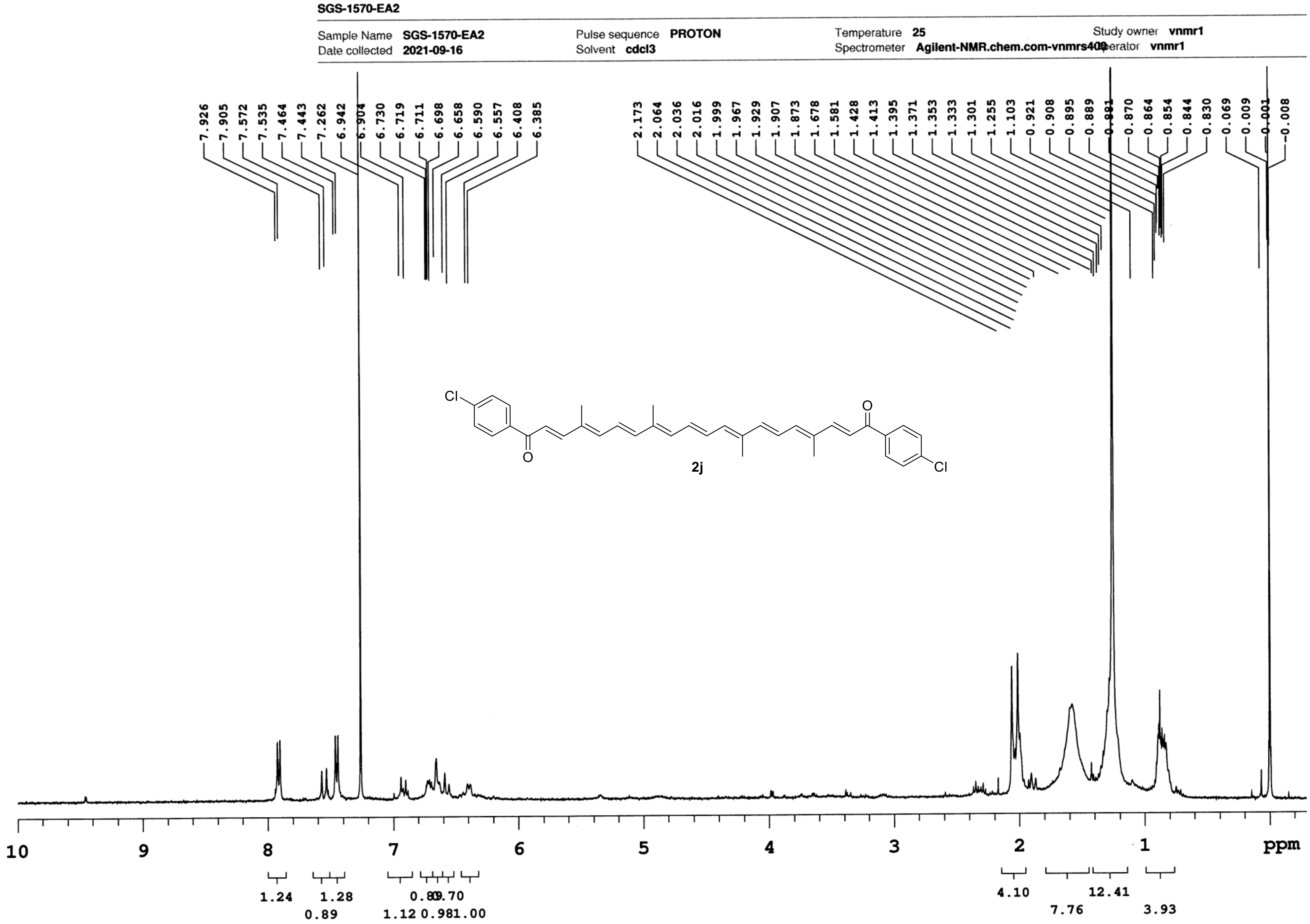
SGS-1570-EA2
Sample Name SGS-1570-EA2
Date collected 2021-09-16
Pulse sequence PROTON
Solvent cdcl3
Temperature 25
Spectrometer Agilent-NMR.chem.com-vnmrs400
Study owner vnmr1
Operator vnmr1
7.926 7.905 7.572 7.535 7.464 7.443 7.262 6.942 6.904 6.730 6.719 6.711 6.698 6.658 6.590 6.557 6.408 6.385
2.173 2.064 2.036 2.016 1.999 1.967 1.929 1.907 1.873 1.678 1.581 1.428 1.413 1.395 1.371 1.353 1.333 1.301 1.255 1.103 0.921 0.908 0.895 0.889 0.881 0.870 0.864 0.854 0.844 0.830 0.069 0.009 0.001 -0.008
Cl
O
2j
10 9 8 7 6 5 4 3 2 1 ppm
1.24 1.28 0.89 0.80 0.70 1.12 0.98 1.00
4.10 7.76 12.41 3.93

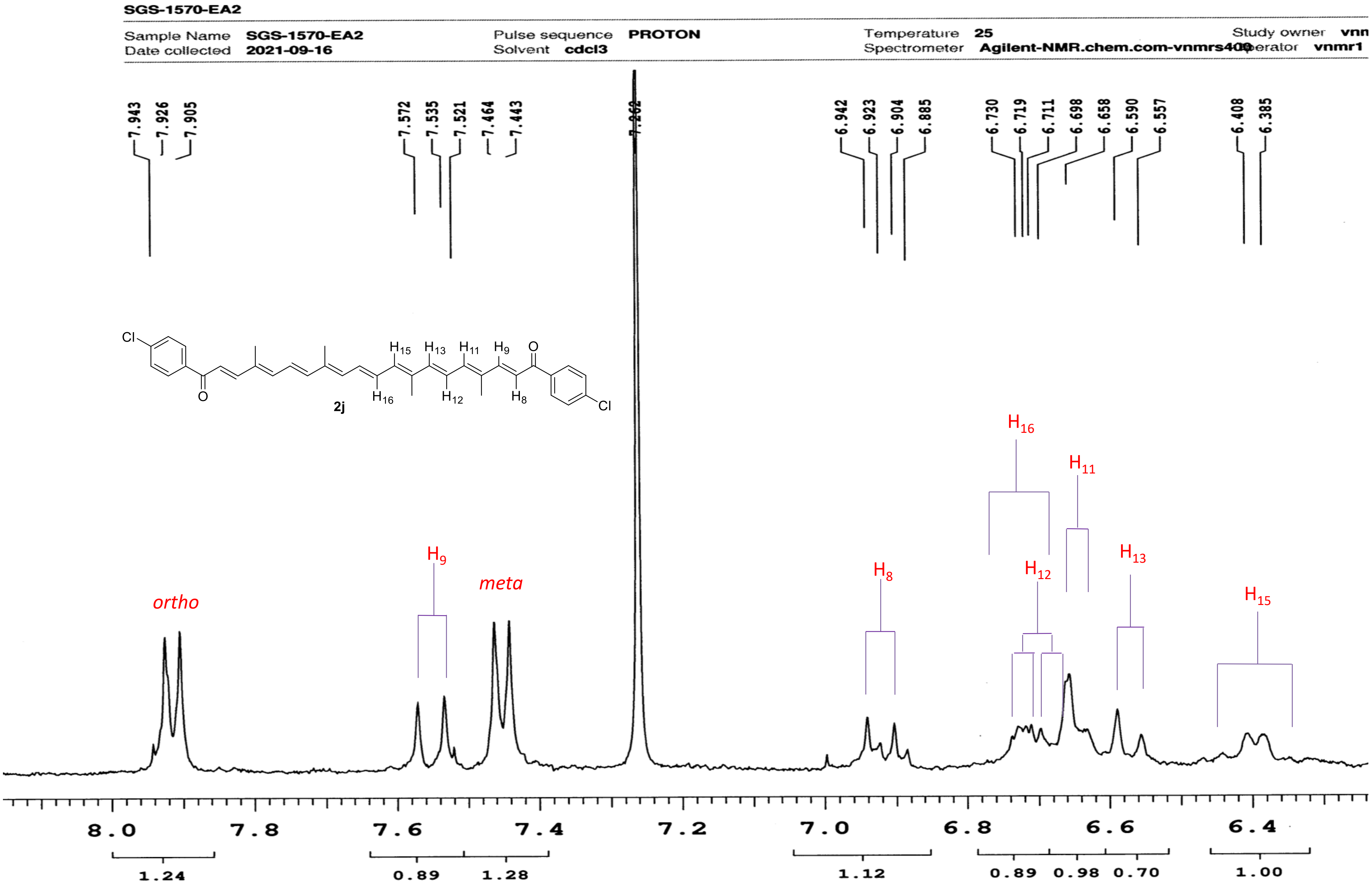

SGS-1570-EA2
Sample Name SGS-1570-EA2
Date collected 2021-09-16
Pulse sequence PROTON
Solvent cdcl3
Temperature 25
Spectrometer Agilent-NMR.chem.com-vnmrs400
Study owner vnn
Operator vnmr1
7.943
7.926
7.905
7.572
7.535
7.521
7.464
7.443
7.262
6.942
6.923
6.904
6.885
6.730
6.719
6.711
6.698
6.658
6.590
6.557
6.408
6.385
2j
H15 H13 H11 H9
H16 H12 H8
ortho
H9
meta
H8
H16
H11
H12
H13
H15
8.0
7.8
7.6
7.4
7.2
7.0
6.8
6.6
6.4
1.24
0.89
1.28
1.12
0.89
0.98
0.70
1.00

SGS-2130-CI-3re

Sample Name **SGS-2130-CI-3re**
Date collected **2022-02-16**
Pulse sequence **PROTON**
Solvent **cdcl3**
Temperature **20**
Spectrometer **Agilent-NMR.chem.com-vnmrs400**
Study owner **vnmr1**
Operator **vnmr1**

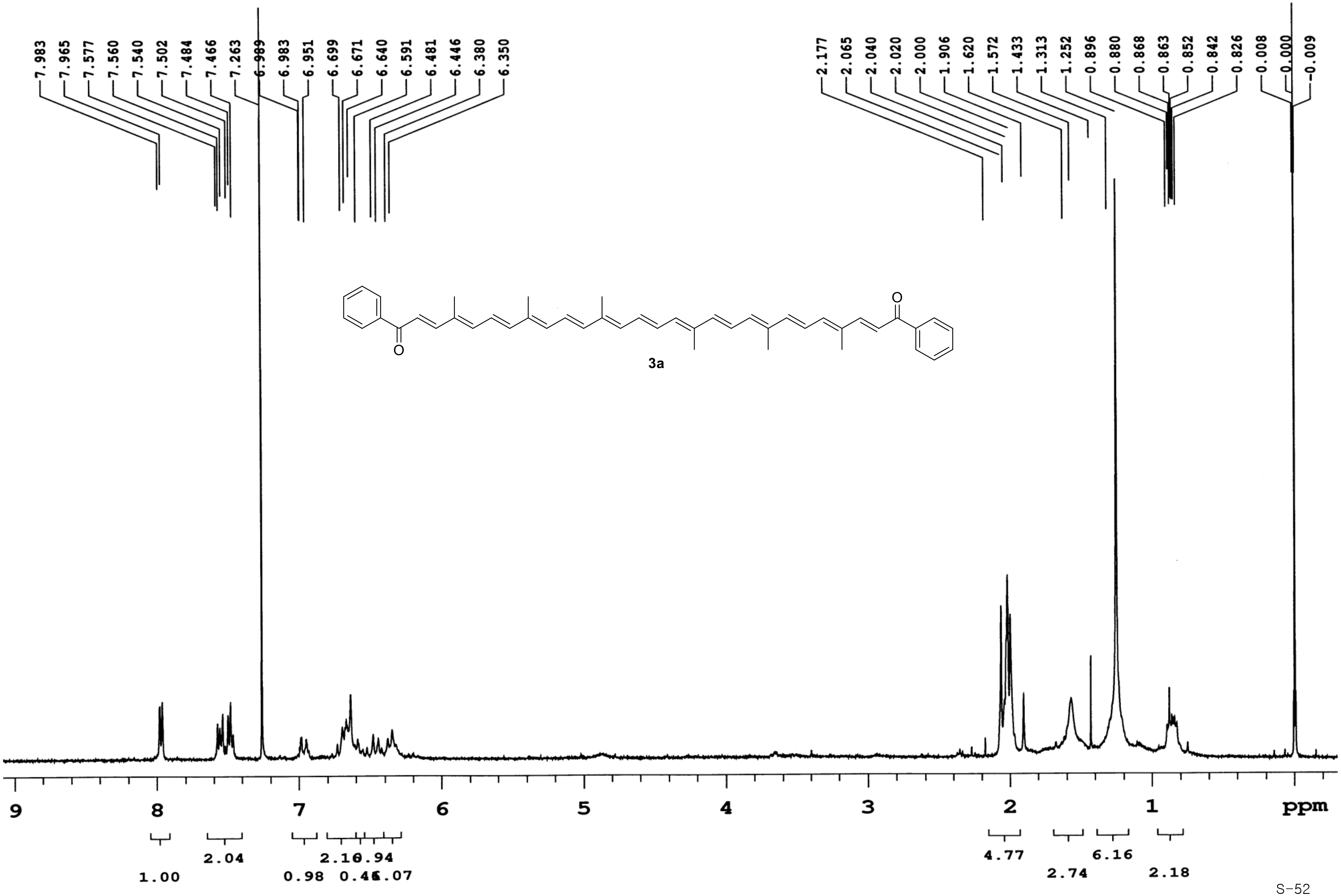

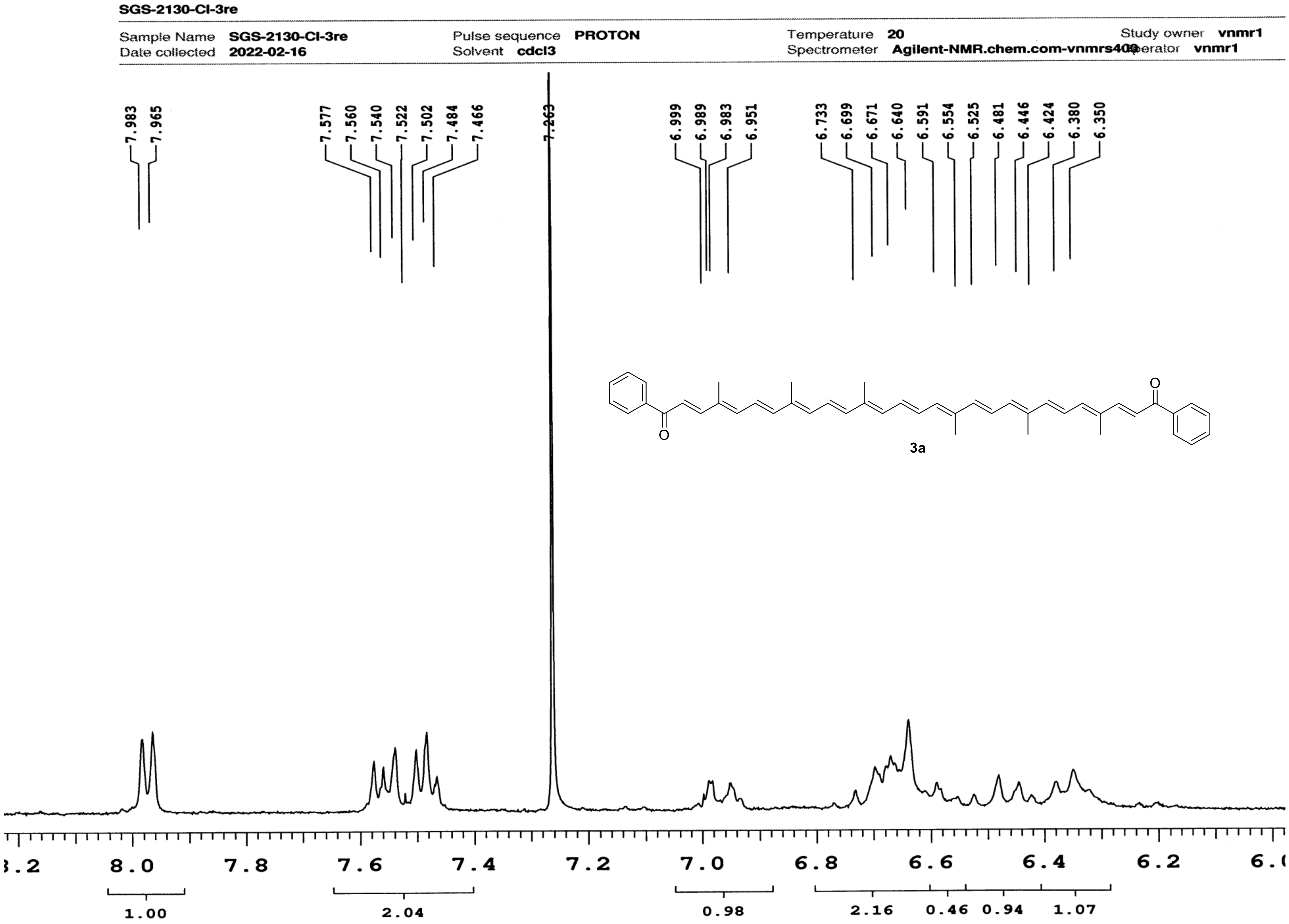
SGS-2130-Cl-3re
Sample Name SGS-2130-Cl-3re
Date collected 2022-02-16
Pulse sequence PROTON
Solvent cdcl3
Temperature 20
Spectrometer Agilent-NMR.chem.com-vnmrs400
Study owner vnmr1
Operator vnmr1
7.983
7.965
7.577
7.560
7.540
7.522
7.502
7.484
7.466
7.263
6.999
6.989
6.983
6.951
6.733
6.699
6.671
6.640
6.591
6.554
6.525
6.481
6.446
6.424
6.380
6.350
3a
8.0
7.8
7.6
7.4
7.2
7.0
6.8
6.6
6.4
6.2
1.00
2.04
0.98
2.16
0.46
0.94
1.07

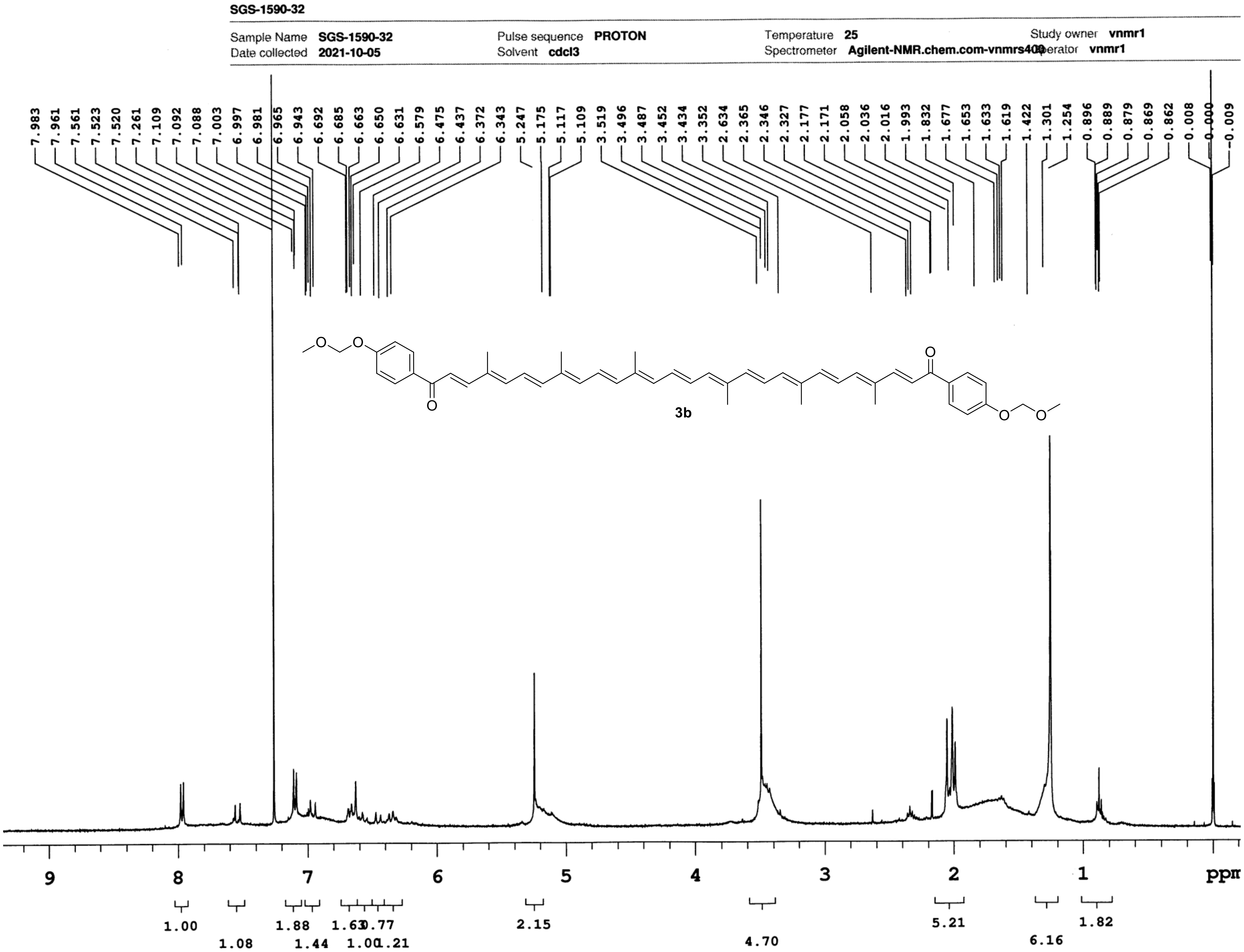
SGS-1590-32
Sample Name SGS-1590-32
Date collected 2021-10-05
Pulse sequence PROTON
Solvent cdcl3
Temperature 25
Spectrometer Agilent-NMR.chem.com-vnmrs400
Study owner vnmr1
Operator vnmr1
7.983 7.961 7.561 7.523 7.520 7.261 7.109 7.092 7.088 7.003 6.997 6.981 6.965 6.943 6.692 6.685 6.663 6.650 6.631 6.579 6.475 6.437 6.372 6.343 5.247 5.175 5.117 5.109 3.519 3.496 3.487 3.452 3.434 3.352 2.634 2.365 2.346 2.327 2.177 2.171 2.058 2.036 2.016 1.993 1.832 1.677 1.653 1.633 1.619 1.422 1.301 1.254 0.896 0.889 0.879 0.869 0.862 0.008 0.000 -0.009
O
O
3b
9 8 7 6 5 4 3 2 1 ppm
1.00 1.08 1.88 1.44 1.63 1.00 0.77 1.21 2.15 4.70 5.21 6.16 1.82

SGS-1590-32

Sample Name **SGS-1590-32** Date collected **2021-10-05** Pulse sequence **PROTON** Solvent **cdcl3** Temperature **25** Spectrometer **Agilent-NMR.chem.com-vnmrs400** Study owner **vnmr1** Operator **vnmr1**

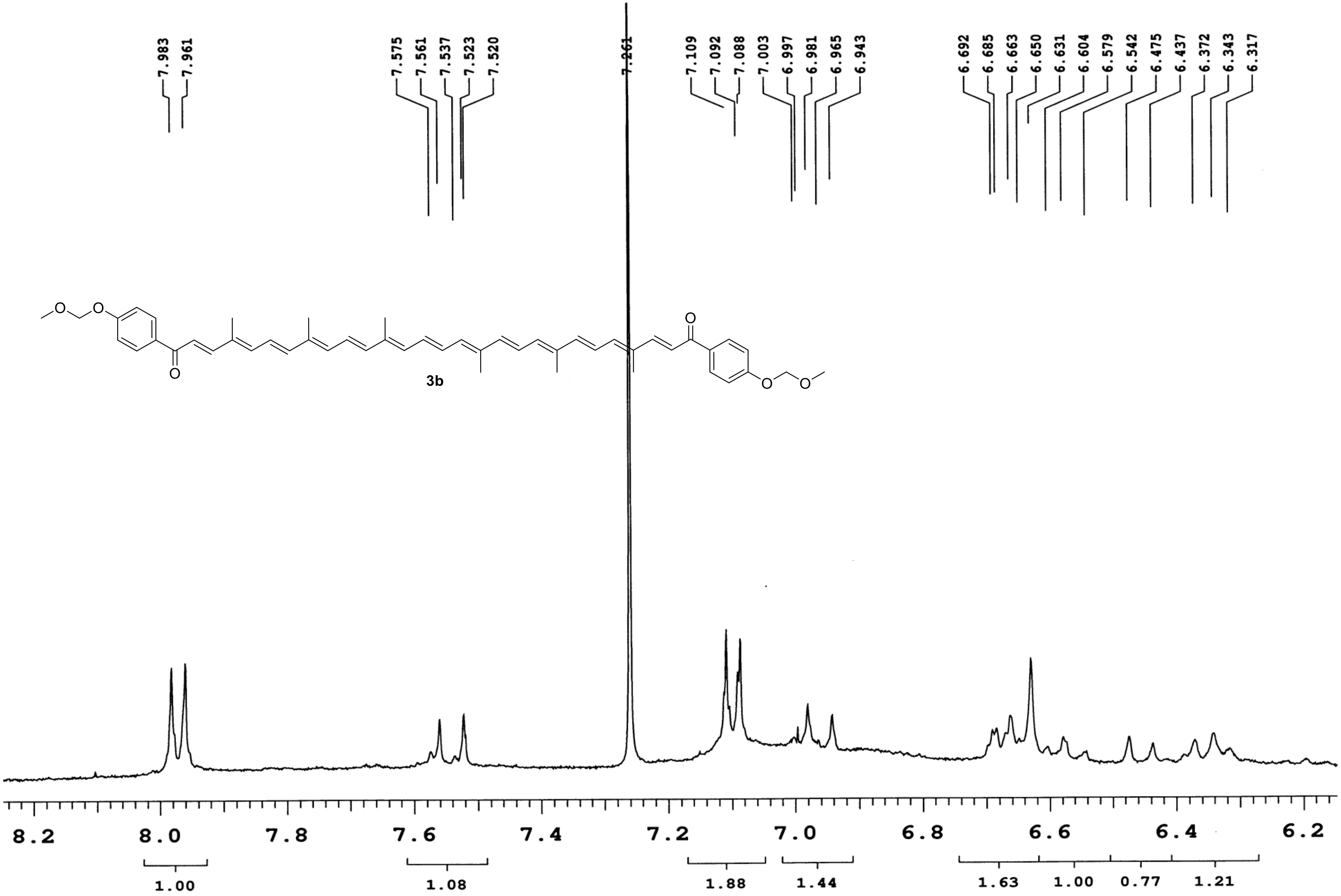

Parameter	Value
Data File Name	D:/ NMR-SHK/ 2201-koo-4-04/ SGS-2039-1re_01/ SGS-2039-1re_PROTON_01.fid/ fid
Title	SGS-2039-1re_PROTON_01
Instrument	vnmrs
Solvent	cdcl3
Probe	5mm_ATB
Acquisition Date	2022-01-04T10:22:29
Spectrometer Frequency	400.04

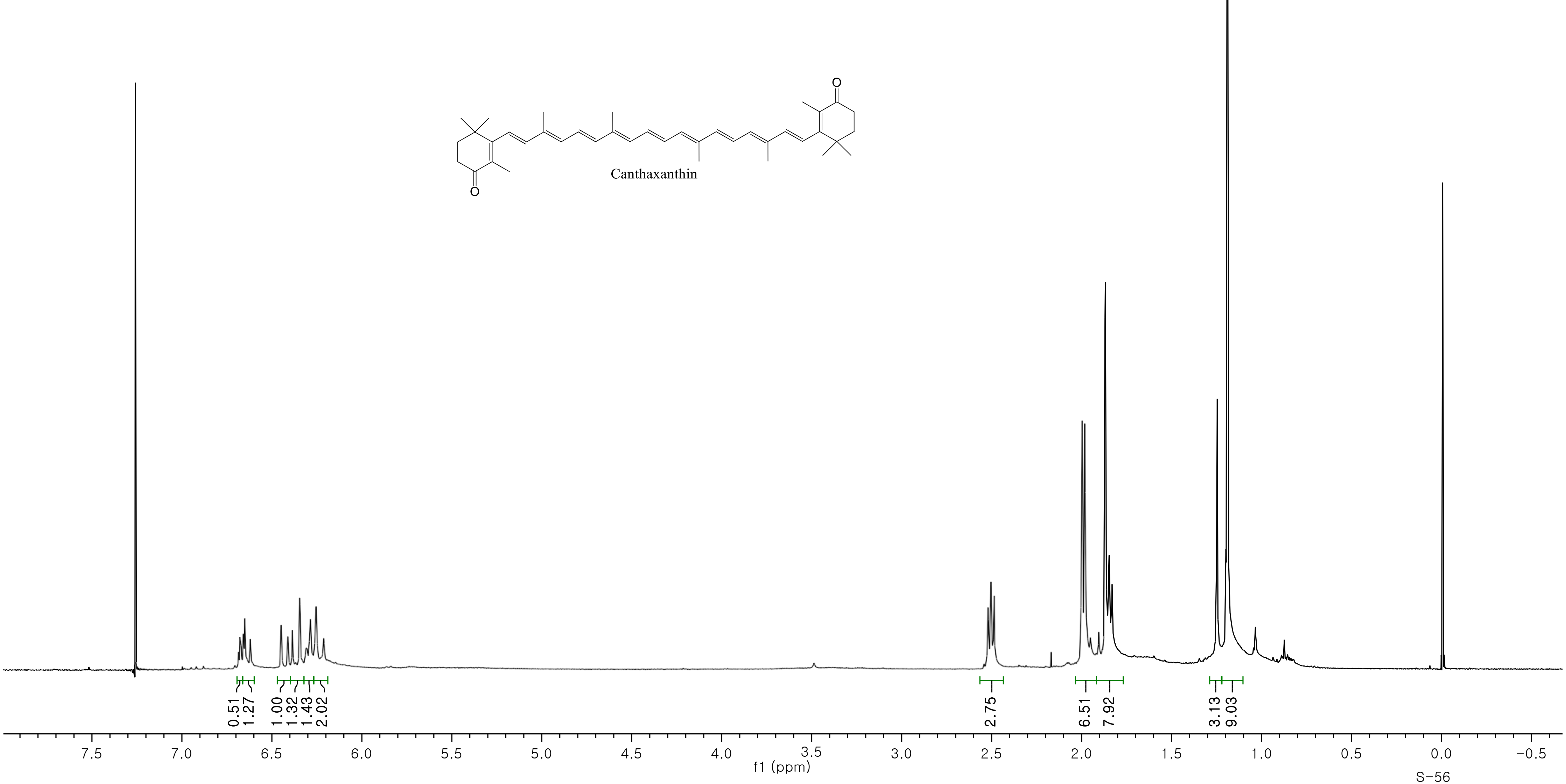

Parameter	Value
Data File Name	D:/ NMR-SHK/ 2201-koo-4-04/ SGS-2039-1re_01/ SGS-2039-1re_PROTON_01.fid/ fid
Title	SGS-2039-1re_PROTON_01
Instrument	vnmrs
Solvent	cdcl3
Probe	5mm_ATB
Acquisition Date	2022-01-04T10:22:29
Spectrometer Frequency	400.04

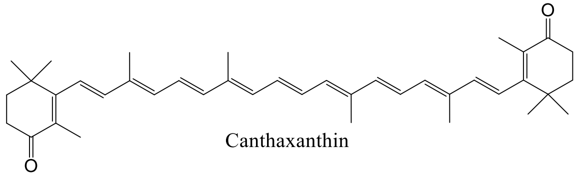

Canthaxanthin

6.60-6.71 (m, 2H)
6.65 (dd, J = 14.8, 11.2 Hz, 2H)

6.42 (d, J = 14.8 Hz, 2H)
6.36 (d, J = 16.1 Hz, 2H)

6.22-6.34 (m, 2H)
6.27 (d, J = 11.2 Hz, 2H)
6.23 (d, J = 16.1 Hz, 2H)

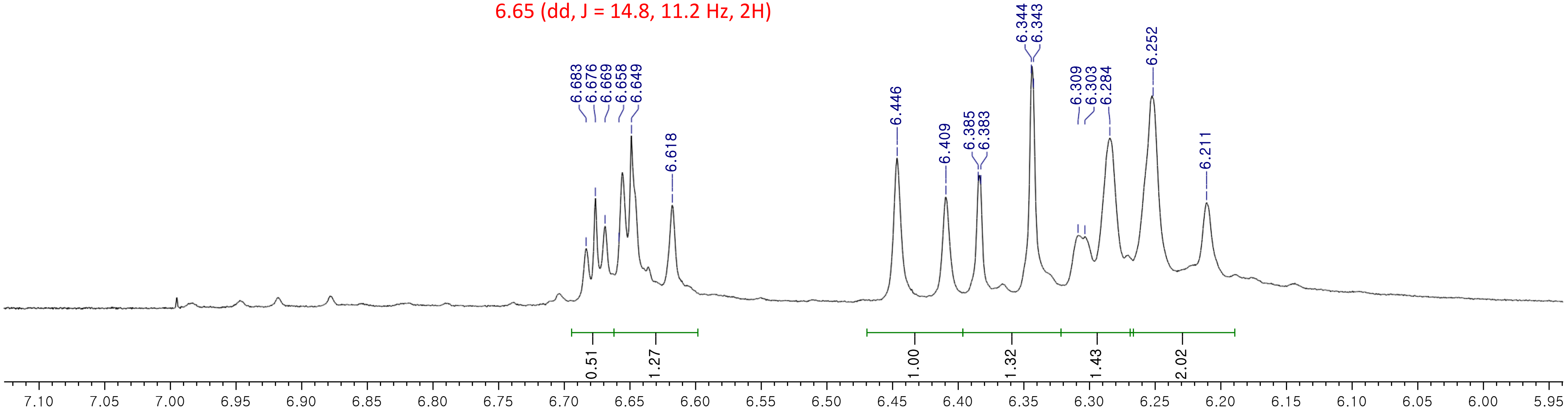

Parameter	Value
Data File Name	D:/ NMR-SHK/ 2201-koo-4-05/ SGS-2039-1re_01/ SGS-2039-1re_CARBON_01.fid/ fid
Title	SGS-2039-1re_CARBON_01
Instrument	vnmrs
Solvent	cdcl3
Probe	5mm_ATB
Acquisition Date	2022-01-05T13:35:39
Spectrometer Frequency	100.60

^{13}C NMR (101 MHz, cdcl$_3$) δ 199.39, 161.30, 141.36, 139.28, 136.67, 134.79, 134.46, 133.58, 130.55, 129.73, 124.68, 124.08, 37.37, 35.74, 34.28, 29.70, 27.69, 13.89, 12.83, 12.62, 0.00.

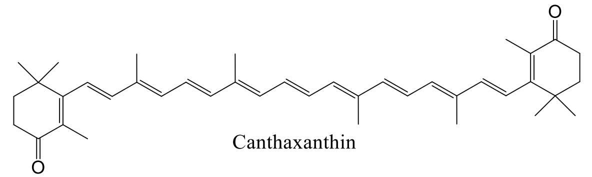

Canthaxanthin

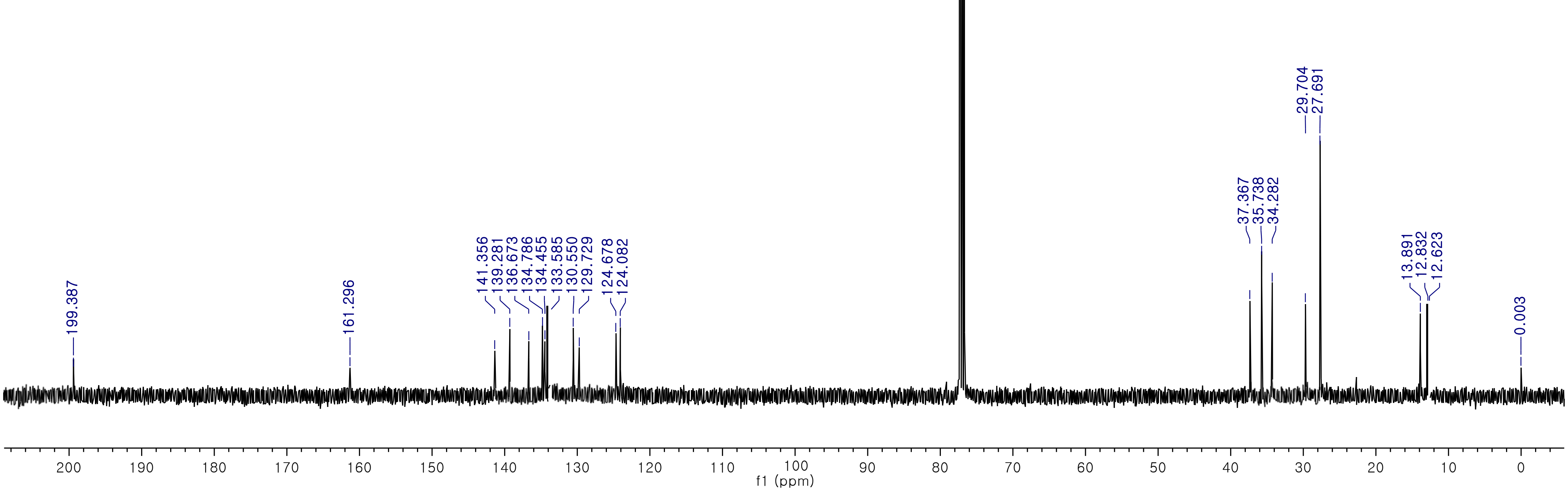

(2) Assignment of *E*-stereochemistry for **4** by N.O.E.

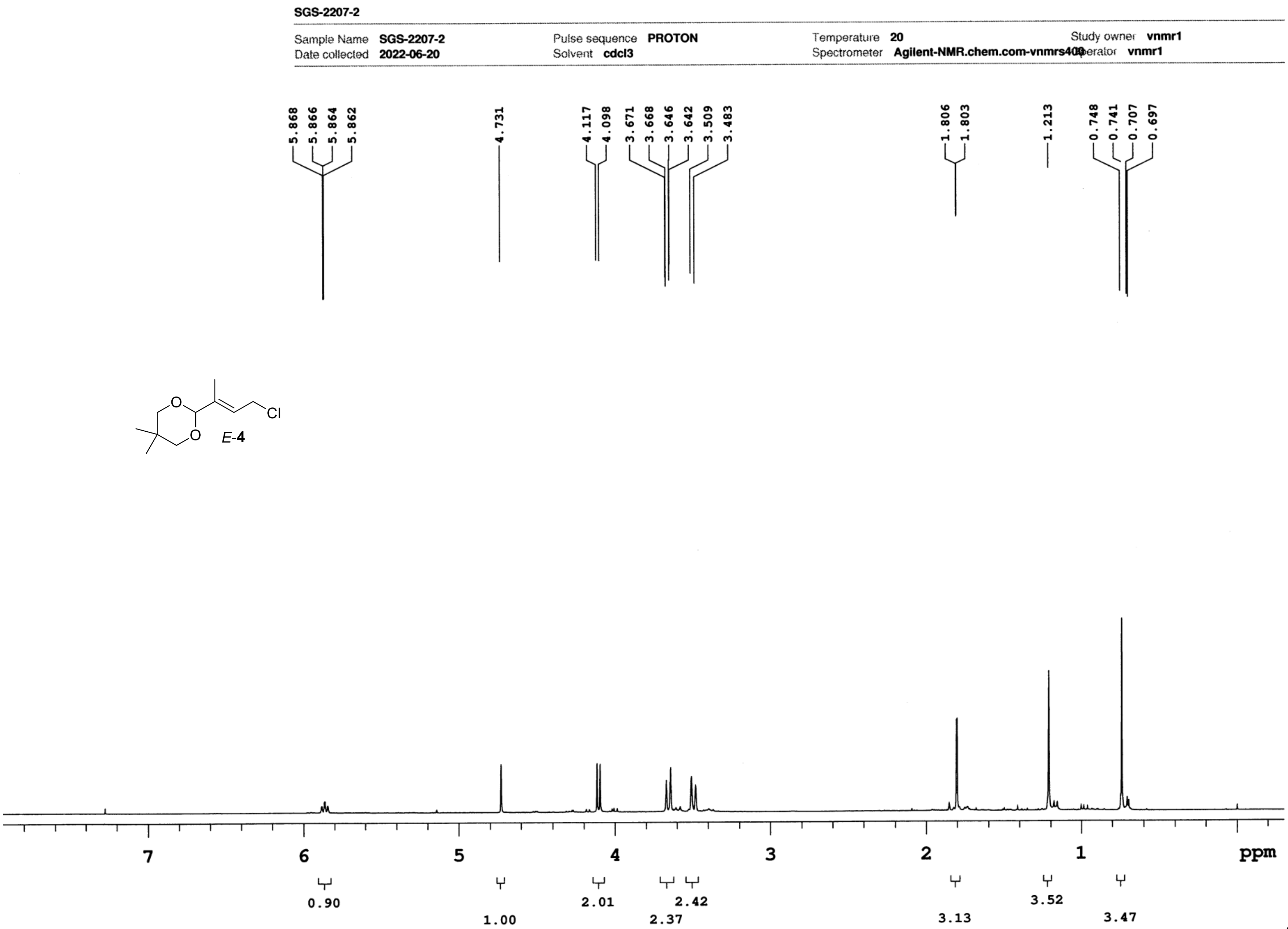

COSY for *E*-**4**

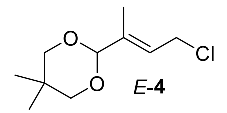
O
O
Cl
E-4

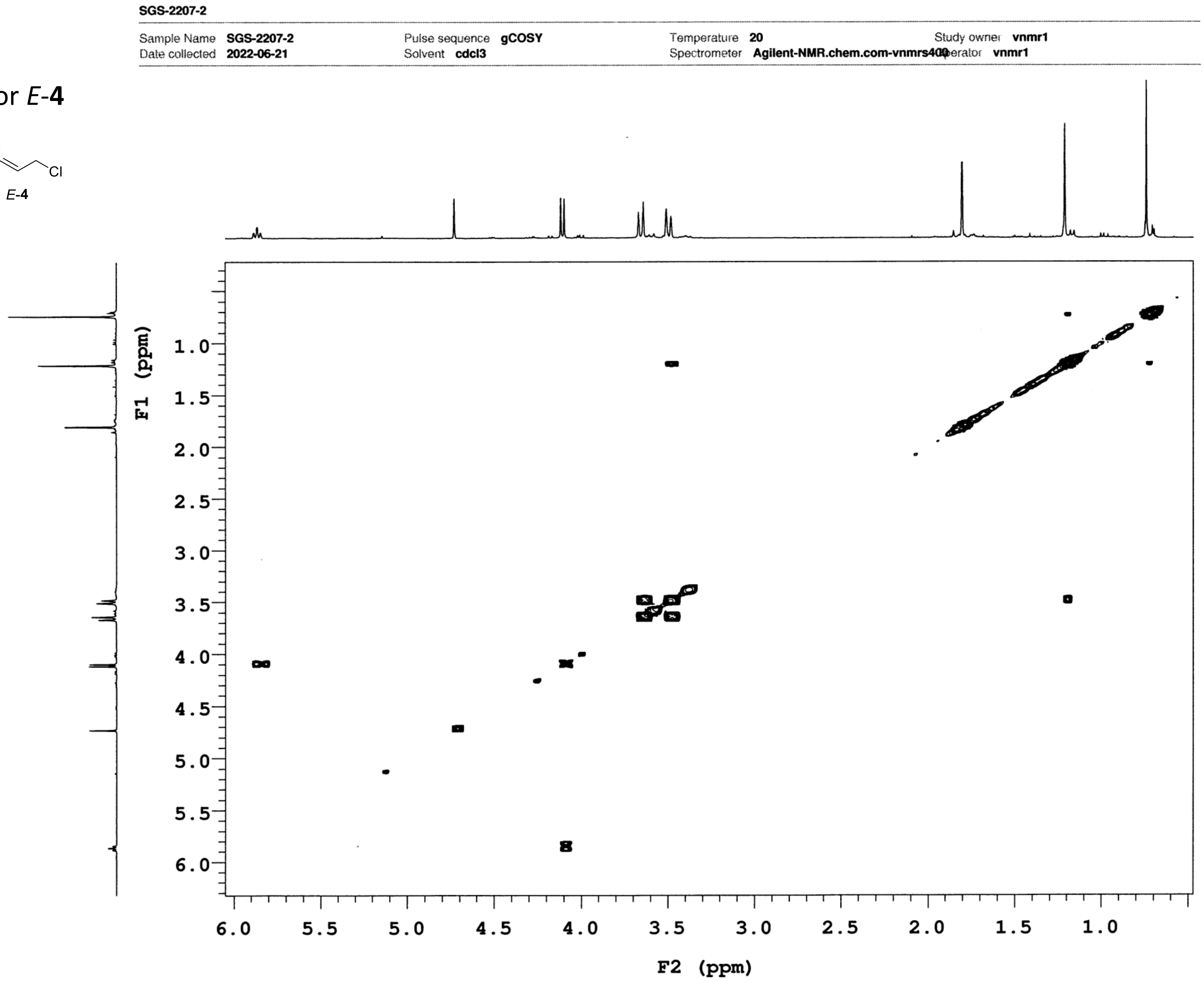
SGS-2207-2
Sample Name SGS-2207-2
Date collected 2022-06-21
Pulse sequence gCOSY
Solvent cdcl3
Temperature 20
Spectrometer Agilent-NMR.chem.com-vnmrs400
Study owner vnmr1
Operator vnmr1
F1 (ppm)
1.0
1.5
2.0
2.5
3.0
3.5
4.0
4.5
5.0
5.5
6.0
6.0
5.5
5.0
4.5
4.0
3.5
3.0
2.5
2.0
1.5
1.0
F2 (ppm)

NOSEY for *E*-**4**

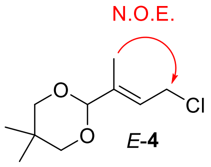
N.O.E.
O
O
Cl
E-4

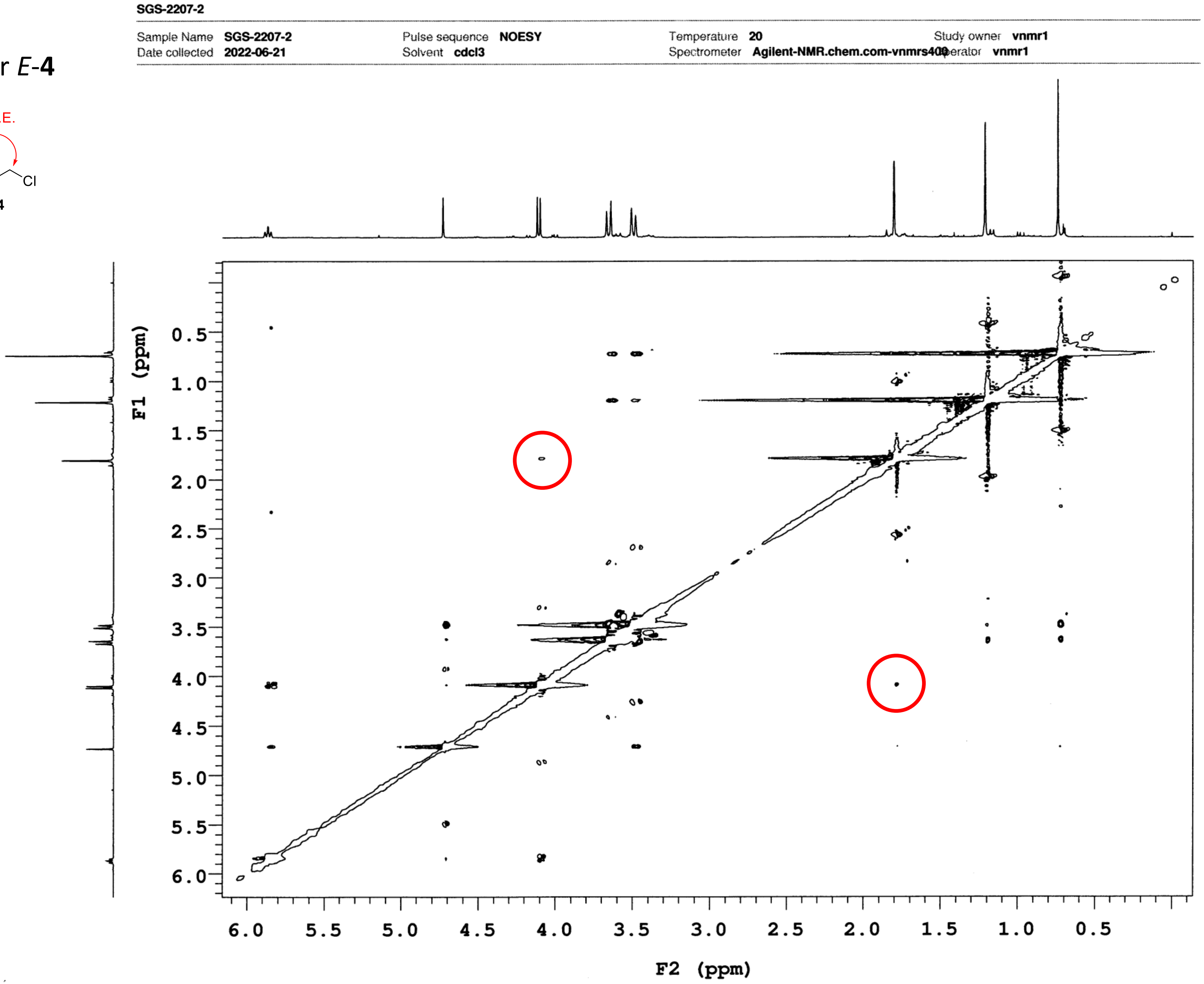
SGS-2207-2
Sample Name SGS-2207-2
Date collected 2022-06-21
Pulse sequence NOESY
Solvent cdcl3
Temperature 20
Spectrometer Agilent-NMR.chem.com-vnmrs400
Study owner vnmr1
Operator vnmr1
F1 (ppm)
0.5
1.0
1.5
2.0
2.5
3.0
3.5
4.0
4.5
5.0
5.5
6.0
F2 (ppm)

1D N.O.E. of *E*-**4**

Sample Name:
SGS-2207-2
Data Collected on:
Agilent-NMR.chem.com-vnmrs400
Archive directory:
/home/vnmr1/vnmrsys/data/2206-koo-4
Sample directory:
SGS-2207-2_01
FidFile: SGS-2207-2_PROTON_01

Pulse Sequence: PROTON (s2pul)
Solvent: cdcl3
Data collected on: Jun 22 2022

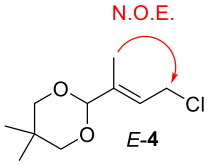

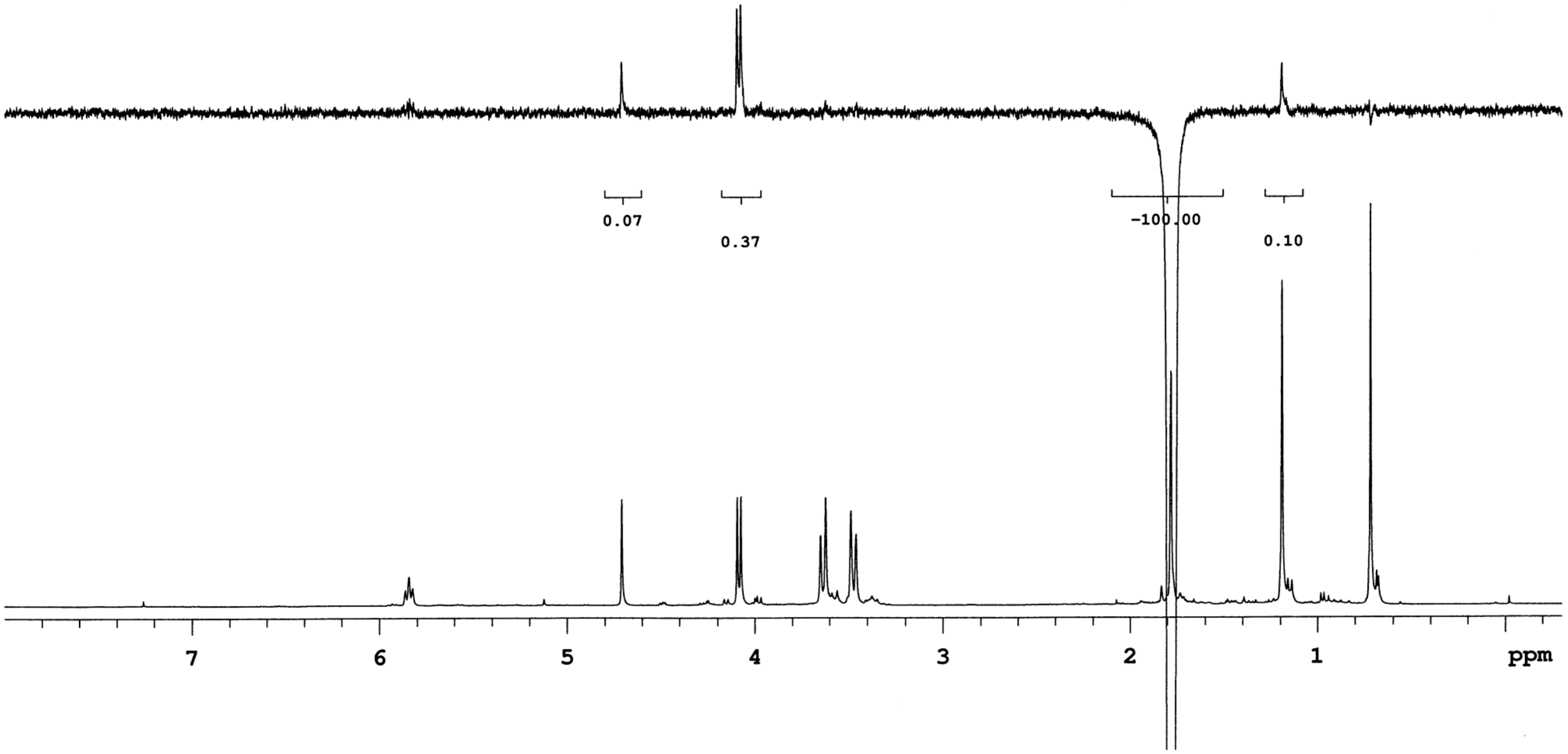

(3) Peak assignment for **2b** by COSY, HMBC, and DEPT

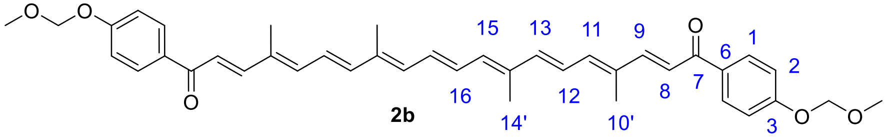

2b

Numbering	^{1}H NMR		^{13}C NMR	
	Shift (ppm)	Multiplicity (Hz)	Shift (ppm)	DEPT
**1**	7,97	d (8.8)	130.5	CH
**2**	7.10	d (8.8)	115.7	CH
**3**	-	-	160.7	C
**3-OCH_2O**	5.25	s	94.1	CH_2
**3-OMe**	3.50	s	56.2	CH_3
**6**	-	-	132.5	C
**7**	-	-	188.9	C
**8**	6.97	d (15.2)	120.2	CH
**9**	7.54	d (15.2)	148.6	CH
**10**	-	-	134.3	C
**10'(Me)**	2.06	s	12.9	CH_3
**11**	6.62	d (11.2)	140.8	CH
**12**	6.69	dd (14.4, 11.2)	124.7	CH
**13**	6.55	d (14.4)	142.0	CH
**14**	-	-	137.0	C
**14'(Me)**	2.01	s	12.8	CH_3
**15**	6.33-6.43	m	135.1	CH
**16**	6.66-6.76	m	131.3	CH

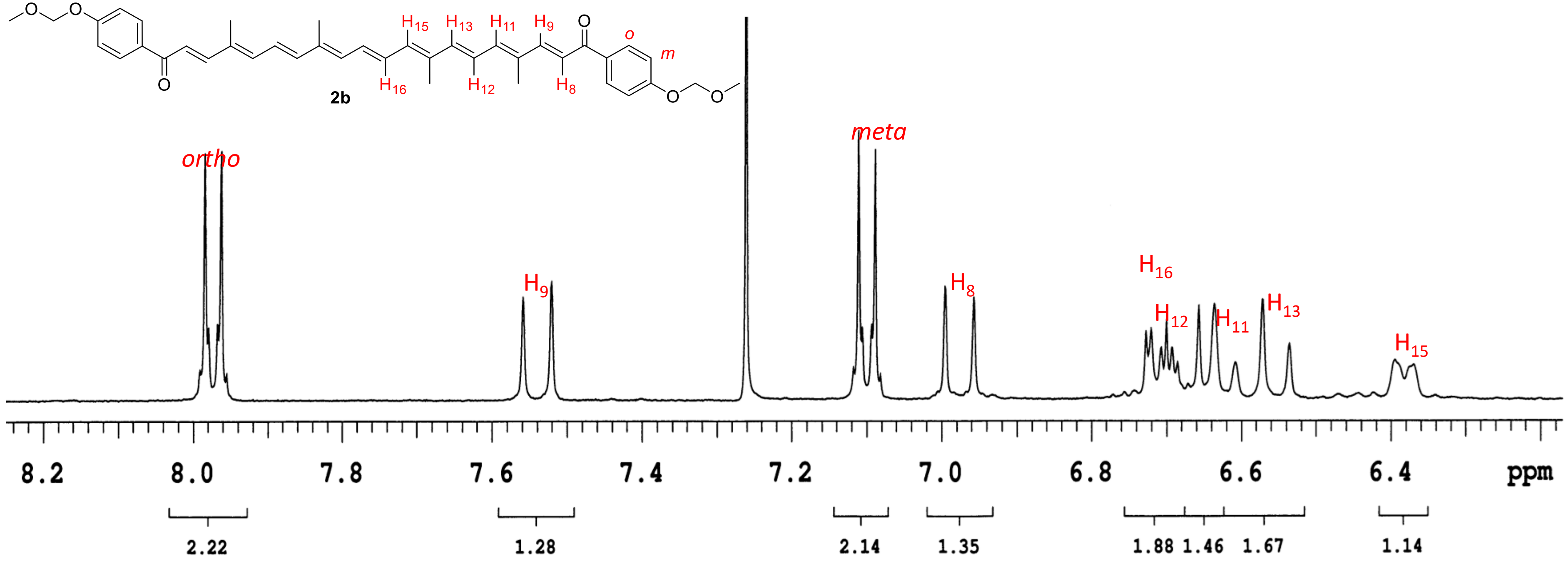
2b
H15 H13 H11 H9
H16 H12 H8
o
m
ortho
meta
H9
H8
H16
H12
H11
H13
H15
8.2 8.0 7.8 7.6 7.4 7.2 7.0 6.8 6.6 6.4 ppm
2.22
1.28
2.14
1.35
1.88 1.46 1.67
1.14

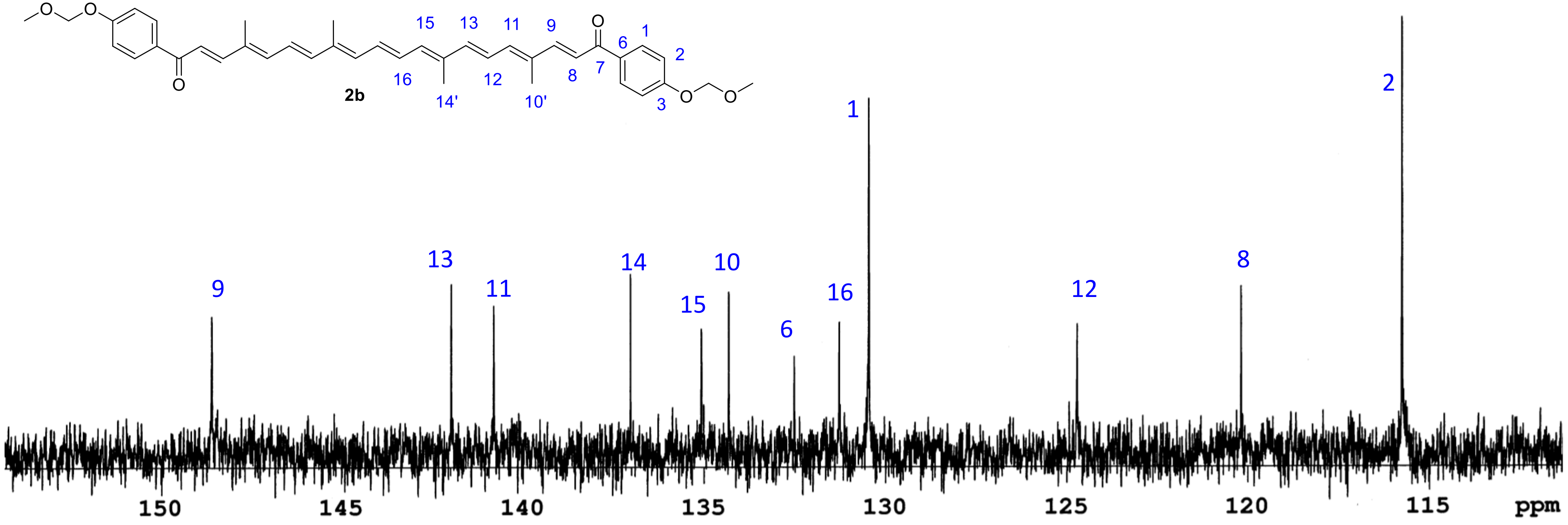
2b
15 13 11 9
16 12 8
14' 10'
1 2 3 6 7
9
13
11
14
15
10
6
16
1
12
8
2
150 145 140 135 130 125 120 115 ppm

COSY for **2b**

Sample Name:
SGS-1586-3re-2D_NMR
Data Collected on:
Agilent-NMR.chem.com-vnmrs400
Archive directory:
/home/vnmr1/vnmrsys/data/2206-koo-4-25
Sample directory:
SGS-1586-3re-2D_NMR_01
FidFile: SGS-1586-3re-2D_NMR_gCOSY_01

Pulse Sequence: gCOSY
Solvent: cdcl3
Data collected on: Jun 25 2022

Sample #21, Operator: vnmr1

Relax. delay 1.000 sec
Acq. time 0.150 sec
Width 3930.8 Hz
2D Width 3930.8 Hz
16 repetitions
512 increments
OBSERVE H1, 400.0405310 MHz
DATA PROCESSING
Sq. sine bell 0.075 sec
F1 DATA PROCESSING
Sq. sine bell 0.080 sec
FT size 4096 x 4096
Total time 2 hr, 50 min

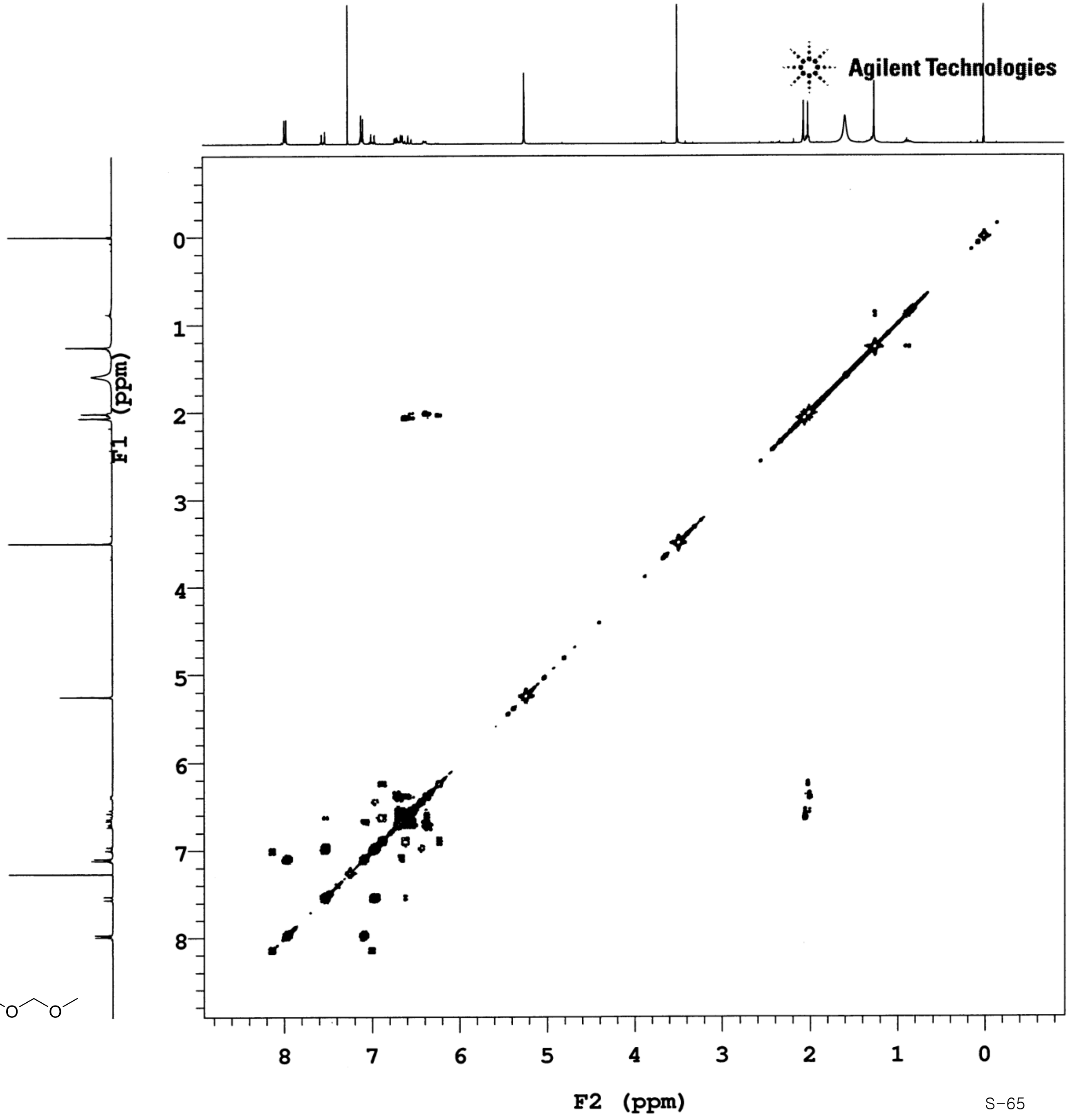

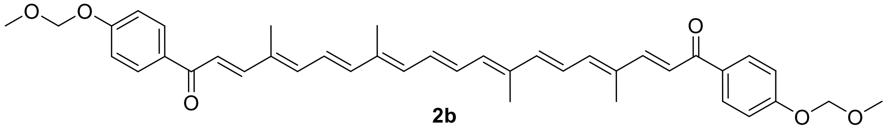

2b

COSY for **2b**

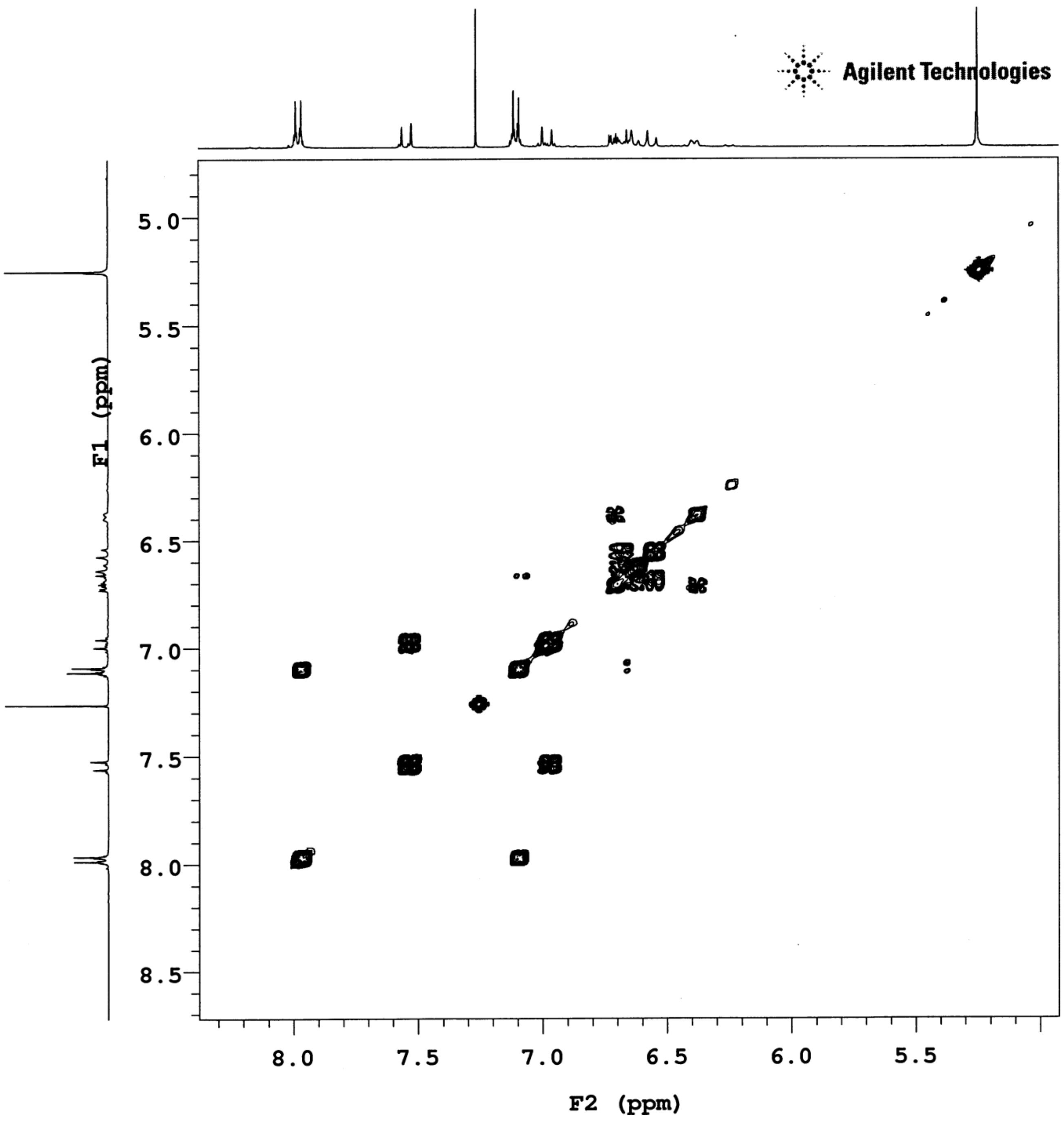

Agilent Technologies
F1 (ppm)
5.0
5.5
6.0
6.5
7.0
7.5
8.0
8.5
8.0
7.5
7.0
6.5
6.0
5.5
F2 (ppm)

COSY for **2b**

Sample Name:
SGS-1586-3re-2D_NMR
Data Collected on:
Agilent-NMR.chem.com-vnmrs400
Archive directory:
/home/vnmr1/vnmrsys/data/2206-koo-4-25
Sample directory:
SGS-1586-3re-2D_NMR_01
FidFile: SGS-1586-3re-2D_NMR_gCOSY_01

Pulse Sequence: gCOSY
Solvent: cdcl3
Data collected on: Jun 25 2022

Sample #21, Operator: vnmr1

Relax. delay 1.000 sec
Acq. time 0.150 sec
Width 3930.8 Hz
2D Width 3930.8 Hz
16 repetitions
512 increments
OBSERVE H1, 400.0405310 MHz
DATA PROCESSING
Sq. sine bell 0.075 sec
F1 DATA PROCESSING
Sq. sine bell 0.080 sec
FT size 4096 x 4096
Total time 2 hr, 50 min

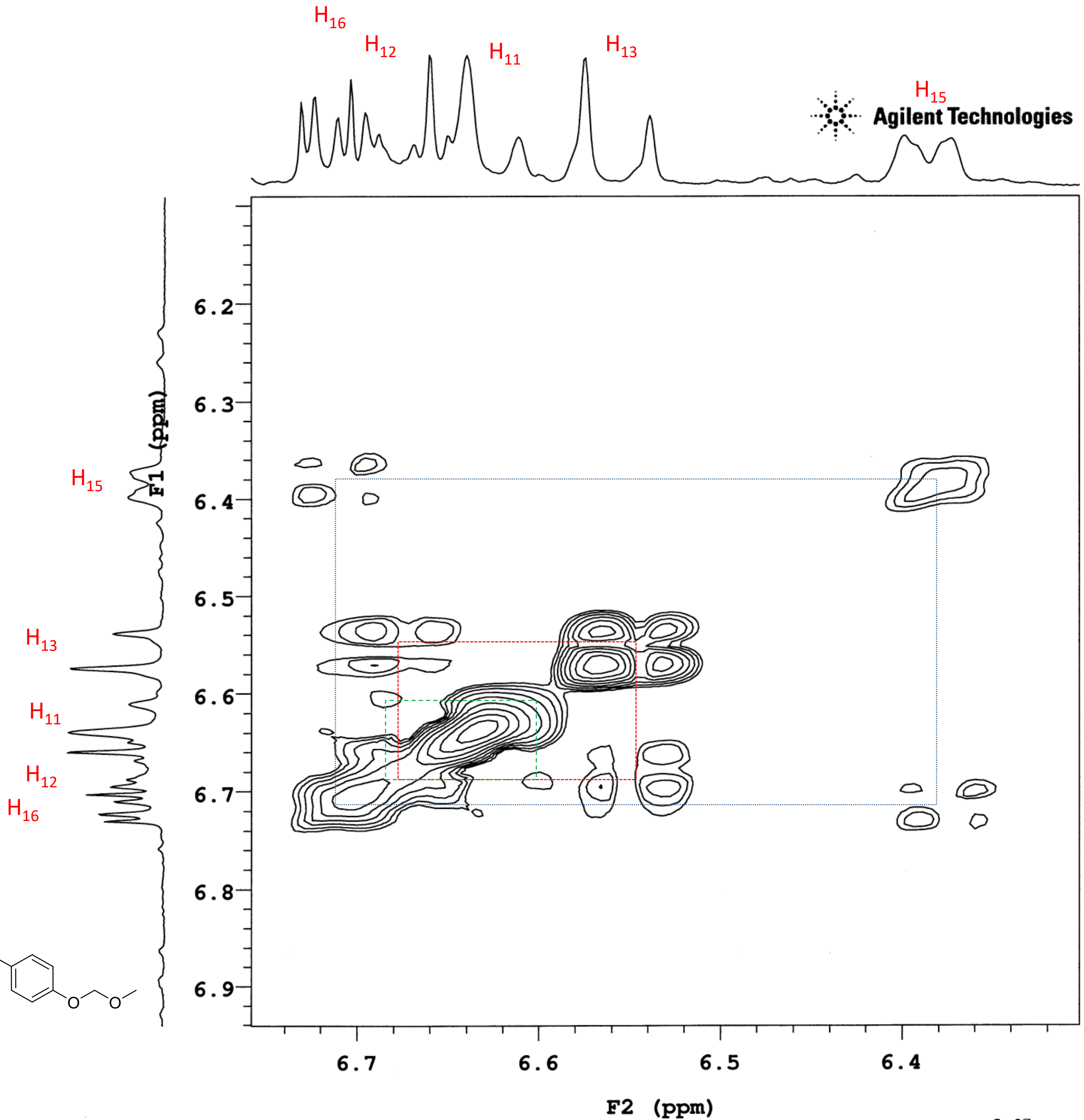

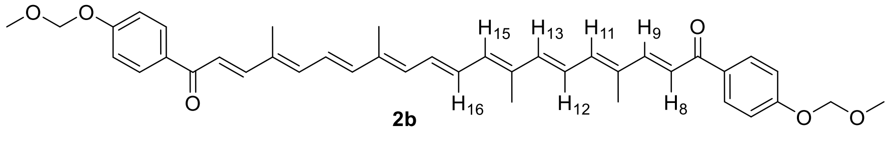

2b

NOSEY for **2b**

Sample Name:
SGS-1586-3re-2D_NMR
Data Collected on:
Agilent-NMR.chem.com-vnmrs400
Archive directory:
/home/vnmr1/vnmrsys/data/2206-koo-4-25
Sample directory:
SGS-1586-3re-2D_NMR_01
FidFile: SGS-1586-3re-2D_NMR_NOESY_01

Pulse Sequence: NOESY
Solvent: cdcl3
Data collected on: Jun 25 2022

Sample #21, Operator: vnmr1

Relax. delay 1.000 sec
Acq. time 0.150 sec
Width 3930.8 Hz
2D Width 3930.8 Hz
16 repetitions
2 x 512 increments
OBSERVE H1, 400.0405310 MHz
DATA PROCESSING
Gauss apodization 0.069 sec
F1 DATA PROCESSING
Gauss apodization 0.074 sec
FT size 4096 x 4096
Total time 6 hr, 28 min

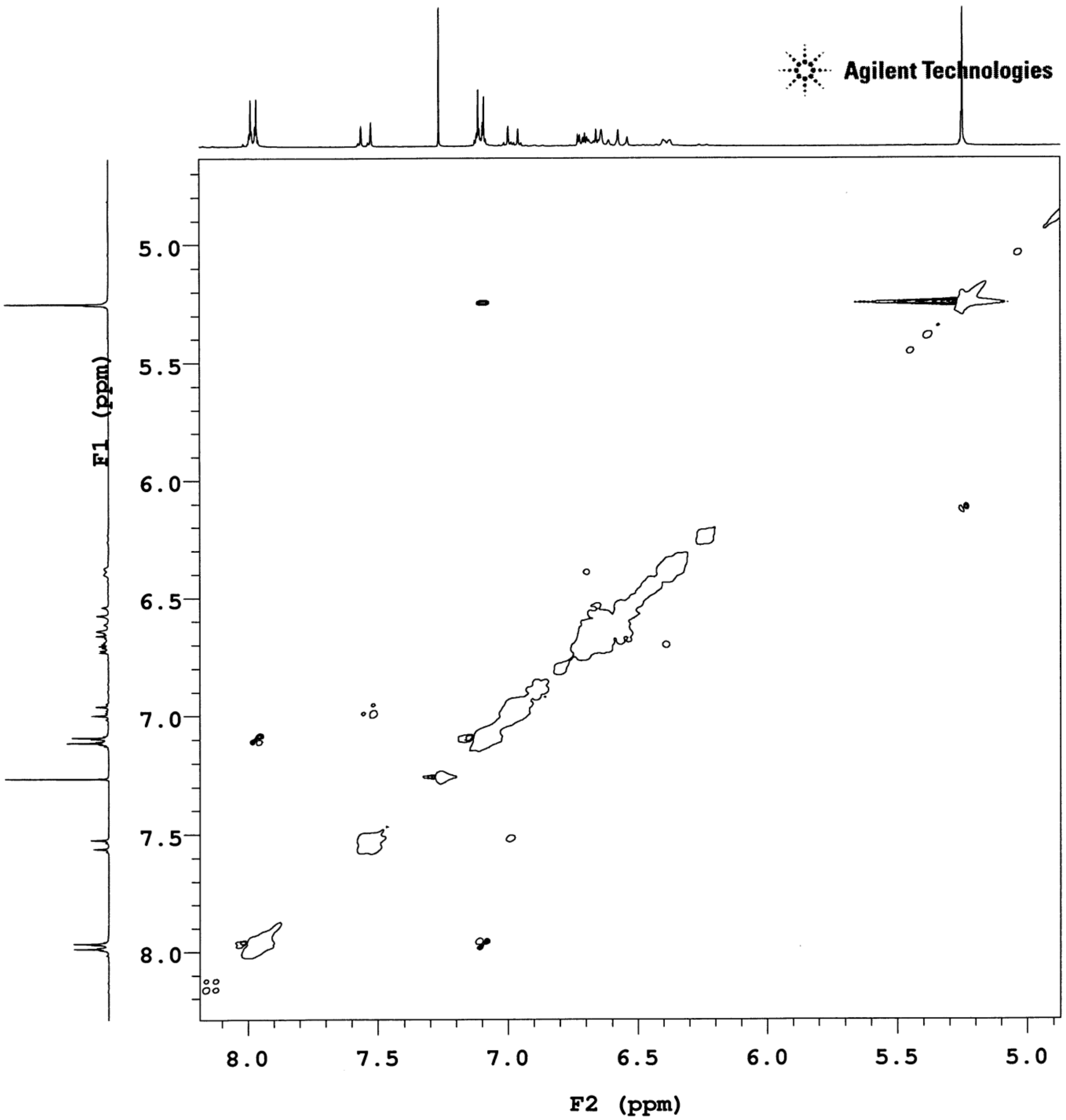

Comparison of COSY and NOESY for **2b**

COSY

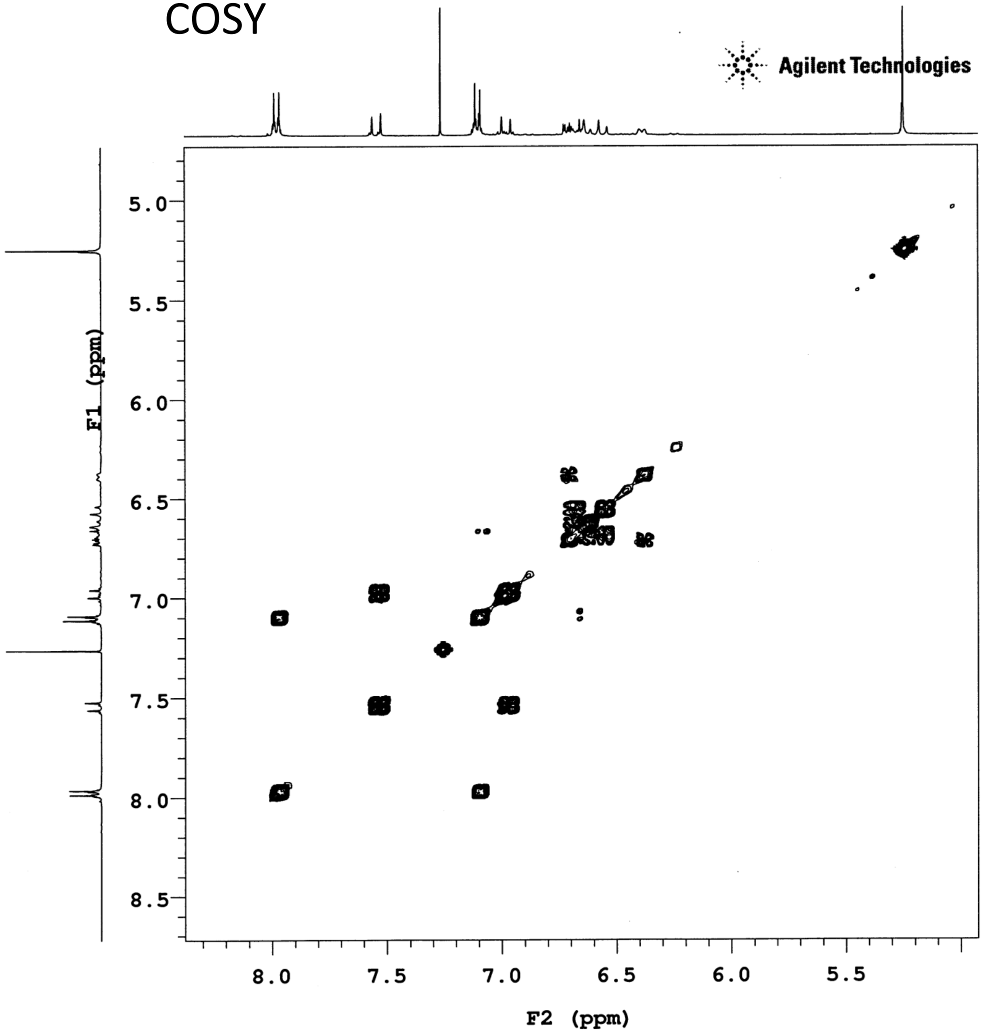

NOESY

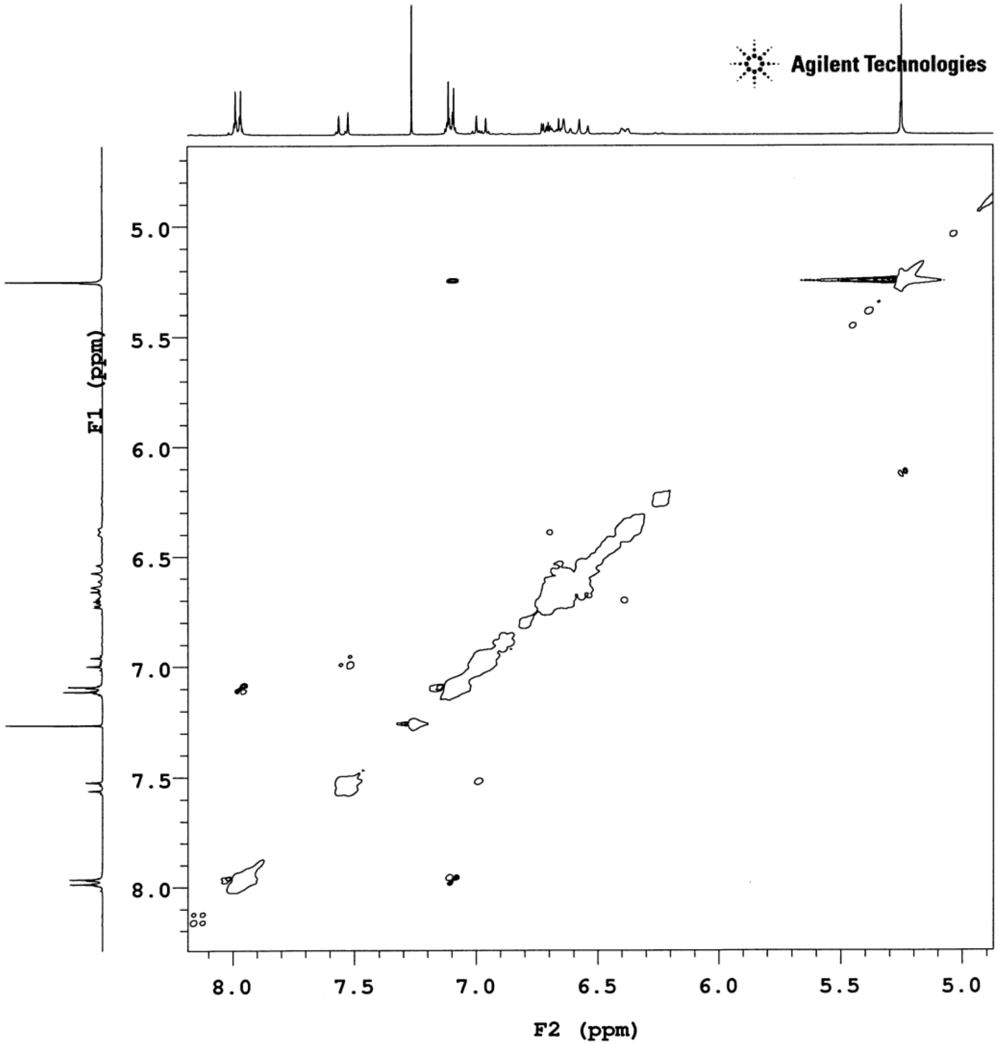

No further correlation in NOESY spectra (compared with COSY) confirmed all-*E* stereochemistry for **2b**

HMBC for **2b**

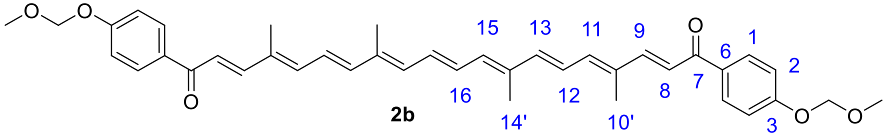

Agilent Technologies

Sample Name:
SGS-1586-3re-2D_NMR
Data Collected on:
Agilent-NMR.chem.com-vnmrs400
Archive directory:
/home/vnmr1/vnmrsys/data/2206-koo-4-25
Sample directory:
SGS-1586-3re-2D_NMR_01
FidFile: SGS-1586-3re-2D_NMR_gHMBCAD_01

Pulse Sequence: gHMBCAD
Solvent: cdcl3
Data collected on: Jun 25 2022

Sample #21, Operator: vnmr1

Relax. delay 1.000 sec
Acq. time 0.150 sec
Width 6410.3 Hz
2D Width 24140.0 Hz
16 repetitions
2 x 512 increments
OBSERVE H1, 400.0405310 MHz
DATA PROCESSING
Sq. sine bell 0.075 sec
F1 DATA PROCESSING
Gauss apodization 0.020 sec
FT size 2048 x 4096
Total time 5 hr, 39 min

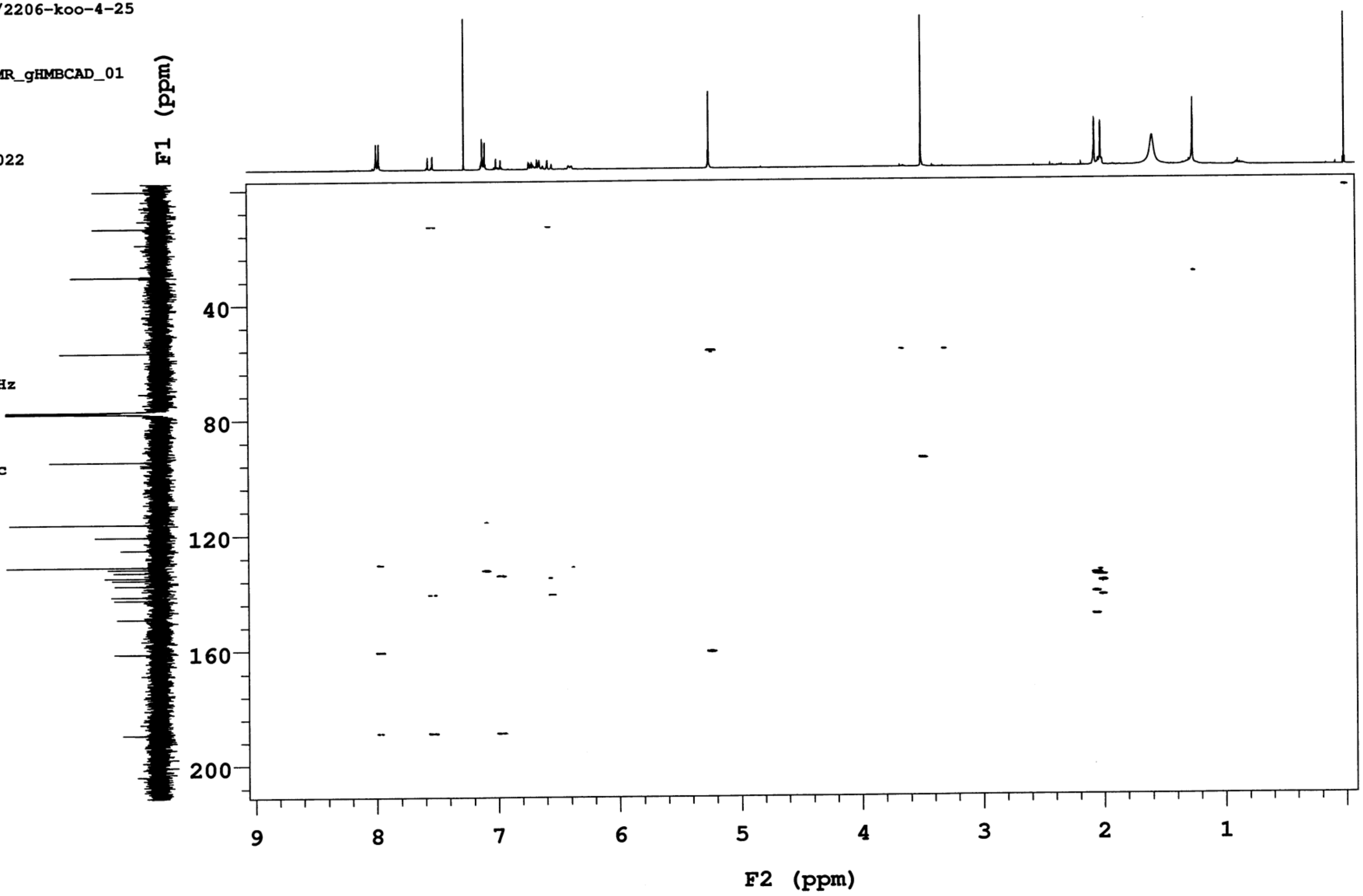

HMBC for **2b**

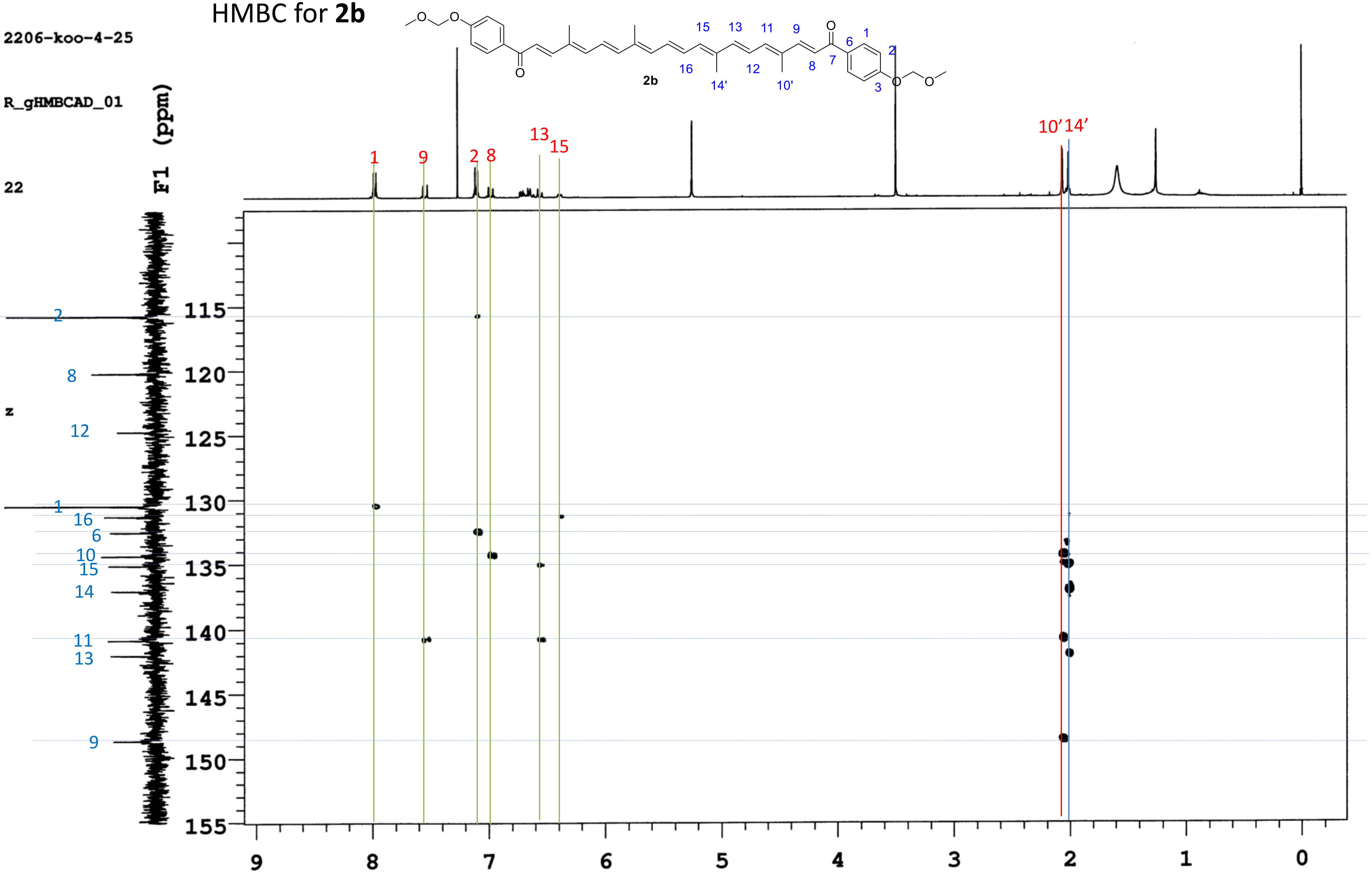
2206-koo-4-25
R_gHMBCAD_01
22
F1 (ppm)
2b
15 13 11 9 6 1 2
16 12 8 7 3
14' 10'
1 9 2 8 13 15 10' 14'
2 8 12 1 16 6 10 15 14 11 13 9
115 120 125 130 135 140 145 150 155
9 8 7 6 5 4 3 2 1 0

HMBC for **2b**

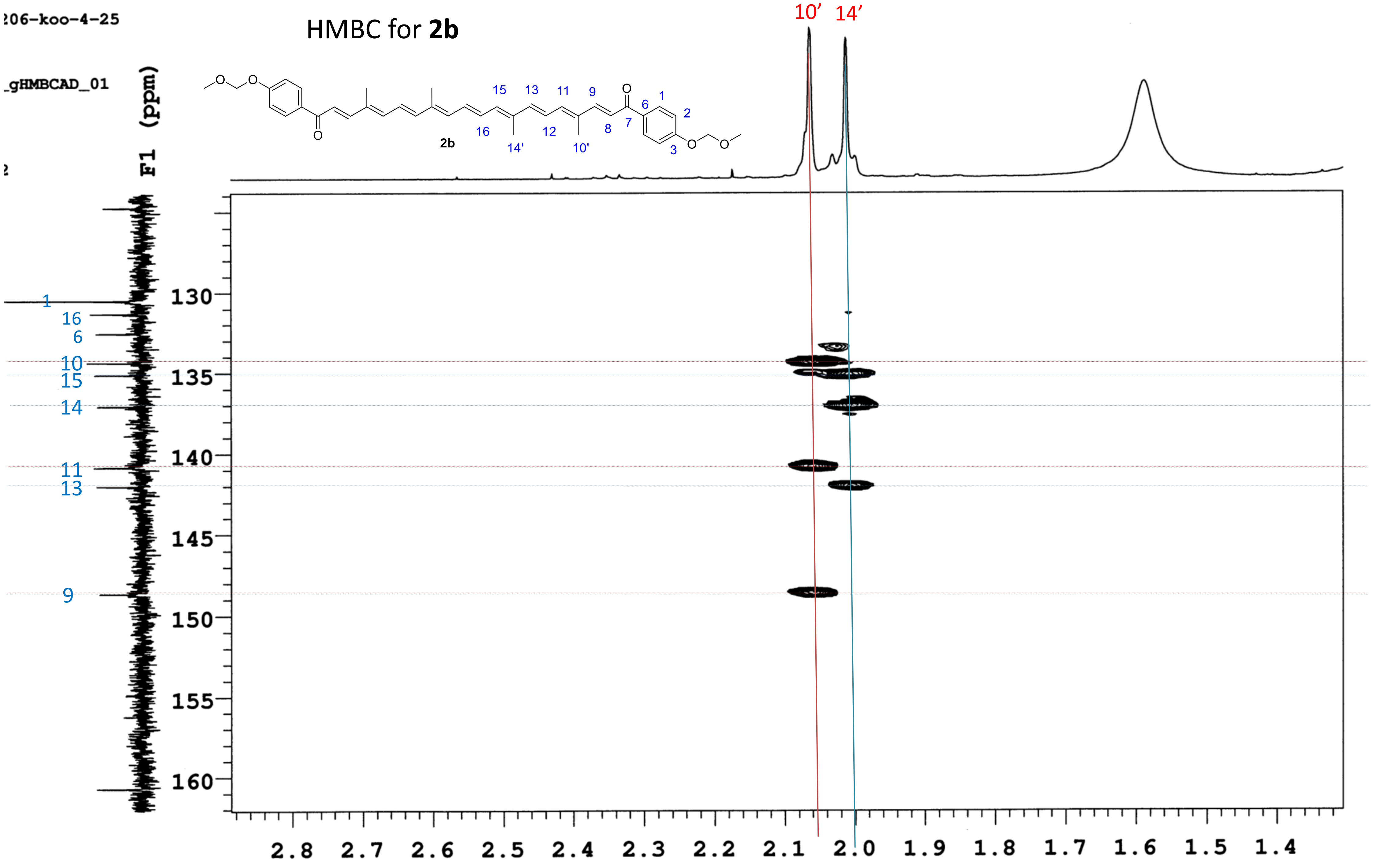

206-koo-4-25
_gHMBCAD_01
F1 (ppm)
2b
10'
14'
1
16
6
10
15
14
11
13
9
15
13
11
9
16
12
8
7
6
1
2
3
130
135
140
145
150
155
160
2.8
2.7
2.6
2.5
2.4
2.3
2.2
2.1
2.0
1.9
1.8
1.7
1.6
1.5
1.4

HMBC for **2b**

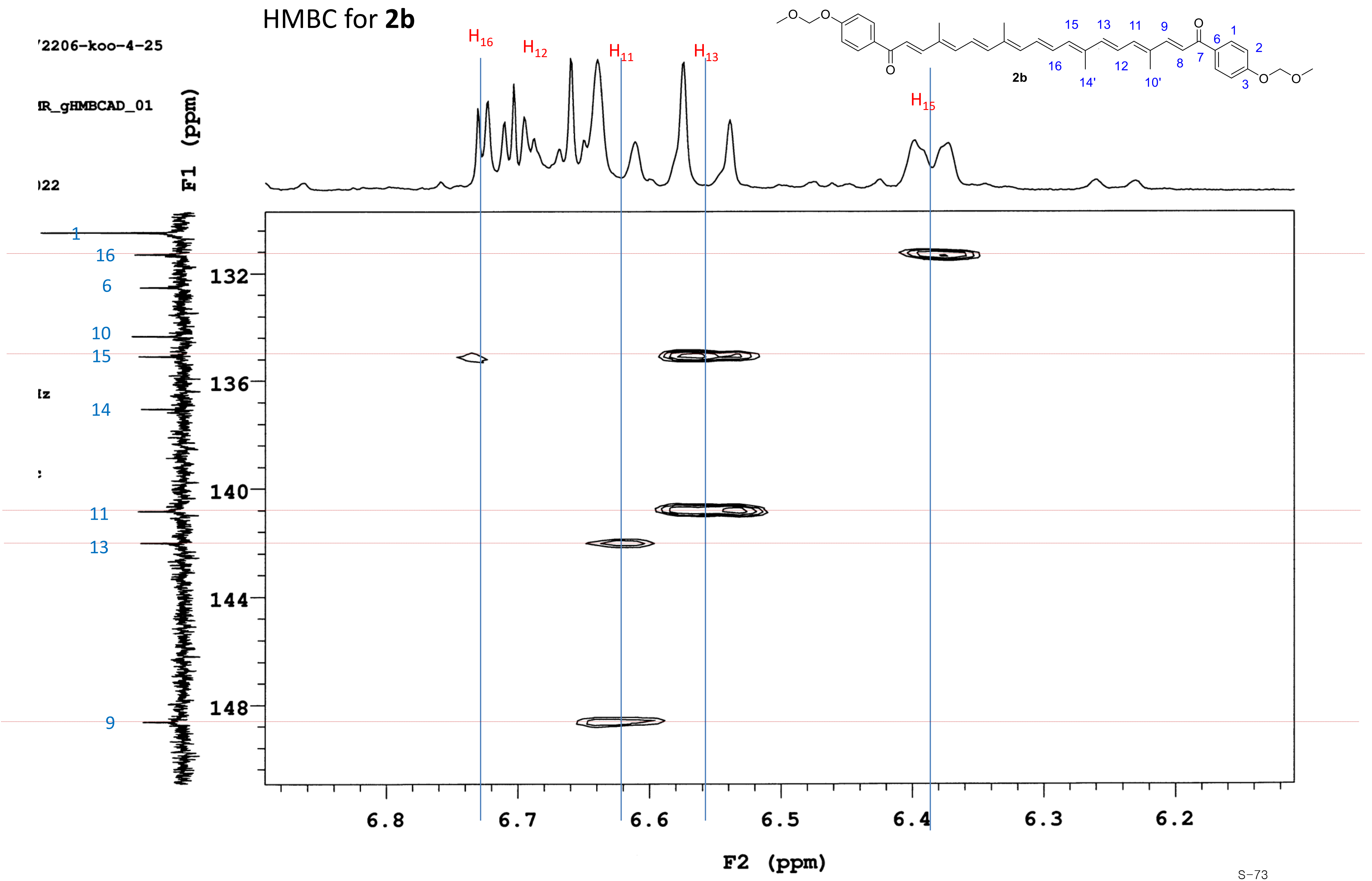

'2206-koo-4-25
IR_gHMBCAD_01
)22
Iz
F1 (ppm)
F2 (ppm)
2b
H16
H12
H11
H13
H15
1
16
6
10
15
14
11
13
9
132
136
140
144
148
6.8
6.7
6.6
6.5
6.4
6.3
6.2

HMBC for **2b**

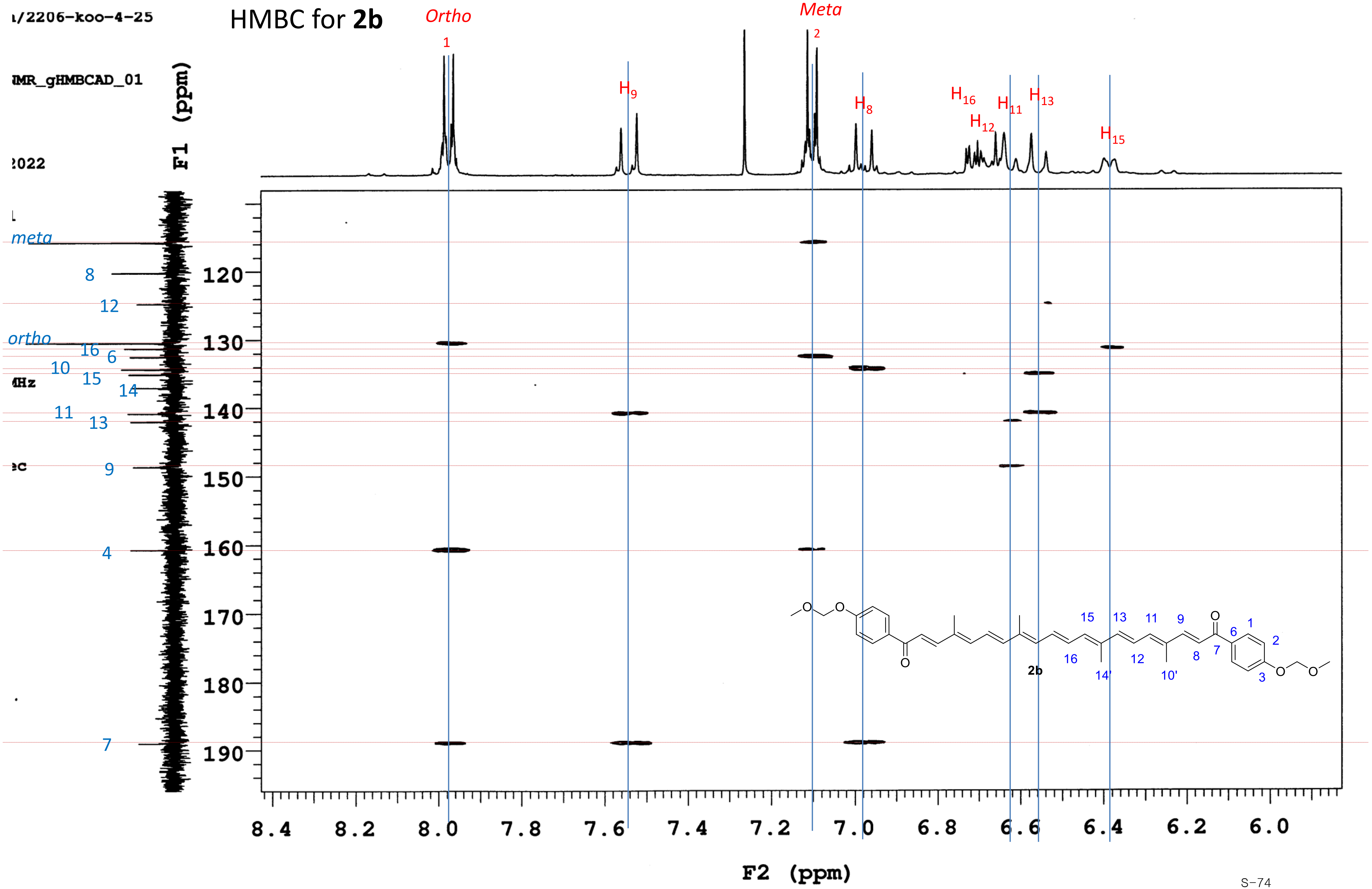

/2206-koo-4-25
MR_gHMBCAD_01
2022
Hz
Ortho
1
Meta
2
H9
H8
H16
H12
H11
H13
H15
F1 (ppm)
meta
8
12
ortho
16
6
10
15
14
11
13
9
4
7
120
130
140
150
160
170
180
190
8.4
8.2
8.0
7.8
7.6
7.4
7.2
7.0
6.8
6.6
6.4
6.2
6.0
F2 (ppm)
2b

^{13}C-NMR for **2b**

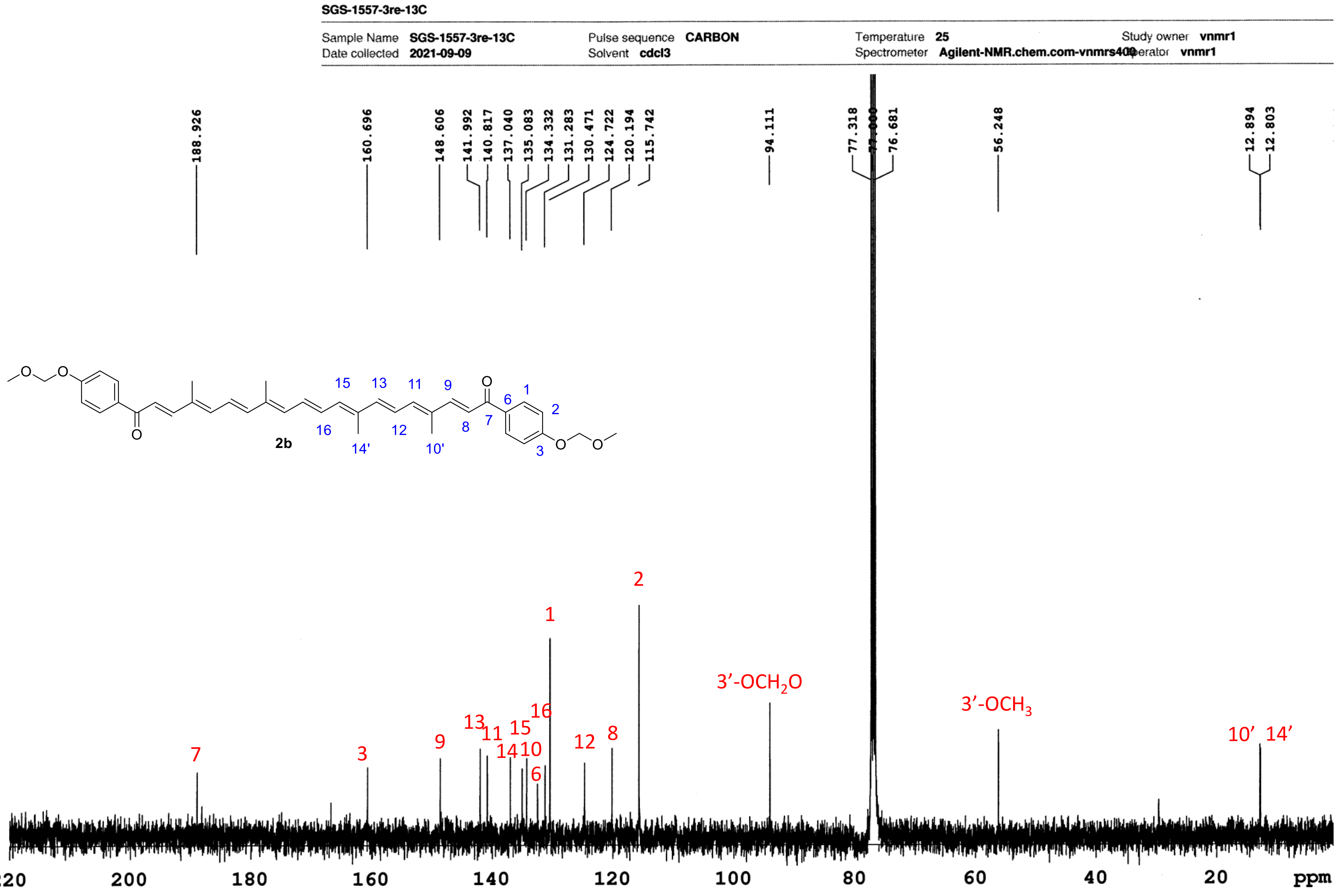

DEPT for **2b**

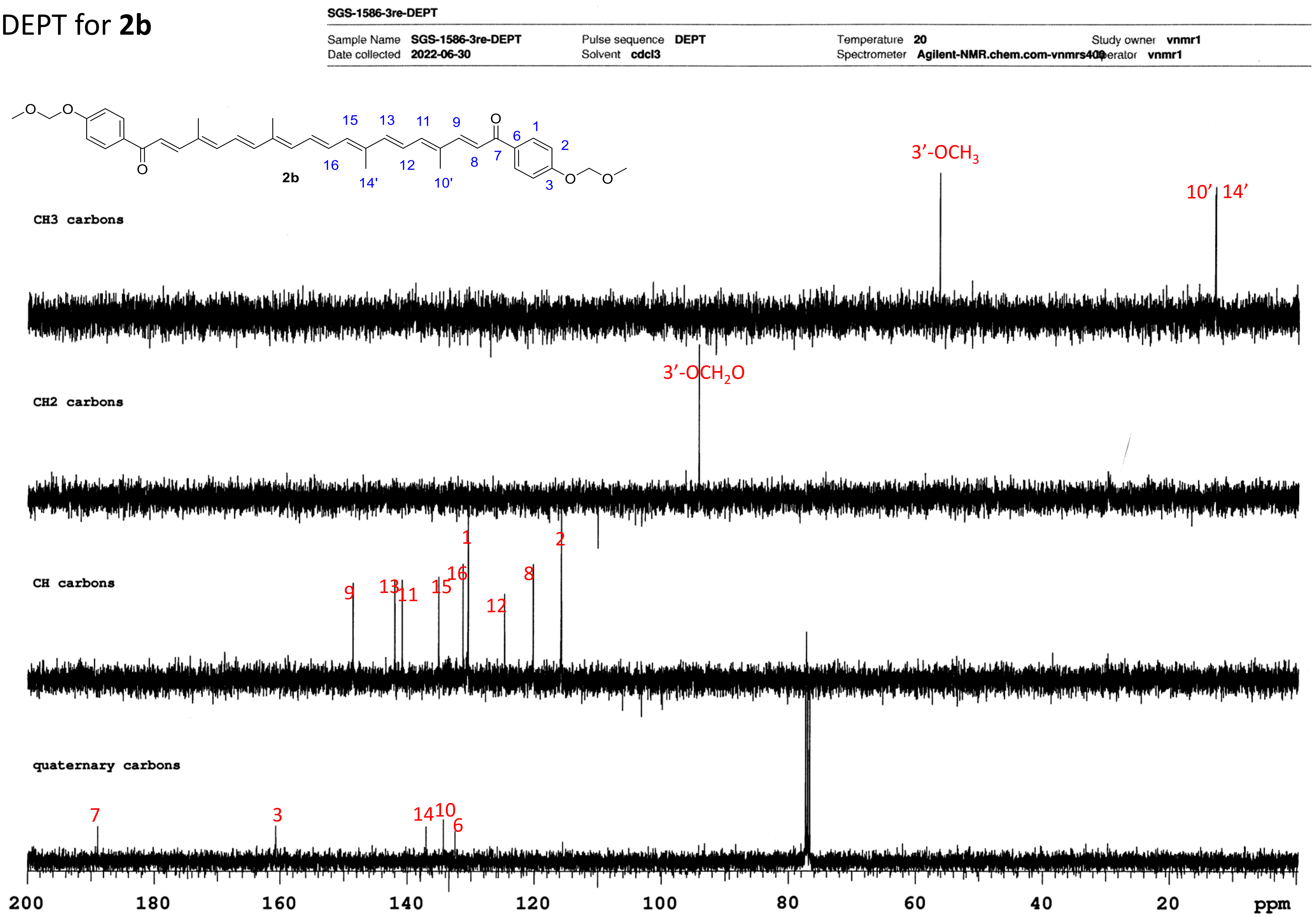
SGS-1586-3re-DEPT
Sample Name SGS-1586-3re-DEPT
Date collected 2022-06-30
Pulse sequence DEPT
Solvent cdcl3
Temperature 20
Spectrometer Agilent-NMR.chem.com-vnmrs400
Study owner vnmr1
Operator vnmr1
2b
15 13 11 9 6 1 2 16 12 8 7 3 14' 10'
CH3 carbons
3'-OCH3
10' 14'
CH2 carbons
3'-OCH2O
CH carbons
1 2 16 8 9 13 11 15 12
quaternary carbons
7 3 14 10 6
200 180 160 140 120 100 80 60 40 20 ppm

DEPT for **2b**

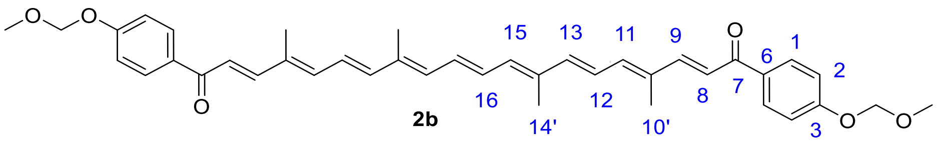

2b
15 13 11 9 1 2 6 7 8 16 12 14' 10' 3

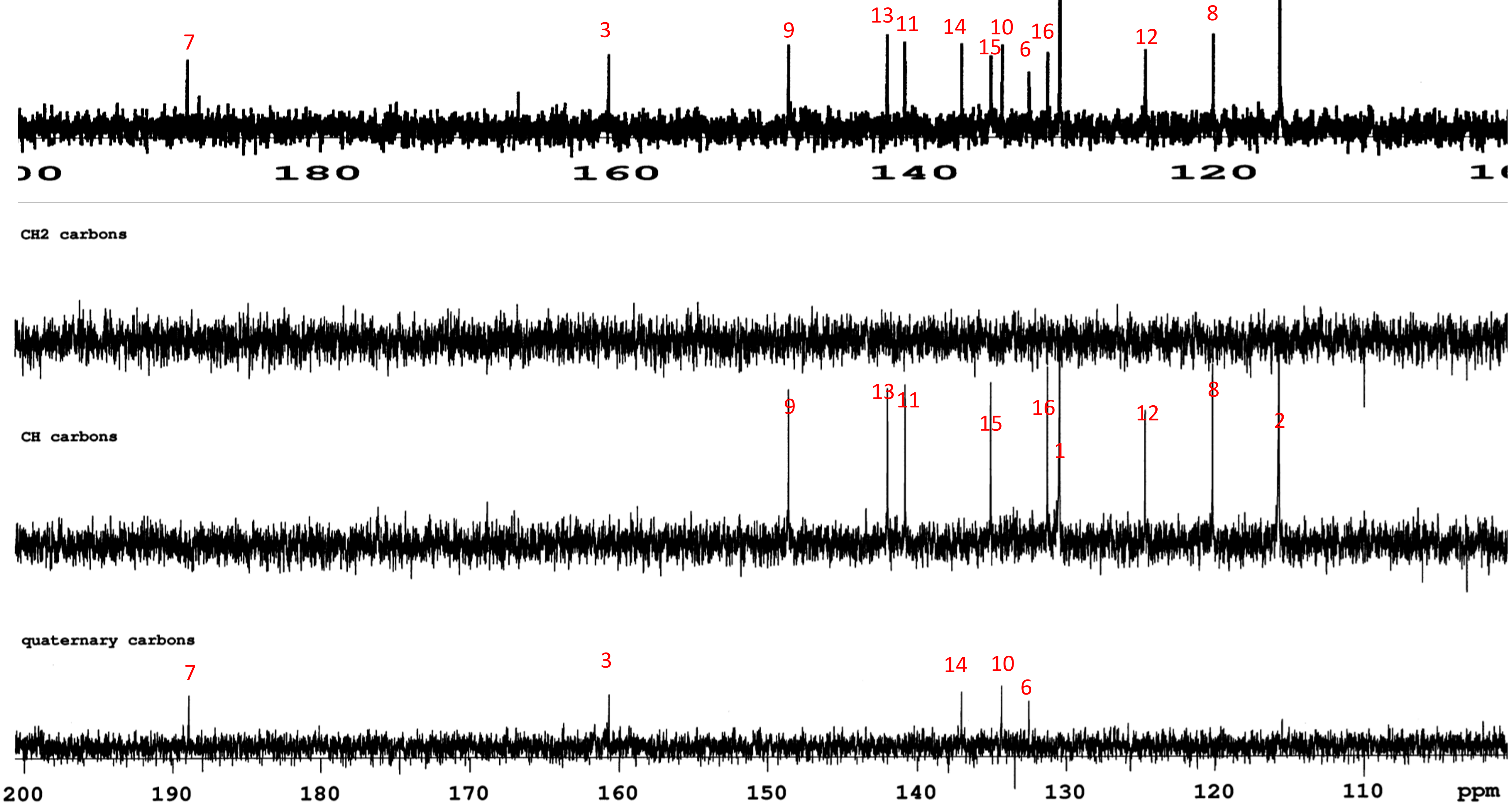

7 3 9 13 11 14 10 15 6 16 1 12 8 2
180 160 140 120
CH2 carbons
CH carbons
9 13 11 15 16 1 12 8 2
quaternary carbons
7 3 14 10 6
200 190 180 170 160 150 140 130 120 110 ppm
